# Proton Irradiation Induces Differential Cellular Responses and Proteomic Signatures in Chondrosarcoma and Chondrocytes

**DOI:** 10.3390/cimb48050450

**Published:** 2026-04-25

**Authors:** Mihaela Tudor, Roxana Cristina Popescu, Benoît Bernay, Mihaela Temelie, Liviu Craciun, Tiberiu Relu Esanu, François Chevalier, Diana Iulia Savu

**Affiliations:** 1 Department of Life and Environmental Physics, Horia Hulubei National Institute for R&D in Physics and Nuclear Engineering, 077125 Magurele, Romania; mihaela.tudor@nipne.ro (M.T.); mihaela.temelie@nipne.ro (M.T.); 2 Faculty of Biology, University of Bucharest, 050095 Bucharest, Romania; 3 Faculty of Medical Engineering, National University of Science and Technology Politehnica Bucharest, Gheorghe Polizu 1–7, 011061 Bucharest, Romania; 4 Plateform Proteogen, US EMerode, Université de Caen Normandie, 14032 Caen, France; benoit.bernay@unicaen.fr; 5 Radiopharmaceutical Research Centre, Horia Hulubei National Institute for R&D in Physics and Nuclear Engineering, 077125 Magurele, Romania; cliviu@nipne.ro (L.C.); tiberiu.esanu@nipne.ro (T.R.E.); 6 Centre de Recherche sur les Ions les Matériaux et la Photonique, Université de Caen Normandie, CEA, CNRS, ENSICAEN, Normandy University, UMR6252 CIMAP, Team Applications in Radiobiology with Accelerated Ions, Bd Becquerel, 14000 Caen, France

**Keywords:** chondrosarcoma, chondrocytes, proton irradiation, proteomics, radioresistance, YAP1 and Fyn signaling

## Abstract

Chondrosarcoma (CHS), the second most common primary malignant cartilage tumor, is largely resistant to conventional therapies, making surgical resection the standard treatment. Proton therapy offers a physical advantage through the Bragg peak, enabling targeted irradiation while sparing surrounding tissues. However, differential biological responses between malignant and normal cartilage cells remain poorly understood. In this study, CHS SW1353 cells and normal chondrocytes (MC615) were exposed to proton irradiation. Biological responses were assessed via clonogenic survival, cell viability, apoptosis (caspase 3/7), micronucleus formation, cell cycle profiling, and oxidative stress markers. Proteomic changes were analyzed using mass spectrometry and bioinformatics. CHS cells exhibited higher radioresistance (D_10_ = 6.45 Gy) than normal chondrocytes (D_10_ = 5.08 Gy), oxidative stress adaptation, G1 arrest and proteomic plasticity, whereas normal chondrocytes displayed increased oxidative stress, extracellular matrix fragility and impaired integrin signaling. Notably, the tumor-specific increased levels of Tyrosine-protein kinase Fyn and Yes1-associated transcriptional regulator (YAP1) signaling suggest molecular drivers of radioresistance. Overall, proton irradiation elicits distinct biological and proteomic responses in malignant versus normal cartilage cells. These findings highlight potential radiosensitization targets, including Fyn/Src and YAP1/Hippo pathways, while underscoring the need to optimize proton therapy to enhance tumor control while minimizing damage to healthy cartilage.

## 1. Introduction

Chondrosarcoma (CHS) represents a primary malignant bone tumor within a heterogeneous group of malignant entities characterized by diverse morphologies, clinical behaviors, and prognoses [1]. These are malignant cartilaginous matrix-producing neoplasms that typically affect adults over 30 and account for approximately 20–30% of all primary bone sarcomas [2].

The cornerstone of treatment remains wide surgical resection; however, the challenging anatomical location and the aggressiveness of certain malignancy subtypes often need adjuvant therapies like chemo- and radiotherapy. Due to CHS resistance, high doses of treatment are recommended, with prognosis being poor for high-grade and dedifferentiated subtypes [3].

Proton therapy, based on the “Bragg peak”, allows for a high-dose delivery to the tumor with a slight effect on the surrounding normal tissue and represents a promising treatment option, especially for skull base and spinal CHS [3,4]. Proton beam therapy has been reported to achieve better tumor control with reduced late toxicity in patients [5,6,7,8]. However, a high dose of postoperative proton irradiation can cause adverse effects. Moreover, accurate treatment planning is complicated by the heterogeneity and variation in the biological effectiveness (RBE) values of protons in tumor and normal cells [9,10]. This biological complexity arises because proton radiation, unlike conventional therapies, induces dense, clustered DNA lesions and a distinct immune-modulatory response, which are critically shaped by the tissue’s specific microenvironment [11,12]. The proton treatment planning of CHS can be refined by integrating spatial and tissue-specific variations in RBE, as well as microenvironmental factors and cellular heterogeneity. In addition, proton therapy can be combined with CHS radiosensitizers to enhance therapeutic outcome. Despite the well-established clinical relevance of proton therapy, only a limited number of preclinical studies address its fundamental cellular biological effects and underlying molecular mechanisms on CHS and its microenvironment cells [13,14,15]. Complex biological responses triggered by proton irradiation require further study for the optimization of clinical proton therapy plans and the protection of normal tissue and biomarker discovery.

Therefore, in this study, we conducted a comprehensive evaluation of the proton irradiation effects on normal mouse chondrocytes (MC615) and malignant human CHS cells (SW1353). MC615 cells serve as a stable and well-characterized non-malignant chondrocytic model, maintaining key cartilage-specific properties [16,17,18], whereas SW1353 cells represent a widely established model for studying radiation biology and DNA damage responses [19,20,21,22,23,24]. A comprehensive profiling of malignant and non-malignant cells focused on survival, genotoxic burden, oxidative stress dynamics, cell cycle alterations, and proteomic remodeling. This integrative approach offers insight into how the physical characteristics of proton irradiation influence cellular behavior. To the best of our knowledge, the differential cellular response of CHS and the surrounding normal cartilage cells to proton irradiation has not yet been comparatively analyzed, despite its relevance for understanding tumor selectivity and normal tissue protection. By characterizing the divergent biological responses between normal and malignant cartilage cells, we aim to identify potential therapeutic targets that could enhance the efficacy of proton therapy while minimizing damage to healthy tissues.

## 2. Materials and Methods

### 2.1. Cell Culture

The SW1353 CHS cell line (CLS Cell Lines Service GmbH, Eppelheim, Germany) and the MC615 chondrocyte cell line (provided by Dr. Jérôme Guicheux, RMeS, UMR 1229, Nantes, France) were utilized in the present study. The MC615 cell line, derived from primary embryonic mouse limb chondrocytes, has been shown in previous studies to exhibit phenotypic and metabolic characteristics typical of chondrocytes, making it a suitable model for studying chondrocyte behavior [25,26]. Cells were cultured in high-glucose Dulbecco’s Modified Eagle’s Medium (DMEM; PAN Biotech, Aidenbach, Germany), supplemented with 10% fetal bovine serum (FBS; EuroClone, Via Figino, Italy), 1% L-glutamine (Sartorius, Beit Haemek, Israel), and 1% penicillin/streptomycin (Capricorn Scientific GmbH, Ebsdorfergrund, Germany). All cultures were maintained at 37 °C in a humidified incubator under an atmosphere of 5% CO_2_.

### 2.2. Cell Irradiation

For proton irradiation, the CHS and chondrocyte cell lines were seeded at a density of 50,000 cells per well in 12-well plates (TPP Techno Plastic Products AG, Trasadingen, Switzerland). Prior to irradiation, plates were sealed with gas-permeable sealing tape (Corning Inc., Corning, NY, USA). Both cell lines were irradiated with 10.6 MeV protons at an LET of 12.6 keV/μm from the IFIN-HH TR-19 Cyclotron (Măgurele, Romania) at a dose-rate of 0.8 Gy/min. The cyclotron accelerates negative ions from an external source of 14–19 MeV. A methyl polymethacrylate (PMMA) filter is used to position samples near the Bragg peak for optimized energy deposition [27]. The selected doses were: 0.1 Gy, 0.5 Gy and 2 Gy. The low-dose conditions (0.1 Gy and 0.5 Gy) were selected based on our previous studies with X-rays and carbon ions [28,29] and with protons (unpublished data) showing that conditioned media from CHS cells irradiated at these doses induces bystander effects in normal cells, underscoring the importance of low-dose-mediated bystander signaling in radiotherapy and radioprotection. The choice of low doses to characterize the bystander effect along with the direct effect in proton therapy is also supported by the literature [30]. Gaining mechanistic insights into proton-induced effects simultaneously in both normal and tumor cells will improve the foundation for strategies aimed at reducing normal tissue toxicity while improving tumor control. The high-dose condition (2 Gy) was selected due to its clinical relevance [5,31]. Assessing proton radiation effects across a dose spectrum ranging from 0.1 to 0.5 Gy to a clinical 2 Gy fraction is essential to characterize the transition from protective hormetic responses and immune activation at low doses to the robust tumoricidal and stress-related effects associated with conventional fractionation [32,33].

### 2.3. Colony Formation Assay

Immediately after irradiation, SW1353 and MC615 cells were detached using a 0.25% Trypsin/0.02% EDTA solution (Capricorn Scientific GmbH, Ebsdorfergrund, Germany), counted in duplicate, and seeded at appropriate densities in 6-well plates with 2 mL of complete medium per well. Cells were incubated for 14 days to allow for colony formation. Subsequently, colonies were fixed, stained and counted as previously described [27,34].

Survival data were fitted to the linear–quadratic model described by the equation ln(SF) = −(αD + βD^2^), where D represents the radiation dose and α and β are fitted parameters. Curve fitting was performed using the non-linear regression tool in SigmaPlot version 15 (Systat Software GmbH, Erkrath, Germany), with an extension developed by Heidelberg University [35].

### 2.4. Micronucleus Assay

For each experimental condition, CHS and chondrocyte cells were seeded at a density of 1 × 10,000 cells on 10 mm coverslips and irradiated as described above. Cells were treated with Cytochalasin B, fixed, and stained with acridine orange as previously described [27,34]. Micronuclei were identified according to the morphological criteria established by Fenech [36]. For each condition, a total of 1000 cells were analyzed per coverslip, using two coverslips per sample.

### 2.5. Cell Viability Assay

Cell viability was assessed five days post-irradiation using the MTT assay. The culture medium was removed and replaced with 0.5 mL/well of a 9:1 mixture of culture medium and MTT solution (5 mg/mL). After 3 h of incubation under standard conditions, the supernatant was discarded and formazan crystals were dissolved in DMSO. A volume of 100 µL from each well was transferred into a 96-well plate (in triplicate), and absorbance was measured at 540 nm using a Mithras plate reader (Berthold Technologies, Bad Wildbad, Germany). Data were analyzed by averaging triplicate wells, subtracting blank values, and normalizing against the non-irradiated control.

### 2.6. Caspase Activity

Caspase-3/7 activity was determined using a chemiluminescent assay kit (G8090 Technical Assay, Promega, Madison, WI, USA), according to the manufacturer’s instructions, 24 h after irradiation. From 12-well plates, a portion of the medium was removed, leaving 300 µL, to which 300 µL of substrate solution mix was added to each well. The mixture was then incubated for 30 min at room temperature in the dark, and luminescence was subsequently measured using a spectrophotometer (Mithras, Berthold Technologies, Bad Wildbad, Germany).

In each experiment, a minimum of three identically treated wells were analyzed. Data analysis involved calculating the arithmetic mean of the three replicates, subtracting the mean value of the blank wells, and expressing the results as fold change relative to the untreated control.

### 2.7. Cell Cycle Analysis

CHS cells and chondrocytes were seeded at 37,500 cells per well prior to exposure. Cells were collected at 3 and 24 h post-irradiation for direct effects. Cells were incubated until collection, trypsinized using 1× trypsin-EDTA, centrifuged at 300× *g* for 5 min at room temperature, and resuspended in cold ethanol (70%). Samples were stored at −20 °C until staining. DNA content was stained with Hoechst 33342 (1 µg/mL) overnight at 4 °C and analyzed using a CytoFLEX flow cytometer (Beckman Coulter, Brea, CA, USA) with CytExpert software (v 2.6.0.105).

### 2.8. Reactive Oxygen Species Measurement

Reactive oxygen species were quantified by flow cytometry using MitoSOX™ Red (M36008, Invitrogen™, Thermo Fisher Scientific, Waltham, MA, USA) for mitochondrial superoxide and CM-H2DCFDA (C6827, Invitrogen™, Thermo Fisher Scientific, Waltham, MA, USA) for total ROS detection. CHS and chondrocyte cells were seeded at 75,000 cells/well and analyzed at 3 and 24 h post-irradiation. After treatment, the medium was replaced, and cells were incubated under standard conditions. Cells were then harvested by trypsinization, centrifuged at 300× *g* for 5 min, and resuspended in Hank’s Balanced Salt Solution (HBSS) containing 5 µM of the respective fluorescent probe. After 15 min of incubation at 37 °C, samples were analyzed using a CytoFLEX flow cytometer according to the manufacturer’s recommended settings.

### 2.9. Protein Extraction

Cells were collected 24 h after irradiation as a dry pellet and stored at −80 °C until protein extraction.

Protein extraction was performed using RIPA buffer (25 mMof Tris-Base, 120 mMof NaCl, 10 mMof Triton X and 1 mMof EDTA), at a volume of approximately 50 µL per one million cells. Samples were incubated on ice for 20 min, followed by mechanical homogenization using a plastic rotor tip for approximately 20 s. After an additional 20 min incubation on ice, samples were centrifuged at 20,000× *g* for 15 min at 4 °C. Supernatants were transferred to fresh tubes and stored at −20 °C. Protein concentrations were determined using the Pierce™ 660 nm Protein Assay Reagent (Thermo Scientific, Waltham, MA, USA), using BSA as standard. Absorbance measurements were performed using an FC Multiskan™ microplate reader (Thermo Scientific, Waltham, MA, USA) and SkanIt software (version RE 6.0.2).

### 2.10. Proteomics Analysis

Proteomic analysis was conducted by TIMS-TOF mass spectrometry (Bruker, Billerica, MA, USA) following the protocol described by Gilbert et al. [29].

For all samples, 5 µg of total protein was processed and subjected to enzymatic digestion with trypsin/Lys-C overnight at 37 °C, generating peptides suitable for MS analysis. Following digestion, peptides were desalted and concentrated using µC18 Omix tips (Agilent Technologies, Santa Clara, CA, USA) prior to nano-LC–MS/MS analysis, ensuring removal of residual contaminants and optimal chromatographic performance. The data-independent acquisition (DIA) scheme comprised 16 variable windows from 400 to 1200 *m*/*z*. Data processing, database searching, and label-free quantification (LFQ) via extracted ion chromatograms (XIC) were performed using DIA-NN (version 1.6.0). An up-to-date UniProt Homo sapiens and Mus musculus protein database was used for in silico library generation and spectral matching. DIA-NN parameters included automatic mass and RT correction, six most intense fragment ions per peptide, 1% FDR at precursor level, and variable modifications: N-terminal acetylation and methionine oxidation. C-propionamide was set as a fixed modification, and trypsin/P was specified as the digestion enzyme. Cross-run normalization was RT-dependent.

Protein digests were analyzed by nano–liquid chromatography coupled with tandem mass spectrometry (nanoLC–MS/MS) operating in DIA mode. DIA was selected to enable reproducible and comprehensive quantitative profiling across all experimental conditions while minimizing the stochastic precursor selection and missing-value issues inherent to data-dependent acquisition (DDA) workflows.

DIA data were acquired using sequential, predefined isolation windows covering the full relevant *m*/*z* range, ensuring systematic fragmentation of all detectable peptide ions. Raw DIA files were processed using a library-free, peptide-centric data analysis strategy. In this approach, peptide identification was performed directly from DIA data without the need for project-specific DDA-derived spectral libraries, thereby avoiding biases related to library completeness and batch-specific DDA sampling.

Protein identification and quantification were performed using a target–decoy-based false discovery rate (FDR) control strategy. FDR thresholds were set at 1% at both the peptide and protein levels. Only proteins meeting these criteria and quantified consistently across biological replicates were retained for downstream analysis. Quantitative values were derived from fragment ion intensities and normalized across samples to account for technical variability.

All comparative analyses were conducted within the same DIA processing pipeline, and radiation-induced proteomic changes were assessed relative to dose-matched, non-irradiated controls for each cell line. This within-condition normalization strategy further reduced the impact of intrinsic baseline differences and ensured the robust detection of irradiation-associated proteomic responses.

The study focused on global protein abundance changes; post-translational modification-specific analyses were not performed.

### 2.11. Functional Analysis of Protein Accessions

Functional enrichment analysis was performed based on the species origin of the cell lines. Identified proteins were mapped using the UniProt database (https://www.uniprot.org, accessed on 6 April 2026). Dose-dependent comparisons of differentially abundant proteins were visualized using Venn diagrams (BioTools.fr, accessed on 12 June 2024). A gene ontology (GO) analysis of biological processes was conducted using the ClueGO plugin in Cytoscape v3.10.4 San Diego, CA, USA [37], with the following settings: medium specificity, *p*-value < 0.05 (Bonferroni correction), GO tree level range 3–8, minimum of three genes per term, and a Kappa score threshold of 1. STRING network visualizations depicted physical and functional associations of proteins, filtered by a minimum confidence score of 0.4. Thick edges represent stronger interactions [38,39,40]. Key hub proteins were identified using the CytoHubba plugin within Cytoscape.

### 2.12. Statistical Analysis

Statistical analysis was performed on data obtained from at least two independent experiments, each with three replicates per condition. Data are presented as mean ± SEM. Clonogenic assay data were analyzed using SigmaPlot, as previously mentioned, while data from other methods were analyzed using OriginPro (version 8.6, OriginLab Corporation, Northampton, MA, USA). Statistical analysis involved comparisons between untreated controls and treated samples using One-Way ANOVA or Two-Way ANOVA, followed by Fisher post hoc test, and Student’s t-test. Two-Way ANOVA for clonogenic assay, micronucleus assay, MTT assay, and apoptosis assay data were also processed using GraphPad Prism (GraphPad Software v 9.1.1, San Diego, CA, USA). Results were considered statistically significant when *p* < 0.05 (*), *p* < 0.01 (**), *p* < 0.001 (***).

Within the proteomic analysis, to assess relative protein abundance between groups, data were analyzed using the R package DEP. Proteins detected in at least two of three replicates per condition were retained. Missing values were imputed using random draws from a manually defined left-shifted Gaussian distribution. Differential enrichment analysis was based on linear modeling with empirical Bayes statistics. Proteins with a fold change ≥ 1.5 and FDR < 0.05 were considered significantly enriched. All data were deposited in iProX public repository database, with the code IPX0014171001 (https://www.iprox.cn, accessed on 1 November 2025).

## 3. Results

This study investigated the biological effects of proton irradiation on CHS tumors using both a tumoral cell line (SW1353) and a normal mouse chondrocyte cell line (MC615). SW1353 was selected because it is a well-characterized, widely used human CHS model with documented relevance for radiation biology studies. MC615 represents a biologically relevant normal tissue reference rather than a strict species-matched control as the aim of this study was not to directly equate human cancer cells with mouse normal cells, but rather to compare malignant versus non-malignant chondrocytic phenotypes under identical irradiation conditions. The analysis assessed the impact of irradiation through multiple parameters, including clonogenic survival, cell death, genotoxic damage, cell cycle alterations, oxidative stress, and proteomic dynamics, providing a comprehensive understanding of the cellular response to proton therapy.

### 3.1. Differential Radiosensitivity and Cell Death Responses

**Figure 1:**
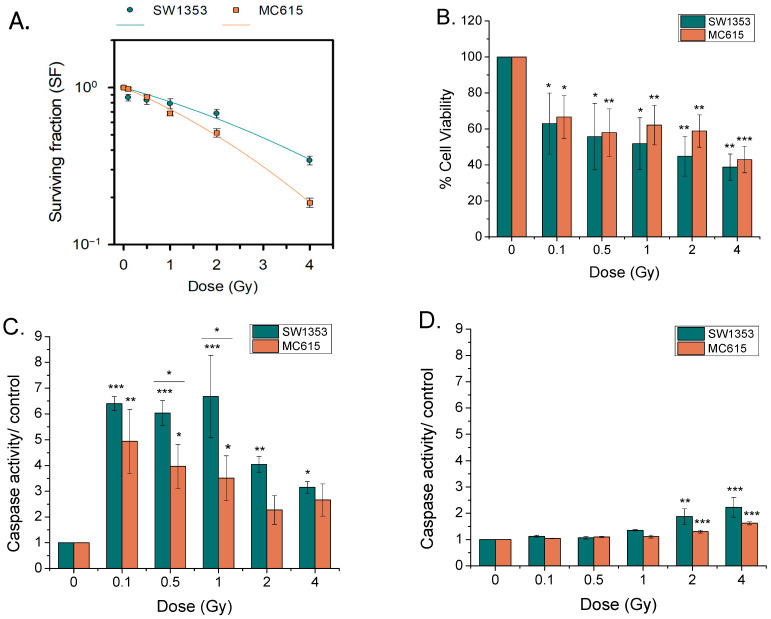
Proton radiation induces cytotoxic effects in SW1353 CHS cells and MC615 chondrocytes. (**A**) dose–response curves for cell survival following clonogenic assay; (**B**) cell viability assessed 5 days post-irradiation using the MTT assay; (**C**,**D**) programmed cell death activation was assessed by measuring caspase-3/7 activity (normalized against each corresponding untreated control) at 3 h (**C**) and 24 h (**D**) post-irradiation. Data are presented as mean ± SEM (*n* = 3), * *p* < 0.05; ** *p* < 0.01; *** *p* < 0.001.

The clonogenic survival of both cell lines (Figure 1A) decreased in a dose-dependent manner (*p*_dose_ < 0.001), the decrease being more pronounced in case of normal chondrocytes than of CHS cells (*p*_cell type_ < 0.001) as shown by Two-Way ANOVA. This result implies that normal MC615 chondrocytes exhibit a greater radiosensitivity than SW1353 CHS cells to proton irradiation as confirmed by their D10 values of 5.08 and 6.45, respectively.

By five days post-irradiation (Figure 1B), viability evaluated by MTT test declined significantly with irradiation doses in both cell types (*p*_dose_ < 0.001).

At 3 h post-irradiation, the caspase 3/7 activity level increased significantly in CHS cells and normal chondrocytes for all the doses used (*p*_dose_ < 0.001), with this effect being more pronounced for lower doses (0.1 Gy, 0.5 Gy, 1 Gy) (Figure 1C). CHS cells seemed to be more prone to the induction of apoptosis than normal chondrocytes (*p*_cell type_ = 0.0012). At 24 h post-irradiation (Figure 1D), both cell types kept an enhanced caspase 3/7 activity only at 2 and 4 Gy relative to controls (*p*_dose_ < 0.001). Again, CHS cells appeared to be more susceptible to apoptosis than normal chondrocytes (*p*_cell type_ = 0.0069).

### 3.2. DNA Damage and Cell Cycle Modulation

**Figure 2:**
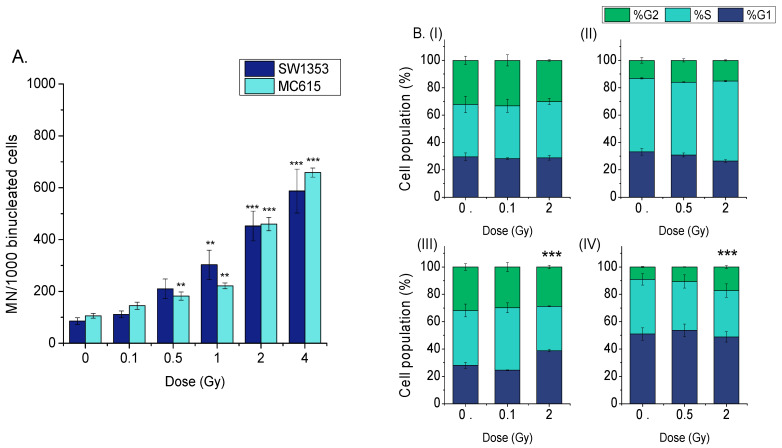
Proton irradiation induced genotoxic alterations and affects cell cycle dynamics in CHS cells and chondrocytes. (**A**) A dose-dependent increase in micronuclei formation was observed in both cell lines. (**B**) Cell cycle analysis of SW1353 (CHS, **I**,**II**) and MC615 (chondrocyte, **III**,**IV**) cells was performed at 3 (**I**,**III**) and 24 (**II**,**IV**) hours post-irradiation, evaluating the distribution of G1, S, and G2 phases. Data are presented as mean ± SEM of a minimum of two experiments, ** *p* < 0.01; *** *p* < 0.001.

A similar dose-dependent induction of DNA damage measured as micronuclei was observed in both cell lines (Figure 2A) as revealed by two-way ANOVA test (*p*_dose_ < 0.001, *p*_cell type_ = non-significant).

Regarding cell cycle distribution, at 3 h post-irradiation (Figure 2B), neither CHS nor chondrocyte cells exhibited significant alterations. However, at 24 h, when comparing to control, CHS cells showed a significant increase in G1 phase arrest at 2 Gy (*p* < 0.001), whereas chondrocytes exhibited a significant increase in G2 phase arrest at the same dose (*p* < 0.001).

These findings indicate distinct radiation-induced cell cycle responses between CHS and chondrocyte cells and varied mechanisms of DNA damage response and repair between normal and tumor cells.

### 3.3. Oxidative Stress Modulation

**Figure 3:**
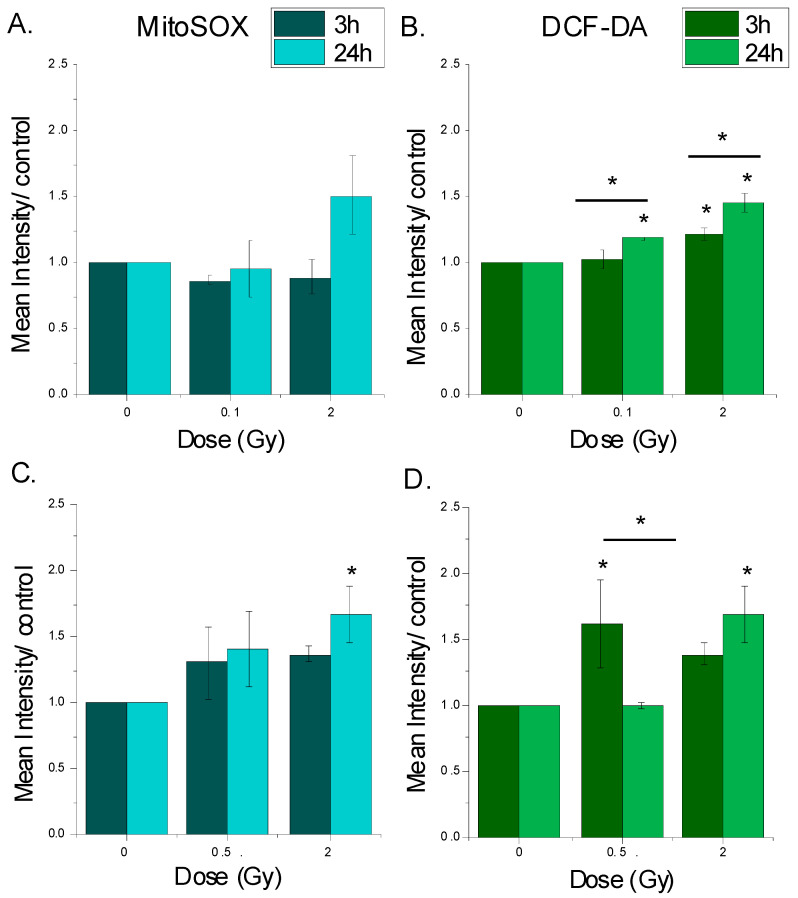
Oxidative stress levels in CHS and chondrocytes cells following irradiation. Effect of irradiation on oxygen reactive species production (ROS) was assessed using MitoSOX (**A**,**C**) and DCF-DA (**B**,**D**) at 3 and 24 h post-irradiation in SW1353 (**A**,**B**) and MC615 (**C**,**D**) cells. Data represent mean ± SEM (*n* = 2); statistical significance was considered at * *p* < 0.05.

Oxidative stress response was evaluated post-irradiation by assessing reactive oxygen species level (Figure 3A–D). At 3 h post-irradiation, mitochondrial ROS levels (Figure 3A,C) remained unchanged in both tumoral and normal cells. However, at 24 h (Figure 3A,C), chondrocytes exhibited an elevation in mitochondrial ROS at 2 Gy (*p* = 0.042), while no significant changes were observed in CHS cells.

In SW1353 cells, intracellular ROS level (Figure 3B) increased at 3 h only following exposure to a high dose of 2 Gy (*p* = 0.014), while at 24 h the ROS level increased for both the low dose of 0.1 and the high dose of 2 Gy (*p* = 0.029 and *p* < 0.001 respectively), and the ROS level was even higher. In chondrocytes, intracellular ROS levels (Figure 3D) increased at 3 h following exposure to a low dose of 0.5 Gy (*p* = 0.037) and at 24 h for 2 Gy (*p* = 0.026).

These results indicate distinct radiation-induced ROS dynamics in CHS and chondrocyte cells.

It is interesting to note that in CHS cells, hydrogen peroxide levels remained elevated for the higher dose even at 24 h, while chondrocytes displayed a fluctuation in oxidative stress with different selected doses. This might suggest a different oxidative stress resistance to proton exposure in accordance with cell type.

### 3.4. Proteomic Profiling

Radiation exposure induces distinct biological responses in CHS cells and normal chondrocytes, affecting several protein accessions and various gene ontology (GO) term-associated pathways. The primary aim of this study was not to compare the absolute proteomic states of the two cell lines, but rather to characterize radiation-induced signaling responses within each cell line relative to its own non-irradiated control. Accordingly, all proteomic and GO enrichment analyses were performed using dose-matched comparisons normalized to the respective baseline (0 Gy) condition for each cell line. This analytical approach minimizes the influence of intrinsic, lineage- or species-specific proteomic differences and focuses on irradiation-driven changes. This study compares the proteomic changes associated with different radiation doses, highlighting key differences between malignant and normal cells.

#### 3.4.1. Quantitative and Qualitative Proteomic Analyses

**Figure 4:**
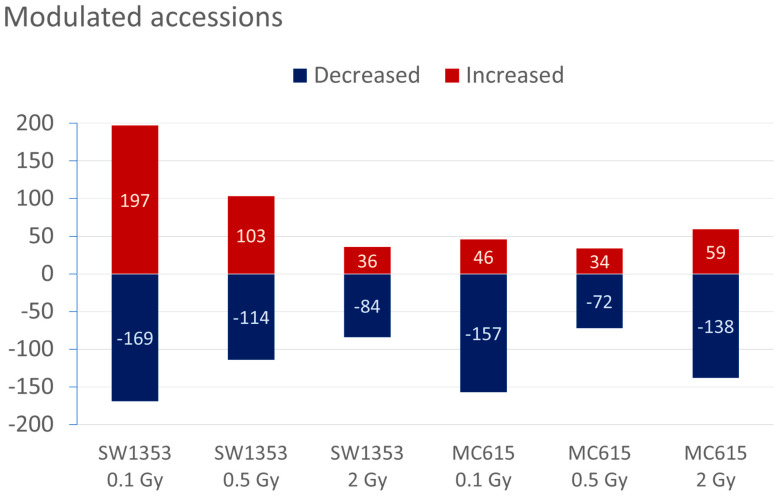
Number of modulated accessions following proton irradiation. The bars indicate the number of proteins with increased or decreased abundance compared to controls 24 h after exposure to protons at the specified doses and in the listed cells. The actual numbers of proteins with altered levels are also displayed on the graph.

Proteomic analysis across different radiation doses provided insights into the molecular adaptations of both cell types, with a total of 8668 and 8340 proteins group analyzed in SW1353 and MC615 cells, respectively (Supplementary Tables S1–S6). In SW1353 cells (Figure 4), exposure to 0.1 Gy resulted in significant proteomic changes, with 366 differentially abundant proteins (169 with decreased and 197 with increased abundance), predominantly involved in metabolic and oxidative stress pathways and adaptive and developmental pathways. At 0.5 Gy, 217 proteins’ abundance was altered (103 with decreased and 114 with increased abundance), with an enrichment of pathways related to DNA repair and mitochondrial energy production. At the highest dose of 2 Gy, we obtained 120 altered proteins (36 with decreased and 84 with increased abundance) involved in cellular transport and organelle membrane maintenance.

In MC615 chondrocytes (Figure 4), exposure to 0.1 Gy altered the abundance of 203 proteins (46 with decreased and 157 with increased abundance) primarily involved in DNA repair and transcriptional regulation. At 0.5 Gy, 106 proteins presented modified abundance (37 with decreased and 72 with increased abundance), with a broad stress response observed but without a dominant pathway being activated. At 2 Gy, 197 proteins were differentially abundant (59 with decreased and 138 with increased abundance) with significant importance for mitochondrial function and protein processing pathways.

##### Highlights on the Most Modulated Accessions Following Irradiation

In SW1353 cells, the protein with the highest decrease in abundance at 0.1 Gy is ciliogenesis and planar polarity effector 1 (Q9H799, CPLN1, −3.5-fold), followed by Thymosin beta-4 (P62328.1, TYB4, 3.19-fold), and an increase was observed in the abundance of the Tyrosine-protein kinase Fyn (P06241, FYN, 6.34 fold) followed by E3 ubiquitin–protein ligase TRIM58 (Q8NG06, 3.15 fold) (Table 1). These proteins are involved in ciliogenesis, cellular polarity, mitochondrial function, and extracellular matrix organization, all critical processes in radiation-induced cellular adaptation. Conversely, FYN and TRIM58 are marked by an increase in abundance, suggesting a key role in signal transduction, cell proliferation, and differentiation [41,42,43].

In MC615 cells, low-density lipoprotein receptor-related protein 2 (A2ARV4, LRP2) showed the greatest decrease in abundance (3.56-fold) at 0.1 Gy, along with Glutamate receptor ionotropic (Q03391, −3.42-fold), in contrast, Metabotropic glutamate receptor 1 (P97772, GRM1, 2.52), Pleckstrin homology domain-containing family G member 2 (Q6KAU7, 2.07-fold) and Desmoplakin (E9Q557, 2.37-fold) showed an increase in abundance (Table 2). These proteins are associated with lipid metabolism, neurotransmission, and cell adhesion, indicating that low-dose irradiation influences pathways related to tissue integrity and cellular communication.

At 0.5 Gy in SW1353 cells, Apolipoprotein L4 (Q9BPW4, APOL4, −3.75-fold) showed a significant decrease in protein abundance, while Tyrosine-protein kinase Fyn again showed an increase in protein abundance (6.2-fold), reinforcing its role in signal transduction and radiation-induced cellular adaptation.

For MC615 cells at 0.5 Gy, Rho family-interacting cell polarization regulator 2 (Q80U16, RIPR2) exhibited the largest decrease in protein abundance (−4.19-fold). Metabotropic glutamate receptor 1 (P97772, 2.25-fold) and Desmoplakin (E9Q557, 2.4-fold) showed an increase in protein abundance similar to the 0.1 Gy condition, suggesting a consistent impact on cell adhesion and signaling pathways across different radiation doses.

At a higher radiation dose of 2 Gy, SW1353 cells exhibit a significant decrease in the abundance of CREB/ATF bZIP transcription factor (Q9NS37, ZHANG, −4.45-fold), as well as a strong increase in the abundance of transcriptional coactivator YAP1 (P46937.1, MAGI1, 4.67-fold), highlighting its role in stress response and gene expression regulation. These findings suggest that high-dose irradiation disrupts protein homeostasis and stress adaptation mechanisms, which may be pivotal in modulating radiation sensitivity.

In MC615 cells, at a dose of 2 Gy, the most decreased protein abundance is shown by BLOC-3 complex member HPS1 (O08983, HPS1, −3.35-fold), while Choline transporter-like protein 3 (Q921V7, CTL3) showed the most increased abundance (4.77-fold). These proteins play key roles in vesicle trafficking and choline transport, indicating significant alterations in cellular transport mechanisms under irradiation stress.

##### Common and Specific Modulated Accessions Between Cell Types and Irradiation Doses

**Figure 5:**
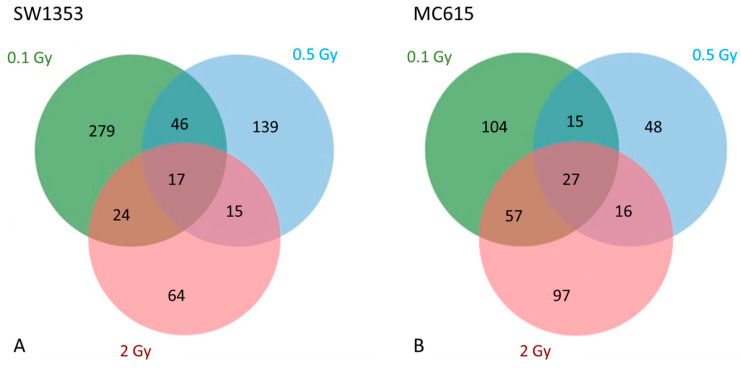
Venn diagram showing the shared proteins with abundance alterations 24 h after proton exposure in SW1353 (**A**) and MC615 (**B**). The numbers inside each section represent the overlaps of significantly altered protein abundances at different doses (0.1 Gy—green, 0.5 Gy—blue, 2 Gy—red).

To identify dose-specific and shared proteomic responses, Venn diagrams (Figure 5) were generated illustrating the overlap of modulated accessions following exposure to all selected doses for each of our cell lines. We observed that the SW1353 cell line presented 17 common proteins across all three doses while MC615 displayed 27 accessions commonly modulated across all doses. After examining the dose-specific responses, notable differences between the two lines were revealed. In CHS, the number of uniquely modulated proteins was strikingly higher, at 0.1 Gy (279 accessions), compared to the other two doses (139 and 64 at 0.5 Gy and 2 Gy, respectively). This suggests a pronounced early dose-specific proteomic response at low radiation exposure, which appears to be dampened at higher doses. However, chondrocytes did not show such a marked disparity among doses. The number of specific accessions modulated at 0.1 Gy (104), 0.5 Gy (48), and 2 Gy (97) were more evenly distributed, indicating a more consistent proteomic profile across radiation levels. This pattern implies that MC615 cell line may employ a more quantitatively buffered response in terms of specific and total number of modulated accessions, in comparison to the sharply dose-sensitive numerical modulation observed in SW1353, although the functional identity of these proteins remains highly dose-specific.

Among overlapping proteins (Figure 5A), SW1353 cells exhibited one protein that consistently presented an increase in abundance across all three radiation doses—Q8IXQ5 (Kelch-like protein 7, KLHL7)—and nine overlapping proteins consistently presented a decrease in abundance: O95716 (Ras-related protein Rab-3D, RAB3D), P10276 (Retinoic acid receptor alpha, RARA), P13647 (Keratin, type II cytoskeletal 5, K2C5), P34932.1 (Heat shock 70 kDa protein 4, HSP74), P51790 (H(+)/Cl(−) exchange transporter 3), P57060 (RWD domain-containing protein 2B, CLCN3), Q5M7Z0 (E3 ubiquitin–protein ligase RNFT1, RNFT1), Q8NCM8 (Cytoplasmic dynein 2 heavy chain 1, DYHC2), and Q93050.1 (V-type proton ATPase 116 kDa subunit a 1, VPP1).

In comparison, MC615 chondrocytes did not exhibit any protein with a consistent increase in abundance across all three doses (Figure 5B). However, 11 proteins consistently showed a decrease in abundance, including F8VQJ3 (Laminin gamma 1), Q2VPQ9 (Chromatin modification-related protein MEAF6, EAF6), A0A5F8MQ70 (Collagen type V), P12032 (Metalloproteinase inhibitor 1, TIMP1), Q6P8H8 (Dolichyl pyrophosphate Glc1Man9GlcNAc2 alpha-1,3-glucosyltransferase, ALG8), Q8BG26 (AP-4 complex accessory subunit RUSC1, RUSC1), Q8BP74 (L-seryl-tRNA(Sec) kinase, PSTK), Q8R5C8 (Zinc finger MYND domain-containing protein 11, ZMY11), Q99LS1 (Cobalamin trafficking protein CblD, MMAD), Q9Z2P8 (vesicle-associated membrane protein 5, VAMP5), and S4R2A9 (Protein transport protein Sec31A).

#### 3.4.2. Gene Ontology Analysis

**Figure 6:**
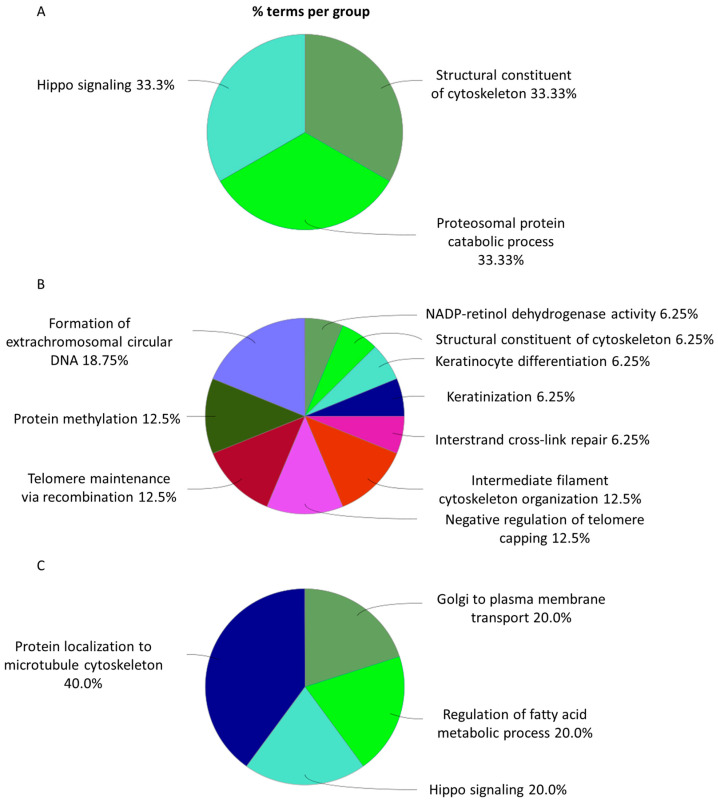
Representation of the proportions of different metabolic pathways in SW1353 cells following proton irradiation, based on GO term enrichment for biological processes at doses of 0.1 Gy (**A**), 0.5 Gy (**B**), and 2 Gy (**C**).

CHS cells exhibit distinct molecular responses to radiation in a dose-dependent manner, with specific biological processes being preferentially activated at different exposure levels. At a low dose of 0.1 Gy (Figure 6A and Table S7), cellular responses are primarily centered on cytoskeletal integrity, proteostasis and Hippo signaling, supporting cell stability, protein homeostasis, and resistance to radiation. At an intermediate dose of 0.5 Gy (Figure 6B and Table S7), biological responses encompass stress adaptation, differentiation, and genomic stability maintenance involving NADP–retinol dehydrogenase activity, keratinization, and DNA repair pathways.

As the radiation dose increases to 2 Gy (Figure 6C and Table S7), the cellular adaptation mechanisms shift toward protein trafficking, metabolic regulation, and further cytoskeletal reorganization.

**Figure 7:**
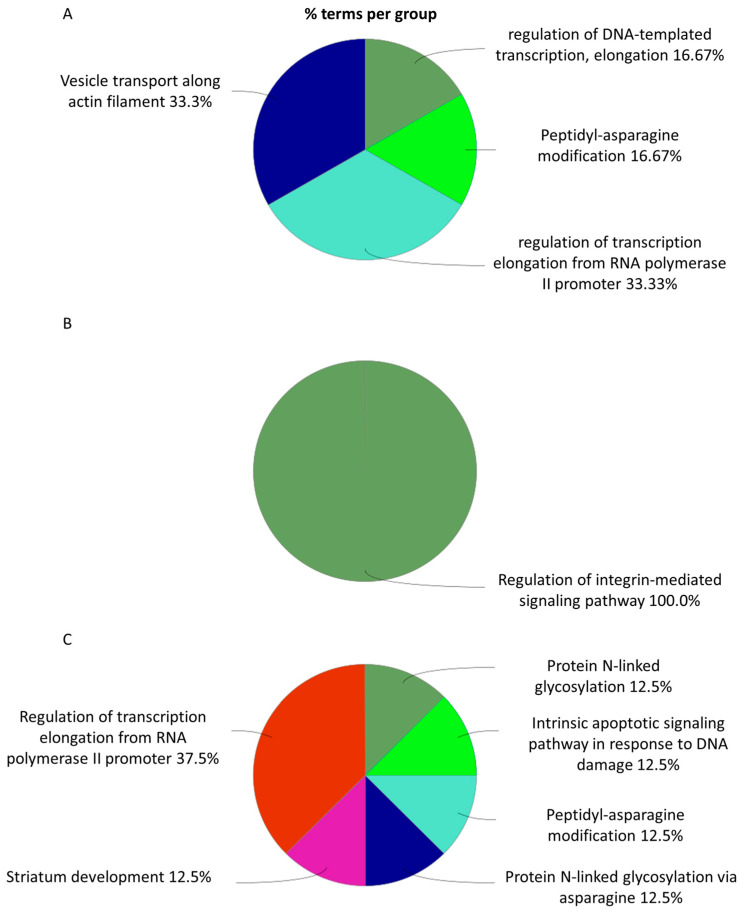
Representation of the proportions of different metabolic pathways in MC615 cells following proton irradiation, based on GO term enrichment for biological processes at doses of 0.1 Gy (**A**), 0.5 Gy (**B**), and 2 Gy (**C**).

Similar dose-dependent biological patterns are observed in MC615 chondrocyte cells (Figure 7A–C). At 0.1 Gy (Figure 7A and Table S8), key processes include DNA recombination, peptidyl–asparagine modification, and transcription elongation from the RNA polymerase II promoter, which are all critical for maintaining genomic integrity and transcriptional fidelity during stress adaptation.

At a moderate dose of 0.5 Gy (Figure 7C and Table S8), modulation of integrin-mediated signaling becomes particularly prominent, emphasizing its role in cell adhesion and interactions with the extracellular matrix, which may enhance resilience against radiation-induced damage.

Exposure to a high dose of 2 Gy (Figure 7B and Table S8) promotes shifts in cellular function, characterized by an increase in the activity of pathways such as protein N-linked glycosylation, intrinsic apoptotic signaling in response to DNA damage, and striatum development, implying alterations in protein processing, activation of programmed cell death, and potential engagement of developmental programs.

#### 3.4.3. Network Analysis of Modulated Accessions

**Figure 8:**
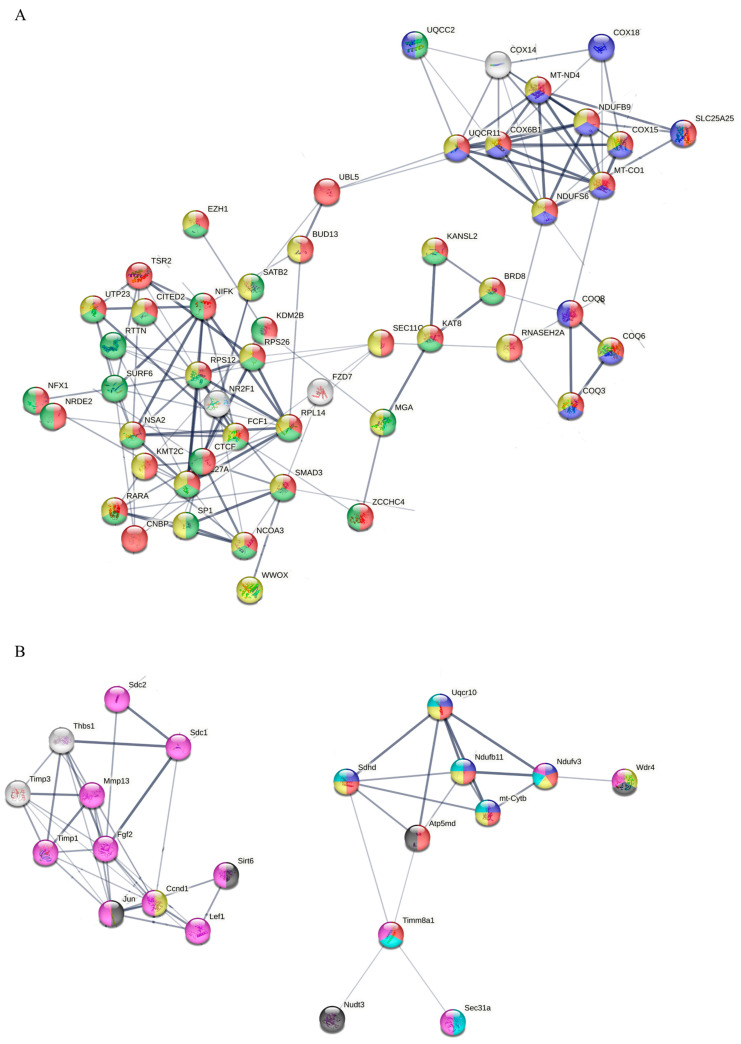
Highlighting the central nodes in the STRING network extracted from Supplementary Figure S1 for (**A**) SW1353 and (**B**) MC615 treated with dose 0.1 Gy. (**A**) Altered proteins are associated with the metabolic process (red), mitochondrial inner membrane (blue), intracellular non-membrane-bound organelle (green), and protein-containing complex (yellow). (**B**) Altered proteins are associated with myosin complex (green), catalytic complex (yellow), mitochondrial protein-containing complex (red), mitochondrial respirasome (blue), transport (cyan), acetylation (black), and phosphoprotein (magenta).

**Figure 9:**
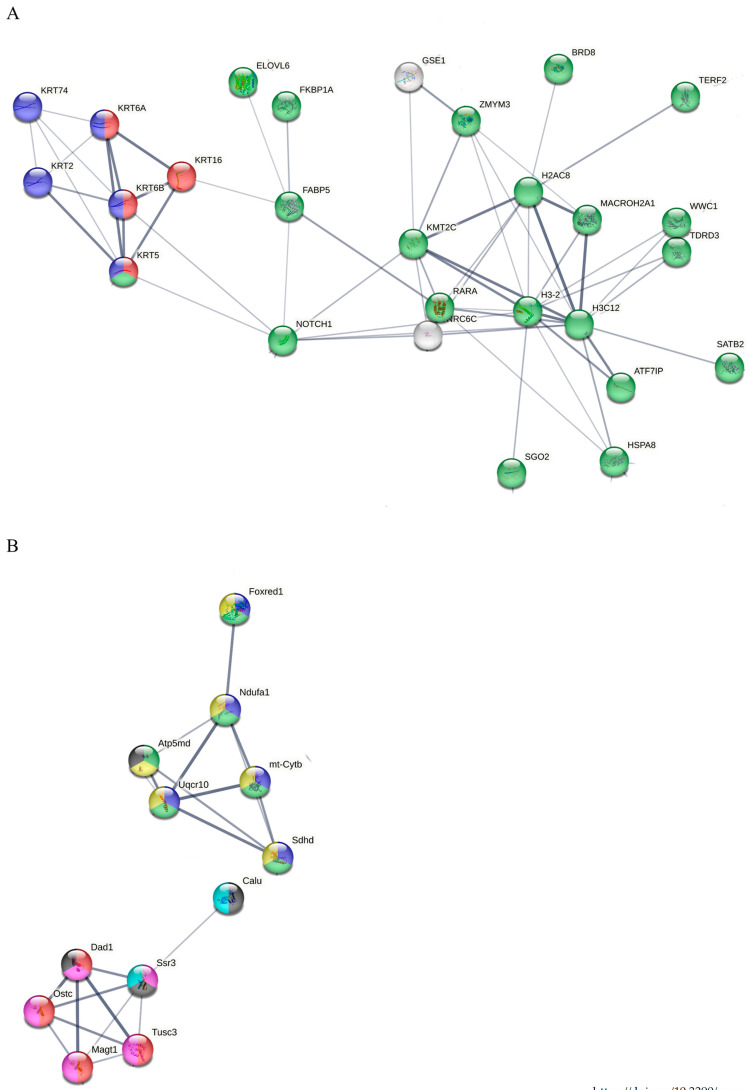
Highlighting the central nodes in the STRING network extracted from Supplementary Figure S1 for (**A**) SW1353 treated with dose 0.5 Gy and (**B**) MC615 treated with dose 2 Gy. (**A**) Altered proteins are associated with intracellular membrane-bounded organelles (green), Pachyonychia congenita and Epidermolysis bullosa simplex Dowling–Meara type (red), and keratin type II head (blue). (**B**) Altered proteins are associated with oligosaccharyl transferase complex (red), mitochondrial protein-containing complex (lime green), mitochondrial inner membrane (yellow), mitochondrial respirasome (blue), acetylation (black), phosphoprotein (cyan), and post-translational protein targeting membrane and translocation (magenta).

STRING network analysis (Supplementary Tables S9 and S10, Figure S1A–F) provided additional insights into the dose-dependent reorganization of protein interaction networks in SW1353 (Supplementary Table S9, Figure S1A–C) and MC615 cells (Supplementary Table S10, Figure S1D–F), revealing distinct patterns of molecular disruption in response to proton irradiation. A selected view of the STRING network for 0.1 Gy and 0.5 Gy conditions is presented in Figure 8 and Figure 9.

In SW1353 cells, low-dose exposure (0.1 Gy, Figure 8A) primarily affected networks associated with mitochondrial function and energy metabolism. Hub proteins such as COX6B1, MT-CO1, and NDUFS6 were central to these interactions, alongside ribosome biogenesis nodes RPL14 and NSA, emphasizing the vulnerability of mitochondrial processes and protein synthesis to early radiation-induced stress.

At an intermediate dose (0.5 Gy, Figure 9B), the proteomic network shifted towards pathways modulating chromatin structure and gene expression. Hub proteins H3-2, H3C12, and KMT2C pointed to disruptions in nuclear organization, transcriptional regulation, and potentially cell differentiation, with additional key proteins (H2AC8, RARA) and multiple keratins (KRT5, KRT6A/B, KRT2, KRT74), indicating a combined effect on both nuclear architecture and cytoskeletal stability.

At a high-dose exposure of 2 Gy, (Supplementary Figure S1C), organelle membrane integrity networks centered on AR and ATXN3 reflected widespread structural damage. Similarly, in MC615 chondrocytes, low-dose exposure (0.1 Gy, Figure 8B) altered proteomic interaction networks primarily related to cell motility, intracellular signaling, and mitochondrial metabolic processes. Hub proteins such as Jun, CCND1, FGF2, and THBS1 emerged as central regulators, suggesting coordinated perturbations in mitochondrial and transport-related functions likely to influence energy flux and motility-associated stress responses.

At 0.5 Gy (Supplementary Figure S1E), the network shifted toward substantial reorganization around vesicular trafficking, and cell cycle regulation. Key hub proteins such as TIMP1, CD63, TSPAN9, LSM5, and PLK4 indicate shifts in membrane-bound signaling, transcriptional control, and proliferative regulation, suggesting that high-dose radiation disrupts structural membrane domains and stress response checkpoints, impairing chondrocyte renewal and adaptive capacity.

High-dose exposure at a dose of 2 Gy (Figure 9B) led to mitochondrial function and post-translational protein regulation, as reflected by hub proteins TUSC3, MAGT1, and SSR3. These proteins are closely associated with protein translocation, oligosaccharyl transferase complex function, and mitochondrial inner membrane components, emphasizing a heightened reliance on protein processing and energy management in response to moderate radiation-induced stress.

## 4. Discussion

The effects of particle therapy on normal cell biological responses relative to tumor cells are of particular clinical interest in treatment planning [9,10] and developing combined modalities that specifically radiosensitize radioresistant tumors such as CHS [44]. Therefore, this study focused on performing a comparative integrated biological analysis of proton irradiation effects in SW1353 CHS cells and normal MC615 chondrocytes, revealing consistent differences in radiosensitivity, cell-cycle responses, oxidative stress handling, and proteome remodeling.

One important finding is that MC615 chondrocytes are more radiosensitive than SW1353 tumor cells (D_10_ = 5.08 Gy vs. 6.45 Gy). As expected, this outcome mirrors broader evidence that CHS displays intrinsic resistance to radiotherapy. The mechanisms of CHS radioresistance are complex and not completely understood. However, several mechanisms are considered to drive radioresistance, namely efficient DNA damage responses, adaptive stress programs, hypoxia metabolism, specific genetic mutations and epigenetic changes in CHS cells, which can alter cell cycle regulation, apoptosis pathways, and metabolic processes, leading to a low percentage of dividing cells and poor vascularity [3,45]. Several studies demonstrated a heterogenous response of CHS cell lines to ionizing radiation, probably due to genetic alterations [25,46,47,48], revealing that the SW1353 CHS cell line is very radioresistant [47,49]. The difference in radiosensitivity to proton irradiation of SW1353 cells versus MC615 cells is difficult to explain. Since a similar level of DNA damage was noticed in both cell types following proton irradiation (Figure 2A), the DNA repair capability could not be considered to contribute to their different sensitivities to proton irradiation. Similarly, Girard et al. showed that different cell lines of CHS exhibited different sensitivities to X-ray irradiation while observing a similar level of DNA damage in these lines, suggesting that the cell response variability is triggered by intrinsic factors [48]. Surprisingly, CHS cells exhibited higher caspase activity levels compared to MC615 cells, which typically suggests increased susceptibility to apoptosis relative to chondrocytes. However, it is now increasingly recognized that caspase activation does not invariably result in cell death, as these proteases also participate in a range of non-apoptotic processes [50], supporting adaptive survival pathways during mild cellular stress [51,52].

The divergent cell-cycle arrests between cell lines that we observed—G1 accumulation in SW1353 and G2 arrest in MC615 (Figure 2B)—suggest distinct checkpoint strategies: tumor cells appear to pause earlier to facilitate repair before replication, whereas normal chondrocytes accumulate in G2, consistent with the activation of damage checkpoints after unresolved double-strand breaks, revealing the involvement of different DNA repair mechanisms. Various reports showed altered cell cycle arrest and DNA repair activation following proton irradiation of CHS cells [14] or other types of cells [53,54,55]. Most of these studies demonstrated a significant arrest of the cells in G2/M [14,54,56]. However, Keta et al. showed a G1 arrest of lung cancer cells at 24 h post-proton irradiation, similarly to our results on CHS cells, obtained at the same time point, while at 48 h, proton irradiation increased the number of cells in the G2/M phase [53].

The distinct ROS dynamics between SW1353 and MC615 cells (Figure 3) highlight differences in oxidative stress management. Chondrocytes exhibited a significant increase in mitochondrial oxidative stress and total intracellular ROS, consistent with a lower antioxidant defense system and mitochondrial vulnerability. In contrast, CHS cells maintained more stable ROS levels, suggesting the engagement of redox-buffering mechanisms and mitochondrial resilience/adaptation. Mitochondria dysfunction could lead not only to apoptosis but also to other types of cell death (necroptosis, ferroptosis, and premature senescence) [57,58] that were not measured in our study. It is well known that proton radiation-induced ROS plays an important role in inducing tumor cytotoxicity [59,60], but on the other hand, tumor cells can better adapt to genotoxic challenge [61].

Quantitative proteomics highlighted a key finding: the repeated increase in the abundance of Fyn (a Src-family protein kinase) and YAP1 (Hippo pathway effector) in SW1353. The increased abundance of these two molecules is consistent with a pro-survival/radioresistant phenotype. Fyn/Src family kinases is expressed in many cancers, including CHS, and promotes cancer growth and metastasis by multiple biological functions, such as cell growth, survival, adhesion, cytoskeletal dynamics, motility and metabolic pathways that contribute to therapy resistance [62,63]. Specifically, these kinases function as central signaling hubs that orchestrate the degradation of the extracellular matrix, thereby accelerating the transition from a localized to a metastatic phenotype [63,64,65]. Van Oosterwijk et al. demonstrated that Fyn is linked to cancer growth, chemoresistance and migration in CHS [41]. Several reports showcased that Src-family kinases, via downstream effectors (PI3K/AKT, ERK), can modulate the radiosensitivity of different tumors to proton irradiation or photon irradiation [66,67,68,69]. YAP1 is a key effector molecule of the Hippo signaling pathway and a transcriptional coactivator, essential in controlling cell and tissue homeostasis [70]. Activated or overexpressed YAP leads to tumorigenesis, proliferation, invasion, migration [71,72,73,74], drug resistance [75] of multiple type of malignancies, including CHS. Hippo pathway is also linked to radioresistance [76,77,78,79]. In addition, it was demonstrated that by using YAP1 inhibitors, the radiosensitivity of different cancer types was increased [76,80]. To our knowledge, this is the first study revealing the enhancement of Fyn and YAP1/Hippo pathway in CHS cells exposed to radiation, suggesting the role of these molecules in the observed radioresistance of CHS. These findings extend previous transcriptomic observations showing that proton irradiation induces pathway-specific remodeling in CHS, including alterations in chromatin remodeling, energy metabolism, and cytoskeletal organization [14]. Notably, our results highlight that normal chondrocytes lack the proteomic flexibility required for sustained stress adaptation, which may explain their higher radiosensitivity under proton exposure.

Proteomic profiling coupled with STRING database analysis reveals the dose-dependent remodeling of functional protein networks in both cell types. In SW1353 CHS cells, low-dose (0.1 Gy) exposure disrupts mitochondrial function and energy metabolism, while a moderate dose (0.5 Gy) shifted the response towards chromatin organization and gene expression regulation, culminating in organelle membrane disintegration at high doses (2 Gy). One key finding regarding the chromatin regulation in chondrosarcoma after proton exposure is the increase in the abundance of histone methyltransferase KMT2C. KMT2C, described in breast cancers, is stabilized by secernin-2 SCRN2, drives histone 3, lysine 4 H3K4 monomethylation and enhancer activation, potentially linked to YAP1-dependent transcription [81,82,83] and collectively consistent with ATM/ATR-mediated DNA damage responses. Another important outcome is the intensified organelle membrane disruption that places the ubiquitin-specific peptidase Ataxin-3 (ATXN3) at the center of cellular stress response after high-dose proton exposure (2 Gy). The increase in ATXN3 abundance supports autophagy initiation and stabilizes oncogenic drivers such as YAP1 and ZEB1 [84,85], indicating the activation of survival, repair, and trafficking pathways. Collectively, these shifts imply the existence of a coordinated membrane, Golgi trafficking, and cytoskeletal signaling programs in CHS’s high-dose radiation adaptation. Similarly, MC615 chondrocytes demonstrate a progressive reorganization of proteomic networks with an increase in dose. MC615 networks progressed from motility/energy modules (0.1 Gy) to vesicular trafficking and cell-cycle nodes (0.5 Gy), and finally to mitochondrial/post-translational regulation (2 Gy). It seems that proton irradiation triggers sequential proteomic adaptation that fundamentally differs between tumor and normal cartilage cells. These findings are consistent with the cellular stress-response to varying doses of irradiation described in the literature [86]. At 0.1 Gy, the cell focuses on energy modulation with the enrichment of hub proteins such as Jun, CCND1 and FGF2, indicating a prioritization of energy flux and transcriptional fidelity to maintain genomic integrity during initial stress adaptation. As the dose increases to 0.5 Gy, the cell shifts focus to structural reorganization and membrane-bound signaling. The emergence of hub proteins like TIMP1, CD63 and PK4, alongside the modulation of integrin-mediated signaling, suggests a massive effort to stabilize the extracellular matrix and cell adhesion, enhancing resilience against radiation-induced damage through substantial vesicular trafficking. Finally, at 2 Gy, the process culminates in post-translational protein regulation and mitochondrial function alterations, reflected by hub proteins such as TUSC3, MAGT1 and SSR3. At this stage, the cell must execute complex protein N-linked glycosylation and protein translocation to manage misfolded proteins, eventually engaging in intrinsic apoptotic signaling if these structural repair mechanisms are overwhelmed.

The differential biological and proteomic responses observed here have important implications for clinical proton therapy. While protons offer superior dose conformity via the Bragg peak, the molecular vulnerability of normal cartilage emphasizes the need to optimize fractionation and LET distribution to minimize collateral damage. The tumor-specific activation of Fyn and YAP1 identifies potential radiosensitization targets, particularly within Src-family kinases and the Hippo signaling axis, which could enhance CHS control when combined with proton therapy. The inhibition of Src/Fyn and YAP1/Hippo pathway has been shown to radiosensitize other tumor cells [63,87], suggesting Fyn and Yap1 are promising radiosensitization targets that warrant the functional validation of CHS cells.

Especially at low-dose irradiation, when metabolic and oxidative stress were modulated, targeting the key nodes that regulate oxidative stress represents a promising therapeutic strategy [88].

Despite all our important findings, our study has some limitations. For example, only canonical proteins, rather than proteoforms, were identified. The use of cell lines derived from different species may limit the comparability and translational relevance of the results. Nevertheless, our conclusions are not based on direct quantitative cross-species comparisons, but rather on qualitative differences in radiation response patterns between malignant and non-malignant chondrocyte cells. Another important limitation is the reliance on a single cell line for each condition. The well-characterized CHS SW1353 was selected to investigate the mechanistic aspects of radiation-induced responses within a controlled and reproducible experimental system, rather than to capture the full extent of intertumoral heterogeneity. However, the inclusion of additional tumor cell lines would be necessary to enhance the generalizability of the results. Other limitations include the in vitro setting using monoculture 2D models, as well as the lack of a parallel 24 h viability check for proteomic analysis, using a single LET, limited timepoints and also the correlative nature of proteomics.

However, these data provide a strong basis for further functional validation and radiosensitization strategies. Future studies should validate these findings in multiple human cell models and 3D tissue/co-culture models and explore the impact of proton irradiation with expanded different LET values along the Bragg curve and with ultra-high dose-rate (FLASH) proton exposure. This may reveal qualitatively different biological behaviors and therapeutic windows. Future studies should integrate parallel viability assays and time-resolved proteomic analyses. The integration of proteomic data with transcriptomic and metabolomic profiles will deepen mechanistic understanding and aid in developing precision proton therapy strategies tailored to cartilage malignancies.

## 5. Conclusions

Proton therapy induces significant biological effects in both normal chondrocytes and CHS cells, with distinct, cell type-specific responses. CHS cells display increased radioresistance as compared to normal chondrocytes. Moreover, CHS cells showcase oxidative stress control, G1 phase arrest and adaptive proteomic remodeling, while chondrocytes show ECM-dependent vulnerability. Proteomic analyses highlighted differences in mitochondrial metabolism, nuclear organization, and integrin signaling. These results emphasize the need for personalized proton therapy to optimize tumor control while protecting healthy tissues and point to the proteomic landscape as a source of targets to enhance CHS radiosensitivity.

## Figures and Tables

**Table 1:** List of the most modulated proteins in the SW1353 chondrosarcoma cell proteome irradiated, with protons at 0.1, 0.5 and 2 Gy (selected from Supplementary Materials, Tables S1, S3 and S5).

Entry SW1353 0.1 Gy	Gene Name	Fold	Entry SW1353 0.5 Gy	Gene Name	Fold	Entry SW1353 2 Gy	Gene Name	Fold
Q9H799	CPLN1	−3.5	Q9BPW4	APOL4	−3.75	Q9NS37	ZHANG	−4.45
P62328.1	TYB4	−3.19	Q15147	PLCB4	−2.51	P34931.1	HS71L	−3.76
P03905	NU4M	−2.67	P51790	CLCN3	−2.38	Q9C0K3	ARP3C	−3.08
Q96R72	OR4K3	−2.67	Q96R72	OR4K3	−2.37	Q9H799	CPLN1	−3.07
P24043	LAMA2	−2.61	Q9BZ81	MAGB5	−2.34	Q93050.1	VPP1	−2.58
P57678	GEMI4	−2.51	Q6IA69	NADE	−2.33	P34932.1	HSP74	−2.26
Q9Y6M9	NDUB9	−2.3	P02533.2	K1C14	−2.25	O95716	RAB3D	−2.19
O43731.1	ERD23	−2.22	O95716	RAB3D	−2.24	P12273	PIP	−2.16
Q5M7Z0	RNFT1	−2.15	Q8N8Y2	VA0D2	−2.14	Q02548	PAX5	2.08
Q8N8E3	CE112	−2.12	P34932.1	HSP74	−2.12	P46937.1	YAP1	4.67
Q93050.1	VPP1	−2.1	P01034	CYTC	−2.11			
Q0VGL1	LTOR4	−2.08	P04259	KRT6B	−2.02			
O95716	RAB3D	−2.07	Q9NWD8	TM248	2.06			
Q13255	GRM1	−2.01	Q02509	OC90	2.12			
A0A590UJ08	DLG1	2	Q8NB66	UN13C	2.14			
O75683	SURF6	2.04	Q562F6	SGO2	2.15			
Q96E29	MTERF3	2.21	Q8TE73	DYH5	2.25			
Q9H223.3	EHD4	2.22	P11586.1	C1TC	2.41			
O60890	OPHN1	2.24	Q6VMQ6	MCAF1	2.71			
P14854	COX6B1	2.48	Q8IX03	KIBRA	3.27			
P68371	TUBB4B	2.81	Q70UQ0	IKIP	4.12			
P02795.1	MT2A	2.83	Q7RTS7	K2C74	4.42			
Q8NG06	TRIM58	3.15	P06241.2	FYN	6.2			
P06241.2	FYN	6.34						

**Table 2:** List of the most modulated proteins in the MC615 chondrocyte cell proteomes irradiated with protons at 0.1, 0.5 and 2 Gy (selected from Supplementary Materials, Tables S2, S4 and S6).

Entry MC615 0.1 Gy	Gene Name	Fold	Entry MC615 0.5 Gy	Gene Name	Fold	Entry MC615 2 Gy	Gene Name	Fold
A2ARV4	LRP2	−3.56	Q80U16	RIPR2	−4.19	O08983	HPS1	−3.35
Q03391	NMDE4	−3.42	B7ZMV8	B7ZMV8	−4.03	Q6PE84	STML3	−2.72
Q8BVF4	CCD30	−2.76	A2ARV4	LRP2	−3.32	Q9ESD7	DYSF	−2.57
Q9JHQ0	ANXA9	−2.5	Q9D1D1	TSN11	−2.06	P28666	MUG2	−2.28
P15655	FGF2	−2.45	Q8BP74	PSTK	−2.01	Q6URW6	MYH14	−2.27
P28666	MUG2	−2.44	P97772	GRM1	2.25	G3X9Y5	G3X9Y5	−2.17
P54265	DMPK	−2.39	E9Q557	DESP	2.4	A0A286YDA5	A0A286YDA5	−2.07
Q6URW6	MYH14	−2.35				P00158	CYB	−2.06
G3X9Y5	G3X9Y5	−2.28				A2AQ07	TBB1	−2.01
Q03517	SCG2	−2.2				Q8BP74	PSTK	−2.01
Q8BP74	PSTK	−2.08				Q5DTN8	JKIP3	3.19
Q9D1D1	TSN11	−2.06				Q921V7	CTL3	4.77
P00158	CYB	−2.01						
Q6KAU7	PKHG2	2.07						
E9Q557	DESP	2.37						
P97772	GRM1	2.52						

## Data Availability

All proteomic data have been deposited in iProX public repository database, with the code IPX0014171001 (https://www.iprox.cn), accessed in 1 November 2025.

## Supplementary Materials

The following supporting information can be downloaded at: https://www.mdpi.com/article/10.3390/cimb48050450/s1.

## Abbreviations

The following abbreviations are used in this manuscript:

ALG8	Dolichyl pyrophosphate Glc1Man9GlcNAc2 α-1,3-glucosyltransferase
APOL4	Apolipoprotein L4
ANOVA	Analysis of Variance
ATM	Ataxia-Telangiectasia Mutated
ATR	Ataxia Telangiectasia and Rad3-Related
ATXN3	Ataxin-3
BSA	Bovine Serum Albumin
CCND1	Cyclin D1
CHS	Chondrosarcoma
CPLN1	Ciliogenesis and Planar Polarity Effector 1
CTL3	Choline transporter-like protein 3
D10	Dose reducing survival to 10%
DCF-DA	2′,7′-Dichlorofluorescin diacetate
DYHC2	Cytoplasmic dynein 2 heavy chain 1
DIA	Data-Independent Acquisition
DIA-NN	Data-Independent Acquisition Neural Network
DMEM	Dulbecco’s Modified Eagle Medium
DMSO	Dimethyl sulfoxide
DSB	Double-Strand Break
EDTA	Ethylenediaminetetraacetic acid
FBS	Fetal Bovine Serum
FDR	False Discovery Rate
FGF2	Fibroblast Growth Factor 2
FYN	Tyrosine–protein kinase Fyn
GO	Gene Ontology
GRM1	Glutamate Metabotropic Receptor 1
Gy	Gray
HBSS	Hanks’ Balanced Salt Solution
HSP74	Heat shock 70 kDa protein 4
KLHL7	Kelch-Like Protein 7
LET	Linear Energy Transfer
LFQ	Label-Free Quantification
LRP2	Low-density lipoprotein receptor-related protein 2
MAGI1	Membrane-Associated Guanylate Kinase, WW and PDZ Domain Containing 1
MeV	Mega-electron Volt
MTT	3-(4,5-Dimethylthiazol-2-yl)-2,5-diphenyltetrazolium bromide
NADP	Nicotinamide adenine dinucleotide phosphate
NSA	Nucleolar protein associated with ribosomal assembly
PMMA	Polymethyl methacrylate
PSTK	Phosphoseryl-tRNA Kinase
RARA	Retinoic Acid Receptor Alpha
RIPA	Radioimmunoprecipitation Assay buffer
ROS	Reactive Oxygen Species
RT	Retention Time
SCRN2	Secernin-2
SEM	Standard Error of the Mean
SF	Survival Fraction
STRING	Search Tool for the Retrieval of Interacting Genes/Proteins
TIMS-TOF	Trapped Ion Mobility Spectrometry—Time of Flight
TIMP1	Tissue Inhibitor of Metalloproteinases 1
TRIM58	Tripartite motif-containing protein 58
VAMP5	Vesicle-Associated Membrane Protein 5
VPP1	V-type proton ATPase 116 kDa subunit a 1
YAP1	Yes-Associated Protein 1
XIC	Extracted Ion Chromatogram
