# Supplementary material for: Proton Irradiation Induces Differential Cellular Responses and Proteomic Signatures in Chondrosarcoma and Chondrocytes
Source: Curr Issues Mol Biol. 2026 Apr 25;48(5):450. doi: 10.3390/cimb48050450 (PMC13204733; doi:10.3390/cimb48050450)
Supplement: Supplementary file 1 [file cimb-48-00450-s001.zip › cimb-4242040-supplementary.pdf]

current issues in molecular biology

MDPI

Article—Supplementary Material

Proton Irradiation Induces Differential Cellular Responses and Proteomic Signatures in Chondrosarcoma and Chondrocytes—Supplementary Material

Table S1. List of the 366 modified proteins in the SW1353 chondrosarcoma cell proteome irradiated with protons 12.6 at a dose of 0.1 Gy.

Entry	Fold	P value	Entry Name	Protein name
Q9H799	-3.5	4.25E-13	CPLN1	Ciliogenesis and planar polarity effector 1
P62328.1	-3.19	0.0283	TYB4	Thymosin beta-4
P03905	-2.67	0.034	NU4M	NADH-ubiquinone oxidoreductase chain 4
Q96R72	-2.67	0.000972	OR4K3	Olfactory receptor 4K3
P24043	-2.61	0.00193	LAMA2	Laminin subunit alpha-2
P57678	-2.51	0.0272	GEMI4	Gem-associated protein 4
Q9Y6M9	-2.3	0.035	NDUB9	NADH dehydrogenase [ubiquinone] 1 beta subcomplex subunit 9
O43731.1	-2.22	4.82E-12	ERD23	ER lumen protein-retaining receptor 3
Q5M7Z0	-2.15	4.25E-13	RNFT1	E3 ubiquitin–protein ligase RNFT1
Q8N8E3	-2.12	0.00124	CE112	Centrosomal protein of 112 kDa
Q93050.1	-2.1	6.71E-07	VPP1	V-type proton ATPase 116 kDa subunit a 1
Q0VGL1	-2.08	5.95E-07	LTOR4	Ragulator complex protein LAMTOR4
O95716	-2.07	1.97E-06	RAB3D	Ras-related protein Rab-3D
Q13255	-2.01	0.000102	GRM1	Metabotropic glutamate receptor 1
P06753.2	-1.86	1.06E-09	TPM3	Tropomyosin alpha-3 chain

 https://doi.org/10.3390/cimb48050450

Q15147	-1.85	0.0347	PLCB4	1-phosphatidylinositol 4,5-bisphosphate phosphodiesterase beta-4
P34932.1	-1.77	0.00316	HSP74	Heat shock 70 kDa protein 4
Q9UPQ8	-1.72	0.000104	DOLK	Dolichol kinase
Q9Y4C4	-1.71	0.000116	MFHA1	Malignant fibrous histiocytoma-amplified sequence 1
Q8WVI0	-1.65	7.54E-12	UQCC5	Small integral membrane protein 4
Q86UD1	-1.56	0.000147	OAF	Out at first protein homolog
O60941	-1.46	0.014	DTNB	Dystrobrevin beta
Q9UPP5	-1.46	2.24E-05	BTBD8	AP2-interacting clathrin-endocytosis protein
P51790	-1.45	3.39E-07	CLCN3	H(+)/Cl(-) exchange transporter 3
Q13936	-1.43	4.14E-05	CAC1C	Voltage-dependent L-type calcium channel subunit alpha-1C
Q92766	-1.4	0.0073	RREB1	Ras-responsive element-binding protein 1
O95678.8	-1.35	0.0248	K2C75	Keratin, type II cytoskeletal 75
Q9BQ13	-1.32	0.00149	KCD14	BTB/POZ domain-containing protein KCTD14
Q9BRU9	-1.32	7.39E-06	UTP23	rRNA-processing protein UTP23 homolog
O00213	-1.3	0.000862	APBB1	Amyloid beta precursor protein binding family B member 1
Q13615.1	-1.3	0.0461	MTMR3	Myotubularin-related protein 3
Q8NCM8	-1.29	4.25E-13	DYHC2	Cytoplasmic dynein 2 heavy chain 1
Q6KCM7	-1.26	0.00541	SCMC2	Calcium-binding mitochondrial carrier protein SCaMC-2
H0YJS1	-1.24	0.000227	H0YJS1	Echinoderm microtubule-associated protein-like 5
Q6ZMR3	-1.24	0.0103	LDH6A	L-lactate dehydrogenase A-like 6A
Q9BT88	-1.24	1.16E-09	SYT11	Synaptotagmin-11
Q14114	-1.23	0.0044	LRP8	Low-density lipoprotein receptor-related protein 8
Q14147	-1.23	0.0387	DHX34	Probable ATP-dependent RNA helicase DHX34

P17097	-1.21	0.0125	ZNF7	Zinc finger protein 7
P51784.1	-1.21	5.43E-06	UBP11	Ubiquitin carboxyl-terminal hydrolase 11
P53667.1	-1.2	0.00172	LIMK1	LIM domain kinase 1
P63313	-1.2	4.21E-07	TYB10	Thymosin beta-10
A6NJ78	-1.18	3.13E-05	MET15	12S rRNA N4-methylcytidine (m4C) methyltransferase
P18085.2	-1.18	0.000239	ARF4	ADP-ribosylation factor 4
P57060	-1.17	5.63E-07	RWD2B	RWD domain-containing protein 2B
Q8TB24	-1.17	0.0155	RIN3	Ras and Rab interactor 3
B7Z8V7	-1.16	3.81E-02	B7Z8V7	NAD kinase 2, mitochondrial
P10276	-1.16	0.00231	RARA	Retinoic acid receptor alpha
Q8IXV7	-1.16	0.0107	KLD8B	Kelch domain-containing protein 8B
H7C3M7	-1.15	0.00185	H7C3M7	FERM, ARHGEF and pleckstrin domain-containing protein 2
O95819.3	-1.15	0.0192	M4K4	Mitogen-activated protein kinase kinase kinase kinase 4
Q13402	-1.14	0.000451	MYO7A	Unconventional myosin-VIIa
Q9NU23	-1.14	0.00506	LYRM2	LYR motif-containing protein 2
P0CAP1	-1.12	0.0274	MYZAP	Myocardial zonula adherens protein
Q8NHM5.1	-1.12	0.0343		Lysine-specific demethylase 2B
Q99967	-1.12	0.0157	CITE2	Cbp/p300-interacting transactivator 2
P0CAP2	-1.1	0.048	GRL1A	DNA-directed RNA polymerase II subunit GRINL1A
Q92804	-1.1	0.0486	RBP56	TATA-binding protein-associated factor 2N
Q969K3	-1.1	0.00231	RNF34	E3 ubiquitin–protein ligase RNF34
Q9NQC8	-1.1	0.00374	IFT46	Intraflagellar transport protein 46 homolog
Q96JI7	-1.08	0.0206	SPTCS	Spatacsin
Q86T82	-1.07	0.029	UBP37	Ubiquitin carboxyl-terminal hydrolase 37

P10276.1	-1.06	0.0061	NR2F6	Retinoic acid receptor alpha
B5MCW3	-1.05	0.0037	B5MCW3	Bromodomain-containing protein 8
P31321	-1.05	0.00407	KAP1	cAMP-dependent protein kinase type I-beta regulatory subunit
P36575	-1.05	0.0146	ARRC	Arrestin-C
Q8NI60	-1.05	0.0106	COQ8A	Atypical kinase COQ8A, mitochondrial
Q9C0D7	-1.05	2.34E-06	ZC12C	Probable ribonuclease ZC3H12C
Q9UBS9	-1.05	0.0428	SUCO	SUN domain-containing ossification factor
Q92994	-1.04	0.0157	TF3B	Transcription factor IIIB 90 kDa subunit
A0A3B3ISE1	-1.03	0.00306	A0A3B3ISE1	Uncharacterized protein C9orf153
O00712.1	-1.03	0.000941	NFIB	Nuclear factor 1 B-type
P07437.5	-1.03	0.000186		Tubulin beta chain
Q8IWI9	-1.03	0.0344	MGAP	MAX gene-associated protein
A1L020	-1.02	0.0392	MEX3A	RNA-binding protein MEX3A
Q8TBZ3	-1.02	0.000187	WDR20	WD repeat-containing protein 20
P13647	-1.01	0.00348		Keratin, type II cytoskeletal 5
Q8IUH8	-1.01	0.0145	SPP2C	Signal peptide peptidase-like 2C
O15013.1	-0.99	0.00267	ARHGA	Rho guanine nucleotide exchange factor 10
Q6N043	-0.979	0.00791	Z280D	Zinc finger protein 280D
Q70EL1	-0.978	2.57E-10	UBP54	Inactive ubiquitin carboxyl-terminal hydrolase 54
P58511	-0.975	0.00735	SIM11	Small integral membrane protein 11
Q7KZN9	-0.973	2.83E-08	COX15	Cytochrome c oxidase assembly protein COX15 homolog
O95258.1	-0.972	0.0175	UCP5	Brain mitochondrial carrier protein 1
Q92539	-0.97	0.000945	LPIN2	Phosphatidate phosphatase LPIN2
Q92546	-0.97	4.75E-05	RGP1	RAB6A-GEF complex partner protein 2
Q00536	-0.943	0.0346	CDK16	Cyclin-dependent kinase 16

Q8TDY8	-0.942	0.0144	IGDC4	Immunoglobulin superfamily DCC subclass member 4
Q8WTW4	-0.935	0.00441	NPRL2	GATOR complex protein NPRL2
Q16611	-0.929	0.000414	BAK	Bcl-2 homologous antagonist/killer
Q6YBV0	-0.928	0.0349	S36A4	Proton-coupled amino acid transporter 4
Q53HV7	-0.922	3.32E-05	SMUG1	Single-strand selective monofunctional uracil DNA glycosylase
Q9UBH6	-0.912	5.56E-07	S53A1	Xenotropic and polytropic retrovirus receptor 1
Q13129	-0.901	0.00776	RLF	Zinc finger protein Rlf
G3V5R9	-0.884	0.0426	G3V5R9	Kinesin light chain
Q07617	-0.882	0.0134	SPAG1	Sperm-associated antigen 1
Q6BDS2	-0.882	0.00097	BLT3A	UHRF1-binding protein 1
Q9UKT7	-0.881	0.0258	FBXL3	F-box/LRR-repeat protein 3
O60337	-0.876	0.0123	MARH6	E3 ubiquitin-protein ligase MARCHF6
O00559	-0.871	0.00211	RCAS1	Receptor-binding cancer antigen expressed on SiSo cells
P05976	-0.868	0.0164	MYL1	Myosin light chain 1/3, skeletal muscle isoform
Q9H5U6	-0.864	0.0423	ZCHC4	rRNA N6-adenosine-methyltransferase ZCCHC4
P25054	-0.861	3.40E-07	APC	Adenomatous polyposis coli protein
P40425	-0.858	0.00497	PBX2	Pre-B-cell leukemia transcription factor 2
A0A5F9W493	-0.856	0.0494	A0A5F9W493	Casein kinase 1, alpha 1, isoform CRA_d
Q16829	-0.847	0.0215	DUS7	Dual specificity protein phosphatase 7
Q86SG6	-0.847	0.0126	NEK8	Serine/threonine-protein kinase Nek8
E5RHB1	-0.843	0.0326	E5RHB1	EF-hand calcium-binding domain-containing protein 3
Q5BKY9	-0.834	0.00867	F133B	Protein FAM133B
Q9UBU6	-0.831	0.0368	FA8A1	Protein FAM8A1

Q9BY50	-0.818	0.0363	SC11C	Signal peptidase complex catalytic subunit SEC11C
A0A5F9ZHS3	-0.814	0.00141	A0A5F9ZHS3	Menin
Q9NUV7	-0.803	0.000417	SPTC3	Serine palmitoyltransferase 3
P00395	-0.802	0.00504	COX1	Cytochrome c oxidase subunit 1
O43734	-0.792	0.00293	CIKS	E3 ubiquitin ligase TRAF3IP2
P68366.1	-0.791	0.00301	TBA4A	Tubulin alpha-4A chain
Q12851	-0.788	0.00406	M4K2	Mitogen-activated protein kinase kinase kinase kinase 2
Q15506	-0.784	0.04	SP17	Sperm surface protein Sp17
Q96G74	-0.781	0.013	OTUD5	OTU domain-containing protein 5
J3KSE9	-0.779	0.00992	J3KSE9	N-acetyltransferase 9
Q8WVR3	-0.778	0.0444	TPC14	Trafficking protein particle complex subunit 14
Q9Y2C2	-0.773	0.00321	UST	Uronyl 2-sulfotransferase
P10588.1	-0.771	0.00163	COT1	Nuclear receptor subfamily 2 group F member 6
O43164	-0.769	0.0401	PJA2	E3 ubiquitin–protein ligase Praja-2
O14957	-0.756	0.00747	QCR10	Cytochrome b-c1 complex subunit 10
P31749.1	-0.756	0.00146	AKT1	RAC-alpha serine/threonine-protein kinase
P49842	-0.756	0.00142	STK19	Serine/threonine-protein kinase 19
Q9BX63	-0.75	0.0223	FANCJ	Fanconi anemia group J protein
P22748	-0.748	0.0211	CAH4	Carbonic anhydrase 4
P40424.1	-0.748	0.00894	PBX1	Pre-B-cell leukemia transcription factor 1
Q9NRD1	-0.745	3.38E-06	FBX6	F-box only protein 6
Q17R98	-0.74	1.46E-05	ZN827	Zinc finger protein 827
O75879	-0.735	0.000341	GATB	Glutamyl-tRNA(Gln) amidotransferase subunit B, mitochondrial
Q53H80	-0.734	0.00789	AKIR2	Akirin-2
Q07955.1	-0.713	0.00526	SRSF1	Serine/arginine-rich splicing factor 1

Q8N8Q8	-0.709	0.0048	COX18	Cytochrome c oxidase assembly protein COX18, mitochondrial
Q96EE4	-0.709	0.00456	CC126	Coiled-coil domain-containing protein 126
Q96MG8	-0.69	0.00657	PCMD1	Protein-L-isoaspartate O-methyltransferase domain-containing protein 1
Q96NL6	-0.674	0.0415	SCLT1	Sodium channel and clathrin linker 1
O15014	-0.657	0.000392	ZN609	Zinc finger protein 609
P62079	-0.653	0.0138	TSN5	Tetraspanin-5
O14735	-0.652	0.000323	CDIPT	CDP-diacylglycerol--inositol 3-phosphatidyltransferase
Q96I36	-0.65	0.026	COX14	Cytochrome c oxidase assembly protein COX14
Q9UK73	-0.65	0.0116	FEM1B	Protein fem-1 homolog B
O43768	-0.649	0.000841	ENSA	Alpha-endosulfine
Q9UPZ3	-0.649	0.00117	HPS5	BLOC-2 complex member HPS5
Q8N4A0	-0.64	0.0123	GALT4	Polypeptide N-acetylgalactosaminyltransferase 4
Q86WQ0	-0.639	0.00766	NR2CA	Nuclear receptor 2C2-associated protein
O00258	-0.637	0.00679	GET1	Guided entry of tail-anchored proteins factor 1
E5RG95	-0.63	0.0292	E5RG95	Beta-enolase
Q6NWY9	-0.63	0.0264	PR40B	Pre-mRNA-processing factor 40 homolog B
Q8IVE3	-0.63	0.0392	PKHH2	Pleckstrin homology domain-containing family H member 2
Q9H7Z3	-0.63	0.00252	NRDE2	Nuclear exosome regulator NRDE2
Q92610	-0.628	0.00135	ZN592	Zinc finger protein 592
Q9BRD0	-0.627	0.000526	BUD13	BUD13 homolog
P24863	-0.625	0.019	CCNC	Cyclin-C
Q8TE04.1	-0.621	0.00573	PANK1	Pantothenate kinase 1
Q92889	-0.617	0.00767	XPF	DNA repair endonuclease XPF
Q53F19	-0.615	6.20E-10	NCBP3	Nuclear cap-binding protein subunit 3

Q86VV8	-0.612	0.0249	RTTN	Rotatin
Q9NP92	-0.604	0.00672	RT30	39S ribosomal protein S30, mitochondrial
Q5JS37	-0.603	0.00465	NHLC3	NHL repeat-containing protein 3
Q8N584	-0.601	5.64E-05	TT39C	Tetratricopeptide repeat protein 39C
P17707	-0.596	0.03	DCAM	S-adenosylmethionine decarboxylase proenzyme
Q5VW38	-0.596	0.000229	GP107	Protein GPR107
P62854	-0.59	0.0198	RS26	40S ribosomal protein S26
Q96JC9	-0.59	5.61E-06	EAF1	ELL-associated factor 1
Q8N6H7.1	-0.589	2.56E-06	ARFG2	ADP-ribosylation factor GTPase-activating protein 2
Q15633	-0.585	5.04E-05	TRBP2	RISC-loading complex subunit TARBP2
P51572	0.589	9.49E-05	BAP31	B-cell receptor-associated protein 31
P52848.1	0.59	0.00324	NDST1	Bifunctional heparan sulfate N-deacetylase/N-sulfotransferase 1
Q9UGP4	0.592	0.00102	LIMD1	LIM domain-containing protein 1
Q86Y13	0.593	0.00726	DZIP3	E3 ubiquitin–protein ligase DZIP3
Q9P2X0	0.593	0.00456	DPM3	Dolichol-phosphate mannosyltransferase subunit 3
Q96L58	0.597	0.000203	B3GT6	Beta-1,3-galactosyltransferase 6
P50914	0.605	0.00847	RL14	60S ribosomal protein L14
O00308.1	0.607	0.0233	WWP2	NEDD4-like E3 ubiquitin-protein ligase WWP2
P46776	0.609	0.00221	RL27A	60S ribosomal protein L27a
Q15555.1	0.609	0.00129	MARE2	Microtubule-associated protein RP/EB family member 2
Q96AJ9	0.609	0.000102	VTI1A	Vesicle transport through interaction with t-SNAREs homolog 1A
P29992.2	0.614	9.26E-07	GNA11	Guanine nucleotide-binding protein subunit alpha-11
P34741	0.615	0.0218	SDC2	Syndecan-2

O75380	0.619	9.93E-07	NDUS6	NADH dehydrogenase [ubiquinone] iron-sulfur protein 6, mitochondrial
P84022	0.619	0.000454	SMAD3	Mothers against decapentaplegic homolog 3
P0CG38.5	0.627	0.0234	POTEI	POTE ankyrin domain family member I
Q15418	0.629	0.0252	KS6A1	Ribosomal protein S6 kinase alpha-1
O75190.1	0.63	7.53E-05	DNJB6	DnaJ homolog subfamily B member 6
Q9BRT2	0.633	0.0202	UQCC2	Ubiquinol-cytochrome-c reductase complex assembly factor 2
P68366.3	0.638	0.027	TBB4B	Tubulin alpha-4A chain
Q6IQ49	0.642	0.0481	SDE2	Replication stress response regulator SDE2
Q5SVS4	0.644	0.0381	KMCP1	Kidney mitochondrial carrier protein 1
O75792	0.646	0.0171	RNH2A	Ribonuclease H2 subunit A
Q99439	0.649	0.0128	CNN2	Calponin-2
Q9UIL1	0.649	0.00852	SCOC	Short coiled-coil protein
F8VXL3	0.652	0.0206	F8VXL3	Myosin light polypeptide 6
Q9BTL3	0.654	2.14E-08	RAMAC	RNA guanine-N7 methyltransferase activating subunit
Q2TAZ0	0.655	0.0263	ATG2A	Autophagy-related protein 2 homolog A
Q9NP97	0.655	0.000584	DLRB1	Dynein light chain roadblock-type 1
Q9H1K1	0.657	0.0258	ISCU	Iron-sulfur cluster assembly enzyme ISCU, mitochondrial
A6NHL2.1	0.664	0.0208	TBAL3	Tubulin alpha chain-like 3
P45877	0.667	0.00651	PPIC	Peptidyl-prolyl cis-trans isomerase C
Q9BZL1	0.672	4.17E-08	UBL5	Ubiquitin-like protein 5
Q92963	0.676	0.0132	RIT1	GTP-binding protein Rit1
P62491	0.686	0.00536	RB11A	Ras-related protein Rab-11A
Q8IY33	0.686	0.0182	MILK2	MICAL-like protein 2
Q8NBI6	0.686	0.039	XXLT1	Xyloside xylosyltransferase 1

O15431	0.692	0.00455	COPT1	High-affinity copper uptake protein 1
Q9BWN1	0.695	0.0147	PRR14	Proline-rich protein 14
O60921	0.703	0.0116	HUS1	Checkpoint protein HUS1
O60336	0.711	0.0457	MABP1	Mitogen-activated protein kinase-binding protein 1
Q07820	0.717	0.0342	MCL1	Induced myeloid leukemia cell differentiation protein Mcl-1
P35914	0.719	0.0199	HMGCL	Hydroxymethylglutaryl-CoA lyase, mitochondrial
Q14681	0.722	0.00193	KCTD2	BTB/POZ domain-containing protein KCTD2
G5EA36	0.726	0.000194	G5EA36	Cell division cycle 27, isoform CRA_c
O15440	0.731	0.000268	MRP5	ATP-binding cassette sub-family C member 5
P49711	0.735	0.03	CTCF	Transcriptional repressor CTCF
Q07866.3	0.744	0.0181	KLC1	Kinesin light chain 1
Q9Y605	0.745	0.00264	MOFA1	MORF4 family-associated protein 1
P54252	0.756	0.000556	ATX3	Ataxin-3
Q96S79	0.756	0.00533	RSLAB	Ras-like protein family member 10B
P18827	0.76	1.08E-06	SDC1	Syndecan-1
Q9Y6Q9	0.774	0.0494	NCOA3	Nuclear receptor coactivator 3
P20700.1	0.776	0.0151	LMNB1	Lamin-B1
P84103.1	0.785	0.0333	SRSF3	Serine/arginine-rich splicing factor 3
O95983.1	0.798	0.00614	MBD3	Methyl-CpG-binding domain protein 3
Q9BWM7.1	0.798	0.00611	SFXN3	Sideroflexin-3
Q15208.1	0.799	0.000265	STK38	Serine/threonine-protein kinase 38
Q8NHM5	0.81	0.00341	KDM2B	Lysine-specific demethylase 2B
P78337	0.811	0.0478	PITX1	Pituitary homeobox 1
Q6ZU65	0.812	0.0142	UBN2	Ubinuclein-2
O75157	0.815	0.002	T22D2	TSC22 domain family protein 2
P04350.5	0.816	0.0104	TBB4A	Tubulin beta-4A chain
Q6NSJ0	0.819	0.0408	MYORG	Myogenesis-regulating glycosidase

Q9NV64	0.819	0.000921	TM39A	Transmembrane protein 39A
O94855.1	0.82	0.00361	SC24D	Protein transport protein Sec24D
Q9H7Z6	0.82	0.00013	KAT8	Histone acetyltransferase KAT8
P48059	0.826	6.79E-06	LIMS1	LIM and senescent cell antigen-like-containing domain protein 1
P00338.2	0.828	0.0153	LDHA	L-lactate dehydrogenase A chain
Q16877	0.829	0.0411	F264	6-phosphofructo-2-kinase/fructose-2,6-bisphosphatase 4
P78332.1	0.835	6.34E-06	RBM6	RNA-binding protein 6
Q13443	0.835	0.0113	ADAM9	Disintegrin and metalloproteinase domain-containing protein 9
Q9UPW6	0.844	0.0253	SATB2	DNA-binding protein SATB2
Q96BF6.1	0.849	0.0369	NACC2	Nucleus accumbens-associated protein 2
Q8N4X5	0.853	0.0146	AF1L2	Actin filament-associated protein 1-like 2
Q8N8R3	0.858	0.0267	S2529	Mitochondrial basic amino acids transporter
Q96QE3	0.864	0.00775	ATAD5	ATPase family AAA domain-containing protein 5
O95478	0.869	2.22E-05	NSA2	Ribosome biogenesis protein NSA2 homolog
Q9NYB0	0.873	0.0386	TE2IP	Telomeric repeat-binding factor 2-interacting protein 1
P46734.1	0.874	0.00593	MP2K3	Dual specificity mitogen-activated protein kinase kinase 3
Q96C01	0.883	0.00597	F136A	Protein FAM136A
P57721.3	0.898	0.000129	PCBP3	Poly(rC)-binding protein 3
P07814.1	0.899	0.000868	SYEP	Bifunctional glutamate/proline--tRNA ligase
Q9Y324	0.902	0.00915	FCF1	rRNA-processing protein FCF1 homolog
Q9BXV9	0.907	0.00362	GON7	EKC/KEOPS complex subunit GON7
Q9BV68	0.908	0.000519	RN126	E3 ubiquitin-protein ligase RNF126
Q9C0B9	0.908	8.57E-06	ZCHC2	Zinc finger CCHC domain-containing protein 2

Q12986	0.91	0.000466	NFX1	Transcriptional repressor NF-X1
Q5VWQ8.2	0.933	8.46E-05	DAB2P	Disabled homolog 2-interacting protein
E7EN19	0.94	0.00199	E7EN19	Mitogen-activated protein kinase kinase kinase kinase 4
Q9H2V7	0.944	0.0495	SPNS1	Protein spinster homolog 1
P63241.1	0.951	0.000121	IF5A1	Eukaryotic translation initiation factor 5A-1
Q7Z5L9.2	0.951	0.0442		Interferon regulatory factor 2-binding protein 2
P25398	0.952	0.00129	RS12	40S ribosomal protein S12
O76080	0.953	0.0221	ZFAN5	AN1-type zinc finger protein 5
P62854.1	0.961	0.00102		40S ribosomal protein S26
Q96PN7	0.963	0.0205	TREF1	Transcriptional-regulating factor 1
A0A087WXI3	0.964	0.0238	A0A087WXI3	Synaptic functional regulator FMR1
Q9BWE0	0.968	0.0111	REPI1	Replication initiator 1
P07437.1	0.969	0.0106	TBB5	Tubulin beta chain
P17252.1	0.97	0.000106	KPCA	Protein kinase C alpha type
Q3SY17	0.981	0.0287	S2552	Mitochondrial nicotinamide adenine dinucleotide transporter SLC25A52
Q96C34	0.988	0.00992	RUND1	RUN domain-containing protein 1
P52943	1	0.0223	CRIP2	Cysteine-rich protein 2
Q8N3I7	1	0.0311	BBS5	Bardet-Biedl syndrome 5 protein
P02533.2	1.01	0.0159	K1C14	Keratin, type I cytoskeletal 14
Q8WXW3	1.01	0.000681	PIBF1	Progesterone-induced-blocking factor 1
P49641.1	1.02	1.16E-08	MA2A2	Alpha-mannosidase 2x
Q969E8	1.02	0.0345	TSR2	Pre-rRNA-processing protein TSR2 homolog
Q9NZC7	1.02	3.24E-06	WWOX	WW domain-containing oxidoreductase
P46531.1	1.03	0.00461	NOTC1	Neurogenic locus notch homolog protein 1
Q8NHQ8	1.03	9.69E-05	RASF8	Ras association domain-containing protein 8

O60343.1	1.05	0.0147	TBCD4	TBC1 domain family member 4
P60880	1.08	0.000656	SNP25	Synaptosomal-associated protein 25
Q96CP6	1.08	0.00872	ASTRA	Protein Aster-A
P53384	1.1	0.0398	NUBP1	Cytosolic Fe-S cluster assembly factor NUBP1
Q15390	1.11	0.00236	MTFR1	Mitochondrial fission regulator 1
Q8TCA0	1.11	0.000794	LRC20	Leucine-rich repeat-containing protein 20
Q9UJC5	1.11	8.98E-05	SH3L2	SH3 domain-binding glutamic acid-rich-like protein 2
F8VRH0	1.12	0.00364	F8VRH0	Poly(rC)-binding protein 2
P06454	1.12	0.0036	PTMA	Prothymosin alpha
P17029	1.12	0.00454	ZKSC1	Zinc finger protein with KRAB and SCAN domains 1
Q9NZJ6	1.13	7.85E-05	COQ3	Ubiquinone biosynthesis O-methyltransferase, mitochondrial
Q9BX95	1.16	0.000489	SGPP1	Sphingosine-1-phosphate phosphatase 1
Q56P03	1.17	0.00851	EAPP	E2F-associated phosphoprotein
O60814.2	1.18	0.00239	H2B1K	Histone H2B type 1-K
Q5VTB9	1.18	0.0108	RN220	E3 ubiquitin–protein ligase RNF220
Q86VQ6	1.18	0.00944	TRXR3	Thioredoxin reductase 3
Q9NY65	1.18	0.0368	TBA8	Tubulin alpha-8 chain
O75635	1.19	0.0105	SPB7	Serpin B7
Q7Z7H5.1	1.2	0.0057	TMED4	Transmembrane emp24 domain-containing protein 4
P63261	1.22	0.00176	ACTG	Actin, cytoplasmic 2
O95835.1	1.23	0.0113	LATS1	Serine/threonine-protein kinase LATS1
Q6P1R4	1.23	2.18E-06	DUS1L	tRNA-dihydrouridine(16/17) synthase
Q9P0P8	1.23	0.0138	MRES1	Mitochondrial transcription rescue factor 1
Q96PU4.1	1.24	0.0031	UHRF2	E3 ubiquitin–protein ligase UHRF2
Q969R2	1.25	0.0222	OSBP2	Oxysterol-binding protein 2

Q7Z7N9	1.26	0.00642	T179B	Transmembrane protein 179B
Q8N441	1.26	0.00166	FGRL1	Fibroblast growth factor receptor-like 1
P23528	1.28	0.000574	COF1	Cofilin-1
Q96HP4	1.28	6.83E-07	OXND1	Oxidoreductase NAD-binding domain-containing protein 1
Q8NEZ4	1.29	0.0071	KMT2C	Histone-lysine N-methyltransferase 2C
Q9H8U3	1.29	0.00131	ZFAN3	AN1-type zinc finger protein 3
Q9HB21	1.31	0.00152	PKHA1	Pleckstrin homology domain-containing family A member 1
Q96PQ0	1.33	0.0144	SORC2	VPS10 domain-containing receptor SorCS2
O00622	1.35	0.00026	CCN1	CCN family member 1
F5H4F6	1.36	0.000287	F5H4F6	Serine/threonine-protein kinase MARK2
O95684	1.37	0.0272	CEP43	Centrosomal protein 43
Q9BYG3	1.37	0.000343	MK67I	MKI67 FHA domain-interacting nucleolar phosphoprotein
P78540	1.4	1.45E-05	ARGI2	Arginase-2, mitochondrial
P10589	1.41	0.00171	K2C5	COUP transcription factor 1
Q16881.1	1.41	1.81E-05	TRXR1	Thioredoxin reductase 1, cytoplasmic
Q9Y2Z9	1.41	0.0041	COQ6	Ubiquinone biosynthesis monooxygenase COQ6, mitochondrial
Q8IWR0.1	1.42	2.72E-12	Z3H7A	Zinc finger CCCH domain-containing protein 7A
Q9H9A7	1.45	0.023	RMI1	RecQ-mediated genome instability protein 1
Q9H9L4	1.45	1.03E-06	KANL2	KAT8 regulatory NSL complex subunit 2
O60229	1.46	0.000134	KALRN	Kalirin
Q92800	1.46	0.037	EZH1	Histone-lysine N-methyltransferase EZH1
Q13283.1	1.48	0.00148	G3BP1	Ras GTPase-activating protein-binding protein 1
P02549	1.49	0.00191	SPTA1	Spectrin alpha chain, erythrocytic 1
Q7Z6B7	1.49	0.0264	SRGP1	SLIT-ROBO Rho GTPase-activating protein 1

Q96AX9	1.53	0.000328	MIB2	E3 ubiquitin-protein ligase MIB2
Q6N063	1.55	4.88E-06	OGFD2	2-oxoglutarate and iron-dependent oxygenase domain-containing protein 2
Q8IXQ5	1.55	3.38E-06	KLHL7	Kelch-like protein 7
Q96AT1	1.57	0.00132	K1143	Uncharacterized protein KIAA1143
Q7Z5L9.1	1.58	0.00044	I2BP2	Interferon regulatory factor 2-binding protein 2
Q9Y5V0	1.58	0.00125	ZN706	Zinc finger protein 706
O00408	1.6	0.00226	PDE2A	cGMP-dependent 3',5'-cyclic phosphodiesterase
P13667.1	1.62	0.000211	PDIA4	Protein disulfide-isomerase A4
P08047	1.63	0.000378	SP1	Transcription factor Sp1
Q53HC0	1.63	0.00379	CCD92	Coiled-coil domain-containing protein 92
A8MT69	1.67	0.0273	CENPX	Centromere protein X
O75084	1.69	9.74E-06	FZD7	Frizzled-7
Q07866.4	1.71	7.60E-06		Kinesin light chain 1
P62633	1.73	0.00708	CNBP	CCHC-type zinc finger nucleic acid-binding protein
Q9NSA3	1.73	7.49E-05	CNBP1	Beta-catenin-interacting protein 1
Q6PJP8	1.76	0.00147	DCR1A	DNA cross-link repair 1A protein
Q9BS18	1.76	0.000313	APC13	Anaphase-promoting complex subunit 13
Q9NWD8	1.77	2.66E-07	TM248	Transmembrane protein 248
Q9UNW1	1.78	0.00245	MINP1	Multiple inositol polyphosphate phosphatase 1
Q9H204	1.83	8.85E-08	MED28	Mediator of RNA polymerase II transcription subunit 28
P55211	1.85	0.000573	CASP9	Caspase-9
Q8IW41	1.85	0.00427	MAPK5	MAP kinase-activated protein kinase 5
Q8IX03	1.86	0.0192	KIBRA	Protein KIBRA
M0QYM7	1.93	0.00301	M0QYM7	Tubulin beta-4A chain
Q9UKN7	1.96	0.00131	MYO15	Unconventional myosin-XV
O43663	1.99	0.00529	PRC1	Protein regulator of cytokinesis 1

A0A590UJ08	2	4.25E-13	DLG1	Disks large homolog 1
O75683	2.04	0.00769	SURF6	Surfeit locus protein 6
Q96E29	2.21	8.99E-05	MTERF3	Transcription termination factor 3, mitochondrial
Q9H223.3	2.22	0.000118	EHD4	EH domain-containing protein 4
O60890	2.24	0.00484	OPHN1	Oligophrenin-1
P14854	2.48	0.000149	COX6B1	Cytochrome c oxidase subunit 6B1
P68371	2.81	0.038	TUBB4B	Tubulin beta-4B chain
P02795.1	2.83	0.00028	MT2A	Metallothionein-2
Q8NG06	3.15	0.00214	TRIM58	E3 ubiquitin–protein ligase TRIM58
P06241.2	6.34	6.31E-07	FYN	Tyrosine-protein kinase Fyn

Table S2. List of the 203 proteins modified in the proteome of chondrosarcoma cells MC615 irradiated with protons 12.6 at the dose of 0.1 Gy.

Entry	Fold	P value	Entry Name	Protein name
A2ARV4	-3.56	0.011391	LRP2	Low-density lipoprotein receptor-related protein 2
Q03391	-3.42	0.045702	NMDE4	Glutamate receptor ionotropic
Q8BVF4	-2.76	0.035322	CCD30	Coiled-coil domain-containing protein 30
Q9JHQ0	-2.5	0.016278	ANXA9	Annexin A9
P15655	-2.45	1.24E-05	FGF2	Fibroblast growth factor 2
P28666	-2.44	0.013169	MUG2	Murinoglobulin-2
P54265	-2.39	0.020099	DMPK	Myotonin-protein kinase
Q6URW6	-2.35	0.02474	MYH14	Myosin-14
G3X9Y5	-2.28	0.017936	G3X9Y5	Ubiquitin conjugation factor
Q03517	-2.2	0.007401	SCG2	Secretogranin-2
Q8BP74	-2.08	0.003788	PSTK	L-seryl-tRNA(Sec) kinase
Q9D1D1	-2.06	0.028269	TSN11	Tetraspanin-11

P00158	-2.01	0.012484	CYB	Cytochrome b
Q99LS1	-1.93	0.007064	MMAD	Cobalamin trafficking protein CblD
S4R2A9	-1.9	0.00357	S4R2A9	Protein transport protein Sec31A
P12032	-1.8	0.002469	TIMP1	Metalloproteinase inhibitor 1
E9Q6D3	-1.75	0.005328	E9Q6D3	Coiled-coil domain containing 121
Q8R5C8	-1.69	0.029608	ZMY11	Zinc finger MYND domain-containing protein 11
Q8BG26	-1.68	0.010148	RUSC1	AP-4 complex accessory subunit RUSC1
A0A0A6YVR7	-1.66	0.007593	A0A0A6YVR7	non-specific serine/threonine protein kinase
Q8CIA9	-1.65	0.013803	MF14B	Hippocampus abundant transcript-like protein 1
Q3UBX0	-1.6	0.040248	TM109	Voltage-gated monoatomic cation channel TMEM109
Q6XLQ8	-1.57	0.014003	Q6XLQ8	Calumenin
A2AJI1	-1.55	0.0074	A2AJI1	MAP7 domain containing 1
P01029	-1.53	0.010624	CO4B	Complement C4-B
Q8R4D1	-1.53	0.030322	SL9A8	Sodium/hydrogen exchanger 8
P05627	-1.49	0.028161	JUN	Transcription factor Jun
Q9CX84	-1.49	0.030963	RGS19	Regulator of G-protein signaling 19
Q0VG62	-1.48	0.004297	RBIS	Ribosomal biogenesis factor
P33435	-1.43	0.035761	MMP13	Collagenase 3
Q9CXV1	-1.43	0.042759	DHSD	Succinate dehydrogenase
A2A483	-1.41	0.03628	A2A483	Zinc finger, MYND-type containing 8
P61219	-1.32	0.03147	RPAB2	DNA-directed RNA polymerases I, II, and III subunit RPABC2
Q9DBX2	-1.32	0.03865	PHLP	Phosducin-like protein
Q99JH8	-1.29	0.023982	ERD21	ER lumen protein-retaining receptor 1
A0A087WQJ5	-1.27	0.043001	A0A087WQJ5	RIKEN cDNA 1810009A15 gene

Q8C0X8	-1.24	0.024424	SMKX	Predicted gene 14147
Q91ZI0	-1.23	0.049508	CELR3	Cadherin EGF LAG seven-pass G-type receptor 3
Q9CX13	-1.22	0.004919	CNIH4	Protein cornichon homolog 4
Q8VEA8	-1.21	0.038547	RAB7B	Ras-related protein Rab-7b
Q6NV72	-1.18	0.039407	WDCP	WD repeat and coiled-coil-containing protein
Q2VPQ9	-1.17	0.000235	EAF6	Chromatin modification-related protein MEAF6
Q8VEW1	-1.16	0.028081	Q8VEW1	Olfactory receptor
Q9JJA9	-1.16	0.030158	GRASP	General receptor for phosphoinositides 1-associated scaffold protein
O55101	-1.13	0.015431	SNG2	Synaptogyrin-2
A0A5F8MQ70	-1.11	0.032771	A0A5F8MQ70	Collagen, type V, alpha 1
P27782	-1.11	0.048214	LEF1	Transcription factor 7
Q505B7	-1.1	0.02197	ARCH	Protein archease
O88512	-1.06	0.029921	AP1G2	AP-1 complex subunit gamma-like 2
Q9JK42	-1.06	0.047348	PDK2	[Pyruvate dehydrogenase
Q9Z2P8	-1.06	0.03459	VAMP5	Vesicle-associated membrane protein 5
A0A1L1SS34	-1.05	0.01906	A0A1L1SS34	Anillin, actin binding protein
Q6P8H8	-1.05	0.001441	ALG8	Dolichyl pyrophosphate Glc1Man9GlcNAc2 alpha-1,3-glucosyltransferase
F8VQJ3	-1.04	0.000377	F8VQJ3	Laminin, gamma 1
Q9R088	-1.04	0.047222	KITM	Thymidine kinase 2, mitochondrial
Q505D7	-1.02	0.048977	OPA3	Optic atrophy 3 protein homolog
Q8CF66	-1.02	0.030554	LTOR4	Ragulator complex protein LAMTOR4
Q9JJZ6	-1.02	0.027123	KLF13	Krueppel-like factor 13
Q62179	-1.01	0.027428	SEM4B	Semaphorin-4B
Q9Z150	-0.997	0.030376	Q9Z150	Zinc finger and BTB domain containing 12

A0A5F8MPF2	-0.987	0.023186	A0A5F8MP F2	Tensin 3
Q3TEW6	-0.979	0.045814	MPZL1	Myelin protein zero-like protein 1
Q80X66	-0.977	0.019372	BTBDA	BTB/POZ domain-containing protein 10
Q8R1I1	-0.976	0.001857	QCR9	Cytochrome b-c1 complex subunit
Q9DB94	-0.951	0.000925	WDR53	WD repeat-containing protein 53
Q9EP78	-0.95	0.045949	CHST7	Carbohydrate sulfotransferase 7
P59598	-0.947	0.023165	ASXL1	Polycomb group protein
Q9CWU4	-0.933	0.002702	CA052	UPF0690 protein C1orf52 homolog
Q8CA71	-0.932	0.045212	SHSA4	Protein shisa-4
Q78IK2	-0.93	0.022776	ATPMK	ATP synthase membrane subunit K, mitochondrial
Q9CQ49	-0.918	0.031259	NCBP2	Nuclear cap-binding protein subunit 2
Q8R033	-0.907	0.025014	LYRM2	LYR motif-containing protein 2
Q8BVH9	-0.901	0.003599	METL6	tRNA N(3)-methylcytidine methyltransferase METTL6
P43407	-0.9	0.041053	SDC2	Syndecan-2
P63034	-0.9	0.016824	CYH2	Cytohesin-2
P70353	-0.9	0.034284	NFYC	Nuclear transcription factor Y subunit gamma
Q8BXN9	-0.9	0.005827	TM87A	Transmembrane protein 87A
P14719	-0.899	0.00842	ILRL1	Interleukin-1 receptor-like 1
Q8VD00	-0.891	0.003769	SGMR2	Sigma intracellular receptor 2
Q9JHS3	-0.886	0.011694	LTOR2	Ragulator complex protein LAMTOR2
Q8VID5	-0.882	0.034913	RECQ5	ATP-dependent DNA helicase Q5
Q3UX10	-0.881	0.03154	TBAL3	Tubulin alpha chain-like 3
P58334	-0.875	0.039604	KLF16	Krueppel-like factor 16
Q8JZU6	-0.873	0.036506	PXDC1	PX domain-containing protein 1
D3Z4J3	-0.871	0.007111	D3Z4J3	Myosin VA
Q8C145	-0.87	0.047446	S39A6	Zinc transporter ZIP6

Q1EG27	-0.859	0.022894	MYO3B	Myosin-IIIb
Q8CHK3	-0.853	0.030193	MBOA7	Lysophospholipid acyltransferase 7
Q9WVA2	-0.849	0.027384	TIM8A	Mitochondrial import inner membrane translocase subunit Tim8 A
P39876	-0.838	0.041717	TIMP3	Metalloproteinase inhibitor 3
Q8K201	-0.837	0.016905	KCT2	Keratinocyte-associated transmembrane protein 2
Q8K0S5	-0.832	0.008109	R4RL1	Reticulon-4 receptor-like 1
Q3UAW9	-0.823	0.004149	BRF2	Transcription factor IIIB 50 kDa subunit
S4R2J9	-0.823	0.025462	S4R2J9	Proline-rich coiled-coil 2C
A0A2R8VHL8	-0.813	0.001544	A0A2R8VHL8	Poly(rC) binding protein 2
Q60961	-0.812	0.01784	LAP4A	Lysosomal-associated transmembrane protein 4A
O35448	-0.803	0.039409	PPT2	Lysosomal thioesterase PPT2
Q9DB28	-0.799	0.008259	POP5	Ribonuclease P/MRP protein subunit POP5
P70196	-0.796	0.033606	TRAF6	TNF receptor-associated factor 6
O08665	-0.787	0.011408	SEM3A	Semaphorin-3A
O35682	-0.78	0.012639	MYADM	Myeloid-associated differentiation marker
Q6PFQ7	-0.776	0.017773	RASL2	Ras GTPase-activating protein 4
E9Q414	-0.767	0.030957	APOB	Apolipoprotein B-100
Q9DC29	-0.764	0.003212	ABCB6	ATP-binding cassette sub-family B member 6
Q99J09	-0.759	0.010269	MEP50	Methylosome protein WDR77
Q9CQT9	-0.75	0.014371	RCAF1	GEL complex subunit OPTI
Q9CQG6	-0.749	0.008823	TM147	BOS complex subunit TMEM147
Q9WTI7	-0.745	0.03829	MYO1C	Unconventional myosin-Ic
Q91VC9	-0.743	0.010581	GHITM	Growth hormone-inducible transmembrane protein
B2RS91	-0.741	0.001855	RRN3	RNA polymerase I-specific transcription initiation factor RRN3
O09111	-0.736	0.037764	NDUBB	NADH dehydrogenase

Q9CQI9	-0.735	0.001758	MED30	Mediator of RNA polymerase II transcription subunit 30
Q69ZB0	-0.733	0.030204	LRCC1	Leucine-rich repeat and coiled-coil domain-containing protein 1
A0A1Y7VP50	-0.725	0.04398	A0A1Y7VP50	Tectonin beta-propeller repeat containing 2
P43275	-0.716	0.04506	H11	Histone H1.1
Q9CQG1	-0.716	0.019783	CHAC2	Putative glutathione-specific gamma-glutamylcyclotransferase 2
Q6ZWQ7	-0.711	0.010074	SPCS3	Signal peptidase complex subunit 3
Q9JKN1	-0.71	0.018686	ZNT7	Zinc transporter 7
P58064	-0.709	0.038909	RT06	Small ribosomal subunit protein bS6m
Q78WZ7	-0.709	0.001479	RPA43	DNA-directed RNA polymerase I subunit RPA43
P25322	-0.707	0.023721	CCND1	G1/S-specific cyclin-D1
Q8BML1	-0.702	0.002256	MICA2	[F-actin]-monooxygenase MICAL2
Q791T5	-0.702	0.021709	MTCH1	Mitochondrial carrier homolog 1
Q9CWT3	-0.701	0.011354	SNX10	Sorting nexin-10
Q9JI46	-0.692	0.038733	NUDT3	Diphosphoinositol polyphosphate phosphohydrolase 1
P35441	-0.689	0.017967	TSP1	Thrombospondin-1
P62874	-0.689	0.01431	GBB1	Guanine nucleotide-binding protein G(I)/G(S)/G(T) subunit beta-1
Q9JIM1	-0.678	0.009644	S29A1	Equilibrative nucleoside transporter 1
P02088	-0.677	0.025713	HBB1	Hemoglobin subunit beta-1
Q78XF5	-0.671	0.015457	OSTC	Oligosaccharyltransferase complex subunit OSTC
Q8VDG5	-0.668	0.04961	PPCS	Phosphopantothenate--cysteine ligase
A0A0J9YTR2	-0.663	0.017385	A0A0J9YTR2	Ncbp2 antisense 2
P30282	-0.66	0.01648	CCND3	G1/S-specific cyclin-D3

Q91X52	-0.656	0.021487	DCXR	L-xylulose reductase (XR)
Q8R107	-0.656	0.018855	PRLD1	PRELI domain-containing protein 1, mitochondrial
Q6ZQ89	-0.652	0.008669	MARH6	E3 ubiquitin–protein ligase MARCHF6
Q80UX8	-0.647	0.040481	ABHDD	Protein ABHD13
Q8BR63	-0.646	0.044843	F177A	Protein FAM177A1
Q8BGH2	-0.645	0.021226	SAM50	Sorting and assembly machinery component 50 homolog
P97470	-0.643	0.016475	PP4C	Serine/threonine-protein phosphatase 4 catalytic subunit
Q3TV70	-0.643	0.02969	NR2CA	Nuclear receptor 2C2-associated protein
Q5SYD0	-0.635	0.036873	MYO1D	Unconventional myosin-Id
Q8BS95	-0.629	0.019664	GPHR	Golgi pH regulator
Q9JM51	-0.627	0.0156	PTGES	Prostaglandin E synthase
Q9EP82	-0.626	0.021101	WDR4	tRNA (guanine-N(7)-)-methyltransferase non-catalytic subunit WDR4
Q923B0	-0.621	0.008614	GGACT	Gamma-glutamylaminecyclotransferase
Q3UDW8	-0.617	0.032211	HGNAT	Heparan-alpha-glucosaminide N-acetyltransferase
Q9CQY5	-0.612	0.00902	MAGT1	Magnesium transporter protein 1
A0A0U1RP81	-0.61	0.038609	A0A0U1RP81	MICOS complex subunit MIC60
E9PY43	-0.61	0.013102	E9PY43	Glutamate rich 1
Q6NZP1	-0.609	0.012009	ZRAB3	DNA annealing helicase and endonuclease ZRANB3
G3X8Y3	-0.609	0.03032	G3X8Y3	N(alpha)-acetyltransferase 15, NatA auxiliary subunit
A2AH75	-0.608	0.00616	A2AH75	Kinesin family member 1B
A0A338P7C9	-0.605	0.008805	A0A338P7C9	Transmembrane protein 181A
Q99LH2	-0.603	0.0036	PTSS1	Phosphatidylserine synthase 1

P62305	-0.601	0.026668	RUXE	Small nuclear ribonucleoprotein E
Q8BLR9	-0.601	0.026671	HIF1N	Hypoxia-inducible factor 1-alpha inhibitor
A2ATU0	0.617	0.013764	DHTK1	2-oxoadipate dehydrogenase complex component E1
A2ARP1	0.632	0.029832	VIP1	Inositol hexakisphosphate and diphosphoinositol-pentakisphosphate kinase 1
Q8CH36	0.641	0.023632	S36A4	Neutral amino acid uniporter 4
Q9CWF6	0.642	0.036024	BBS2	Bardet-Biedl syndrome 2 protein homolog
Q8BFR6	0.643	0.022862	ZFAN1	AN1-type zinc finger protein 1
Q8CIE2	0.657	0.014231	ZMIZ2	Zinc finger MIZ domain-containing protein 2
P59941	0.662	0.03686	SIR6	NAD-dependent protein deacylase sirtuin-6
Q8BK30	0.677	0.039781	NDUV3	NADH dehydrogenase [ubiquinone] flavoprotein 3, mitochondrial
E9Q4M2	0.68	0.016576	E9Q4M2	Hormone-sensitive lipase
Q6P5E8	0.681	0.010351	DGKQ	Diacylglycerol kinase theta
Q9Z172	0.685	0.01888	SUMO3	Small ubiquitin-related modifier 3
Q3UR97	0.692	0.013653	SNX21	Sorting nexin-21
Q9CWB7	0.714	0.014054	YD286	Glutaredoxin-like protein C5orf63 homolog
Q9WUA6	0.741	0.000702	AKT3	RAC-gamma serine/threonine-protein kinase
Q80W14	0.744	0.046909	PR40B	Pre-mRNA-processing factor 40 homolog B
Q80V91	0.753	0.005468	DTX3	Probable E3 ubiquitin–protein ligase DTX3
Q3V3R4	0.777	0.012805	ITA1	Integrin alpha-1
Q3U422	0.782	0.020165	Q3U422	NADH:ubiquinone oxidoreductase core subunit V3
F8VPX2	0.797	0.045314	F8VPX2	F-box and WD-40 domain protein 9
Q9R1A8	0.836	0.011687	COP1	E3 ubiquitin–protein ligase COP1
F8VPQ2	0.845	0.015537	ARI4A	AT-rich interactive domain-containing protein 4A
Q7TMM8	0.886	0.004552	PAR16	Protein mono-ADP-ribosyltransferase PARP16
P17183	0.905	0.007455	ENOG	Gamma-enolase

Q9Z2M6	0.905	0.015033	UBL3	Ubiquitin-like protein 3
P18828	0.974	0.016318	SDC1	Syndecan-1
Q80YY7	0.987	0.011932	ZN618	Zinc finger protein 618
Q0VBK2	1.1	0.044065	K2C80	Keratin, type II cytoskeletal 80
Q8R5M0	1.1	0.024553	GIPC3	PDZ domain-containing protein GIPC3
Q4VBD9	1.12	0.000488	GZF1	GDNF-inducible zinc finger protein 1
Q9CPX9	1.13	0.004986	APC11	Anaphase-promoting complex subunit 11
M0QWZ1	1.15	0.033849	M0QWZ1	Family with sequence homology 193, member A
Q69Z89	1.16	0.017778	RADIL	Ras-associating and dilute domain-containing protein
Q9D0M5	1.16	0.043121	DYL2	Dynein light chain 2, cytoplasmic
Q8K4T5	1.2	0.014456	DUS19	Dual specificity protein phosphatase 19
A0A0A6YY72	1.22	0.012592	A0A0A6YY72	Inosine monophosphate dehydrogenase 2
Q5Y5T5	1.35	0.018868	ZDHC8	Palmitoyltransferase ZDHHC8
Q62252	1.36	0.006312	SP17	Sperm surface protein Sp17
Q99LX5	1.36	0.005421	MMTA2	Multiple myeloma tumor-associated protein 2 homolog
A0A140LI20	1.39	0.018403	A0A140LI20	Nucleosome assembly protein 1-like 4
Q3UWM4	1.47	0.021997	KDM7A	Lysine-specific demethylase 7A
Q8BYZ1	1.49	0.042381	ABI3	ABI gene family member 3
Q91XC8	1.54	0.026171	DAP1	Death-associated protein 1
Q9DAD6	1.68	0.048172	PROF3	Profilin-3
Q6KAU7	2.07	0.03917	PKHG2	Pleckstrin homology domain-containing family G member 2
E9Q557	2.37	0.008303	DESP	Desmoplakin
P97772	2.52	0.001764	GRM1	Metabotropic glutamate receptor 1

Table S3. . List of the 217 modified proteins in the SW1353 chondrosarcoma cell proteome irradiated with protons 12.6 at the dose of 0.5 Gy.

Entry	Fold	P value	Entry Name	Protein name
Q9BPW4	-3.75	0.00543	APOL4	Apolipoprotein L4
Q15147	-2.51	0.0129	PLCB4	1-phosphatidylinositol 4,5-bisphosphate phosphodiesterase beta-4
P51790	-2.38	5.75E-13	CLCN3	H(+)/Cl(-) exchange transporter 3
Q96R72	-2.37	0.0273	OR4K3	Olfactory receptor 4K3
Q9BZ81	-2.34	0.00103	MAGB5	Melanoma-associated antigen B5
Q6IA69	-2.33	0.00308	NADE	Glutamine-dependent NAD(+) synthetase
P02533.2	-2.25	5.90E-08	K1C14	Keratin, type I cytoskeletal 14
O95716	-2.24	1.35E-05	RAB3D	Ras-related protein Rab-3D
Q8N8Y2	-2.14	0.0182	VA0D2	V-type proton ATPase subunit d 2
P34932.1	-2.12	0.00272	HSP74	Heat shock 70 kDa protein 4
P01034	-2.11	0.00296	CYTC	Cystatin-C
P04259	-2.02	5.75E-13	KRT6B	Keratin, type II cytoskeletal 6B
Q14114	-1.89	5.79E-05	LRP8	Low-density lipoprotein receptor-related protein 8
O14593	-1.84	5.12E-06	RFXK	DNA-binding protein RFXANK
P02538	-1.84	3.57E-08	K2C6B	Keratin, type II cytoskeletal 6A
P13647	-1.84	3.15E-07	K2C5	Keratin, type II cytoskeletal 5
P04908.3	-1.83	4.25E-05		Histone H2A type 1-B/E
P18085.2	-1.78	3.29E-07	ARF4	ADP-ribosylation factor 4
Q5T440	-1.69	0.00194	CAF17	Putative transferase CAF17, mitochondrial
Q92766	-1.67	0.00718	RREB1	Ras-responsive element-binding protein 1
Q5M7Z0	-1.64	3.45E-07	RNFT1	E3 ubiquitin-protein ligase RNFT1
E5RHB1	-1.6	6.20E-05	E5RHB1	EF-hand calcium-binding domain-containing protein 3
Q9Y2G2	-1.6	0.00413	CARD8	Caspase recruitment domain-containing protein 8

O00213	-1.58	0.000508	APBB1	Amyloid beta precursor protein binding family B member 1
Q5D0E6	-1.58	0.00986	DALD3	DALR anticodon-binding domain-containing protein 3
Q9BZD2	-1.57	5.51E-06	S29A3	Equilibrative nucleoside transporter 3
P06702	-1.55	6.85E-07	S10A9	Protein S100-A9
Q9H7E2	-1.55	0.006	TDRD3	Tudor domain-containing protein 3
P02533.5	-1.54	0.00205	K2C6A	Keratin, type I cytoskeletal 14
P08779	-1.54	0.0479	K1C16	Keratin, type I cytoskeletal 16
Q9Y365	-1.53	0.0472	STA10	START domain-containing protein 10
O00559	-1.46	1.35E-06	RCAS1	Receptor-binding cancer antigen expressed on SiSo cells
P02538.4	-1.46	0.00818	H2AC18 _BACAM	Keratin, type II cytoskeletal 6A
Q53FZ2	-1.46	0.0264	ACSM3	Acyl-coenzyme A synthetase ACSM3, mitochondrial
Q93050.1	-1.46	0.0116	VPP1	V-type proton ATPase 116 kDa subunit a 1
Q96DD7	-1.46	2.68E-03	SHSA4	Protein shisa-4
Q07617	-1.43	0.000194	SPAG1	Sperm-associated antigen 1
Q96CC6	-1.41	0.0488	RHDF1	Inactive rhomboid protein 1
Q9Y394	-1.37	0.0333	DHRS7	Dehydrogenase/reductase SDR family member 7
Q6ZMR3	-1.36	0.0239	LDH6A	L-lactate dehydrogenase A-like 6A
P31321	-1.35	0.00141	KAP1	cAMP-dependent protein kinase type I-beta regulatory subunit
Q8NBN7	-1.33	1.28E-05	RDH13	Retinol dehydrogenase 13
Q8WVI0	-1.33	1.05E-05	UQCC5	Small integral membrane protein 4
Q7L5Y1	-1.32	0.000774	ENOF1	Mitochondrial enolase superfamily member 1
Q96ED9	-1.32	0.0178	HOOK2	Protein Hook homolog 2

O75771	-1.31	0.0466	RA51D	DNA repair protein RAD51 homolog 4
P02538.1	-1.28	0.0313	H2A1B	Keratin, type II cytoskeletal 6A
Q9UBH6	-1.25	4.53E-10	S53A1	Xenotropic and polytropic retrovirus receptor 1
P40425	-1.24	0.000281	PBX2	Pre-B-cell leukemia transcription factor 2
Q8NCM8	-1.24	5.75E-13	DYHC2	Cytoplasmic dynein 2 heavy chain 1
Q14627	-1.22	0.0342	I13R2	Interleukin-13 receptor subunit alpha-2
Q68DH5	-1.22	4.85E-02	LMBD2	G-protein coupled receptor-associated protein LMBRD2
Q9BSU1	-1.2	0.00279	PHAF1	Phagosome assembly factor 1
P81877	-1.18	0.000334	SSBP2	Single-stranded DNA-binding protein 2
Q6YBV0	-1.18	0.0237	S36A4	Proton-coupled amino acid transporter 4
P57060	-1.16	3.48E-05	RWD2B	RWD domain-containing protein 2B
Q8TAC2	-1.13	0.000532	JOS2	Josephin-2
Q9BT88	-1.12	8.11E-06	SYT11	Synaptotagmin-11
H7C3M7	-1.1	0.0222	H7C3M7	FERM, ARHGEF and pleckstrin domain-containing protein 2
Q86YD1	-1.07	0.017	PTOV1	Prostate tumor-overexpressed gene 1 protein
Q96G74	-1.07	0.00256	OTUD5	OTU domain-containing protein 5
Q96MG8	-1.07	0.00011	PCMD1	Protein-L-isoaspartate O-methyltransferase domain-containing protein 1
Q6PH81	-1.06	2.44E-05	CP087	UPF0547 protein C16orf87
A6NJ78	-1.05	0.00345	MET15	12S rRNA N4-methylcytidine (m4C) methyltransferase
O60218	-1.05	0.0434	AK1BA	Aldo-keto reductase family 1 member B10
P10276	-1.04	0.044	RARA	Retinoic acid receptor alpha
Q8TAV0	-1.03	0.0464	FA76A	Protein FAM76A
P0CG38.5	-1	0.000767	POTEI	POTE ankyrin domain family member I
O14807	-0.999	0.0097	RASM	Ras-related protein M-Ras

Q8N448	-0.986	0.00128	LNX2	Ligand of Numb protein X 2
A0A3B3ISE1	-0.977	0.0333	A0A3B3ISE1	Uncharacterized protein C9orf153
Q9NX95	-0.976	0.000163	SYBU	Syntabulin
Q16829	-0.963	0.0357	DUS7	Dual specificity protein phosphatase 7
P20929	-0.954	0.00427	NEBU	Nebulin
Q13129	-0.953	0.0268	RLF	Zinc finger protein Rlf
Q15052	-0.949	0.00125	ARHG6	Rho guanine nucleotide exchange factor 6
Q92546	-0.947	0.00133	RGP1	RAB6A-GEF complex partner protein 2
Q5BKY9	-0.921	0.0207	F133B	Protein FAM133B
P51784.1	-0.918	0.0104	UBP11	Ubiquitin carboxyl-terminal hydrolase 11
Q15119	-0.898	0.00035	PDK2	[Pyruvate dehydrogenase (acetyl-transferring)] kinase isozyme 2, mitochondrial
Q8IVG5	-0.897	0.00129	SAM9L	Sterile alpha motif domain-containing protein 9-like
Q7KZN9	-0.877	4.45E-05	COX15	Cytochrome c oxidase assembly protein COX15 homolog
Q9H008	-0.855	0.0304	LHPP	Phospholysine phosphohistidine inorganic pyrophosphate phosphatase
P08572	-0.833	0.0142	CO4A2	Collagen alpha-2(IV) chain
P34949	-0.823	0.00906	MPI	Mannose-6-phosphate isomerase
Q9NP92	-0.788	0.00213	RT30	39S ribosomal protein S30, mitochondrial
Q92597	-0.777	0.0349	NDRG1	Protein NDRG1
Q9NXG0	-0.777	0.0421	CNTLN	Centlein
P40424.1	-0.769	0.0375	PBX1	Pre-B-cell leukemia transcription factor 1
O00468	-0.764	0.000841	AGRIN	Agrin
Q9UPZ3	-0.76	0.00135	HPS5	BLOC-2 complex member HPS5
O43768	-0.758	0.00103	ENSA	Alpha-endosulfine
Q6P4F2	-0.752	0.0185	FDX2	Ferredoxin-2, mitochondrial
Q92828	-0.736	0.0283	COR2A	Coronin-2A

Q9NUA8	-0.725	0.0441	ZBT40	Zinc finger and BTB domain-containing protein 40
Q16760	-0.723	0.0296	DGKD	Diacylglycerol kinase delta
P51970	-0.711	0.00221	NDUA8	NADH dehydrogenase [ubiquinone] 1 alpha subcomplex subunit 8
Q06481	-0.705	6.97E-05	APLP2	Amyloid beta precursor like protein 2
Q8NBQ5	-0.705	0.00176	DHB11	Estradiol 17-beta-dehydrogenase 11
P00390	-0.699	4.19E-02	GSHR	Glutathione reductase, mitochondrial
Q8N138	-0.692	0.00539	ORML3	ORM1-like protein 3
Q9HD34	-0.691	0.000295	LYRM4	LYR motif-containing protein 4
Q5PRF9.1	-0.688	2.45E-03	SMAG2	Protein Smaug homolog 2
O00258	-0.681	0.0218	GET1	Guided entry of tail-anchored proteins factor 1
Q9C0C2	-0.669	0.0446	TB182	182 kDa tankyrase-1-binding protein
Q9Y2U5	-0.658	4.28E-05	M3K2	Mitogen-activated protein kinase kinase kinase 2
P63167.1	-0.654	1.31E-09	DYL1	Dynein light chain 1, cytoplasmic
P46531	-0.64	0.0178	NOTC1	Neurogenic locus notch homolog protein 1
Q96AV8	-0.626	0.0103	E2F7	Transcription factor E2F7
Q92889	-0.62	0.0397	XPF	DNA repair endonuclease XPF
Q5VWJ9	-0.61	6.98E-06	SNX30	Sorting nexin-30
Q68CZ2	-0.603	0.0237	TENS3	Tensin-3
P33897	-0.589	0.0137	ABCD1	ATP-binding cassette sub-family D member 1
Q14202	-0.587	0.0198	ZMYM3	Zinc finger MYM-type protein 3
P59190	0.587	0.0197	RAB15	Ras-related protein Rab-15
P28347.1	0.594	0.000505	TEAD1	Transcriptional enhancer factor TEF-1
O00762	0.595	0.0299	UBE2C	Ubiquitin-conjugating enzyme E2 C
Q6P158.2	0.602	0.0021	DHX57	Putative ATP-dependent RNA helicase DHX57
P60709.2	0.603	0.00842	ACTB	
P84090	0.608	0.00167	ERH	Enhancer of rudimentary homolog
P49366	0.609	1.54E-05	DHYS	Deoxyhypusine synthase

Q15554	0.613	1.44E-05	TERF2	Telomeric repeat-binding factor 2
A0A7P0T8A3	0.616	0.00283	A0A7P0T8A3	Transitional endoplasmic reticulum ATPase
O94985	0.621	0.0105	CSTN1	Calsyntenin-1
Q5JSL3	0.621	1.96E-02	DOC11	Dedicator of cytokinesis protein 11
Q8TE04	0.638	3.45E-05	PANK1	Pantothenate kinase 1
Q96CS2	0.64	0.00203	HAUS1	HAUS augmin-like complex subunit 1
O00458	0.642	0.0143	IFRD1	Interferon-related developmental regulator 1
P52747	0.643	0.00653	ZN143	Zinc finger protein 143
P62942	0.646	0.00508	FKB1A	Peptidyl-prolyl cis-trans isomerase FKBP1A
Q9NVF7	0.648	0.00576	FBX28	F-box-only protein 28
Q05DH4	0.65	0.0149	FHI1A	FHF complex subunit HOOK-interacting protein 1A
O14548	0.653	0.00197	COX7R	Cytochrome c oxidase subunit 7A-related protein, mitochondrial
Q92633	0.665	0.00971	LPAR1	Lysophosphatidic acid receptor 1
A6NHQ2	0.666	0.03	FBLL1	rRNA/tRNA 2'-O-methyltransferase fibrillarin-like protein 1
O75874.1	0.677	0.0394	IDHC	Isocitrate dehydrogenase [NADP] cytoplasmic
P13639.1	0.678	0.00862	EF2	Elongation factor 2
Q6PCB5	0.685	0.0415	RSBNL	Lysine-specific demethylase RSBN1L
Q99547	0.687	0.00232	MPH6	M-phase phosphoprotein 6
P51965	0.704	0.0189	UB2E1	Ubiquitin-conjugating enzyme E2 E1
Q9Y535	0.715	0.00446	RPC8	DNA-directed RNA polymerase III subunit RPC8
Q9Y6J0	0.739	0.0114	CABIN	Calcineurin-binding protein cabin-1
Q96CN4	0.745	0.0174	EVI5L	EVI5-like protein
Q96M34	0.751	0.000499	TEX55	Testis-specific expressed protein 55
P04908	0.752	6.20E-05		Histone H2A type 1-B/E

P30039	0.753	0.00193	PBLD	Phenazine biosynthesis-like domain-containing protein
Q9NVP2.1	0.762	0.0311	ASF1B	Histone chaperone ASF1B
P62079	0.764	0.0185	TSN5	Tetraspanin-5
O95427	0.767	4.08E-02	PIGN	GPI ethanolamine phosphate transferase 1
Q01469	0.776	0.00134	FABP5	Fatty acid-binding protein 5
P52848.1	0.787	0.000609	NDST1	Bifunctional heparan sulfate N-deacetylase/N-sulfotransferase 1
Q8N4J0	0.791	0.00268	CARME	Carnosine N-methyltransferase
Q96GS6	0.793	0.000774	AB17A	Alpha/beta hydrolase domain-containing protein 17A
Q9HCM2.1	0.795	0.00372	PLXA4	Plexin-A4
Q9UNH7.1	0.799	0.0211	SNX6	Sorting nexin-6
Q8TBK2	0.818	0.00243	SETD6	N-lysine methyltransferase SETD6
P20700.1	0.835	0.0402	LMNB1	Lamin-B1
Q969Q6	0.838	1.44E-05	P2R3C	Serine/threonine-protein phosphatase 2A regulatory subunit B'' subunit gamma
Q9BXV9	0.857	0.0377	GON7	EKC/KEOPS complex subunit GON7
P61165	0.859	0.00125	TM258	Transmembrane protein 258
Q5VT06	0.86	0.019	CE350	Centrosome-associated protein 350 (Cep350) (Centrosome-associated protein of 350 kDa)
P68431	0.872	0.0154	H31	Histone H3.1
Q8NHQ8	0.876	0.0114	RASF8	Ras association domain-containing protein 8
P22681.1	0.928	0.00608	CBL	E3 ubiquitin–protein ligase CBL
P07992	0.934	0.00547	ERCC1	DNA excision repair protein ERCC-1
P52735	0.947	0.0441	VAV2	Guanine nucleotide exchange factor VAV2
Q96B02	0.949	0.0156	UBE2W	Ubiquitin-conjugating enzyme E2 W
Q9UPW6	0.949	0.0447	SATB2	DNA-binding protein SATB2

Q9BWN1	0.953	0.00312	PRR14	Proline-rich protein 14
P06241	0.955	0.00173	FYN	Tyrosine-protein kinase Fyn
Q6DKI1	0.98	4.03E-10	RL7L	60S ribosomal protein L7-like 1
Q9UJC5	0.983	0.00704	SH3L2	SH3 domain-binding glutamic acid-rich-like protein 2
Q96QE3	1.02	0.00929	ATAD5	ATPase family AAA domain-containing protein 5
B5MCW3	1.03	0.0294	B5MCW3	Bromodomain-containing protein 8
Q9NS84	1.03	3.40E-10	CHST7	Carbohydrate sulfotransferase 7
P58004	1.05	0.0275	SESN2	Sestrin-2
Q9H5J4	1.06	0.00196	ELOV6	Elongation of very long chain fatty acids protein 6
E7EQL5	1.11	4.71E-05	E7EQL5	Cytoplasmic dynein 1 intermediate chain 2
Q9H7N4	1.11	0.00627	SFR19	Splicing factor, arginine/serine-rich 19
A6NE01	1.18	3.93E-05	F186A	Protein FAM186A
Q9NX18	1.25	0.000194	SDHF2	Succinate dehydrogenase assembly factor 2, mitochondrial
Q8TDY8	1.26	0.00424	IGDC4	Immunoglobulin superfamily DCC subclass member 4
Q9HCJ0	1.26	0.000324	TNR6C	Trinucleotide repeat-containing gene 6C protein
P57721.1	1.31	0.00032	PCBP3	Poly(rC)-binding protein 3
O75367	1.32	0.0419	H2AY	Core histone macro-H2A.1
P31942.1	1.34	0.0318	HNRH3	Heterogeneous nuclear ribonucleoprotein H3
Q6N075	1.35	0.0226	MFSD5	Molybdate-anion transporter
E9PK54	1.37	0.026	E9PK54	Heat shock cognate 71 kDa protein
Q8N441	1.38	0.00424	FGRL1	Fibroblast growth factor receptor-like 1
Q5TEC6	1.39	0.000691	H37	Histone H3-7
Q9H6Y2	1.39	0.0229	WDR55	WD repeat-containing protein 55
Q96CP6	1.42	0.00273	ASTRA	Protein Aster-A
Q9Y664	1.43	0.0166	KPTN	KICSTOR complex protein kaptin

O43542	1.45	2.47E-04	XRCC3	DNA repair protein XRCC3
Q8TF65	1.46	0.0227	GIPC2	PDZ domain-containing protein GIPC2
Q7Z5L2	1.48	0.000127	R3HCL	Coiled-coil domain-containing protein R3HCC1L
P01130	1.49	6.00E-05	LDLR	Low-density lipoprotein receptor
O60885.1	1.65	2.32E-09	BRD4	Bromodomain-containing protein 4
A0A1B0GUL3	1.69	0.00135	A0A1B0GUL3	Genetic suppressor element 1
P10589	1.69	0.00144	COT1	COUP transcription factor 1
Q5U5Q3	1.7	0.0359	MEX3C	RNA-binding E3 ubiquitin-protein ligase MEX3C
Q8NEZ4	1.73	0.00157	KMT2C	Histone-lysine N-methyltransferase 2C
O95793.1	1.74	0.0491	STAU1	Double-stranded RNA-binding protein Staufen homolog 1
A1L020.1	1.77	7.16E-11	MEX3A	RNA-binding protein MEX3A
P35908	1.94	0.0021	K22E	Keratin, type II cytoskeletal 2 epidermal
Q8IXQ5	1.96	2.34E-07	KLHL7	Kelch-like protein 7
Q9NWD8	2.06	1.75E-07	TM248	Transmembrane protein 248
Q02509	2.12	0.00211	OC90	Otoconin-90
Q8NB66	2.14	6.20E-05	UN13C	Protein unc-13 homolog C
Q562F6	2.15	0.0388	SGO2	Shugoshin 2
Q8TE73	2.25	0.00169	DYH5	Dynein axonemal heavy chain 5
P11586.1	2.41	0.00201	C1TC	C-1-tetrahydrofolate synthase, cytoplasmic
Q6VMQ6	2.71	0.00103	MCAF1	Activating transcription factor 7-interacting protein 1
Q8IX03	3.27	7.70E-05	KIBRA	Protein KIBRA
Q70UQ0	4.12	0.0488	IKIP	Inhibitor of nuclear factor kappa-B kinase-interacting protein
Q7RTS7	4.42	0.00292	K2C74	Keratin, type II cytoskeletal 74
P06241.2	6.2	5.31E-05	FYN	Tyrosine-protein kinase Fyn

Table S4. List of the 106 proteins modified in the proteome of chondrosarcoma cells MC615 irradiated with protons 12.6 at the dose of 0.5 Gy.

Entry	Fold	P value	Entry Name	Protein name
Q80U16	-4.19	0.047415819	RIPR2	Rho family-interacting cell polarization regulator 2
B7ZMV8	-4.03	0.048887842	B7ZMV8	Clathrin heavy-chain linker domain-containing protein 1
A2ARV4	-3.32	0.016312628	LRP2	Low-density lipoprotein receptor-related protein 2
Q9D1D1	-2.06	0.028112279	TSN11	Tetraspanin-11
Q8BP74	-2.01	0.004721822	PSTK	L-seryl-tRNA(Sec) kinase
Q8R5C8	-1.92	0.016771644	ZMY11	Zinc finger MYND domain-containing protein 11
Q99LS1	-1.73	0.012578318	MMAD	Cobalamin-trafficking protein CblD
S4R2A9	-1.7	0.006741453	S4R2A9	Protein transport protein Sec31A
Q8R4D1	-1.59	0.025764131	SL9A8	Sodium/hydrogen exchanger 8
Q8BG26	-1.58	0.013950007	RUSC1	AP-4 complex accessory subunit RUSC1
Q03517	-1.57	0.038066399	SCG2	Secretogranin-2 (Chromogranin-C)
Q8CIA9	-1.55	0.019034136	MF14B	Hippocampus abundant transcript-like protein 1
Q8K0S5	-1.42	0.000218947	R4RL1	Reticulon-4 receptor-like 1
Q3UHF7	-1.37	0.040315239	ZEP2	Transcription factor HIVEP2
Q5H8B9	-1.33	0.049888132	FREM3	FRAS1-related extracellular matrix protein 3
Q9JJZ6	-1.31	0.007531629	KLF13	Krueppel-like factor
A4Q9F0	-1.29	0.040469378	TTLL7	Tubulin polyglutamylase TTLL7
P12032	-1.23	0.020744731	TIMP1	Metalloproteinase inhibitor 1
Q8BVW0	-1.22	0.003166332	GANC	Neutral alpha-glucosidase C
A2AJI1	-1.21	0.026037534	A2AJI1	MAP7 domain containing 1

P20490	-1.2	0.028849158	FCERB	High-affinity immunoglobulin epsilon receptor subunit beta
Q2VPQ9	-1.15	0.000261614	EAF6	Chromatin modification-related protein MEAF6
A0A0R4J196	-1.14	0.043191848	A0A0R4J196	Sperm-associated antigen 9
Q9Z2P8	-1.14	0.024887064	VAMP5	Vesicle-associated membrane protein 5
Q91WD0	-1.13	0.025037722	GP108	Protein GPR108
P01029	-1.11	0.046492535	CO4B	Complement C4-B
P43407	-1.09	0.017303254	SDC2	Syndecan-2
A0A5F8MQ70	-1.08	0.0363835	A0A5F8MQ70	Collagen, type V, alpha 1
Q0VG62	-1.04	0.02715401	RBIS	Ribosomal biogenesis factor
Q9JJA9	-1.04	0.046873867	GRASP	General receptor for phosphoinositides 1-associated scaffold protein
Q5DU05	-1.03	0.025693059	CE164	Centrosomal protein of 164 kDa
D3Z4J3	-1.01	0.002857513	D3Z4J3	Myosin VA
Q9CX13	-0.926	0.021059561	CNIH4	Protein cornichon homolog 4
Q9CZT5	-0.923	0.03855847	VASN	Vasorin
E9Q5K9	-0.861	0.048931167	YTDC1	YTH domain-containing protein 1
Q8BWH0	-0.856	0.041551478	S38A7	Sodium-coupled neutral amino acid transporter 7
O54962	-0.849	0.040064356	BAF	Barrier-to-autointegration factor
Q64702	-0.842	0.017651356	PLK4	Serine/threonine–protein kinase PLK4
Q6P8H8	-0.832	0.006128648	ALG8	Dolichyl pyrophosphate Glc1Man9GlcNAc2 alpha-1,3-glucosyltransferase
Q8BGF9	-0.81	0.015647354	S2544	Solute carrier family 25 member 44
P0DOV1	-0.807	0.038363489	IFI5B	Interferon-activable protein 211
O89051	-0.806	0.041405977	ITM2B	Integral membrane protein 2B
P15066	-0.797	0.033483171	JUND	Transcription factor JunD
Q8BXN9	-0.751	0.015452241	TM87A	Transmembrane protein 87A

Q80WQ6	-0.75	0.045961498	RHDF2	Inactive rhomboid protein 2
Q9DB94	-0.745	0.004600572	WDR53	WD repeat-containing protein 53
Q8BWD8	-0.73	0.032425484	CDK19	Cyclin-dependent kinase 19
Q67BJ4	-0.713	0.048447315	A4GAT	Lactosylceramide 4-alpha-galactosyltransferase
P41731	-0.705	0.046841804	CD63	CD63 antigen
Q9JHS3	-0.705	0.034274805	LTOR2	Ragulator complex protein LAMTOR2
P32507	-0.704	0.021510535	NECT2	Nectin-2
Q9CWU4	-0.7	0.014088055	CA052	UPF0690 protein C1orf52 homolog
Q9CQT9	-0.699	0.020265999	RCAF1	GEL complex subunit OPTI
P30282	-0.694	0.012783256	CCND3	G1/S-specific cyclin-D3
Q3U0P5	-0.686	0.039873619	ENTP6	Ectonucleoside triphosphate diphosphohydrolase 6
Q6T264	-0.685	0.024939447	MAML1	Mastermind-like protein 1
Q61070	-0.683	0.044517749	EI24	Etoposide-induced protein 2.4
Q00493	-0.663	0.045160518	CBPE	Carboxypeptidase E
Q8BVH9	-0.659	0.02000812	METL6	tRNA N(3)-methylcytidine methyltransferase METTL6
Q8BG73	-0.657	0.035668206	SH3L2	SH3 domain-binding glutamic acid-rich-like protein 2
Q8BLR9	-0.653	0.018206698	HIF1N	Hypoxia-inducible factor 1-alpha inhibitor
Q9EP52	-0.649	0.025663924	TWSG1	Twisted gastrulation protein homolog 1
Q8R1I1	-0.646	0.019791624	QCR9	Cytochrome b-c1 complex subunit 9
A0A5H1ZRL1	-0.638	0.001436611	A0A5H1ZRL1	Spire-type actin nucleation factor 1
F8VQJ3	-0.636	9.57E-03	F8VQJ3	Laminin, gamma 1
Q8BJU2	-0.633	0.0396166	TSN9	Tetraspanin-9
Q5SYD0	-0.632	0.037560354	MYO1D	Unconventional myosin-Id
Q8VD00	-0.622	0.025651366	SGMR2	Sigma intracellular receptor 2

Q3TWL2	-0.617	0.0260615	PP4P1	Type 1 phosphatidylinositol 4,5-bisphosphate 4-phosphatase
Q78XF5	-0.614	0.023730048	OSTC	Oligosaccharyltransferase complex subunit OSTC
Q9D2C7	-0.612	0.02846499	BI1	Bax inhibitor 1
Q8K078	-0.606	0.007829876	SO4A1	Solute carrier organic anion transporter family member 4A1
A0A1L1SQ41	0.631	0.049726715	A0A1L1SQ41	Arginyl-tRNA--protein transferase 1
O70131	0.631	0.038888015	NINJ1	Ninjurin-1
Q9CYC6	0.655	0.025598556	DCP2	m7GpppN-mRNA hydrolase
Q78IS1	0.69	0.028604605	TMED3	Transmembrane emp24 domain-containing protein 3
Q9CQG3	0.69	0.036655307	ZMY19	Zinc finger MYND domain-containing protein 19
Q61160	0.694	0.012296191	FADD	FAS-associated death domain protein
Q9D1L0	0.748	0.013519324	CHCH2	Coiled-coil-helix-coiled-coil-helix domain-containing protein 2
A2ATU0	0.765	0.004121373	DHTK1	2-oxoadipate dehydrogenase complex component E1
Q7TMM8	0.801	0.008153882	PAR16	Protein mono-ADP-ribosyltransferase PARP16
Q6PAR0	0.815	0.012775883	KLD10	Kelch domain-containing protein 10
P59048	0.839	0.026035069	PDRG1	p53 and DNA damage-regulated protein 1
Q99JW4	0.869	0.03270801	LIMS1	LIM and senescent cell antigen-like-containing domain protein 1
Q80XH1	0.874	0.021369679	KXDL1	KxDL motif-containing protein 1
P59941	0.904	0.008183459	SIR6	NAD-dependent protein deacylase sirtuin-6
P62322	0.905	0.020384444	LSM5	U6 snRNA-associated Sm-like protein LSm5
Q62273	0.912	0.04829678	S26A2	Sulfate transporter
Q3TTL0	0.914	0.014403978	CC038	Uncharacterized protein C3orf38 homolog
Q8BKU8	0.915	0.045450222	TM87B	Transmembrane protein 87B

E0CXT7	0.923	0.013778254	E0CXT7	Cleavage and polyadenylation specificity factor subunit 4
Q3TC33	0.928	0.046155786	CC127	Coiled-coil domain-containing protein 127
Q69Z98	0.968	0.038441792	BRSK2	Serine/threonine-protein kinase BRSK2
O88573	0.97	0.025948704	AFF1	AF4/FMR2 family member 1
Q5U4E2	0.971	0.013110959	REPI1	Replication initiator 1
Q9WTZ1	1.04	0.000810889	RBX2	RING-box protein 2
Q9WV84	1.04	0.045702108	NDKM	Nucleoside diphosphate kinase, mitochondrial
Q6EJB6	1.08	0.03807507	UT14B	U3 small nucleolar RNA-associated protein 14 homolog B
Q9EPK2	1.1	0.018910564	XRP2	Protein XRP2
Q8K440	1.11	0.02727765	ABC8B	ABC-type organic anion transporter ABCA8B
Q9CQA0	1.15	0.009673432	CENPM	Centromere protein M
P97298	1.23	0.003632354	PEDF	Pigment epithelium-derived factor
Q80TD3	1.68	0.007488341	FNIP2	Folliculin-interacting protein 2
Q9JJN2	1.99	0.035793684	ZFHX4	Zinc finger homeobox protein 4
P97772	2.25	0.003715023	GRM1	Metabotropic glutamate receptor 1
E9Q557	2.4	0.007786366	DESP	Desmoplakin

Table S5. List of the 120 modified proteins in the SW1353 chondrosarcoma cell proteome irradiated with protons 12.6 at the dose of 2 Gy.

Intrare	Pliere	Valoare P	Entry Name	Denumire
Q9NS37	-4.45	0.0204	ZHANG	CREB/ATF bZIP transcription factor
P34931.1	-3.76	0.00112	HS71L	Heat shock 70 kDa protein 1-like
Q9C0K3	-3.08	0.00147	ARP3C	Actin-related protein 3C
Q9H799	-3.07	1.79E-12	CPLN1	Ciliogenesis and planar polarity effector 1
Q93050.1	-2.58	5.67E-06	VPP1	V-type proton ATPase 116 kDa subunit a 1

P34932.1	-2.26	0.00898	HSP74	Heat shock 70 kDa protein 4
Q95716	-2.19	0.000517	RAB3D	Ras-related protein Rab-3D
P12273	-2.16	0.00157	PIP	Prolactin-inducible protein
P31946.3	-1.99	0.00255	1433B	14-3-3 protein beta/alpha
Q13255	-1.95	0.0156	GRM1	Metabotropic glutamate receptor 1
Q5M7Z0	-1.73	5.13E-06	RNFT1	E3 ubiquitin–protein ligase RNFT1
Q6PJG6	-1.7	0.0322	BRAT1	BRCA1-associated ATM activator 1
Q96CQ1	-1.7	0.0369	S2536	Solute carrier family 25 member 36
Q96DD7	-1.67	0.00359	SHSA4	Protein shisa-4
O43731.1	-1.6	0.00159	ERD23	ER lumen protein-retaining receptor 3
Q9BRX9	-1.6	0.0166	WDR83	WD repeat domain-containing protein 83
P10276	-1.57	0.00319	RARA	Retinoic acid receptor alpha
Q0VGL1	-1.57	0.026	LTOR4	Ragulator complex protein LAMTOR4
Q9Y4C4	-1.57	0.0317	MFHA1	Malignant fibrous histiocytoma-amplified sequence 1
Q8IWA5	-1.55	0.000363	CTL2	Choline transporter-like protein 2
Q9H4B6	-1.55	0.00364	SAV1	Protein salvador homolog 1
P13647	-1.53	0.000995	K2C5	Keratin, type II cytoskeletal 5
P51790	-1.53	0.000155	CLCN3	H(+)/Cl(-) exchange transporter 3
Q99571	-1.51	0.00325	P2RX4	P2X purinoceptor 4
O75112	-1.49	0.0406	LDB3	LIM domain-binding protein 3
Q9Y592	-1.47	0.0359	CEP83	Centrosomal protein of 83 kDa
Q9UBU6	-1.43	0.00531	FA8A1	Protein FAM8A1
O00559	-1.38	0.000208	RCAS1	Receptor-binding cancer antigen expressed on SiSo cells
Q8TAC2	-1.36	0.000287	JOS2	Josephin-2
E5RHB1	-1.34	0.0117	E5RHB1	EF-hand calcium-binding domain-containing protein 3
Q2M3G4	-1.34	0.024	SHRM1	Protein Shroom1
P10275	-1.32	0.0161	ANDR	Androgen receptor
Q9NUA8	-1.32	0.000144	ZBT40	Zinc finger and BTB domain-containing protein 40

Q86SG6	-1.31	0.00376	NEK8	Serine/threonine-protein kinase Nek8
P51784.1	-1.27	0.00117	UBP11	Ubiquitin carboxyl-terminal hydrolase 11
Q7KZN9	-1.24	9.83E-08	COX15	Cytochrome c oxidase assembly protein COX15 homolog
Q8TCB7	-1.24	0.0394	METL6	tRNA N(3)-methylcytidine methyltransferase METTL6
Q9BSU1	-1.24	0.0128	PHAF1	Phagosome assembly factor 1
Q92546	-1.23	0.000194	RGP1	RAB6A-GEF complex partner protein 2
O60237	-1.18	0.00632	MYPT2	Protein phosphatase 1 regulatory subunit 12B
Q8NBN7	-1.14	0.00345	RDH13	Retinol dehydrogenase 13
Q8TBZ3	-1.14	0.00421	WDR20	WD repeat-containing protein 20
O14807	-1.13	0.0146	RASM	Ras-related protein M-Ras
P57060	-1.13	0.00115	RWD2B	RWD domain-containing protein 2B
Q5PRF9.1	-1.13	9.67E-07	SMAG2	Protein Smaug homolog 2
Q96Q07	-1.09	0.0387	BTBD9	BTB/POZ domain-containing protein 9
P54709	-1.08	0.0401	AT1B3	Sodium/potassium-transporting ATPase subunit beta-3
P31641.1	-1.07	0.0045	SC6A6	Sodium- and chloride-dependent taurine transporter
Q70EL1	-1.07	5.29E-07	UBP54	Inactive ubiquitin carboxyl-terminal hydrolase 54
Q9UBH6	-1.07	1.69E-05	S53A1	Xenotropic and polytropic retrovirus receptor 1
P07437.5	-1.05	0.0128	TBB5	Tubulin beta chain
O60268	-1.04	0.0368	K0513	Uncharacterized protein KIAA0513
Q8NCM8	-1.03	7.86E-07	DYHC2	Cytoplasmic dynein 2 heavy chain 1
Q15119	-1.01	0.000648	PDK2	Pyruvate dehydrogenase
Q9NPJ3	-0.997	0.00568	ACO13	Acyl-coenzyme A thioesterase 13
Q6PH81	-0.986	0.00164	CP087	UPF0547 protein C16orf87
Q8N4A0	-0.95	0.00647	GALT4	Polypeptide N-acetylgalactosaminyltransferase 4
Q58FF6	-0.943	0.0158	H90B4	Putative heat shock protein HSP 90-beta 4
Q9BT88	-0.926	0.00428	SYT11	Synaptotagmin-11
Q8NDA8	-0.9	0.000768	MROH1	Maestro heat-like repeat-containing protein family member 1

Q6Y1H2	-0.896	0.00429	HACD2	Very-long-chain (3R)-3-hydroxyacyl-CoA dehydratase 2
Q8IV08	-0.893	0.000883	PLD3	5'-3' exonuclease PLD3
Q9UNL2	-0.881	0.0044	SSRG	Translocon-associated protein subunit gamma
Q06481	-0.854	2.32E-05	APLP2	Amyloid beta precursor like protein 2
Q96H78	-0.851	0.0328	S2544	Solute carrier family 25 member 44
P63167.1	-0.828	3.57E-12	DYL1	Dynein light chain 1, cytoplasmic
Q6P1M0.1	-0.806	0.00334	S27A4	Long-chain fatty acid transport protein 4
Q9BZL4	-0.785	0.0498	PP12C	Protein phosphatase 1 regulatory subunit 12C
P11388.1	-0.784	0.0103	TOP2A	DNA topoisomerase 2-alpha
Q9HD34	-0.741	0.00115	LYRM4	LYR motif-containing protein 4
Q9P2X0	-0.732	0.018	DPM3	Dolichol-phosphate mannosyltransferase subunit 3
P52298	-0.709	0.0113	NCBP2	Nuclear cap-binding protein subunit 2
Q9BZV1	-0.707	0.00115	UBXN6	UBX domain-containing protein 6
Q9NRY6	-0.666	0.0148	PLS3	Phospholipid scramblase 3
Q96AV8	-0.639	0.0423	E2F7	Transcription factor E2F7
Q5JWR5.1	-0.638	0.0104	DOP1	Protein dopey-1
O00291.1	-0.636	0.000454	HIP1	Huntingtin-interacting protein 1
P46459.1	-0.626	0.000861	NSF	Vesicle-fusing ATPase
O15120	-0.625	0.000879	PLCB	1-acyl-sn-glycerol-3-phosphate acyltransferase beta
Q6NVY1	-0.624	0.0144	HIBCH	3-hydroxyisobutyryl-CoA hydrolase, mitochondrial
Q14332	-0.623	0.0103	FZD2	Frizzled-2
Q5VW38	-0.602	0.016	GP107	Protein GPR107
Q9H0X9	-0.601	0.00118	OSBL5	Oxysterol-binding protein-related protein 5
Q92543	-0.596	0.00162	SNX19	Sorting nexin-19
O00499.1	0.605	0.0059	BIN1	Myc box-dependent-interacting protein 1
P62072	0.649	0.0301	TIM10	Mitochondrial import inner membrane translocase subunit Tim10
Q9Y587	0.66	0.00328	AP4S1	AP-4 complex subunit sigma-1

Q5T5X7	0.68	0.0128	BEND3	BEN domain-containing protein 3
Q12933	0.684	0.0198	TRAF2	TNF receptor-associated factor 2
Q5VVJ2	0.686	0.00355	MYSM1	Deubiquitinase MYSM1
Q9C0G0	0.711	0.000399	ZN407	Zinc finger protein 407
Q15555.1	0.713	0.011	MARE2	Microtubule-associated protein RP/EB family member 2
Q9Y388	0.714	0.0498	RBMX2	RNA-binding motif protein, X-linked 2
F8W7C6	0.717	0.0136	F8W7C6	60S ribosomal protein L10
Q8NEJ9	0.752	0.000186	NGDN	Neuroguidin
Q9H5H4	0.753	7.60E-05	ZN768	Zinc finger protein 768
G5EA36	0.789	0.00615	G5EA36	Cell division cycle 27, isoform CRA_c
Q8TA86	0.821	0.0451	RP9	Retinitis pigmentosa 9 protein
Q92567	0.844	0.0488	F168A	Protein FAM168A
P54252	0.857	0.00849	ATX3	Ataxin-3
F8VXL3	0.867	0.0395	F8VXL3	Myosin light polypeptide 6
E7EQL5	0.872	0.0196	E7EQL5	Cytoplasmic dynein 1 intermediate chain 2
Q96S79	0.923	0.0238	RSLAB	Ras-like protein family member 10B
O60281	0.935	0.0442	ZN292	Zinc finger protein 292
P24386.1	0.984	8.62E-07	RAE1	Rab proteins geranylgeranyltransferase component A 1
Q9UIL1	0.988	0.00289	SCOC	Short coiled-coil protein
Q9H7Z6	1.02	0.000744	KAT8	Histone acetyltransferase KAT8
Q01469	1.05	7.47E-05	FABP5	Fatty acid-binding protein 5
Q5T011	1.08	0.0133	SZT2	KICSTOR complex protein SZT2
Q8WXW3	1.12	0.0131	PIBF1	Progesterone-induced-blocking factor 1
Q86VZ5	1.2	0.0203	SMS1	Phosphatidylcholine:ceramide cholinephosphotransferase 1
Q8N9T8	1.21	0.0358	KRI1	Protein KRI1 homolog
Q96EL2	1.21	1.60E-10	RT24	28S ribosomal protein S24, mitochondrial
O95983.1	1.22	0.00168	MBD3	Methyl-CpG-binding domain protein 3

Q8IXQ5	1.32	0.0138	KLHL7	Kelch-like protein 7
Q969R2	1.63	0.0486	OSBP2	Oxysterol-binding protein 2
Q96PN7	1.63	0.00247	TREF1	Transcriptional-regulating factor 1
O75478	1.67	0.0425	TAD2A	Transcriptional adapter 2-alpha
Q02548	2.08	0.05	PAX5	Paired box protein Pax-5
P46937.1	4.67	9.12E-07	YAP1	Transcriptional coactivator YAP1

Table S6. List of the 197 proteins modified in the proteome of chondrosarcoma cells MC615 irradiated with protons 12.6 at the dose of 2 Gy.

Entry	Fold	P value	Entry Name	Protein name
O08983	-3.35	0.015597017	HPS1	BLOC-3 complex member HPS1
Q6PE84	-2.72	0.008546653	STML3	Stomatin-like protein 3
Q9ESD7	-2.57	0.042175882	DYSF	Dysferlin
P28666	-2.28	0.018766912	MUG2	Murinoglobulin-2
Q6URW6	-2.27	0.029255695	MYH14	Myosin-14
G3X9Y5	-2.17	0.023030639	G3X9Y5	Ubiquitin conjugation factor E4
A0A286YDA5	-2.07	0.036584654	A0A286YDA5	G protein-coupled receptor kinase
P00158	-2.06	0.011052545	CYB	Cytochrome b
A2AQ07	-2.01	0.006653231	TBB1	Tubulin beta-1 chain
Q8BP74	-2.01	0.004678227	PSTK	L-seryl-tRNA(Sec) kinase
S4R2A9	-1.85	0.004190808	S4R2A9	Protein transport protein Sec31A
Q8R5C8	-1.79	0.023012446	ZMY11	Zinc finger MYND domain-containing protein 11
Q6NZD2	-1.75	0.007474113	Q6NZD2	Sorting nexin-1
A0A0N4SVT8	-1.72	0.034352216	A0A0N4SVT8	DnaJ heat shock protein family (Hsp40) member B8
A0A0A6YVR7	-1.64	0.008240302	A0A0A6YVR7	non-specific serine/threonine protein kinase
Q3UBX0	-1.55	0.044755748	TM109	Voltage-gated monoatomic cation channel TMEM109

Q9JK42	-1.52	0.008681047	PDK2	[Pyruvate dehydrogenase (acetyl-transferring)] kinase isozyme 2, mitochondrial
Q8BG26	-1.49	0.019047502	RUSC1	AP-4 complex accessory subunit RUSC1
Q5H8B9	-1.48	0.032525486	FREM3	FRAS1-related extracellular matrix protein 3
A0A2I3BQS8	-1.47	0.043233362	A0A2I3BQS8	Predicted gene, 55359
Q9CXV1	-1.44	0.041114508	DHSD	Succinate dehydrogenase [ubiquinone] cytochrome b small subunit, mitochondrial
Q9R088	-1.41	0.011853566	KITM	Thymidine kinase 2, mitochondrial
Q6XLQ8	-1.39	0.025366415	Q6XLQ8	Calumenin
Q99LS1	-1.39	0.035180425	MMAD	Cobalamin trafficking protein CblD
Q8CEG8	-1.37	0.049730419	UBP27	Ubiquitin carboxyl-terminal hydrolase 27
A1BN54	-1.31	0.004291537	A1BN54	Alpha-actinin-1
F6RUI8	-1.3	0.043106138	F6RUI8	Multiple ankyrin repeats single-KH domain-binding protein 3
Q9ERE3	-1.3	0.047108973	SGK3	Serine/threonine-protein kinase Sgk3
E9PV41	-1.27	0.048305554	E9PV41	Diaphanous-related formin 1
P12032	-1.22	0.021906455	TIMP1	Metalloproteinase inhibitor 1
Q9CYC3	-1.2	0.01518524	TM39A	Transmembrane protein 39A
O55101	-1.2	0.01125303	SNG2	Synaptogyrin-2
Q8VEA8	-1.19	0.041511786	RAB7B	Ras-related protein Rab-7b
Q8CA71	-1.17	0.016773036	SHSA4	Protein shisa-4
Q8VEW1	-1.17	0.027708339	Q8VEW1	Olfactory receptor
Q91X52	-1.15	0.000730206	DCXR	L-xylulose reductase
Q9CX13	-1.15	0.006937146	CNIH4	Protein cornichon homolog 4
Q9D2L1	-1.13	0.032650549	ARSK	Arylsulfatase K
P26883	-1.1	0.028754016	FKB1A	Peptidyl-prolyl cis-trans isomerase FKBP1A
Q78IK2	-1.09	0.010233998	ATPMK	ATP synthase membrane subunit K, mitochondrial
Q8K0S5	-1.08	0.001634668	R4RL1	Reticulon-4 receptor-like 1

A0A5F8MQ70	-1.08	0.036764598	A0A5F8MQ70	Collagen, type V, alpha 1
Q505B7	-1.06	0.025805549	ARCH	Protein archease
Q8R033	-1.06	0.011525883	LYRM2	LYR motif-containing protein 2
Q8R1I1	-1.05	0.001146731	QCR9	Cytochrome b-c1 complex subunit 9
F6TFN2	-1.05	0.011548048	F6TFN2	LIM domain only 7
P61804	-1.05	0.044663987	DAD1	Dolichyl-diphosphooligosaccharide--protein glycosyltransferase subunit DAD1
Q9Z2P8	-1.05	0.035630532	VAMP5	Vesicle-associated membrane protein 5
B2M1R6	-1.04	0.03317889	B2M1R6	Heterogeneous nuclear ribonucleoprotein K
Q2VPQ9	-1.01	0.000686766	EAF6	Chromatin modification-related protein MEAF6
O35309	-1.01	0.024352249	NMI	N-myc-interactor
Q8BVH9	-0.994	0.001904995	METL6	tRNA N(3)-methylcytidine methyltransferase METTL6
E9PX63	-0.992	4.48E-02	E9PX63	H-2 class I histocompatibility antigen, D-B alpha chain
Q8K211	-0.979	0.005955375	COPT1	High-affinity copper uptake protein 1
O35448	-0.977	0.016345352	PPT2	Lysosomal thioesterase PPT2
Q8BN82	-0.976	0.0197108	S17A5	Sialin (H(+)/nitrate cotransporter)
Q8CF66	-0.973	0.037872349	LTOR4	Ragulator complex protein LAMTOR4
Q1EG27	-0.962	0.013123181	MYO3B	Myosin-IIIb
Q9JMG7	-0.958	0.019358589	HDGR3	Hepatoma-derived growth factor-related protein 3
Q9CQX5	-0.957	0.019925853	CLDN1	Claudin domain-containing protein 1
Q8CHK3	-0.951	0.018277028	MBOA7	Lysophospholipid acyltransferase 7
Q923B0	-0.936	0.000613078	GGACT	Gamma-glutamylaminecyclotransferase
O35683	-0.932	0.009745883	NDUA1	NADH dehydrogenase [ubiquinone] 1 alpha subcomplex subunit 1
F8VQJ3	-0.919	9.50E-04	F8VQJ3	Laminin, gamma 1
Q91WM2	-0.915	0.031926608	HDHD5	Haloacid dehalogenase-like hydrolase domain-containing 5

Q8BXN9	-0.914	0.005300024	TM87A	Transmembrane protein 87A
P30282	-0.913	0.002591637	CCND3	G1/S-specific cyclin-D3
Q9D7I5	-0.913	0.00167978	LHPP	Phospholysine phosphohistidine inorganic pyrophosphate phosphatase
P0DOV1	-0.911	0.02256737	IFI5B	Interferon-activable protein 211
Q8JZU6	-0.894	0.033107058	PXDC1	PX domain-containing protein 1
Q9CQ49	-0.888	0.035932223	NCBP2	Nuclear cap-binding protein subunit 2
E9Q5K9	-0.885	0.043931772	YTDC1	YTH domain-containing protein 1
Q62179	-0.885	0.046598457	SEM4B	Semaphorin-4B
A0A5F8MPF2	-0.879	0.038367099	A0A5F8MPF2	Tensin 3
Q9CQT9	-0.876	0.006178148	RCAF1	GEL complex subunit OPTI
G3X8Y3	-0.875	0.004650437	G3X8Y3	N(alpha)-acetyltransferase 15, NatA auxiliary subunit
Q9JHG6	-0.871	0.012348734	RCAN1	Calcipressin-1
Q3UX10	-0.86	0.034948248	TBAL3	Tubulin alpha chain-like 3
Q9WVA2	-0.851	0.026986007	TIM8A	Mitochondrial import inner membrane translocase subunit Tim8 A
Q8VID5	-0.849	0.040836497	RECQ5	ATP-dependent DNA helicase Q5
Q9DCF9	-0.835	0.017813281	SSRG	Translocon-associated protein subunit gamma
Q6P8H8	-0.82	0.00662392	ALG8	Dolichyl pyrophosphate Glc1Man9GlcNAc2 alpha-1,3-glucosyltransferase
Q80X45	-0.815	0.037661308	KISHB	Protein kish-B
D3Z4J3	-0.787	0.012314479	D3Z4J3	Myosin VA
D3YVS1	-0.786	0.006972964	Q9D7A0	Smoothelin
Q8VI63	-0.784	0.044409216	MOB2	MOB kinase activator 2
Q91VC9	-0.784	0.007897935	GHITM	Growth hormone-inducible transmembrane protein
Q9JHS3	-0.771	0.023141327	LTOR2	Ragulator complex protein LAMTOR2
Q8BGF9	-0.766	0.020528638	S2544	Solute carrier family 25 member 44

Q8K201	-0.756	0.027190116	KCT2	Keratinocyte-associated transmembrane protein 2
Q9DB94	-0.756	0.004234911	WDR53	WD repeat-containing protein 53
Q61337	-0.755	0.018138098	BAD	Bcl2-associated agonist of cell death
Q64702	-0.754	0.029254815	PLK4	Serine/threonine-protein kinase PLK4
P11031	-0.745	0.02317385	TCP4	Activated RNA polymerase II transcriptional coactivator p15
Q5PT53	-0.745	0.022991756	NTCP7	Sodium/bile acid cotransporter 7
P62874	-0.743	0.009578422	GBB1	Guanine nucleotide-binding protein G(I)/G(S)/G(T) subunit beta-1
Q69ZB0	-0.743	0.028435346	LRCC1	Leucine-rich repeat and coiled-coil domain-containing protein 1
A0A338P7C9	-0.738	0.002693076	A0A338P7C9	Transmembrane protein 181A
A0A0U1RP81	-0.737	0.016572774	A0A0U1RP81	MICOS complex subunit MIC60
Q3TWL2	-0.737	0.010855784	PP4P1	Type 1 phosphatidylinositol 4,5-bisphosphate 4-phosphatase
Q8BG73	-0.731	0.022314941	SH3L2	SH3 domain-binding glutamic acid-rich-like protein 2
Q61070	-0.726	0.034934792	EI24	Etoposide-induced protein 2.4 (p53-induced gene 8 protein)
A2AH75	-0.722	0.002127353	A2AH75	Kinesin family member 1B
Q8BLR9	-0.72	0.011061024	HIF1N	Hypoxia-inducible factor 1-alpha inhibitor
P58064	-0.716	0.037353993	RT06	Small ribosomal subunit protein bS6m
Q9R123	-0.715	0.000842232	NAA80	N-alpha-acetyltransferase 80
Q99L20	-0.714	0.014081987	GSTT3	Glutathione S-transferase theta-3
Q9CQG1	-0.712	0.020407256	CHAC2	Putative glutathione-specific gamma-glutamylcyclotransferase 2
Q9WU81	-0.711	0.03089095	G6PT3	Glucose-6-phosphate exchanger SLC37A2
Q8BTV1	-0.707	0.000913336	TUSC3	Tumor suppressor candidate 3
P15655	-0.705	4.58E-02	FGF2	Fibroblast growth factor 2

Q6T264	-0.7	0.022471316	MAML1	Mastermind-like protein 1
Q8VD00	-0.696	0.015000464	SGMR2	Sigma intracellular receptor 2
Q6P3D0	-0.693	0.016958296	NUD16	U8 snoRNA-decapping enzyme
Q9JI46	-0.693	0.038348713	NUDT3	Diphosphoinositol polyphosphate phosphohydrolase 1
Q99LH2	-0.692	0.001460624	PTSS1	Phosphatidylserine synthase 1
Q80UX8	-0.69	0.030996834	ABHDD	Protein ABHD13
Q3UDW8	-0.684	0.020247912	HGNAT	Heparan-alpha-glucosaminide N-acetyltransferase
Q791T5	-0.681	0.024911353	MTCH1	Mitochondrial carrier homolog 1
Q3U0P5	-0.673	0.042994	ENTP6	Ectonucleoside triphosphate diphosphohydrolase 6
A0A2R8VHL8	-0.672	0.005177355	A0A2R8VHL8	Poly(rC) binding protein 2
Q9CWT3	-0.672	0.014166327	SNX10	Sorting nexin-10
P70210	-0.671	0.00096046	TEAD3	Transcriptional enhancer factor TEF-5
Q9CWU4	-0.671	0.017401273	CA052	UPF0690 protein C1orf52 homolog
Q8BGH2	-0.658	0.019182618	SAM50	Sorting and assembly machinery component 50 homolog
Q99J09	-0.658	0.021112846	MEP50	Methylosome protein WDR77
Q8BWD8	-0.656	0.049698122	CDK19	Cyclin-dependent kinase 19
P97762	-0.654	0.033316918	RP9	Retinitis pigmentosa 9 protein homolog
Q9CQI9	-0.653	0.003766936	MED30	Mediator of RNA polymerase II transcription subunit 30
Q78XF5	-0.652	0.017875382	OSTC	Oligosaccharyltransferase complex subunit OSTC
A2BG75	-0.639	0.045400388	A2BG75	Nuclear factor 1
Q3TJ91	-0.629	0.001003879	L2GL2	LLGL scribble cell polarity complex component 2
Q3TQB2	-0.624	0.017067516	FXRD1	FAD-dependent oxidoreductase domain-containing protein 1
O35640	-0.622	0.00431089	ANXA8	Annexin A8
P35293	-0.622	0.043495616	RAB18	Ras-related protein Rab-18

P97470	-0.618	0.019910521	PP4C	Serine/threonine-protein phosphatase 4 catalytic subunit
Q7TNC9	-0.614	0.035795661	I5P1	Inositol polyphosphate-5-phosphatase A
Q9CQY5	-0.613	0.008909123	MAGT1	Magnesium transporter protein 1
Q8C6M1	0.601	0.04099934	UBP20	Ubiquitin carboxyl-terminal hydrolase 20
Q3UMG6	0.605	0.035948381	NDK7	Nucleoside diphosphate kinase homolog 7
P17183	0.607	0.04918334	ENOG	Gamma-enolase
Q9JL61	0.622	0.000583745	RFX5	DNA-binding protein Rfx5
Q8BL95	0.632	0.039378675	CF298	Cilia- and flagella-associated protein 298
Q9D168	0.635	0.011629355	INT12	Integrator complex subunit 12
Q9R1A8	0.648	0.038300701	COP1	E3 ubiquitin–protein ligase COP1
Q68FE8	0.649	0.021207797	Z280D	Zinc finger protein 280D
Q3THF9	0.666	0.036827931	CQ10B	Coenzyme Q-binding protein COQ10 homolog B, mitochondrial
Q7TNG8	0.671	0.032206411	LDHD	Probable D-lactate dehydrogenase, mitochondrial
Q9JI70	0.676	0.047403467	MKKS	Molecular chaperone MKKS
P41971	0.679	0.04158703	ELK3	ETS domain-containing protein Elk-3
Q91XS1	0.679	0.039892341	MTMR4	Myotubularin-related protein 4
Q8R184	0.699	0.018037139	F110A	Protein FAM110A
Q91VN1	0.703	0.00201794	ZNF24	Zinc finger protein 24
O08747	0.72	0.004163869	UNC5C	Netrin receptor UNC5B
P15864	0.725	0.005130381	H12	Histone H1.2
Q71B07	0.725	0.023679163	D19L3	Protein C-mannosyl-transferase DPY19L3
Q8R344	0.727	0.003017667	CCD12	Coiled-coil domain-containing protein 12
A0A0G2JDR6	0.728	0.025021026	A0A0G2JDR6	Leucine-rich repeat flightless-interacting protein 2
Q5PR68	0.73	0.004422983	CE112	Centrosomal protein of 112 kDa
Q9CQG3	0.741	0.027054951	ZMY19	Zinc finger MYND domain-containing protein 19

Q9D1L0	0.753	0.01305682	CHCH2	Coiled-coil-helix–coiled-coil-helix domain-containing protein 2
Q8BFY7	0.754	0.028092555	PIMRE	Protein PIMREG
A0A494BBC4	0.769	0.047965752	A0A494BBC4	BCL2-associated agonist of cell death
Q8BUV6	0.779	0.014319852	LSM11	U7 snRNA-associated Sm-like protein LSm11
Q9D1F3	0.784	0.000447528	EOLA1	Protein EOLA1
Q60773	0.803	0.002221799	CDN2D	Cyclin-dependent kinase 4 inhibitor D
Q61411	0.836	0.006352654	RASH	GTPase HRas
Q8C753	0.846	0.042347994	KATIP	Katanin-interacting protein
Q8R3R8	0.847	0.000622887	GBRL1	Gamma-aminobutyric acid receptor-associated protein
Q80V91	0.85	0.002601332	DTX3	Probable E3 ubiquitin–protein ligase DTX3
B7ZNL9	0.881	0.001142288	B7ZNL9	TatD DNase domain containing 2
P59941	0.892	0.008812365	SIR6	NAD-dependent protein deacylase sirtuin-6
Q8CCI5	0.892	0.047236609	RYBP	RING1 and YY1-binding protein
P62322	0.895	0.021437603	LSM5	U6 snRNA-associated Sm-like protein LSm5
Q7TSG3	0.925	0.023462448	FBX5	F-box-only protein 5
A0A0A6YY72	0.93	0.043013176	A0A0A6YY72	Inosine monophosphate dehydrogenase 2
E9PXR3	0.944	0.003083366	E9PXR3	Sestrin 1
A0A338P695	0.964	0.048698521	A0A338P695	Sphingomyelin phosphodiesterase 4
P59913	1.06	0.03300825	PCMD1	Protein-L-isoaspartate O-methyltransferase domain-containing protein 1
Q9JJE4	1.08	0.034246928	PAQR4	Progestin and adipoQ receptor family member 4
Q8VEB3	1.12	0.012023266	MACIR	Macrophage immunometabolism regulator
Q6EJB6	1.13	0.03162425	UT14B	U3 small nucleolar RNA-associated protein 14 homolog B
Q7TMM8	1.15	0.000833221	PAR16	Protein mono-ADP-ribosyltransferase PARP16
Q8BU27	1.17	0.046271151	PPM1M	Protein phosphatase 1M

Q8BYN5	1.23	0.038848637	FSD1L	FSD1-like protein
Q6GQW0	1.38	0.012744441	ABTB3	Ankyrin repeat and BTB/POZ domain-containing protein 3
Q99KS2	1.38	0.013252847	NGRN	Neugrin
E0CZ03	1.44	0.001553578	E0CZ03	Zinc finger protein 263
Q8C827	1.47	0.037309834	ZFP62	Zinc finger protein 62
Q80TD3	1.5	0.013671345	FNIP2	Folliculin-interacting protein 2
Q60722	1.6	0.035552588	ITF2	Transcription factor 4
Q6R5P0	1.67	0.004363429	TLR11	Toll-like receptor 11
Q3U1C4	1.7	0.009846824	SEBP2	Selenocysteine insertion sequence-binding protein 2
Q8VIK5	1.74	0.037773781	PEAR1	Platelet endothelial aggregation receptor 1
Q9CPW0	1.81	0.035349918	CNTP2	Contactin-associated protein-like 2
Q5DTN8	3.19	0.049576065	JKIP3	Janus kinase and microtubule-interacting protein 3
Q921V7	4.77	0.040689821	CTL3	Choline transporter-like protein 3

Table S7. SW1353 Gene Ontology enrichment and associated genes across different radiation doses.

SW1353	GO ID	GO term	Associated Genes (%)	Nr. Genes	Gene associate
0 Gy vs 0.1 Gy	GO:0005200	structural constituent of cytoskeleton	9.82	11.00	ACTG1, KRT14, KRT5, LMNB1, SPTA1, TUBA4A, TUBA8, TUBAL3, TUBB, TUBB4A, TUBB4B
	GO:0010498	proteasomal protein catabolic process	4.28	23.00	AKIRIN2, AKT1, ANAPC13, APC, ATXN3, BCAP31, CDC27, CSNK1A1, DAB2IP, FAM8A1, FBXL3, FBXO6, FEM1B, KCTD2, MARCHF6, NHLRC3, OPHN1, PCBP2, PRKCA, RNF126, RNF34, RNFT1, WWP2
	GO:0035329	hippo signaling	13.33	6.00	LATS1, LIMD1, MAP2K3, NEK8, PJA2, WWC1
0 Gy vs 0.5 Gy	GO:0052650	NADP-retinol dehydrogenase activity	21.43	3.00	AKR1B10, DHRS7, RDH13
	GO:0005200	structural constituent of cytoskeleton	8.04	9.00	ACTB, AGRN, KRT14, KRT16, KRT2, KRT5, KRT6A, KRT6B, LMNB1
	GO:0030216	keratinocyte differentiation	4.55	8.00	KRT16, KRT2, KRT5, KRT6A, KRT6B, KRT74, MACROH2A1, NOTCH1

	GO:0031424	Keratinization	6.98	6.00	KRT16, KRT2, KRT5, KRT6A, KRT6B, KRT74
	GO:0036297	interstrand cross-link repair	10.87	5.00	ERCC1, ERCC4, RAD51D, VCP, XRCC3
	GO:0045104	intermediate filament cytoskeleton organization	7.45	7.00	KRT14, KRT16, KRT2, KRT5, KRT6A, KRT6B, KRT74
	GO:0045109	intermediate filament organization	9.72	7.00	KRT14, KRT16, KRT2, KRT5, KRT6A, KRT6B, KRT74
	GO:0031848	protection from non-homologous end joining at telomere	30.00	3.00	ERCC1, ERCC4, TERF2
	GO:1904354	negative regulation of telomere capping	33.33	3.00	ERCC1, ERCC4, TERF2
	GO:0006312	mitotic recombination	17.24	5.00	ERCC1, ERCC4, RAD51D, TERF2, XRCC3
	GO:0000722	telomere maintenance via recombination	29.41	5.00	ERCC1, ERCC4, RAD51D, TERF2, XRCC3
	GO:0006479	protein methylation	4.07	9.00	BRD4, FBLL1, GET1, KMT2C, MACROH2A1, PCMTD1, RAB3D, RLF, SETD6
	GO:0008213	protein alkylation	4.07	9.00	BRD4, FBLL1, GET1, KMT2C, MACROH2A1, PCMTD1, RAB3D, RLF, SETD6
	GO:0001325	formation of extrachromosomal circular DNA	20.00	3.00	ERCC1, TERF2, XRCC3
	GO:0090737	telomere maintenance via telomere trimming	20.00	3.00	ERCC1, TERF2, XRCC3
	GO:0090656	t-circle formation	20.00	3.00	ERCC1, TERF2, XRCC3
0 Gy vs 2 Gy	GO:0006893	Golgi to plasma membrane transport	4.62	3.00	NSF, OSBPL5, PHAF1
	GO:0019217	regulation of fatty acid metabolic process	4.12	4.00	APLP2, FABP5, PDK2, PIBF1
	GO:0035329	hippo signaling	6.67	3.00	NEK8, SAV1, YAP1
	GO:0044380	protein localization to cytoskeleton	4.84	3.00	CEP83, MAPRE2, PIBF1
	GO:0072698	protein localization to microtubule cytoskeleton	5.17	3.00	CEP83, MAPRE2, PIBF1

Table S8. MC615 gene ontology enrichment and associated genes across different radiation doses.

MC615	GO ID	GO term	Associated Genes (%)	Nr. Genes	Gene associate
0 Gy vs 0.1 Gy	GO:0000018	regulation of DNA recombination	4.48	6.00	H1f1, Meaf6, Ppp4c, Recql5, Sirt6, Zranb3
	GO:0018196	peptidyl-asparagine modification	16.67	3.00	Alg8, Hif1an, Magt1
	GO:0032784	regulation of DNA-templated transcription, elongation	5.68	5.00	Med30, Ncbp2, Recql5, Sirt6, Zmynd11
	GO:0034243	regulation of transcription elongation from RNA polymerase II promoter	6.67	5.00	Med30, Ncbp2, Recql5, Sirt6, Zmynd11
	GO:0099515	actin filament-based transport	13.64	3.00	Myo1c, Myo1d, Myo5a
	GO:0030050	vesicle transport along actin filament	15.00	3.00	Myo1c, Myo1d, Myo5a
0 Gy vs 0.5 Gy	GO:2001044	regulation of integrin-mediated signaling pathway	13.04	3.00	Cd63, Lamc1, Timp1
0 Gy vs 2 Gy	GO:0006487	protein N-linked glycosylation	7.04	5.00	Alg8, Dad1, Magt1, Ostc, Tusc3
	GO:0008630	intrinsic apoptotic signaling pathway in response to DNA damage	4.96	6.00	Bad, Cdkn2d, Ei24, Fnip2, Hnrnpk, Tmem109
	GO:0018196	peptidyl-asparagine modification	22.22	4.00	Alg8, Hif1an, Magt1, Tusc3
	GO:0018279	protein N-linked glycosylation via asparagine	17.65	3.00	Alg8, Magt1, Tusc3
	GO:0021756	striatum development	12.50	3.00	Cntnap2, Mkks, Secisbp2
	GO:0032784	regulation of DNA-templated transcription, elongation	5.68	5.00	Med30, Ncbp2, Recql5, Sirt6, Zmynd11
	GO:0006368	transcription elongation from RNA polymerase II promoter	5.21	5.00	Med30, Ncbp2, Recql5, Sirt6, Zmynd11
	GO:0034243	regulation of transcription elongation from RNA polymerase II promoter	6.67	5.00	Med30, Ncbp2, Recql5, Sirt6, Zmynd11

Table S9. SW1353 STRING network enrichment and associated proteins.

	Term ID	Term description	Strength	False discovery rate	Matching proteins in your network (labels)
SW1353 0.1 Gy	GO:0008152	Metabolic process	0.18	1.04E-06	COX15,OTUD5,CDIPT,RNASEH2A,RAB3D,RPS12,MED28,KALRN,SGPP1,RARA, COQ3,BRD8,APC,AKIRIN2,USP37,HUS1,BRIP1,FAM8A1,BUD13,LPIN2,ARG2,SPG11,MTFR1,KMT2C,GATB,TARBP2,NEK8,SDE2,LIMD1,NDUFS6,MARCHF6,CDK16,NDUFB9,ZC3H12C,LDHAL6A,NUBP1,NIFK,RNF126,MAP4K2,MYORG,TERF2IP,CA4,PPIC,ZCCHC4,RNFT1,GON7,RAMAC,FEM1B,TSPAN5, UTP23,XXYLT1,ERCC4,ISCU,ATAD5,RMI1,PIBF1,GEMIN4,ENO3,TMEM39A, CASP9,SPPL2C,SMAD3,ABCC5,COQ6,PDE2A,KLHL7,FCF1,PTMA,TRAF3IP2,RPL27A,NRDE2,FBXL3,RPS26,UBL5,PCBP2,MT-CO1,PJA2,MT-ND4,DCLRE1A,COQ8A, UST,SYT11,DPM3,PLEKHA1, FMR1,MINPP1,NCOA3,DOLK,RLF, SLC25A25,BAK1,HMGCL,TSR2,STK19,FBXO6,KDM2B,ATG2A,B3GALT6,NFX1,NHLRC3,RREB1,NADK2,NCBP3,RNF34,VTI1A,RPL14,FGFRL1,SPTLC3,METTL15,PRKAR1B,MARK2,CCN1,EZH1,KAT8,CNBP,KANSL2,DUSP7,REPIN1,ADAM9,ANAPC13,ZNF827,SMUG1,MRPS30,MIB2,CDC27,RPS6KA1,GALNT4,TRERF1,GRAMD1A,CITED2,PRPF40B,BRF1,MAPKAPK5,CENPX, COX6B1,UQCR11,SEC11C,TAF15,APBB1,NSA2,MTRES1,CTCF,ATXN3,DYNC2H1

	GO:0044237	Cellular metabolic process	0.2	3.52E-06	COX15,CDIPT,RNASEH2A,RAB3D,RPS12,MED28,KALRN,SGPP1,RARA,COQ3,APC,AKIRIN2,USP37,HUS1,BRIP1,FAM8A1,BUD13,LPIN2,ARG2,SPG11,MTFR1,KMT2C,GATB,TARBP2,NEK8,SDE2,LIMD1,NDUFS6,MARCHF6,CDK16,NDUFB9,ZC3H12C,LDHAL6A,NUBP1,NIFK,RNF126,MAP4K2,TERF2IP,CA4,ZCCHC4,GON7,RAMAC,FEM1B,UTP23,XXYLT1,ERCC4,ISCU,ATAD5,RMI1,PIBF1,GEMIN4,ENO3,TMEM39A,SMAD3,ABCC5,COQ6,PDE2A,FCF1,PTMA,RPL27A,NRDE2,FBXL3,RPS26,UBL5,PCBP2,MT-CO1,MT-ND4,DCLRE1A,COQ8A,UST, SYT11,DPM3,PLEKHA1,FMR1,MINPP1,DOLK,RLF,SLC25A25,BAK1,HMGCL,TSR2,STK19,FBXO6,ATG2A,B3GALT6,NFX1,NHLRC3,RREB1,NADK2,NCBP3,RNF34,VTI1A,RPL14,FGFRL1,SPTLC3,METTL15,PRKAR1B,MARK2,CCN1,EZH1,CNBP,DUSP7,REPIN1,ANAPC13,ZNF827,SMUG1,MRPS30,CDC27,RPS6KA1,GALNT4,GRAMD1A,CITED2,PRPF40B,BRF1,MAPKAPK5,CENPX,COX6B1,UQCR11,SEC11C,TAF15,NSA2,MTRES1,CTCF,ATXN3
	GO:0009987	Cellular process	0.08	0.00013	COX15,OTUD5,RIN3,CDIPT,MGA,RNASEH2A,RAB3D,RPS12,NPRL2,MED28,KALRN,SGPP1,KRT5,RARA,COQ3,BRD8,SNAP25,APC,AKIRIN2,USP37,HUS1,BRIP1,FAM8A1,BUD13,CLCN3,LPIN2,ARG2,SPG11,RAB11A,MTFR1,KMT2C,GATB,IFT46,TUBB4A,PITX1,HPS5,TARBP2,NEK8,SDE2,LIMD1,NDUFS6,MARCHF6,CDK16,NDUFB9,ZC3H12C,LDHAL6A,SCLT1,PLEKHH2,GRM1,NUBP1,NIFK,FZD7,MTERF3,RNF126,MAP4K2,BBS5,MICALL2,MYORG,POLR2M,TERF2IP,CA4,PPIC,ZCCHC4,GON7,SDC2,RAMAC,FEM1B,TSPAN5,UTP23,XXYLT1,SPNS1,ERCC4,ISCU,ARR3,ATAD5,RMI1,PIBF1,GEMIN4,ENO3,MAP11,NR2F1,COX14,TMEM39A,SP1,CASP9,OSBP2,SMAD3,ABCC5,COQ6,PDE2A,EBAG9,TUBB4B,ENSA,LAMTOR4,FCF1,PTMA,TRAF3IP2,RPL27A,NRDE2,FBXL3,RPS26,DYNLRB1,UBL5,SLC25A29,PCBP2,MT-CO1,PJA2,MT-ND4, DCLRE1A,COQ8A,FGFR1OP,UST,XPR1,SYT11,DPM3,PLEKHA1,MCL1,FMR1,MINPP1,NCOA3,SURF6,DOLK,RLF,SLC25A25,SLC3

					1A1,BAK1,HMGCL,PBX2,TSR2,STK19,FBXO6,KDM2B,CTNNBIP1,ATG2A,RGP1,B3GALT6,NFX1,NHLRC3,RREB1,SDC1,NADK2,NCBP3,RNF34,VTI1A,PRC1,RPL14,FGFRL1,SPTLC3,DTNB,METTL15,PRKAR1B,MARK2,MYO7A,CPLANE1,BCAP31,CCN1,SATB2,EZH1,KAT8,CNBP,KLC1,KANSL2,DUSP7,REPIN1,UBN2,ADAM9,ANAPC13,ZNF827,SORCS2,SMUG1,MRPS30,COX18,MIB2,WWC1,CCNC,SLC25A30,TXNRD3,SPA17,CFL1,CDC27,RPS6KA1,GALNT4,TRERF1,GRAMD1A,CITED2,MYL6,PRPF40B,BRF1,MAPKAPK5,CNN2,WWOX,ACTG1,CENPX,COX6B1,UQCR11,SEC11C,TAF15,UQCC2,APBB1,NSA2,MTRES1,BTBD8,RTTN,CTCF,MYO15A,ATXN3,GET1,DYNC2H1,SLC25A52
	GO:0006807	Nitrogen compound metabolic process	0.18	0.00018	COX15,OTUD5,RNASEH2A,RAB3D,RPS12,MED28,KALRN,SGPP1,RARA,BRD8,APC,AKIRIN2,USP37,HUS1,BRIP1,FAM8A1,BUD13,ARG2,KMT2C,GATB,TARBP2,NEK8,SDE2,NDUFS6,MARCHF6,CDK16,NDUFB9,ZC3H12C,NIFK,RNF126,MAP4K2,TERF2IP,PPIC,ZCCHC4,RNFT1,GON7,RAMAC,FEM1B,TSPAN5,UTP23,XXYLT1,ERCC4,ATAD5,RMI1,PIBF1,GEMIN4,ENO3,CASP9,SPPL2C,SMAD3,ABCC5,PDE2A,KLHL7,FCF1,PTMA,TRAF3IP2,RPL27A,NRDE2,FBXL3,RPS26,UBL5,PCBP2,PJA2,MT-ND4,DCLRE1A,UST,DPM3,FMR1,NCOA3,DOLK,RLF,SLC25A25,BAK1,HMGCL,TSR2,STK19,FBXO6,KDM2B,B3GALT6,NFX1,NHLRC3,RREB1,NADK2,NCBP3,RNF34,RPL14,FGFRL1,SPTLC3,METTL15,PRKAR1B,MARK2,EZH1,KAT8,CNBP,KANSL2,DUSP7,REPIN1,ADAM9,ANAPC13,ZNF827,SMUG1,MRPS30,MIB2,CDC27,RPS6KA1,GALNT4,TRERF1,PRPF40B,BRF1,MAPKAPK5,CENPX,SEC11C,TAF15,APBB1,NSA2,MTRES1,CTCF,ATXN3,DYNC2H1
	GO:0043170	Macromolecule metabolic process	0.19	0.00025	OTUD5,RNASEH2A,RAB3D,RPS12,MED28,KALRN,RARA,BRD8,APC,AKIRIN2,USP37,HUS1,BRIP1,FAM8A1,BUD13,KMT2C,GATB,TARBP2,NEK8,SDE2,MARCHF6,CDK16,ZC3H12C,NIFK,RNF126,MAP4K2,TERF2IP,PPIC,ZCCHC4,RNFT1,GON7,RAMAC,FEM1B,TSPAN5,UTP23,XXYLT1,ERCC4,ATAD5,RMI1,PIBF1,GEMIN4,CAS

					P9,SPPL2C,SMAD3,ABCC5,KLHL7,FCF1,PTMA,TRAF3IP2,RPL27A,NRDE2,FBXL3,RPS26,UBL5,PCBP2,PJA2,DCLRE1A,UST,DPM3,FMR1,NCOA3,DOLK,RLF,BAK1,TSR2,STK19,FBXO6,KDM2B,B3GALT6,NFX1,NHLRC3,RREB1,NCBP3,RNF34,RPL14,FGFRL1,METTL15,PRKAR1B,MARK2,EZH1,KAT8,CNBP,KANSL2,DUSP7,REPIN1,ADAM9,ANAPC13,ZNF827,SMUG1,MRPS30,MIB2,CDC27,RPS6KA1,GALNT4,TRERF1,PRPF40B,BRF1,MAPKAPK5,CENPX,SEC11C,TAF15,APBB1,NSA,MTRES1,CTCF,ATXN3,DYNC2H1
	GO:0071704	Organic substance metabolic process	0.16	0.0005	COX15,OTUD5,CDIPT,RNASEH2A,RAB3D,RPS12,MED28,KALRN,SGPP1,RARA,COQ3,BRD8,APC,AKIRIN2,USP37,HUS1,BRIP1,FAM8A1,BUD13,LPIN2,ARG2,KMT2C,GATB,TARBP2,NEK8,SDE2,NDUFS6,MARCHF6,CDK16,NDUFB9,ZC3H12C,LDHAL6A,NIFK,RNF126,MAP4K2,MYORG,TERF2IP,PPIC,ZCCHC4,RNFT1,GON7,RAMAC,FEM1B,TSPAN5,UTP23,XXYLT1,ERCC4,ATAD5,RMI1,PIBF1,GEMIN4,ENO3,CASP9,SPPL2C,SMAD3,ABCC5,COQ6,PDE2A,KLHL7,FCF1,PTMA,TRAF3IP2,RPL27A,NRDE2,FBXL3,RPS26,UBL5,PCBP2,PJA2,MT-ND4,DCLRE1A, COQ8A,UST,DPM3,PLEKHA1,FMR1,MINPP1,NCOA3,DOLK,RLF,SLC25A25,BAK1,HMGCL,TSR2,STK19,FBXO6,KDM2B,B3GALT6,NFX1,NHLRC3,RREB1,NADK2,NCBP3,RNF34,RPL14,FGFRL1,SPTLC3,METTL15,PRKAR1B,MARK2,EZH1,KAT8,CNBP,KANSL2,DUSP7,REPIN1,ADAM9,ANAPC13,ZNF827,SMUG1,MRPS30,MIB2,CDC27,RPS6KA1,GALNT4,TRERF1,PRPF40B,BRF1,MAPKAPK5,CENPX,SEC11C,TAF15,APBB1,NSA2,MTRES1,CTCF,ATXN3,DYNC2H1
	GO:0044238	Primary metabolic process	0.16	0.0011	OTUD5,CDIPT,RNASEH2A,RAB3D,RPS12,MED28,KALRN,SGPP1,RARA,COQ3,BRD8,APC,AKIRIN2,USP37,HUS1,BRIP1,FAM8A1,BUD13,LPIN2,ARG2,KMT2C,GATB,TARBP2,NEK8,SDE2,NDUFS6,MARCHF6,CDK16,NDUFB9,ZC3H12C,NIFK,RNF126,MAP4K2,MYORG,TERF2IP,PPIC,ZCCHC4,RNFT1,GON7,RAMAC,FEM1B,TSPAN5,UTP23,XXYLT1,ERCC4,ATAD5,RMI1,PIBF1,GEMI

					N4,ENO3,CASP9,SPPL2C,SMAD3,PDE2A,KLHL7,FCF1,PTMA,TRAF3IP2,RPL27A,NRDE2,FBXL3,RPS26,UBL5,PCBP2,PJA2,MT-ND4,DCLRE1A,UST,DPM3, PLEKHA1,FMR1,NCOA3, DOLK,RLF,SLC25A25,BAK1,HMGCL,TSR2,STK19, FBXO6,KDM2B,B3GALT6,NFX1,NHLRC3,RREB1,NADK2,NCBP3,RNF34,RPL14,FGFRL1,SPTLC3,METTL15,PRKAR1B,MARK2,EZH1,KAT8,CNBP,KANSL2,DUSP7,REPIN1,ADAM9,ANAPC13,ZNF827,SMUG1,MRPS30,MIB2,CDC27,RPS6KA1,GALNT4,TRERF1,PRPF40B,BRF1,MAPKAPK5,CENPX,SEC11C,TAF15,APBB1,NSA2,MTRES1,CTCF,ATXN3,DYNC2H1
	GO:0043412	Macromolecule modification	0.24	0.0351	OTUD5,RAB3D,KALRN,RARA,BRD8,USP37,HUS1,KMT2C,NEK8,SDE2,MARCHF6, CDK16,RNF126,MAP4K2,PPIC,ZCCHC4,RNFT1,GON7,RAMAC, FEM1B,XXYLT1,PIBF1,KLHL7,TRAF3IP2,FBXL3,UBL5,PJA2,UST,DPM3,NCOA3,DOLK,RLF,BAK1,STK19,FBXO6,KDM2B,B3GALT6,NFX1,NHLRC3,RNF34,FGFRL1,METTL15,PRKAR1B,MARK2,EZH1,KAT8,KANSL2,DUSP7,ANAPC13,SMUG1,MIB2,CDC27,RPS6KA1,GALNT4,TRERF1,MAPKAPK5,APBB1,CTCF,ATXN3
	GO:0043227	Membrane-bounded organelle	0.17	1.00E-37	COX15,OTUD5,RIN3,CDIPT,MGA,RNASEH2A,RAB3D,RPS12,NPRL2,MED28, CCDC92,KALRN,SGPP1,EAPP,KRT5,RARA,COQ3,BRD8,SNAP25,APC,AKIRIN2,USP37,HUS1,BRIP1,FAM8A1,BUD13,CLCN3,LPIN2,ARG2,SPG11,RAB11A,MTFR1,KMT2C,GATB,IFT46,TUBB4A,PITX1,HPS5,TARBP2,ZNF280D,NEK8,SDE2,LIMD1,NDUFS6,MARCHF6,CDK16,NDUFB9,ZC3H12C,LDHAL6A,SCLT1,PLEKHH2, GRM1,NUBP1,NIFK,FZD7,MTERF3,RNF126,MAP4K2,BBS5,MICALL2,MYORG,POLR2M,TERF2IP,CA4,PPIC,ZCCHC4,RNFT1,GON7,SDC2,RAMAC, FEM1B,TSPAN5,UTP23,XXYLT1,SPNS1,ERCC4,ISCU,ARR3,ATAD5,ZNF609,RMI1,PIBF1,MRFAP1,GEMIN4,ZKSCAN1,ENO3,MAP11,NR2F1,COX14,TMEM39A, SP1,CASP9,SPPL2C,OSBP2,SMAD3,TMEM179B,ABCC5,COQ6,PDE2A,EBAG9,TUBB4B,ENSA,LAMTOR4,KLHL7,FCF1,PTMA,TRAF3IP2,RPL27A,

					NRDE2,FBXL3,RPS26,DYNLRB1,UBL5,SLC25A29,PCBP2,MT-CO1,PJA2,MT-ND4,DCLRE1A,COQ8A,FGFR1OP,UST,XPR1,SUCO,SYT11,DPM3,PLEKHA1,MCL1,SH3BGRL2,FMR1,MINPP1,NCOA3,SURF6,GPR107,DOLK,RLF,SLC25A25,SLC31A1,BAK1,HMGCL,PBX2,TSR2,STK19,FBXO6,KDM2B,CTNNBIP1,ATG2A,RGP1,B3GALT6,NFX1,NHLRC3,RREB1,SDC1,NADK2,NCBP3,RNF34,VTI1A,PRC1,RPL14,EAF1,FGFRL1,SPTLC3,DTNB,METTL15,PRKAR1B,MARK2,MYO7A,CPLANE1,BCAP31,MAPKBP1,ZNF7,WDR20,CCN1,SATB2,NR2C2AP,EZH1,KAT8,CNBP,KLC1,KANSL2,DUSP7,REPIN1,UBN2,ADAM9,ANAPC13,ZNF827,SORCS2,SMUG1,MRPS30,COX18,MIB2,WWC1,CCNC,SLC25A30,TXNRD3,ZNF706,SPA17,CFL1,MEX3A,CDC27,RPS6KA1,GALNT4,TRERF1,GRAMD1A,PRR14,CITED2,MYL6,PRPF40B,BRF1,MAPKAPK5,EML5,ZNF592,CNN2,WWOX,ACTG1,CENPX,COX6B1,UQCR11,SEC11C,TAF15,UQCC2,APBB1,SCOC,NSA2,MTRES1,BTBD8,RTTN,CTCF,MYO15A,ATXN3,GET1,DYNC2H1,SLC25A52
	GO:0043229	Intracellular organelle	0.16	1.92E-28	COX15,OTUD5,RIN3,CDIPT,MGA,RNASEH2A,RAB3D,RPS12,NPRL2,MED28,CCDC92,KALRN,SGPP1,EAPP,KRT5,RARA,COQ3,BRD8,SNAP25,APC,AKIRIN2,USP37,HUS1,BRIP1,FAM8A1,BUD13,CLCN3,LPIN2,ARG2,SPG11,RAB11A,MTFR1,KMT2C,GATB,IFT46,TUBB4A,PITX1,HPS5,TARBP2,ZNF280D,NEK8,SDE2,LIMD1,NDUFS6,MARCHF6,CDK16,NDUFB9,ZC3H12C,SCLT1,PLEKHH2,GRM1,NUBP1,NIFK,FZD7,MTERF3,RNF126,MAP4K2,BBS5,MICALL2,MYORG,POLR2M,TERF2IP,CA4,PPIC,ZCCHC4,RNFT1,GON7,SDC2,RAMAC,FEM1B,TSPAN5,UTP23,XXYLT1,SPNS1,ERCC4,ISCU,ATAD5,ZNF609,RMI1,PIBF1,MRFAP1,GEMIN4,ZKSCAN1,MAP11,NR2F1,COX14,TMEM39A,SP1,CASP9,SPPL2C,OSBP2,SMAD3,TMEM179B,ABCC5,COQ6,PDE2A,EBAG9,TUBB4B,ENSA,LAMTOR4,KLHL7,FCF1,PTMA,TRAF3IP2,RPL27A,NRDE2,FBXL3,RPS26,DYNLRB1,UBL5,SLC25A29,PCBP2,MT-CO1,PJA2,MT-ND4,DCLRE1A,COQ8A,FGFR1OP,

					UST,XPR1,SUCO,SYT11,DPM3,PLEKHA1,MCL1,SH3BGRL2,FMR1,MINPP1,NCOA3,SURF6,GPR107,DOLK,RLF,SLC25A25,SLC31A1,BAK1,HMGCL,PBX2,TSR2,STK19,FBXO6,KDM2B,CTNNBIP1,ATG2A,RGP1,B3GALT6,NFX1,NHLRC3,RREB1,SDC1,NADK2,NCBP3,RNF34,VTI1A,PRC1,RPL14,EAF1,FGFRL1,SPTLC3,DTNB,METTL15,PRKAR1B,MARK2,MYO7A,BCAP31,MAPKBP1,ZNF7,WDR20,CCN1,SATB2,NR2C2AP,EZH1,KAT8,CNBP,KLC1,KANSL2,DUSP7,REPIN1,UBN2,ANAPC13,ZNF827,SORCS2,SMUG1,MRPS30,COX18,MIB2,WWC1,CCNC,SLC25A30,TXNRD3,ZNF706,CFL1,MEX3A,CDC27,RPS6KA1,GALNT4,TRERF1,GRAMD1A,PRR14,CITED2,MYL6,PRPF40B,BRF1,MAPKAPK5,EML5,ZNF592,CNN2,WWOX,ACTG1,CENPX,COX6B1,UQCR11,SEC11C,TAF15,UQCC2,APBB1,SCOC,NSA2,MTRES1,BTBD8,RTTN,CTCF,MYO15A,ATXN3,GET1,DYNC2H1,SLC25A52
	GO:0043231	Intracellular membrane-bounded organelle	0.18	1.60E-26	COX15,OTUD5,RIN3,CDIPT,MGA,RNASEH2A,RAB3D,RPS12,NPRL2,MED28,CCDC92,KALRN,SGPP1,EAPP,KRT5,RARA,COQ3,BRD8,SNAP25,APC,AKIRIN2,USP37,HUS1,BRIP1,FAM8A1,BUD13,CLCN3,LPIN2,ARG2,SPG11,RAB11A,MTFR1,KMT2C,GATB,TUBB4A,PITX1,HPS5,TARBP2,ZNF280D,NEK8,SDE2,LIMD1,NDUFS6,MARCHF6,CDK16,NDUFB9,ZC3H12C,PLEKHH2,GRM1,NUBP1,NIFK,FZD7,MTERF3,RNF126,MAP4K2,MICALL2,MYORG,POLR2M,TERF2IP,CA4,PPIC,ZCCHC4,RNFT1,GON7,SDC2,RAMAC,FEM1B,TSPAN5,UTP23,XXYLT1,SPNS1,ERCC4,ISCU,ATAD5,ZNF609,RMI1,PIBF1,MRFAP1,GEMIN4,ZKSCAN1,MAP11,NR2F1,COX14,TMEM39A,SP1,CASP9,SPPL2C,OSBP2,SMAD3,TMEM179B,ABCC5,COQ6,PDE2A,EBAG9,TUBB4B,ENSA,LAMTOR4,KLHL7,FCF1,PTMA,TRAF3IP2,RPL27A,NRDE2,FBXL3,RPS26,UBL5,SLC25A29,PCBP2,MT-CO1,PJA2,MT-ND4,DCLRE1A,COQ8A,FGFR1OP,UST,XPR1,SUCO,SYT11, DPM3,PLEKHA1,MCL1,SH3BGRL2,FMR1,MINPP1,NCOA3,SURF6,GPR107,DOLK,RLF,SLC25A25,SLC31A1,BAK1,HMGCL,PBX2,TSR2,STK19,FBXO6,KDM2B,CTNNBIP1,ATG2A,RGP1,

					B3GALT6,NFX1,NHLRC3,RREB1,SDC1,NADK2,NCBP3,RNF34,VTI1A,PRC1,EAF1,FGFRL1,SPTLC3,DTNB,METTL15,PRKAR1B,MARK2,MYO7A,BCAP31,MAPKBP1,ZNF7,WDR20,CCN1,SATB2,NR2C2AP,EZH1,KAT8,CNBP,KLC1,KANSL2,DUSP7,REPIN1,UBN2,ANAPC13,ZNF827,SORCS2,SMUG1,MRPS30,COX18,MIB2,WWC1,CCNC,SLC25A30,TXNRD3,ZNF706,CFL1,MEX3A,CDC27,RPS6KA1,GALNT4,TRERF1,GRAMD1A,PRR14,CITED2,PRPF40B,BRF1,MAPKAPK5,ZNF592,CNN2,WWOX,ACTG1,CENPX,COX6B1,UQCR11,SEC11C,TAF15,UQCC2,APBB1,SCOC,NSA2,MTRES1,BTBD8,CTCF,ATXN3,GET1,DYNC2H1,SLC25A52
	GO:0005622	Intracellular anatomical structure	0.12	8.68E-23	COX15,OTUD5,RIN3,CDIPT,MGA,RNASEH2A,RAB3D,RPS12,NPRL2,MED28,CCDC92,KALRN,SGPP1,EAPP,KRT5,RARA,COQ3,BRD8,SNAP25,APC,AKIRIN2,USP37,HUS1,BRIP1,FAM8A1,BUD13,CLCN3,LPIN2,ARG2,SPG11,RAB11A,MTFR1,KMT2C,GATB,IFT46,TUBB4A,PITX1,HPS5,TARBP2,ZNF280D,NEK8,SDE2,LIMD1,NDUFS6,MARCHF6,CDK16,NDUFB9,ZC3H12C,LDHAL6A,SCLT1,PLEKHH2,GRM1,NUBP1,NIFK,FZD7,MTERF3,RNF126,MAP4K2,BBS5,MICALL2,MYORG,POLR2M,TERF2IP,CA4,PPIC,ZCCHC4,RNFT1,GON7,SDC2,RAMAC,FEM1B,TSPAN5,UTP23,XXYLT1,SPNS1,ERCC4,ISCU,ARR3,ATAD5,ZNF609,RMI1,PIBF1,MRFAP1,GEMIN4,ZKSCAN1,ENO3,MAP11,NR2F1,COX14,TMEM39A,SP1,CASP9,SPPL2C,OSBP2,SMAD3,TMEM179B,ABCC5,COQ6,PDE2A,EBAG9,TUBB4B,ENSA,LAMTOR4,KLHL7,FCF1,PTMA,TRAF3IP2,RPL27A,NRDE2,FBXL3,RPS26,DYNLRB1,UBL5,SLC25A29,PCBP2,MT-CO1,PJA2,MT-ND4, DCLRE1A,COQ8A,FGFR1OP,UST,XPR1,SUCO,SYT11,DPM3,PLEKHA1,MCL1,SH3BGRL2,FMR1,MINPP1,NCOA3,SURF6,GPR107,DOLK,RLF,SLC25A25,SLC31A1,BAK1,HMGCL,PBX2,TSR2,STK19,FBXO6,KDM2B,CTNNBIP1,ATG2A,RGP1,B3GALT6,NFX1,NHLRC3,RREB1,SDC1,NADK2,NCBP3,RNF34,VTI1A,PRC1,RPL14,EAF1,FGFRL1,SPTLC3,DTNB,METTL15,PRKAR1B,MARK2,MYO7A,BCAP31,MAPKBP1,ZNF7,WDR20,CCN1,SATB2,NR2C2AP,EZH1,KAT8,CN

					BP,KLC1,KANSL2,DUSP7,REPIN1,UBN2,ANAPC13,ZNF827,SORCS2,SMUG1,MRPS30,COX18,MIB2,WWC1,CCNC,SLC25A30,TXNRD3,ZNF706,SPA17,CFL1,MEX3A,CDC27,RPS6KA1,GALNT4,TRERF1,GRAMD1A,PRR14,CITED2,MYL6,PRPF40B,BRF1,MAPKAPK5,EML5,ZNF592,CNN2,WWOX,ACTG1,CENPX,COX6B1,UQCR11,SEC11C,TAF15,UQCC2,APBB1,SCOC,NSA2,MTRES1,BTBD8,RTTN,CTCF,MYO15A,ATXN3,GET1,DYNC2H1,SLC25A52
	GO:0005737	Cytoplasm	0.12	1.94E-08	COX15,OTUD5,RIN3,CDIPT,RNASEH2A,RAB3D,RPS12,NPRL2,MED28,CCDC92,KALRN,SGPP1,EAPP,KRT5,RARA,COQ3,BRD8,SNAP25,APC,AKIRIN2,USP37,HUS1,BRIP1,FAM8A1,CLCN3,LPIN2,ARG2,SPG11,RAB11A,MTFR1,GATB,IFT46,TUBB4A,PITX1,HPS5,TARBP2,NEK8,SDE2,LIMD1,NDUFS6,MARCHF6,CDK16,NDUFB9,ZC3H12C,LDHAL6A,SCLT1,PLEKHH2,NUBP1,NIFK,FZD7,MTERF3,RNF126,MAP4K2,BBS5,MICALL2,MYORG,TERF2IP,CA4,PPIC,ZCCHC4,RNFT1,GON7,SDC2,FEM1B,TSPAN5,XXYLT1,SPNS1,ISCU,ARR3,PIBF1,MRFAP1,GEMIN4,ENO3,MAP11,NR2F1,COX14,TMEM39A,SP1,CASP9,SPPL2C,OSBP2,SMAD3,TMEM179B,ABCC5,COQ6,PDE2A,EBAG9,TUBB4B,ENSA,LAMTOR4,KLHL7,PTMA,TRAF3IP2,RPL27A,FBXL3,RPS26,DYNLRB1,UBL5,SLC25A29,PCBP2,MT-CO1,PJA2,MT-ND4,COQ8A,FGFR1OP,UST,XPR1, SUCO,SYT11,DPM3,PLEKHA1,MCL1,FMR1,MINPP1,NCOA3,GPR107,DOLK,SLC25A25,SLC31A1,BAK1,HMGCL,FBXO6,CTNNBIP1,ATG2A,RGP1,B3GALT6,NFX1,NHLRC3,RREB1,SDC1,NADK2,NCBP3,RNF34,VTI1A,PRC1,RPL14,FGFRL1,SPTLC3,DTNB,METTL15,PRKAR1B,MARK2,MYO7A,BCAP31,MAPKBP1,CCN1,CNBP,KLC1,KANSL2,DUSP7,SORCS2,SMUG1,MRPS30,COX18,MIB2,WWC1,SLC25A30,TXNRD3,ZNF706,SPA17,CFL1,MEX3A,CDC27,RPS6KA1,GALNT4,TRERF1,GRAMD1A,CITED2,MYL6,MAPKAPK5,EML5,CNN2,WWOX,ACTG1,COX6B1,UQCR11,SEC11C,TAF15,UQCC2,APBB1,SCOC,MTRES1,BTBD8,RTTN,MYO15A,ATXN3,GET1,DYNC2H1,SLC25A52

	GO:0070013	Intracellular organelle lumen	0.23	2.52E-08	COX15,MGA,RNASEH2A,RPS12,MED28,CCDC92,KALRN,RARA,COQ3,BRD8,APC,AKIRIN2,USP37,HUS1,BRIP1,BUD13,ARG2,SPG11,KMT2C,TARBP2,SDE2,LIMD1,PLEKHH2,NIFK,RNF126,POLR2M,TERF2IP,ZCCHC4,GON7,SDC2,FEM1B,TSPAN5,UTP23,ERCC4,ISCU,ZNF609,RMI1,MRFAP1,GEMIN4,NR2F1,SP1,SMAD3,TMEM179B,ABCC5,PDE2A,TUBB4B,ENSA,KLHL7,FCF1,PTMA,NRDE2,FBXL3,RPS26,PCBP2,DCLRE1A,PLEKHA1,MCL1,SH3BGRL2,FMR1,MINPP1,NCOA3,SURF6,GPR107,HMGCL,STK19,KDM2B,CTNNBIP1,NFX1,NHLRC3,RREB1,SDC1,NADK2,NCBP3,RNF34,PRC1,EAF1,METTL15,MARK2,MAPKBP1,WDR20,CCN1,SATB2,NR2C2AP,EZH1,KAT8,KANSL2,DUSP7,REPIN1,UBN2,SMUG1,MRPS30,CCNC,TXNRD3,CFL1,CDC27,RPS6KA1,TRERF1,PRR14,CITED2,PRPF40B,BRF1,MAPKAPK5,CNN2,WWOX,CENPX,TAF15,UQCC2,APBB1,SCOC,NSA2,MTRES1,BTBD8,CTCF,ATXN3
	GO:0005654	Nucleoplasm	0.27	1.11E-07	COX15,MGA,RNASEH2A,RPS12,MED28,CCDC92,KALRN,RARA,BRD8,APC,AKIRIN2,USP37,HUS1,BRIP1,BUD13,KMT2C,TARBP2,SDE2,LIMD1,PLEKHH2,NIFK,RNF126,POLR2M,TERF2IP,GON7,FEM1B,ERCC4,ZNF609,RMI1,MRFAP1,GEMIN4,NR2F1,SP1,SMAD3,TMEM179B,ENSA,KLHL7,FCF1,PTMA,NRDE2,FBXL3,RPS26,PCBP2,DCLRE1A,PLEKHA1,MCL1,SH3BGRL2,FMR1,NCOA3,SURF6,GPR107,STK19,KDM2B,CTNNBIP1,NFX1,RREB1,NCBP3,RNF34,PRC1,EAF1,MARK2,MAPKBP1,WDR20,SATB2,NR2C2AP,EZH1,KAT8,KANSL2,DUSP7,REPIN1,UBN2,SMUG1,CCNC,TXNRD3,CDC27,RPS6KA1,TRERF1,PRR14,CITED2,PRPF40B,BRF1,MAPKAPK5,WWOX,CENPX,TAF15,UQCC2,APBB1,SCOC,BTBD8,CTCF,ATXN3
	GO:0031981	Nuclear lumen	0.25	1.14E-07	COX15,MGA,RNASEH2A,RPS12,MED28,CCDC92,KALRN,RARA,BRD8,APC,AKIRIN2,USP37,HUS1,BRIP1,BUD13,SPG11,KMT2C,TARBP2,SDE2,LIMD1,PLEKHH2,NIFK,RNF126,POLR2M,TERF2IP,ZCCHC4,GON7,FEM1B,UTP23,ERCC4,ZNF609,RMI1,MRFAP1,GEMIN4,NR2F1,SP1,SMAD3,TMEM179B,ENSA,KLHL7,FCF1,PTMA,NRDE2,FBXL3,RPS26,PCBP2,DCLRE1A,PLEKHA1,MCL1,SH3BGRL2,F

					MR1,NCOA3,SURF6,GPR107,STK19,KDM2B,CTNNBIP1,NFX1,RREB1,NCBP3,RNF34,PRC1,EAF1,MARK2,MAPKBP1,WDR20,SATB2,NR2C2AP,EZH1,KAT8,KANSL2,DUSP7,REPIN1,UBN2,SMUG1,CCNC,TXNRD3,CFL1,CDC27,RPS6KA1,TRERF1, PRR14,CITED2,PRPF40B,BRF1,MAPKAPK5,WWOX,CENPX,TAF15,UQCC2,APBB1, SCOC,NSA2,BTBD8,CTCF,ATXN3
	GO:0005634	Nucleus	0.17	1.64E-06	COX15,OTUD5,MGA,RNASEH2A,RPS12,MED28,CCDC92,KALRN,EAPP,KRT5, RARA,BRD8,APC,AKIRIN2,USP37,HUS1,BRIP1,BUD13,LPIN2,SPG11,KMT2C,TUBB4A,PITX1,TARBP2,ZNF280D,NEK8,SDE2,LIMD1,CDK16,ZC3H12C,PLEKHH2,GRM1,NUBP1,NIFK,RNF126,MYORG,POLR2M,TERF2IP,ZCCHC4,GON7,RAMAC,FEM1B,UTP23,ERCC4,ISCU,ATAD5,ZNF609,RMI1,PIBF1,MRFAP1,GEMIN4,ZKSCAN1,NR2F1,SP1,CASP9,SMAD3,TMEM179B,PDE2A,TUBB4B,ENSA,KLHL7,FCF1,PTMA,TRAF3IP2,NRDE2,FBXL3,RPS26,UBL5,PCBP2,DCLRE1A,FGFR1OP,PLEKHA1,MCL1, SH3BGRL2,FMR1,NCOA3,SURF6,GPR107,RLF,PBX2,TSR2,STK19,KDM2B,CTNNBIP1,NFX1,RREB1,NCBP3,RNF34,PRC1,EAF1,DTNB,MARK2,MAPKBP1,ZNF7,WDR20, SATB2,NR2C2AP,EZH1,KAT8,CNBP,KANSL2,DUSP7,REPIN1,UBN2,ANAPC13,ZNF827,SMUG1,WWC1,CCNC,TXNRD3,ZNF706,CFL1,MEX3A,CDC27,RPS6KA1,TRERF1,PRR14,CITED2,PRPF40B,BRF1,MAPKAPK5,ZNF592, WWOX,ACTG1,CENPX,TAF15,UQCC2,APBB1,SCOC,NSA2,BTBD8,CTCF,ATXN3,GET1
	GO:0032991	Protein-containing complex	0.2	1.51E-05	COX15,MGA,RNASEH2A,RPS12,NPRL2,MED28,RARA,COQ3,BRD8,SNAP25, APC,AKIRIN2,HUS1,BRIP1,FAM8A1,BUD13,RAB11A,KMT2C,GATB,IFT46,PITX1,HPS5,TARBP2,LIMD1,NDUFS6,MARCHF6,CDK16,NDUFB9,SCLT1,GRM1,BBS5, POLR2M,TERF2IP,GON7,RAMAC,FEM1B,UTP23,ERCC4,ISCU,ATAD5,RMI1,GEMIN4,ENO3,MAP11,SP1,CASP9,SMAD3,COQ6,LAMTOR4,KLHL7,FCF1, RPL27A,FBXL3,RPS26,DYNLRB1,MT-CO1,MT-ND4,DPM3,MCL1,FMR1,

					NCOA3,BAK1,HMGCL,PBX2,FBXO6,CTNNBIP1,RGP1,SDC1,NCBP3,VTI1A,RPL14, EAF1,SPTLC3,PRKAR1B,MYO7A,SATB2,EZH1,KAT8,KLC1,KANSL2,REPIN1,ANA PC13,ZNF827,MRPS30,WWC1,CCNC,CDC27,TRERF1,CITED2,MYL6, PRPF40B,BRF1,MAPKAPK5,WWOX,ACTG1,CENPX,COX6B1,UQCR11,SEC11C,NS A2,MYO15A,GET1,DYNC2H1
	GO:0043232	Intracellular non-membrane-bounded organelle	0.18	0.0007	MGA,RAB3D,RPS12,MED28,CCDC92,KALRN,KRT5,RARA,BRD8,SNAP25,APC,AKI RIN2,HUS1,SPG11,RAB11A,IFT46,TUBB4A,PITX1,NEK8,LIMD1,CDK16,ZC3H12C,S CLT1,PLEKHH2,NUBP1,NIFK,BBS5,MICALL2,TERF2IP,ZCCHC4,GON7,UTP23, ERCC4,ATAD5,RMI1,PIBF1,GEMIN4,MAP11,SP1,SMAD3,TMEM179B,TUBB4B, KLHL7,FCF1,RPL27A,NRDE2,RPS26,DYNLRB1,PJA2,DCLRE1A,FGFR1OP,FMR1,N COA3,SURF6,PBX2,KDM2B,ATG2A,NFX1,RREB1,PRC1,RPL14,MARK2,MYO7A,BC AP31,MAPKBP1,SATB2,EZH1,KAT8,KLC1,KANSL2,REPIN1,ZNF827,SMUG1, MRPS30,CFL1,MEX3A,CDC27,TRERF1,PRR14,CITED2,MYL6,MAPKAPK5,EML5, CNN2,ACTG1,CENPX,UQCC2,NSA2,RTTN,CTCF,MYO15A,ATXN3,DYNC2H1
	GO:0031090	Organelle membrane	0.21	0.0013	COX15,CDIPT,RAB3D,NPRL2,SGPP1,COQ3,SNAP25,BRIP1,FAM8A1,CLCN3,LPIN2 ,SPG11,RAB11A,NDUFS6,MARCHF6,NDUFB9,FZD7,MTERF3,MAP4K2,BBS5,MYO RG,CA4,RNFT1,XXYLT1,SPNS1,COX14,TMEM39A,SPPL2C,SMAD3,TMEM179B,AB CC5,COQ6,PDE2A,EBAG9,LAMTOR4,RPS26,SLC25A29,MT-CO1,PJA2,MT-ND4,COQ8A,UST,SUCO,SYT11,DPM3, MCL1,SH3BGRL2,DOLK,SLC25A25,BAK1, ATG2A,RGP1,B3GALT6,VTI1A,SPTLC3,MYO7A,BCAP31,SORCS2,MRPS30,COX18,S LC25A30,GALNT4,GRAMD1A,COX6B1,UQCR11,SEC11C,UQCC2,SCOC,ATXN3,G ET1,SLC25A52

	GO:0005743	Mitochondrial inner membrane	0.51	0.0015	COX15,COQ3,NDUFS6,NDUFB9,SPNS1,COQ6,PDE2A,SLC25A29,MT-CO1, MT-ND4,COQ8A,SLC25A25,MRPS30,COX18,SLC25A30,COX6B1,UQCR11,UQCC2,SLC25A52
	GO:0031966	Mitochondrial membrane	0.43	0.0016	COX15,COQ3,NDUFS6,NDUFB9,MTERF3,SPNS1,COX14,COQ6,PDE2A,SLC25A29, MT-CO1,MT-ND4,COQ8A,MCL1,SLC25A25,BAK1,MRPS30,COX18,SLC25A30, COX6B1,UQCR11, UQCC2,ATXN3,SLC25A52
	GO:0019866	Organelle inner membrane	0.48	0.002	COX15,COQ3,NDUFS6,NDUFB9,SPNS1,SMAD3,COQ6,PDE2A,SLC25A29,MT-CO1,MT-ND4,COQ8A,SLC25A25,MRPS30,COX18,SLC25A30,COX6B1,UQCR11, UQCC2,SLC25A52
	GO:0005739	Mitochondrion	0.28	0.0092	COX15,COQ3,BRD8,ARG2,MTFR1,GATB,NDUFS6,NDUFB9,MTERF3,SPNS1,ISCU, COX14,CASP9,COQ6,PDE2A,SLC25A29,MT-CO1,MT-ND4,COQ8A,MCL1, SLC25A25,BAK1,HMGCL, NADK2,METTL15,MARK2,BCAP31,MRPS30,COX18, SLC25A30,TXNRD3,WWOX,COX6B1,UQCR11,UQCC2,MTRES1,ATXN3,SLC25A52
	GO:0005746	Mitochondrial respirasome	0.8	0.0164	COX15,NDUFS6,NDUFB9,MT-CO1,MT-ND4,COX6B1,UQCR11
	GO:0140535	Intracellular protein-containing complex	0.38	0.0176	RNASEH2A,BRD8,FAM8A1,GATB,MARCHF6,POLR2M,RAMAC,FEM1B,MAP11,LAMTOR4,KLHL7,FBXL3,DPM3,FMR1,FBXO6,RGP1, NCBP3,PRKAR1B,KAT8,KANSL2,ANAPC13,CDC27
	GO:1902494	Catalytic complex	0.27	0.0287	MGA,BRD8,FAM8A1,KMT2C,TARBP2,NDUFS6,MARCHF6,CDK16,NDUFB9, POLR2M,RAMAC,FEM1B,ATAD5,ENO3,CASP9,KLHL7,FBXL3,DYNLRB1,MT-

					CO1,MT-ND4,DPM3,FBXO6,SPTLC3,SATB2,EZH1,KAT8,KANSL2,ANAPC13, CCNC,CDC27,TRERF1,UQCR11,SEC11C,DYNC2H1
	GO:0012505	Endomembrane system	0.15	0.0325	RIN3,CDIPT,RAB3D,RPS12,SGPP1,SNAP25,APC,BRIP1,FAM8A1,CLCN3,LPIN2,RAB11A,HPS5,SDE2,MARCHF6,CDK16,FZD7,MAP4K2,MICALL2,MYORG,POLR2M, CA4,RNFT1,SDC2,TSPAN5,XXYLT1,MAP11,TMEM39A,SPPL2C,OSBP2,SMAD3,TMEM179B,ABCC5,COQ6,PDE2A,EBAG9,TUBB4B,LAMTOR4,RPL27A,RPS26,PJA2, UST,XPR1,SUCO,SYT11,DPM3,SH3BGRL2,MINPP1,GPR107,DOLK,SLC31A1,BAK1, FBXO6,ATG2A,RGP1,B3GALT6,NHLRC3,SDC1,RNF34,VTI1A,FGFRL1,SPTLC3,PRKAR1B,BCAP31,CCN1,CNBP,SORCS2,MIB2,TXNRD3,GALNT4,GRAMD1A,CNN2,WWOX,SEC11C,APBB1,SCOC,BTBD8,GET1,DYNC2H1
	GO:0031967	Organelle envelope	0.29	0.0407	COX15,COQ3,BRIP1,NDUFS6,NDUFB9,MTERF3,MYORG,POLR2M,SPNS1,COX14, SMAD3,COQ6,PDE2A,SLC25A29,MT-CO1,MT-ND4,COQ8A,MCL1,SH3BGRL2, SLC25A25,BAK1, MRPS30,COX18,SLC25A30,COX6B1,UQCR11,UQCC2, ATXN3,SLC25A52
	CL:11063	Respiratory electron transport, ATP synthesis by chemiosmotic coupling, and heat production by uncoupling proteins., and	0.74	0.042	COX15,NDUFS6,NDUFB9,COX14,EBAG9,MT-CO1,MT-ND4, COX18,COX6B1, UQCR11,UQCC2

		Cytochrome complex			
	CL:11065	Respiratory electron transport, ATP synthesis by chemiosmotic coupling, and heat production by uncoupling proteins., and Cytochrome complex	0.72	0.0498	COX15,NDUFS6,NDUFB9,COX14,MT-CO1,MT-ND4, COX18,COX6B1, UQCR11,UQCC2
	hsa04714	Thermogenesis	0.65	0.0084	COX15,NDUFS6,NDUFB9,COX14,SLC25A29,MT-CO1,MT-ND4,COX18 ,RPS6KA1,ACTG1, COX6B1,UQCR11
	hsa05010	Alzheimer disease	0.52	0.0196	APC,TUBB4A,NDUFS6,NDUFB9,FZD7,CASP9,TUBB4B,MT-CO1,MT-ND4,ATG2A, KLC1,COX6B1,UQCR11,APBB1
	hsa05014	Amyotrophic lateral sclerosis	0.5	0.0313	SPG11,TUBB4A,NDUFS6,NDUFB9,CASP9,TUBB4B,MT-CO1,MT-ND4,ATG2A, KLC1, ACTG1,COX6B1,UQCR11
	hsa05016	Huntington disease	0.54	0.0313	TUBB4A,NDUFS6,NDUFB9,SP1,CASP9,TUBB4B,MT-CO1,MT-ND4,ATG2A,KLC1, COX6B1,UQCR11

	hsa05012	Parkinson disease	0.55	0.0445	TUBB4A,NDUFS6,NDUFB9,CASP9,TUBB4B,MT-CO1,MT-ND4,KLC1, COX6B1,UQCR11
	HP:0002060	Abnormal cerebral morphology	0.36	0.00097	COX15,OTUD5,RNASEH2A,NPRL2,APC,BRIP1,SPG11,RAB11A,KMT2C, TUBB4A,PITX1,NDUFS6,NDUFB9,MYORG,ERCC4,PIBF1,GEMIN4,MAP11, NR2F1,COX14,SMAD3,PDE2A,FBXL3,RPS26,MT-CO1,MT-ND4,COQ8A,XPR1, FMR1,DOLK,HMGCL,TSR2,RREB1,NADK2,PRKAR1B,MYO7A,CPLANE1,BCAP31, SATB2,KAT8,BRF1,WWOX,ACTG1,COX6B1,RTTN,CTCF,ATXN3,DYNC2H1
	HP:0002977	Aplasia/Hypoplasia involving the central nervous system	0.37	0.00097	COX15,OTUD5,RNASEH2A,APC,BRIP1,SPG11,RAB11A,KMT2C,TUBB4A,PITX1, NDUFS6,NDUFB9,GRM1,MYORG,ERCC4,PIBF1,GEMIN4,MAP11,NR2F1,PDE2A, FBXL3,RPS26,MT-CO1,MT-ND4,XPR1,FMR1,DOLK,HMGCL,TSR2,RREB1,NADK2, PRKAR1B,MYO7A, CPLANE1,BCAP31,SATB2,KAT8,BRF1,WWOX,ACTG1,RTTN, CTCF,DYNC2H1
	HP:0007364	Aplasia/Hypoplasia of the cerebrum	0.4	0.00097	COX15,OTUD5,RNASEH2A,APC,BRIP1,SPG11,RAB11A,KMT2C,TUBB4A,PITX1, NDUFS6,NDUFB9,MYORG,ERCC4,PIBF1,GEMIN4,MAP11,NR2F1,PDE2A,FBXL3, RPS26,MT-CO1,MT-ND4,XPR1,DOLK,HMGCL,TSR2,RREB1,NADK2,CPLANE1, BCAP31,SATB2,KAT8,BRF1,WWOX,ACTG1,RTTN,CTCF,DYNC2H1
	HP:0000496	Abnormality of eye movement	0.37	0.00098	COX15,OTUD5,RNASEH2A,KRT5,SNAP25,APC,BRIP1,SPG11,TUBB4A,HPS5, NDUFS6,NDUFB9,GRM1,BBS5,MYORG,CA4,ERCC4,PIBF1,NR2F1,SMAD3,KLHL7, FBXL3,RPS26,MT-CO1,MT-ND4,COQ8A,FMR1,TSR2,RREB1,NADK2,MYO7A, CPLANE1,BCAP31,SATB2,KAT8,BRF1,WWOX,RTTN,CTCF,ATXN3,GET1
	HP:0012443	Abnormality of brain morphology	0.31	0.00098	COX15,OTUD5,RNASEH2A,NPRL2,KRT5,APC,BRIP1,SPG11,RAB11A,KMT2C, TUBB4A,PITX1,NDUFS6,NDUFB9,GRM1,MYORG,ERCC4,PIBF1,GEMIN4, MAP11,NR2F1,COX14,SMAD3,PDE2A,FBXL3,RPS26,MT-CO1,MT-ND4,COQ8A,

					XPR1,DPM3,FMR1,DOLK,HMGCL,TSR2, B3GALT6,RREB1,NADK2,PRKAR1B, MYO7A,CPLANE1,BCAP31,SATB2,KAT8,CNBP,CITED2,BRF1,WWOX,ACTG1, COX6B1,RTTN,CTCF,ATXN3,DYNC2H1
	HP:0011446	Abnormality of higher mental function	0.29	0.003	COX15,OTUD5,RNASEH2A,NPRL2,SNAP25,APC,BRIP1,SPG11,RAB11A,KMT2C, TUBB4A,NDUFS6,MARCHF6,NDUFB9,GRM1,BBS5,MYORG,CA4,ERCC4,PIBF1,GE MIN4,MAP11,NR2F1,COX14,PDE2A,KLHL7,FBXL3,RPS26,MT-CO1,MT-ND4, COQ8A,XPR1,DPM3,FMR1,DOLK, HMGCL,TSR2,B3GALT6,RREB1,PRKAR1B, MYO7A,CPLANE1,BCAP31,SATB2,KAT8,BRF1,WWOX,ACTG1,COX6B1,UQCC2,RT TN,CTCF,ATXN3
	HP:0000077	Abnormality of the kidney	0.4	0.0032	OTUD5,RARA,APC,BRIP1,LPIN2,KMT2C,NEK8,NDUFS6,NDUFB9,BBS5,ERCC4,GE MIN4,MAP11,COX14,COQ6,TRAF3IP2,RPS26,MT-CO1,MT-ND4,TSR2,B3GALT6, RREB1,NADK2, CPLANE1,MAPKBP1,BRF1,WWOX,ACTG1,COX6B1,UQCC2, RTTN,DYNC2H1
	HP:0002118	Abnormal cerebral ventricle morphology	0.46	0.0032	OTUD5,RNASEH2A,KRT5,BRIP1,SPG11,RAB11A,KMT2C,GRM1,MYORG, ERCC4,PIBF1,MAP11,PDE2A,XPR1,B3GALT6,RREB1,NADK2,CPLANE1,KAT8,BRF 1,ACTG1,COX6B1,RTTN,CTCF,ATXN3,DYNC2H1
	HP:0000708	Behavioral abnormality	0.32	0.0033	COX15,OTUD5,RNASEH2A,NPRL2,KRT5,RARA,SNAP25,APC,SPG11,RAB11A, KMT2C,TUBB4A,NDUFS6,NDUFB9,GRM1,MYORG,CA4,ERCC4,GEMIN4,NR2F1, PDE2A,KLHL7,MT-CO1,MT-ND4,COQ8A,XPR1,DPM3,FMR1,HMGCL,B3GALT6, RREB1,NADK2,PRKAR1B, MYO7A,BCAP31,MAPKBP1,SATB2,KAT8,ADAM9, WWOX,ACTG1,UQCC2,RTTN,CTCF,ATXN3

	HP:0010935	Abnormality of the upper urinary tract	0.39	0.0037	OTUD5,RARA,APC,BRIP1,LPIN2,KMT2C,NEK8,NDUFS6,NDUFB9,BBS5,ERCC4,GEMIN4,MAP11,COX14,COQ6,TRAF3IP2,RPS26,MT-CO1,MT-ND4,TSR2,B3GALT6,RREB1,NADK2, CPLANE1,MAPKBP1,BRF1,WWOX,ACTG1,COX6B1,UQCC2,RTTN,DYNC2H1
	HP:0003011	Abnormality of the musculature	0.27	0.0043	COX15,OTUD5,RNASEH2A,KRT5,RARA,SNAP25,APC,LPIN2,SPG11,RAB11A,KMT2C,TUBB4A,PITX1,NDUFS6,MARCHF6,NDUFB9,GRM1,BBS5,ERCC4,ISCU,PIBF1,GEMIN4,ENO3,NR2F1,COX14,SMAD3,PDE2A,KLHL7,RPS26,MT-CO1,MT-ND4,COQ8A,DPM3,FMR1,DOLK,HMGCL, TSR2,B3GALT6,RREB1,NADK2,PRKAR1B,MYO7A,CPLANE1,SATB2,CNBP,CITED2,BRF1,WWOX,COX6B1,UQCC2,RTTN,CTCF,ATXN3
	HP:0012759	Neurodevelopmental abnormality	0.28	0.0043	COX15,OTUD5,RNASEH2A,NPRL2,KRT5,SNAP25,APC,BRIP1,SPG11,RAB11A,KMT2C,TUBB4A,NDUFS6,MARCHF6,NDUFB9,GRM1,BBS5,CA4,ERCC4,PIBF1,GEMIN4,MAP11,NR2F1,COX14,PDE2A,KLHL7,FBXL3,RPS26,MT-CO1,MT-ND4,COQ8A,DPM3,FMR1,HMGCL,TSR2, B3GALT6,RREB1,NADK2,PRKAR1B,MYO7A,CPLANE1,BCAP31,SATB2,KAT8,BRF1,WWOX,ACTG1,COX6B1,UQCC2,RTTN,CTCF,ATXN3
	HP:0033127	Abnormality of the musculoskeletal system	0.24	0.0043	COX15,OTUD5,RNASEH2A,KRT5,RARA,SNAP25,APC,BRIP1,LPIN2,SPG11,RAB11A,KMT2C,TUBB4A,PITX1,NEK8,NDUFS6,MARCHF6,NDUFB9,GRM1,BBS5,MYORG,CA4,ERCC4,ISCU,PIBF1,GEMIN4,ENO3,MAP11,NR2F1,COX14,SMAD3,PDE2A,KLHL7,TRAF3IP2,FBXL3,RPS26,MT-CO1,MT-ND4,COQ8A,XPR1,DPM3,FMR1,DOLK,HMGCL,TSR2,B3GALT6,RREB1,NADK2, PRKAR1B,MYO7A,CPLANE1,BCAP31,SATB2,KAT8,CNBP,BRF1,WWOX,ACTG1,COX6B1,TAF15,UQCC2,RTTN,CTCF,ATXN3,DYNC2H1

	HP:0002119	Ventriculomegaly	0.48	0.0044	OTUD5,RNASEH2A,KRT5,BRIP1,SPG11,RAB11A,KMT2C,GRM1,MYORG, ERCC4,MAP11,PDE2A,XPR1,NADK2,KAT8,BRF1,ACTG1,COX6B1,RTTN,CTCF, ATXN3,DYNC2H1
	HP:0012373	Abnormal eye physiology	0.27	0.0045	COX15,OTUD5,RNASEH2A,KRT5,SNAP25,APC,BRIP1,SPG11,RAB11A, KMT2C,TUBB4A,HPS5,NDUFS6,MARCHF6,NDUFB9,GRM1,BBS5,MYORG, CA4,SDC2,ERCC4,ARR3,PIBF1,NR2F1,COX14,SMAD3,TUBB4B,KLHL7,TRAF3IP2,F BXL3,RPS26,MT-CO1,MT-ND4,COQ8A,FMR1, TSR2,B3GALT6,RREB1,NADK2, MYO7A,CPLANE1,BCAP31,SATB2,KAT8,ADAM9,BRF1,WWOX,ACTG1,COX6B1,R TTN,CTCF,ATXN3,GET1
	HP:0100543	Cognitive impairment	0.3	0.0045	COX15,OTUD5,RNASEH2A,NPRL2,SNAP25,APC,BRIP1,SPG11,RAB11A,KMT2C, TUBB4A,MARCHF6,GRM1,BBS5,MYORG,CA4,ERCC4,PIBF1,MAP11,NR2F1, COX14,PDE2A,KLHL7,MT-CO1,MT-ND4,COQ8A,XPR1,DPM3,FMR1,DOLK, HMGCL,B3GALT6,RREB1,PRKAR1B, MYO7A,CPLANE1,BCAP31,SATB2,BRF1, WWOX,ACTG1,COX6B1,RTTN,CTCF,ATXN3
	HP:0001250	Seizure	0.32	0.0046	COX15,OTUD5,RNASEH2A,NPRL2,SNAP25,SPG11,RAB11A,KMT2C,TUBB4A, NDUFS6,MARCHF6,GRM1,MYORG,ERCC4,PIBF1,GEMIN4,NR2F1,COX14, COQ6,PDE2A,TRAF3IP2,MT-CO1,MT-ND4,COQ8A,XPR1,FMR1,DOLK,HMGCL, B3GALT6,RREB1,NADK2,CPLANE1, BCAP31,SATB2,KAT8,WWOX,ACTG1, COX6B1,UQCC2,RTTN
	HP:0012638	Abnormal nervous system physiology	0.23	0.0046	COX15,OTUD5,RNASEH2A,NPRL2,KRT5,RARA,SNAP25,APC,BRIP1,LPIN2,SPG11, RAB11A,KMT2C,TUBB4A,NDUFS6,MARCHF6,NDUFB9,GRM1,BBS5,MYORG, CA4,ERCC4,PIBF1,GEMIN4,MAP11,NR2F1,COX14,SMAD3,COQ6,PDE2A,KLHL7, TRAF3IP2,FBXL3,RPS26,MT-CO1,MT-ND4,COQ8A,XPR1,DPM3,PLEKHA1,FMR1, DOLK,HMGCL,TSR2,B3GALT6,RREB1, NADK2,PRKAR1B,MYO7A,CPLANE1,

					BCAP31,MAPKBP1,SATB2,KAT8,ADAM9,TRERF1,BRF1,WWOX,ACTG1,COX6B1,UQCC2,RTTN,CTCF,ATXN3
	HP:0012639	Abnormal nervous system morphology	0.26	0.0046	COX15,OTUD5,RNASEH2A,NPRL2,KRT5,SNAP25,APC,BRIP1,SPG11,RAB11A, KMT2C,TUBB4A,PITX1,NDUFS6,NDUFB9,GRM1,MYORG,ERCC4,PIBF1,GEMIN4, MAP11,NR2F1,COX14,SMAD3,PDE2A,FBXL3,RPS26,MT-CO1,MT-ND4, COQ8A,XPR1,DPM3,FMR1,DOLK,HMGCL, TSR2,B3GALT6,RREB1,NADK2, PRKAR1B,MYO7A,CPLANE1,BCAP31,SATB2,KAT8,CNBP,CITED2,BRF1,WWOX, ACTG1,COX6B1,RTTN,CTCF,ATXN3,DYNC2H1
	HP:0000707	Abnormality of the nervous system	0.22	0.0063	COX15,OTUD5,RNASEH2A,NPRL2,KRT5,RARA,SNAP25,APC,BRIP1,LPIN2, SPG11,RAB11A,KMT2C,TUBB4A,PITX1,NDUFS6,MARCHF6,NDUFB9,GRM1, BBS5,MYORG,CA4,ERCC4,PIBF1,GEMIN4,MAP11,NR2F1,COX14,SMAD3,COQ6, PDE2A,KLHL7,TRAF3IP2,FBXL3,RPS26,MT-CO1,MT-ND4,COQ8A,XPR1, DPM3,PLEKHA1,FMR1,DOLK,HMGCL,TSR2,B3GALT6,RREB1,NADK2,PRKAR1B, MYO7A,CPLANE1,BCAP31,MAPKBP1,SATB2,KAT8,CNBP,ADAM9,TRERF1,CITED2,BRF1,WWOX,ACTG1,COX6B1,UQCC2,RTTN,CTCF,ATXN3,DYNC2H1
	HP:0000486	Strabismus	0.4	0.0069	COX15,OTUD5,SNAP25,APC,BRIP1,SPG11,HPS5,NDUFS6,NDUFB9,GRM1,ERCC4, PIBF1,NR2F1,SMAD3,FBXL3,RPS26,COQ8A,FMR1,TSR2,RREB1,CPLANE1,BCAP31, SATB2,KAT8,BRF1,CTCF,GET1
	HP:0000252	Microcephaly	0.37	0.0084	COX15,OTUD5,RNASEH2A,BRIP1,SPG11,RAB11A,KMT2C,TUBB4A,NDUFS6, NDUFB9,MYORG,ERCC4,GEMIN4,MAP11,PDE2A,FBXL3,RPS26,XPR1,DOLK, HMGCL,TSR2,RREB1,NADK2,BCAP31,SATB2,BRF1,WWOX,ACTG1,RTTN,CTCF

	HP:0025454	Abnormal CSF metabolite concentration	0.82	0.0084	COX15,RNASEH2A,NDUFS6,NDUFB9,COX14,MT-CO1,MT-ND4,COQ8A,COX6B1
	HP:0040195	Decreased head circumference	0.37	0.0089	COX15,OTUD5,RNASEH2A,BRIP1,SPG11,RAB11A,KMT2C,TUBB4A,NDUFS6, NDUFB9,MYORG,ERCC4,GEMIN4,MAP11,PDE2A,FBXL3,RPS26,XPR1,DOLK, HMGCL,TSR2,RREB1,NADK2,BCAP31,SATB2,BRF1,WWOX,ACTG1,RTTN,CTCF
	HP:0001507	Growth abnormality	0.27	0.0091	COX15,OTUD5,RNASEH2A,KRT5,RARA,APC,BRIP1,LPIN2,SPG11,RAB11A, KMT2C,TUBB4A,PITX1,NDUFS6,NDUFB9,GRM1,BBS5,MYORG,CA4,ERCC4, MAP11,NR2F1,COX14,SMAD3,KLHL7,FBXL3,RPS26,MT-CO1,MT-ND4,XPR1, DOLK,HMGCL,TSR2,B3GALT6,RREB1,NADK2, CPLANE1,BCAP31,MAPKBP1, SATB2,BRF1,WWOX,ACTG1,COX6B1,UQCC2,RTTN,CTCF,DYNC2H1
	HP:0012337	Abnormal homeostasis	0.34	0.0103	COX15,RNASEH2A,KRT5,RARA,APC,LPIN2,SPG11,NEK8,NDUFS6,NDUFB9, CA4,ERCC4,ISCU,COX14,KLHL7,TRAF3IP2,RPS26,MT-CO1,MT-ND4,COQ8A, FMR1,DOLK,HMGCL,TSR2, NADK2,BCAP31,CNBP,CITED2,ACTG1,COX6B1, UQCC2,ATXN3,DYNC2H1
	HP:0012372	Abnormal eye morphology	0.27	0.0108	COX15,OTUD5,RNASEH2A,KRT5,APC,BRIP1,SPG11,KMT2C,TUBB4A,PITX1, HPS5,NDUFS6,NDUFB9,BBS5,MYORG,CA4,ERCC4,PIBF1,GEMIN4,NR2F1, COX14,SMAD3,TUBB4B,KLHL7,RPS26,MT-CO1,MT-ND4,XPR1,TSR2,B3GALT6, RREB1,MYO7A,CPLANE1,BCAP31,SATB2, KAT8,CNBP,ADAM9,BRF1,WWOX, ACTG1,COX6B1,RTTN,CTCF,ATXN3,DYNC2H1
	HP:0000478	Abnormality of the eye	0.23	0.0109	COX15,OTUD5,RNASEH2A,KRT5,SNAP25,APC,BRIP1,SPG11,RAB11A,KMT2C, TUBB4A,PITX1,HPS5,NDUFS6,MARCHF6,NDUFB9,GRM1,BBS5,MYORG,CA4, SDC2,ERCC4,ARR3,PIBF1,GEMIN4,NR2F1,COX14,SMAD3,TUBB4B,KLHL7,

					TRAF3IP2,FBXL3,RPS26,MT-CO1,MT-ND4,COQ8A,XPR1,FMR1,TSR2,B3GALT6, RREB1,NADK2,MYO7A,CPLANE1,BCAP31,SATB2,KAT8,CNBP,ADAM9,BRF1,WW OX,ACTG1,COX6B1,RTTN,CTCF,ATXN3,GET1,DYNC2H1
	HP:0001510	Growth delay	0.31	0.0109	OTUD5,RNASEH2A,KRT5,BRIP1,LPIN2,RAB11A,KMT2C,TUBB4A,PITX1,NDUFS6, NDUFB9,GRM1,BBS5,MYORG,ERCC4,MAP11,NR2F1,FBXL3,RPS26,MT-CO1,MT-ND4,XPR1,TSR2, B3GALT6,RREB1,CPLANE1,BCAP31,MAPKBP1,SATB2,BRF1, WWOX,ACTG1,COX6B1,UQCC2,RTTN,CTCF,DYNC2H1
	HP:0002490	Increased CSF lactate	0.85	0.0109	COX15,NDUFS6,NDUFB9,COX14,MT-CO1,MT-ND4,COQ8A,COX6B1
	HP:0010993	Abnormal cerebral subcortex morphology	0.37	0.0109	COX15,OTUD5,RNASEH2A,APC,SPG11,RAB11A,KMT2C,PITX1,ERCC4,PIBF1, MAP11,NR2F1,MT-CO1,MT-ND4,COQ8A,XPR1,HMGCL,NADK2,CPLANE1, BCAP31,SATB2,KAT8,BRF1, WWOX,ACTG1,RTTN,ATXN3,DYNC2H1
	HP:0000079	Abnormality of the urinary system	0.3	0.0113	OTUD5,RARA,APC,BRIP1,LPIN2,SPG11,KMT2C,NEK8,NDUFS6,NDUFB9,BBS5,ER CC4,ISCU,GEMIN4,MAP11,COX14,SMAD3,COQ6,TRAF3IP2,RPS26,MT-CO1,MT-ND4,FMR1,HMGCL, TSR2,B3GALT6,RREB1,NADK2,CPLANE1,MAPKBP1,WWC1, BRF1,WWOX,ACTG1,COX6B1,UQCC2,RTTN,ATXN3,DYNC2H1
	HP:0001249	Intellectual disability	0.3	0.0113	COX15,OTUD5,RNASEH2A,NPRL2,SNAP25,APC,BRIP1,SPG11,RAB11A,KMT2C, TUBB4A,MARCHF6,GRM1,BBS5,CA4,ERCC4,PIBF1,MAP11,NR2F1,COX14,PDE2A, KLHL7,MT-CO1, COQ8A,DPM3,FMR1,HMGCL,B3GALT6,RREB1,MYO7A, CPLANE1,BCAP31,SATB2,BRF1,WWOX,ACTG1,COX6B1,RTTN,CTCF
	HP:0002354	Memory impairment	0.84	0.0113	SPG11,MYORG,MT-CO1,MT-ND4,XPR1,FMR1,PRKAR1B,ATXN3

	HP:0003800	Muscle abnormality related to mitochondrial dysfunction	0.92	0.0113	NDUFS6,NDUFB9,ISCU,COX14,MT-CO1,MT-ND4,COX6B1
	HP:0031704	Abnormal ear physiology	0.32	0.0113	COX15,OTUD5,RARA,SNAP25,APC,BRIP1,KMT2C,TUBB4A,NDUFS6,NDUFB9,GRM1,BBS5,CA4,ERCC4,NR2F1,COX14,COQ6,KLHL7,RPS26,MT-CO1,MT-ND4,COQ8A,FMR1,DOLK,B3GALT6,RREB1,MYO7A,CPLANE1,BCAP31,ACTG1,COX6B1,RTTN,MYO15A,ATXN3
	HP:0000508	Ptosis	0.44	0.0121	COX15,RNASEH2A,SNAP25,APC,BRIP1,NDUFS6,NDUFB9,GRM1,ERCC4,PIBF1,COX14,FBXL3,RPS26,MT-CO1,TSR2,RREB1,CPLANE1,WWOX,ACTG1,COX6B1,ATXN3
	HP:0012758	Neurodevelopmental delay	0.28	0.0124	COX15,OTUD5,RNASEH2A,KRT5,SNAP25,BRIP1,SPG11,KMT2C,TUBB4A,NDUFS6,NDUFB9,GRM1,ERCC4,PIBF1,GEMIN4,MAP11,NR2F1,COX14,PDE2A,FBXL3,RPS26,MT-CO1,MT-ND4,COQ8A,FMR1,HMGCL,TSR2,B3GALT6,RREB1,NADK2,MYO7A,CPLANE1,BCAP31,SATB2,KAT8,BRF1,WWOX,ACTG1,COX6B1,UQCC2,RTTN,CTCF,ATXN3
	HP:0000119	Abnormality of the genitourinary system	0.26	0.0129	OTUD5,RNASEH2A,RARA,APC,BRIP1,LPIN2,SPG11,KMT2C,HPS5,NEK8,NDUFS6,NDUFB9,BBS5,CA4,ERCC4,ISCU,GEMIN4,MAP11,COX14,SMAD3,COQ6,KLHL7,TRAF3IP2,RPS26,MT-CO1,MT-ND4,FMR1,HMGCL,TSR2,B3GALT6,RREB1,NADK2,MYO7A,CPLANE1,MAPKBP1, SATB2,CNBP,WWC1,BRF1,WWOX,ACTG1,COX6B1,UQCC2,RTTN,CTCF,ATXN3,DYNC2H1

	HP:0001263	Global developmental delay	0.3	0.0129	COX15,OTUD5,RNASEH2A,KRT5,BRIP1,SPG11,KMT2C,NDUFS6,NDUFB9, GRM1,ERCC4,PIBF1,GEMIN4,MAP11,NR2F1,COX14,PDE2A,FBXL3,MT-CO1, MT-ND4,COQ8A,HMGCL,B3GALT6,RREB1,NADK2,MYO7A,CPLANE1,BCAP31,SATB, KAT8,BRF1,WWOX,ACTG1,COX6B1,UQCC2,RTTN,CTCF
	HP:0001639	Hypertrophic cardiomyopathy	0.62	0.0129	COX15,RNASEH2A,BRIP1,NEK8,NDUFS6,NDUFB9,ERCC4,COX14,MT-CO1,MT-ND4,MYO7A,COX6B1
	HP:0012211	Abnormal renal physiology	0.46	0.0144	RARA,BRIP1,LPIN2,NEK8,NDUFS6,NDUFB9,BBS5,ERCC4,COX14,COQ6,TRAF3IP2, MT-CO1,MT-ND4,NADK2,MAPKBP1,WWOX,COX6B1,UQCC2,DYNC2H1
	HP:0000240	Abnormality of skull size	0.32	0.0149	COX15,OTUD5,RNASEH2A,BRIP1,SPG11,RAB11A,KMT2C,TUBB4A,PITX1, NDUFS6,NDUFB9,MYORG,ERCC4,GEMIN4,MAP11,PDE2A,FBXL3,RPS26, XPR1,FMR1,DOLK,HMGCL,TSR2,RREB1,NADK2,BCAP31,SATB2,BRF1,WWOX,ACTG1,RTTN,CTCF,DYNC2H1
	HP:0004325	Decreased body weight	0.33	0.0149	COX15,OTUD5,KRT5,RARA,APC,BRIP1,LPIN2,SPG11,KMT2C,TUBB4A,NDUFS6,NDUFB9,ERCC4,COX14,RPS26,MT-CO1,MT-ND4,DOLK,HMGCL,TSR2,B3GALT6, RREB1,NADK2, CPLANE1,BCAP31,SATB2,BRF1,WWOX,ACTG1,COX6B1, RTTN,CTCF
	HP:0012240	Increased intramyocellular lipid droplets	1.13	0.0149	ISCU,COX14,MT-CO1,COQ8A,COX6B1
	HP:0000639	Nystagmus	0.37	0.0154	COX15,OTUD5,RNASEH2A,KRT5,SNAP25,APC,BRIP1,SPG11,TUBB4A,HPS5, NDUFS6,NDUFB9,GRM1,BBS5,CA4,ERCC4,PIBF1,NR2F1,KLHL7,MT-ND4, FMR1,NADK2,MYO7A,CPLANE1,WWOX,ATXN3

	HP:0004328	Abnormal anterior eye segment morphology	0.35	0.0154	OTUD5,RNASEH2A,KRT5,APC,BRIP1,KMT2C,HPS5,MYORG,CA4,ERCC4,PIBF1,GEMIN4,NR2F1,SMAD3,KLHL7,RPS26,MT-CO1,MT-ND4,XPR1,TSR2,B3GALT6,RREB1,MYO7A,CPLANE1, CNBP,BRF1,ACTG1,CTCF,DYNC2H1
	HP:0003546	Exercise intolerance	0.8	0.0156	ISCU,ENO3,COX14,MT-CO1,MT-ND4,COQ8A,CITED2,COX6B1
	HP:0012547	Abnormal involuntary eye movements	0.37	0.0164	COX15,OTUD5,RNASEH2A,KRT5,SNAP25,APC,BRIP1,SPG11,TUBB4A,HPS5,NDUFS6,NDUFB9,GRM1,BBS5,CA4,ERCC4,PIBF1,NR2F1,KLHL7,MT-ND4,FMR1,NADK2,MYO7A,CPLANE1,WWOX,ATXN3
	HP:0002793	Abnormal pattern of respiration	0.43	0.0166	COX15,RARA,SNAP25,APC,TUBB4A,NDUFS6,ISCU,PIBF1,COX14,SMAD3,KLHL7,RPS26,MT-CO1,MT-ND4,HMGCL,B3GALT6,NADK2,CPLANE1,CITED2,COX6B1
	HP:0000924	Abnormality of the skeletal system	0.23	0.0174	COX15,OTUD5,RNASEH2A,KRT5,RARA,SNAP25,APC,BRIP1,LPIN2,SPG11,RAB11A,KMT2C,TUBB4A,PITX1,NEK8,NDUFS6,NDUFB9,BBS5,MYORG,ERCC4,PIBF1,GEMIN4,MAP11,NR2F1,SMAD3,PDE2A,KLHL7,TRAF3IP2,FBXL3,RPS26,MT-CO1,MT-ND4,COQ8A,XPR1,FMR1, DOLK,HMGCL,TSR2,B3GALT6,RREB1,NADK2,MYO7A,CPLANE1,BCAP31,SATB2,KAT8,BRF1,WWOX,ACTG1,TAF15,UQCC2,RTTN,CTCF,DYNC2H1
	HP:0001626	Abnormality of the cardiovascular system	0.24	0.0174	COX15,OTUD5,RNASEH2A,KRT5,RARA,APC,BRIP1,LPIN2,SPG11,KMT2C,HPS5,NEK8,NDUFS6,NDUFB9,BBS5,MYORG,CA4,ERCC4,ISCU,PIBF1,COX14,SMAD3,KLHL7,TRAF3IP2,RPS26,MT-CO1,MT-ND4,XPR1,DPM3,FMR1,DOLK,HMGCL,TSR2,B3GALT6,RREB1,PRKAR1B,MYO7A, CPLANE1,MAPKBP1,KAT8,CNBP,CITED2,BRF1,WWOX,ACTG1,COX6B1,RTTN,CTCF,ATXN3,DYNC2H1

	HP:0000598	Abnormality of the ear	0.27	0.0177	COX15,OTUD5,RNASEH2A,RARA,SNAP25,APC,BRIP1,KMT2C,TUBB4A,PITX1, NDUFS6,NDUFB9,GRM1,BBS5,CA4,ERCC4,PIBF1,NR2F1,COX14,COQ6,KLHL7,RPS 26,MT-CO1,MT-ND4, COQ8A,FMR1,DOLK,TSR2,B3GALT6,RREB1,MYO7A, CPLANE1,BCAP31,SATB2,KAT8,BRF1,ACTG1,COX6B1,RTTN,CTCF, MYO15A,ATXN3
	HP:0000124	Renal tubular dysfunction	0.77	0.0196	NDUFS6,NDUFB9,COX14,MT-CO1,MT-ND4,NADK2,COX6B1,UQCC2
	HP:0004970	Ascending tubular aorta aneurysm	1.3	0.0196	SMAD3,FMR1,B3GALT6,BRF1
	HP:0025142	Constitutional symptom	0.37	0.0198	NPRL2,KRT5,RARA,APC,LPIN2,SPG11,GRM1,ZCCHC4,ERCC4,ISCU,ENO3,COX14 ,SMAD3,MT-CO1,MT- ND4,COQ8A,DPM3,FMR1,DOLK,HMGCL,RREB1,CNBP,CITED2,COX6B1,ATXN3
	EFO:0004340	Body mass index	0.32	0.0204	MGA,CCDC92,APC,USP37,LPIN2,RAB11A,ZNF280D,LIMD1,MARCHF6,ZC3H12C, POLR2M,ZCCHC4,XXYLT1,SPNS1,ATAD5,SP1,RPS26,PJA2,RLF,KDM2B,RREB1,SP TLC3,DTNB,METTL15,MAPKBP1,KAT8,KLC1,WWC1,MEX3A,WWOX,DYNC2H1
	HP:0001251	Ataxia	0.38	0.0204	COX15,SNAP25,APC,SPG11,TUBB4A,NDUFS6,NDUFB9,GRM1,MYORG,ERCC4,PIB F1,COX14,PDE2A,MT-CO1,MT-ND4,COQ8A,FMR1,HMGCL, NADK2,MYO7A,CPLANE1,WWOX, COX6B1,ATXN3
	HP:0002094	Dyspnea	0.53	0.0204	RARA,SNAP25,APC,TUBB4A,ISCU,COX14,SMAD3,KLHL7,RPS26,MT-CO1,MT- ND4,B3GALT6,CITED2,COX6B1

	HP:0034370	Abnormal muscle tissue metabolite concentration	0.94	0.0204	ISCU,ENO3,COX14,MT-CO1,COQ8A,COX6B1
	HP:0001627	Abnormal heart morphology	0.32	0.0222	COX15,OTUD5,RNASEH2A,BRIP1,KMT2C,NEK8,NDUFS6,NDUFB9,ERCC4,PIBF1, COX14,SMAD3,TRAF3IP2,RPS26,MT-CO1,MT-ND4,DPM3,FMR1,DOLK,HMGCL, TSR2,B3GALT6, RREB1,MYO7A,CPLANE1,KAT8,CITED2,BRF1,COX6B1,CTCF
	HP:0000364	Hearing abnormality	0.31	0.0223	COX15,OTUD5,SNAP25,APC,BRIP1,KMT2C,TUBB4A,NDUFS6,NDUFB9,GRM1,BBS 5,CA4,ERCC4,NR2F1,COX14,COQ6,KLHL7,RPS26,MT-CO1,MT-ND4,COQ8A,FMR1, DOLK,B3GALT6,RREB1,MYO7A,CPLANE1,BCAP31,ACTG1,COX6B1, RTTN,MYO15A
	HP:0002086	Abnormality of the respiratory system	0.28	0.0224	COX15,OTUD5,RNASEH2A,KRT5,RARA,SNAP25,APC,BRIP1,LPIN2,SPG11, KMT2C,TUBB4A,NEK8,NDUFS6,NDUFB9,ERCC4,ISCU,PIBF1,COX14,SMAD3,KLH L7,TRAF3IP2,RPS26,MT-CO1,MT-ND4,FMR1,HMGCL,B3GALT6,RREB1,NADK2, CPLANE1,SATB2,CITED2,BRF1, COX6B1,CTCF,DYNC2H1
	HP:0002795	Abnormal respiratory system physiology	0.33	0.0225	COX15,RNASEH2A,RARA,SNAP25,APC,LPIN2,SPG11,TUBB4A,NEK8,NDUFS6, NDUFB9,ISCU,PIBF1,COX14,SMAD3,KLHL7,TRAF3IP2,RPS26,MT-CO1,MT-ND4, HMGCL,B3GALT6,RREB1, NADK2,CPLANE1,CITED2,COX6B1,CTCF,DYNC2H1
	HP:0004323	Abnormality of body weight	0.29	0.0225	COX15,OTUD5,KRT5,RARA,APC,BRIP1,LPIN2,SPG11,KMT2C,TUBB4A,NDUFS6,N DUFB9,BBS5,CA4,ERCC4,COX14,KLHL7,RPS26,MT-CO1,MT-ND4,DOLK, HMGCL,TSR2,B3GALT6,RREB1, NADK2,CPLANE1,BCAP31,SATB2,BRF1, WWOX,ACTG1,COX6B1,RTTN,CTCF

	HP:0011804	Abnormal muscle physiology	0.25	0.0225	COX15,OTUD5,RNASEH2A,RARA,SNAP25,SPG11,RAB11A,KMT2C,TUBB4A, NDUFS6,MARCHF6,NDUFB9,GRM1,ERCC4,ISCU,PIBF1,GEMIN4,ENO3, NR2F1,COX14,PDE2A,MT-CO1,MT-ND4,COQ8A,DPM3,FMR1,DOLK,HMGCL, B3GALT6,RREB1,NADK2,PRKAR1B,MYO7A,CPLANE1,SATB2,CNBP,CITED2,BRF1 ,WWOX,COX6B1,UQCC2,RTTN,CTCF,ATXN3
	HP:0001882	Leukopenia	0.82	0.0244	OTUD5,RARA,BRIP1,ERCC4,RPS26,HMGCL,TSR2
	HP:0004360	Abnormality of acid-base homeostasis	0.51	0.0244	COX15,APC,NEK8,NDUFS6,NDUFB9,ISCU,COX14,MT-CO1,MT-ND4,COQ8A,HMGCL,NADK2,COX6B1,UQCC2
	HP:0033725	Thin corpus callosum	0.4	0.0244	OTUD5,RNASEH2A,APC,SPG11,RAB11A,KMT2C,PITX1,PIBF1,MAP11,NR2F1, MT-CO1,MT-ND4,NADK2,CPLANE1, BCAP31,KAT8,BRF1,WWOX,ACTG1, RTTN,DYNC2H1
	HP:0000365	Hearing impairment	0.31	0.0266	COX15,OTUD5,SNAP25,APC,BRIP1,KMT2C,TUBB4A,NDUFS6,NDUFB9,BBS5,CA4, ERCC4,NR2F1,COX14,COQ6,KLHL7,RPS26,MT-CO1,MT-ND4,COQ8A,FMR1, DOLK,B3GALT6,RREB1, MYO7A,CPLANE1,BCAP31,ACTG1,COX6B1, RTTN,MYO15A
	HP:0011805	Abnormal skeletal muscle morphology	0.28	0.0275	OTUD5,RNASEH2A,KRT5,SNAP25,LPIN2,SPG11,TUBB4A,PITX1, NDUFS6,NDUFB9,BBS5,ERCC4,ISCU,GEMIN4,ENO3,COX14,SMAD3,KLHL7, RPS26,MT-CO1,MT-ND4,COQ8A,DPM3, DOLK,TSR2,B3GALT6,MYO7A,SATB2, CNBP,BRF1,WWOX,COX6B1,RTTN,CTCF,ATXN3

	HP:0002167	Neurological speech impairment	0.3	0.0278	COX15,RNASEH2A,NPRL2,SNAP25,SPG11,RAB11A,KMT2C,TUBB4A,GRM1, BBS5,MYORG,ERCC4,GEMIN4,NR2F1,PDE2A,FBXL3,MT-CO1,MT-ND4,XPR1, FMR1,HMGCL,B3GALT6,RREB1,SATB2,KAT8,BRF1,WWOX,ACTG1,UQCC2,RTTN, CTCF,ATXN3
	HP:0011873	Abnormal platelet count	0.53	0.0278	OTUD5,RNASEH2A,RARA,APC,BRIP1,HPS5,MYORG,ERCC4,RPS26, XPR1,HMGCL,TSR2,RREB1
	HP:0000234	Abnormality of the head	0.21	0.0281	COX15,OTUD5,RNASEH2A,KRT5,RARA,SNAP25,APC,BRIP1,SPG11,RAB11A, KMT2C,TUBB4A,PITX1,HPS5,NDUFS6,NDUFB9,BBS5,MYORG,CA4,ERCC4, PIBF1,GEMIN4,MAP11,NR2F1,COX14,SMAD3,PDE2A,KLHL7,TRAF3IP2, FBXL3,RPS26,MT-CO1,MT-ND4,XPR1,MCL1,FMR1, DOLK,HMGCL, TSR2,B3GALT6,RREB1,NADK2,MYO7A,CPLANE1,BCAP31,SATB2,KAT8,BRF1,WW OX,ACTG1,COX6B1,UQCC2,RTTN,CTCF,DYNC2H1
	HP:0012795	Abnormal optic disc morphology	0.4	0.0286	COX15,APC,TUBB4A,NDUFS6,NDUFB9,CA4,ERCC4,GEMIN4,NR2F1,COX14, KLHL7,MT-CO1,MT-ND4,B3GALT6,RREB1,BCAP31,WWOX,ACTG1,COX6B1,RTTN
	HP:0003326	Myalgia	0.73	0.0292	APC,LPIN2,ENO3,FMR1,DOLK,RREB1,CNBP,COX6B1
	HP:0000726	Dementia	0.67	0.0302	SPG11,TUBB4A,ERCC4,MT-CO1,MT-ND4,XPR1,FMR1,PRKAR1B,ATXN3
	HP:0001941	Acidosis	0.53	0.0302	COX15,NEK8,NDUFS6,NDUFB9,ISCU,COX14,MT-CO1,MT-ND4,COQ8A, HMGCL,NADK2, COX6B1,UQCC2
	HP:0002134	Abnormal basal ganglia morphology	0.62	0.0302	COX15,RNASEH2A,SPG11,ERCC4,MT-CO1,MT-ND4, COQ8A,XPR1,NADK2,ATXN3

	HP:0003128	Lactic acidosis	0.67	0.0302	NDUFS6,NDUFB9,ISCU,COX14,MT-CO1,MT-ND4,COQ8A,NADK2,COX6B1
	HP:0003287	Abnormality of mitochondrial metabolism	0.72	0.0312	COX15,NDUFS6,NDUFB9,ISCU,MT-CO1,MT-ND4,HMGCL,NADK2
	HP:0007703	Abnormality of retinal pigmentation	0.52	0.0312	COX15,APC,BBS5,CA4,ERCC4,COX14,KLHL7,MT-CO1,MT-ND4,MYO7A, ADAM9,COX6B1, DYNC2H1
	HP:0000587	Abnormality of the optic nerve	0.38	0.0324	COX15,OTUD5,APC,TUBB4A,NDUFS6,NDUFB9,CA4,ERCC4,GEMIN4,NR2F1, COX14,KLHL7,MT-CO1,MT-ND4,B3GALT6,RREB1,BCAP31,WWOX, ACTG1,COX6B1,RTTN
	HP:0001098	Abnormal fundus morphology	0.32	0.0351	COX15,OTUD5,APC,SPG11,TUBB4A,HPS5,NDUFS6,NDUFB9,BBS5,CA4,ERCC4,GE MIN4,NR2F1,COX14,TUBB4B,KLHL7,MT-CO1,MT-ND4,B3GALT6,RREB1, MYO7A,BCAP31,ADAM9,WWOX,ACTG1,COX6B1,RTTN,DYNC2H1
	HP:0001871	Abnormality of blood and blood-forming tissues	0.31	0.0399	COX15,OTUD5,RNASEH2A,KRT5,RARA,APC,BRIP1,LPIN2,HPS5,MYORG, ERCC4,ISCU,COX14,SMAD3,RPS26,MT-CO1,MT-ND4,XPR1,DOLK,HMGCL, TSR2,B3GALT6,RREB1,MAPKBP1, CNBP,KANSL2,CITED2,COX6B1,CTCF
	HP:0000118	Phenotypic abnormality	0.15	0.0408	COX15,OTUD5,RIN3,RNASEH2A,NPRL2,KRT5,RARA,SNAP25,APC,USP37, BRIP1,LPIN2,SPG11,RAB11A,KMT2C,TUBB4A,PITX1,HPS5,NEK8,NDUFS6,MARC HF6,NDUFB9,GRM1,BBS5,MYORG,CA4,ZCCHC4,SDC2,ERCC4,ISCU,ARR3, PIBF1,GEMIN4,ENO3,MAP11,NR2F1,COX14,SMAD3,COQ6,PDE2A, TUBB4B,KLHL7,TRAF3IP2,FBXL3,RPS26,MT-CO1,MT-ND4,COQ8A, XPR1,DPM3,PLEKHA1,MCL1,FMR1,DOLK,HMGCL,TSR2,B3GALT6,RREB1,NADK 2,PRKAR1B,MYO7A,CPLANE1,BCAP31,MAPKBP1,SATB2,KAT8,CNBP,KANSL2,A

					DAM9,WWC1,CDC27,TRERF1,CITED2,BRF1,WWOX,ACTG1,COX6B1,TAF15, UQCC2,RTTN,CTCF,MYO15A,ATXN3,GET1,DYNC2H1
	HP:0007370	Aplasia/Hypoplasia of the corpus callosum	0.38	0.0408	OTUD5,RNASEH2A,APC,SPG11,RAB11A,KMT2C,PITX1,PIBF1,MAP11,NR2F1,MT-CO1, MT-ND4,NADK2,CPLANE1,KAT8,BRF1,WWOX,ACTG1,RTTN,DYNC2H1
	HP:0000504	Abnormality of vision	0.31	0.0425	KRT5,SNAP25,APC,BRIP1,SPG11,RAB11A,TUBB4A,HPS5,NDUFS6,MARCHF6, NDUFB9,BBS5,CA4,ERCC4,NR2F1,TUBB4B,KLHL7,TRAF3IP2,MT-CO1,MT-ND4, NADK2,MYO7A,SATB2,ADAM9,WWOX,COX6B1,RTTN,ATXN3
	HP:0001268	Mental deterioration	0.53	0.0436	SPG11,TUBB4A,MYORG,ERCC4,MT-CO1,MT-ND4,XPR1,FMR1,PRKAR1B,WWOX,COX6B1 ,ATXN3
	HP:0025031	Abnormality of the digestive system	0.22	0.0437	COX15,OTUD5,RNASEH2A,KRT5,RARA,SNAP25,APC,BRIP1,LPIN2, SPG11,RAB11A,KMT2C,TUBB4A,NEK8,NDUFS6,NDUFB9,GRM1,BBS5,MYORG, ERCC4,PIBF1,GEMIN4,COX14,SMAD3,KLHL7,TRAF3IP2,RPS26,MT-CO1,MT-ND4, XPR1,FMR1,DOLK,HMGCL,TSR2,RREB1, NADK2,CPLANE1,SATB2,KAT8, BRF1,WWOX,ACTG1,COX6B1,RTTN,CTCF,ATXN3,DYNC2H1
	HP:0004370	Abnormality of temperature regulation	0.5	0.045	RNASEH2A,KRT5,RARA,APC,LPIN2,ERCC4,KLHL7,TRAF3IP2,MT-CO1,MT-ND4, HMGCL, BCAP31,ATXN3
	HP:0011277	Abnormality of the urinary system physiology	0.34	0.0451	RARA,APC,BRIP1,LPIN2,NEK8,NDUFS6,NDUFB9,BBS5,ERCC4,ISCU,GEMIN4, COX14,COQ6,TRAF3IP2,MT-CO1,MT-ND4,HMGCL,NADK2,MAPKBP1,WWC1, WWOX,COX6B1,UQCC2, DYNC2H1

	HP:0001873	Thrombocytopenia	0.52	0.0452	OTUD5,RNASEH2A,RARA,APC,BRIP1,HPS5,MYORG,ERCC4,RPS26,XPR1,TSR2,RREB1
	HP:0001903	Anemia	0.43	0.0452	COX15,KRT5,RARA,APC,BRIP1,LPIN2,ERCC4,ISCU,COX14,RPS26,MT-CO1,MT-ND4,HMGCL,TSR2,MAPKBP1,COX6B1
	HP:0008678	Renal hypoplasia/aplasia	0.55	0.0483	APC,BRIP1,ERCC4,GEMIN4,MAP11,RPS26,TSR2,RREB1,CPLANE1,RTTN,DYNC2H1
	HP:0012447	Abnormal myelination	0.43	0.0483	COX15,RNASEH2A,SPG11,TUBB4A,NDUFS6,NDUFB9,ERCC4,GEMIN4,NR2F1,MT-CO1,MT-ND4,NADK2,BCAP31,SATB2,WWOX,COX6B1
	HP:0410042	Abnormal liver morphology	0.37	0.0483	COX15,OTUD5,RNASEH2A,APC,LPIN2,NEK8,NDUFS6,NDUFB9,BBS5,MYORG,ERCC4,COX14,TRAF3IP2,MT-CO1,MT-ND4,XPR1,DOLK,HMGCL,COX6B1,DYNC2H1
	HP:0001638	Cardiomyopathy	0.45	0.0487	COX15,RNASEH2A,BRIP1,NEK8,NDUFS6,NDUFB9,ERCC4,COX14,MT-CO1, MT-ND4,DPM3,DOLK,HMGCL,MYO7A,COX6B1
	HP:0002098	Respiratory distress	0.62	0.05	SNAP25,TUBB4A,COX14,KLHL7,RPS26,MT-CO1,MT-ND4,B3GALT6,COX6B1
	HP:0011443	Abnormality of coordination	0.33	0.05	COX15,SNAP25,APC,SPG11,TUBB4A,NDUFS6,NDUFB9,GRM1,MYORG,ERCC4,PIBF1,COX14,PDE2A,MT-CO1,MT-ND4,COQ8A,FMR1,HMGCL,NADK2,MYO7A,CPLANE1,WWOX, COX6B1,ATXN3
	BTO: 0000042	Animal	0.08	9.60E-08	COX15,OTUD5,RIN3,CDIPT,MGA,RNASEH2A,RAB3D,RPS12,NPRL2,MED28,CCDC92,KALRN,SGPP1,EAPP,KRT5,RARA,COQ3,BRD8,SNAP25,APC,AKIRIN2,USP37,HUS1,FAM8A1,BUD13,CLCN3,LPIN2,ARG2,SPG11,RAB11A,MTFR1,KMT2C,

					GATB,IFT46,TUBB4A,PITX1,HPS5,TARBP2,ZNF280D,SDE2,LIMD1,NDUFS6,MARCHF6,CDK16,NDUFB9,ZC3H12C,SCLT1,PLEKHH2,GRM1,NUBP1,NIFK,FZD7,MTERF3,RNF126,MAP4K2,MICALL2,TERF2IP,CA4,PPIC,RNFT1,GON7,SDC2,RAMAC,FEM1B,TSPAN5,UTP23,XXYLT1,SPNS1,ERCC4,ISCU,ARR3,ATAD5,ZNF609,RMI1,PIBF1,MRFAP1,GEMIN4,ZKSCAN1,ENO3,MAP11,NR2F1,TMEM39A, SP1,CASP9,SPPL2C,OSBP2,SMAD3,TMEM179B,ABCC5,COQ6,PDE2A,EBAG9,TUBB4B,ENSA,LAMTOR4,KLHL7,PTMA,TRAF3IP2,RPL27A,FBXL3,RPS26,DYNLRB1, UBL5,SLC25A29,PCBP2,MT-CO1, PJA2,MT-ND4,DCLRE1A,COQ8A,FGFR1OP,UST, XPR1,SUCO,SYT11,PLEKHA1,MCL1, SH3BGRL2,FMR1,MINPP1,NCOA3,SURF6, GPR107,DOLK,RLF,SLC25A25,SLC31A1,BAK1,HMGCL,PBX2,TSR2,STK19,FBXO6,KDM2B,CTNNBIP1,ATG2A,RGP1,B3GALT6,NFX1,NHLRC3,RREB1,SDC1,NADK2,NCBP3,RNF34,VTI1A,PRC1,RPL14,EAF1,FGFRL1,SPTLC3,DTNB,METTL15,PRKAR1B,MARK2,MYO7A,CPLANE1,BCAP31,MAPKBP1,ZNF7,WDR20,CCN1,SATB2,NR2C2AP,EZH1,KAT8,CNBP,KLC1,KANSL2,DUSP7,REPIN1,UBN2,ADAM9,ANAPC13, ZNF827,SORCS2,SMUG1,MRPS30,COX18,MIB2,WWC1,CCNC,SLC25A30,TXNRD3, ZNF706,SPA17,CFL1,CDC27,RPS6KA1,TRERF1,GRAMD1A,PRR14,CITED2,MYL6, PRPF40B,BRF1,MAPKAPK5,EML5,ZNF592,CNN2,WWOX,ACTG1,CENPX,COX6B1, UQCR11,SEC11C,TAF15,APBB1,SCOC,NSA2,BTBD8,RTTN,CTCF,MYO15A,ATXN3, GET1,DYNC2H1
	BTO: 0001489	Whole body	0.1	7.12E-06	COX15,OTUD5,RIN3,CDIPT,MGA,RNASEH2A,RAB3D,RPS12,NPRL2,CCDC92, KALRN,SGPP1,EAPP,KRT5,RARA,COQ3,BRD8,SNAP25,APC,AKIRIN2,USP37,HUS1,FAM8A1,BUD13,CLCN3,LPIN2,ARG2,SPG11,RAB11A,MTFR1,KMT2C,GATB,IFT46,TUBB4A,PITX1,HPS5,ZNF280D,NDUFS6,MARCHF6,CDK16,NDUFB9,ZC3H12C, PLEKHH2,GRM1,NIFK,FZD7,MTERF3,RNF126,MAP4K2,MICALL2,TERF2IP,CA4, RNFT1,GON7,SDC2,RAMAC,FEM1B,TSPAN5,XXYLT1,SPNS1,ERCC4,ISCU,ARR3,

					ATAD5,ZNF609,RMI1,PIBF1,MRFAP1,GEMIN4,ZKSCAN1,ENO3,NR2F1, TMEM39A,SP1,CASP9,SPPL2C,OSBP2,SMAD3,ABCC5,COQ6,PDE2A,EBAG9,TUBB4B,ENSA,LAMTOR4,KLHL7,PTMA,TRAF3IP2,RPL27A,FBXL3,RPS26,DYNLRB1, UBL5,SLC25A29,PCBP2,MT-CO1,PJA2,MT-ND4,DCLRE1A,COQ8A,FGFR1OP, XPR1,SUCO,SYT11,PLEKHA1,MCL1, SH3BGRL2,FMR1,NCOA3,GPR107,SLC25A25, SLC31A1,BAK1,HMGCL,PBX2,STK19,FBXO6,KDM2B,CTNNBIP1,ATG2A,B3GALT6, NFX1,NHLRC3,SDC1,NADK2,NCBP3,RNF34,VTI1A,RPL14,EAF1,FGFRL1,SPTLC3, DTNB,PRKAR1B,MARK2,MYO7A,CPLANE1,BCAP31,MAPKBP1,ZNF7,WDR20,CCN1,SATB2,NR2C2AP,EZH1,KAT8,CNBP,KLC1,KANSL2,DUSP7,REPIN1,UBN2, ADAM9,ANAPC13,SMUG1,MRPS30,COX18,MIB2,WWC1,CCNC,TXNRD3,ZNF706, SPA17,CFL1,CDC27,RPS6KA1,TRERF1,GRAMD1A,PRR14,CITED2,MYL6,PRPF40B, BRF1,MAPKAPK5,EML5,ZNF592,CNN2,WWOX,ACTG1,CENPX,COX6B1,UQCR11, SEC11C,TAF15,APBB1,SCOC,NSA2,BTBD8,RTTN,CTCF,MYO15A,ATXN3,GET1,DYNC2H1
	BTO: 0000083	Female reproductive system	0.17	0.00049	COX15,CDIPT,MGA,RAB3D,RPS12,CCDC92,KALRN,SGPP1,EAPP,KRT5,BRD8,USP37,FAM8A1,CLCN3,ARG2,SPG11,RAB11A,MTFR1,KMT2C,IFT46,TUBB4A,HPS5,ZNF280D,MARCHF6,CDK16,PLEKHH2,NIFK,RNF126,RNFT1,SDC2,FEM1B,TSPAN5, RMI1,PIBF1,MRFAP1,GEMIN4,NR2F1,SP1,SPPL2C,OSBP2,SMAD3,EBAG9, TUBB4B,ENSA,KLHL7,PTMA,TRAF3IP2,RPL27A,FBXL3,RPS26,DYNLRB1,UBL5,PCBP2,MT-CO1,PJA2,DCLRE1A,COQ8A,FGFR1OP,XPR1, SUCO,PLEKHA1,MCL1, FMR1,HMGCL,KDM2B,ATG2A,NFX1,NHLRC3,SDC1,RPL14,EAF1,SPTLC3,PRKAR1B,MYO7A,BCAP31,MAPKBP1,WDR20,CCN1,NR2C2AP,EZH1,KAT8,CNBP,KLC1, KANSL2,UBN2,ADAM9,SMUG1,MIB2,WWC1,CCNC,ZNF706,SPA17,CFL1,CDC27, RPS6KA1,MYL6,MAPKAPK5,CNN2,WWOX,ACTG1,CENPX,TAF15,APBB1,SCOC,CTCF,ATXN3,GET1,DYNC2H1

	BTO: 0003091	Urogenital system	0.16	0.00053	COX15,OTUD5,CDIPT,MGA,RAB3D,RPS12,NPRL2,CCDC92,KALRN,SGPP1,EAPP, KRT5,COQ3,BRD8,SNAP25,USP37,HUS1,FAM8A1,CLCN3,ARG2,SPG11,RAB11A,MTFR1,KMT2C,IFT46,TUBB4A,HPS5,ZNF280D,MARCHF6,CDK16,PLEKHH2,NIFK, RNF126,RNFT1,SDC2,FEM1B,TSPAN5,RMI1,PIBF1,MRFAP1,GEMIN4,NR2F1,SP1, CASP9,SPPL2C,OSBP2,SMAD3,EBAG9,TUBB4B,ENSA,KLHL7,PTMA,TRAF3IP2,RPL27A,FBXL3,RPS26,DYNLRB1,UBL5,PCBP2,MT-CO1,PJA2,DCLRE1A,COQ8A, FGFR1OP,XPR1,SUCO,PLEKHA1,MCL1,FMR1,HMGCL,FBXO6,KDM2B,ATG2A,NFX1,NHLRC3,SDC1,RNF34,RPL14,EAF1,SPTLC3,PRKAR1B,MYO7A,BCAP31,MAPKBP1,ZNF7,WDR20,CCN1,NR2C2AP,EZH1,KAT8,CNBP,KLC1,KANSL2,UBN2,ADAM9,ANAPC13,SMUG1,MIB2,WWC1,CCNC,ZNF706,SPA17,CFL1,CDC27,RPS6KA1,MYL6,MAPKAPK5,CNN2,WWOX,ACTG1,CENPX,COX6B1,TAF15,APBB1,SCOC,NSA2,CTCF,ATXN3,GET1,DYNC2H1
	BTO: 0000282	Head	0.13	0.0287	OTUD5,CDIPT,RNASEH2A,RAB3D,CCDC92,KALRN,KRT5,RARA,BRD8, SNAP25,APC,AKIRIN2,FAM8A1,CLCN3,ARG2,SPG11,RAB11A,MTFR1,KMT2C,GATB,IFT46,TUBB4A,ZNF280D,MARCHF6,CDK16,NDUFB9,ZC3H12C,NIFK,RNF126, TERF2IP,CA4,GON7,SDC2,FEM1B,SPNS1,ISCU,ARR3,MRFAP1,ZKSCAN1,SP1,CASP9,OSBP2,SMAD3,ABCC5,COQ6,PDE2A,ENSA,KLHL7,PTMA,RPL27A,RPS26,UBL5,SLC25A29,PCBP2,MT-CO1,PJA2,SUCO,SYT11,MCL1,SH3BGRL2, FMR1,NCOA3, GPR107,HMGCL,KDM2B,CTNNBIP1,B3GALT6,NFX1,NADK2,RNF34,RPL14,DTNB, PRKAR1B,MYO7A,CPLANE1,BCAP31,MAPKBP1,SATB2,EZH1,CNBP,KLC1,REPIN1,SMUG1,COX18,MIB2,WWC1,ZNF706,CFL1,GRAMD1A,PRR14,MYL6,PRPF40B,BRF1,MAPKAPK5,EML5,ZNF592,ACTG1,COX6B1,SEC11C,TAF15,APBB1,SCOC,BTBD8,CTCF,MYO15A,ATXN3,DYNC2H1

	BTO: 0001484	Nervous system	0.14	0.0434	OTUD5,CDIPT,RNASEH2A,CCDC92,KALRN,KRT5,RARA,BRD8,SNAP25,APC, AKIRIN2,USP37,FAM8A1,ARG2,SPG11,RAB11A,MTFR1,KMT2C,IFT46,TUBB4A,ZN F280D,MARCHF6,CDK16,NDUFB9,ZC3H12C,NIFK,RNF126,TERF2IP,CA4, GON7,SDC2,FEM1B,XXYLT1,SPNS1,ISCU,MRFAP1,ZKSCAN1,SP1,OSBP2,SMAD3, ABCC5,COQ6,PDE2A,ENSA,KLHL7,PTMA,RPL27A,RPS26,SLC25A29,PCBP2,PJA2, SUCO,SYT11,MCL1,SH3BGRL2,FMR1,NCOA3,GPR107,HMGCL,KDM2B,CTNNBIP 1,B3GALT6,NADK2,RNF34,RPL14,DTNB,PRKAR1B,MYO7A,CPLANE1,BCAP31,M APKBP1,SATB2,EZH1,CNBP,KLC1,REPIN1,SMUG1,COX18,MIB2,WWC1,ZNF706, CFL1,GRAMD1A,MYL6,PRPF40B,BRF1,MAPKAPK5,ZNF592,ACTG1,COX6B1,SEC1 1C,APBB1,SCOC,BTBD8,RTTN,CTCF,MYO15A,ATXN3
	GOCC: 0043226	Organelle	0.22	4.40E-25	COX15,OTUD5,RIN3,CDIPT,MGA,RNASEH2A,RAB3D,RPS12,NPRL2,MED28, CCDC92,KALRN,EAPP,KRT5,RARA,COQ3,BRD8,SNAP25,APC,AKIRIN2,USP37,H US1,BRIP1,FAM8A1,BUD13,CLCN3,ARG2,SPG11,RAB11A,MTFR1,KMT2C,GATB,IF T46,TUBB4A,PITX1,TARBP2,NEK8,SDE2,LIMD1,NDUFS6,MARCHF6,CDK16, NDUFB9,SCLT1,PLEKHH2,NUBP1,NIFK,FZD7,MTERF3,RNF126,MAP4K2,BBS5,MI CALL2,MYORG,POLR2M,TERF2IP,CA4,ZCCHC4,RNFT1,GON7,SDC2,RAMAC, FEM1B,XXYLT1,SPNS1,ERCC4,ISCU,ARR3,ATAD5,ZNF609,RMI1,PIBF1,GEMIN4, ZKSCAN1,MAP11,NR2F1,COX14,TMEM39A,SP1,CASP9,SPPL2C,SMAD3,TMEM179 B,COQ6,PDE2A,EBAG9,TUBB4B,ENSA,LAMTOR4,KLHL7,FCF1,PTMA,RPL27A, NRDE2,FBXL3,RPS26,DYNLRB1,SLC25A29,PCBP2,MT-CO1,PJA2,MT-ND4, DCLRE1A,COQ8A,FGFR1OP,UST,SUCO, SYT11,DPM3,PLEKHA1,MCL1,FMR1, MINPP1,NCOA3,SURF6,GPR107,DOLK,RLF,SLC25A25,BAK1,HMGCL,PBX2,FBXO6 ,KDM2B,CTNNBIP1,ATG2A,RGP1,B3GALT6,NFX1,NHLRC3,RREB1,SDC1,NADK2, NCBP3,RNF34,VTI1A,PRC1,RPL14,EAF1,FGFRL1,SPTLC3,DTNB,METTL15,PRKAR 1B,MARK2,MYO7A,CPLANE1,BCAP31,MAPKBP1,ZNF7,SATB2,EZH1,KAT8,KLC1,

					KANSL2,REPIN1,UBN2,ADAM9,ANAPC13,ZNF827,SMUG1,MRPS30,COX18, WWC1,CCNC,ZNF706,SPA17,CFL1,MEX3A,CDC27,RPS6KA1,TRERF1,GRAMD1A, PRR14,CITED2,MYL6,PRPF40B,BRF1,MAPKAPK5,CNN2,WWOX,ACTG1,CENPX, COX6B1,UQCR11,SEC11C,TAF15,UQCC2,APBB1,SCOC,NSA2,MTRES1,BTBD8,RTT N,CTCF,MYO15A,ATXN3,GET1,DYNC2H1,SLC25A52
	GOCC: 0005622	Intracellular	0.19	1.47E-23	COX15,OTUD5,RIN3,CDIPT,MGA,RNASEH2A,RAB3D,RPS12,NPRL2,MED28, CCDC92,KALRN,EAPP,KRT5,RARA,COQ3,BRD8,SNAP25,APC,AKIRIN2,USP37, HUS1,BRIP1,FAM8A1,BUD13,CLCN3,LPIN2,ARG2,SPG11,RAB11A,MTFR1,KMT2C, GATB,IFT46,TUBB4A,PITX1,HPS5,TARBP2,SDE2,LIMD1,NDUFS6,MARCHF6,CDK1 6, NDUFB9,LDHAL6A,SCLT1,PLEKHH2,NUBP1,NIFK,FZD7,MTERF3, RNF126, MAP4K2,MICALL2,MYORG,POLR2M,TERF2IP,CA4,ZCCHC4,RNFT1, GON7,SDC2, RAMAC,FEM1B,XXYLT1,SPNS1,ERCC4,ISCU,ARR3,ATAD5,ZNF609, RMI1,PIBF1, MRFAP1,GEMIN4,ZKSCAN1,ENO3,MAP11,NR2F1,COX14,TMEM39A, SP1,CASP9, SPPL2C,SMAD3,TMEM179B,COQ6,PDE2A,EBAG9,TUBB4B,ENSA, LAMTOR4, KLHL7,FCF1,PTMA,RPL27A,NRDE2,FBXL3,RPS26,DYNLRB1,UBL5, SLC25A29, PCBP2,MT-CO1,PJA2,MT-ND4, DCLRE1A,COQ8A,FGFR1OP,UST, SUCO,SYT11, DPM3,PLEKHA1,MCL1,SH3BGRL2,FMR1,MINPP1,NCOA3,SURF6, GPR107,DOLK,RLF,SLC25A25,BAK1,HMGCL,PBX2,STK19,FBXO6,KDM2B,CTNNBI P1,ATG2A, RGP1,B3GALT6,NFX1,NHLRC3,RREB1,SDC1,NADK2,NCBP3,RNF34, VTI1A,PRC1, RPL14,EAF1,FGFRL1,SPTLC3,DTNB,METTL15,PRKAR1B,MARK2, MYO7A,BCAP31, MAPKBP1,ZNF7,WDR20,CCN1,SATB2,NR2C2AP,EZH1,KAT8, CNBP,KLC1,KANSL2, DUSP7,REPIN1,UBN2,ANAPC13,ZNF827,SMUG1,MRPS30, COX18,MIB2,WWC1, CCNC,ZNF706,SPA17,CFL1,MEX3A,CDC27,RPS6KA1, TRERF1,GRAMD1A,PRR14, CITED2,MYL6,PRPF40B,BRF1,MAPKAPK5,CNN2,

					WWOX,ACTG1,CENPX, COX6B1,UQCR11,SEC11C,TAF15,UQCC2,APBB1,SCOC, NSA2,MTRES1,BTBD8, RTTN,CTCF,MYO15A,ATXN3,GET1,DYNC2H1,SLC25A52
	GOCC: 0043229	Intracellular organelle	0.22	5.44E-22	COX15,OTUD5,RIN3,CDIPT,MGA,RNASEH2A,RAB3D,RPS12,NPRL2,MED28, CCDC92,KALRN,EAPP,KRT5,RARA,COQ3,BRD8,SNAP25,APC,AKIRIN2,USP37,HUS1,BRIP1,FAM8A1,BUD13,CLCN3,ARG2,SPG11,RAB11A,MTFR1,KMT2C,GATB,TUBB4A,PITX1,TARBP2,SDE2,LIMD1,NDUFS6,MARCHF6,CDK16,NDUFB9,SCLT1, PLEKHH2,NUBP1,NIFK,FZD7,MTERF3,RNF126,MICALL2,MYORG,POLR2M, TERF2IP,CA4,ZCCHC4,RNFT1,GON7,SDC2,RAMAC,FEM1B,XXYLT1,SPNS1, ERCC4,ISCU,ATAD5,ZNF609,RMI1,PIBF1,GEMIN4,ZKSCAN1,MAP11,NR2F1, COX14,TMEM39A,SP1,CASP9,SPPL2C,SMAD3,TMEM179B,COQ6,PDE2A,EBAG9,TUBB4B,ENSA,LAMTOR4,KLHL7,FCF1,PTMA,RPL27A,NRDE2,FBXL3, RPS26,DYNLRB1,SLC25A29,PCBP2,MT-CO1,PJA2,MT-ND4,DCLRE1A,COQ8A, FGFR1OP,UST,SUCO,SYT11,DPM3,PLEKHA1,MCL1, FMR1,MINPP1,NCOA3, SURF6,GPR107,DOLK,RLF,SLC25A25,BAK1,HMGCL,PBX2,FBXO6,KDM2B,CTNNBIP1,RGP1,B3GALT6,NFX1,NHLRC3,RREB1,SDC1,NADK2,NCBP3,RNF34,VTI1A,PRC1,RPL14,EAF1,FGFRL1,SPTLC3,DTNB,METTL15,MARK2,MYO7A,BCAP31,MAPKBP1,ZNF7,SATB2,EZH1,KAT8,KLC1,KANSL2,REPIN1,UBN2,ANAPC13,ZNF827, SMUG1,MRPS30,COX18,WWC1,CCNC,ZNF706,CFL1,MEX3A,CDC27,RPS6KA1,TRERF1,GRAMD1A,PRR14,CITED2,MYL6,PRPF40B,BRF1,MAPKAPK5,CNN2, WWOX,ACTG1,CENPX,COX6B1,UQCR11,SEC11C,TAF15,UQCC2,APBB1,SCOC,NSA2,MTRES1,BTBD8,RTTN,CTCF,MYO15A,ATXN3,GET1,DYNC2H1,SLC25A52
	GOCC: 0043231	Intracellular membrane-	0.25	1.88E-19	COX15,OTUD5,RIN3,CDIPT,MGA,RNASEH2A,RAB3D,NPRL2,MED28,EAPP,KRT5, RARA,COQ3,BRD8,SNAP25,APC,AKIRIN2,USP37,HUS1,BRIP1,FAM8A1,BUD13,CLCN3,ARG2,SPG11,RAB11A,MTFR1,KMT2C,GATB,PITX1,TARBP2,SDE2,LIMD1,NDUFS6,MARCHF6,NDUFB9,NIFK,FZD7,MTERF3,RNF126,MICALL2,MYORG,

		bounded organelle			POLR2M,TERF2IP,CA4,ZCCHC4,RNFT1,GON7,SDC2,RAMAC,FEM1B,XXYLT1, SPNS1,ERCC4,ISCU,ZNF609,RMI1,PIBF1,GEMIN4,ZKSCAN1,MAP11,NR2F1, COX14,TMEM39A,SP1,CASP9,SPPL2C,SMAD3,TMEM179B,COQ6,PDE2A,EBAG9,TUBB4B,ENSA,LAMTOR4,KLHL7,FCF1,PTMA,NRDE2,FBXL3,RPS26,SLC25A29, PCBP2,MT-CO1,MT-ND4,DCLRE1A,COQ8A,FGFR1OP,UST,SUCO,SYT11,DPM3, PLEKHA1,MCL1,FMR1, MINPP1,NCOA3,SURF6,GPR107,DOLK,RLF,SLC25A25, BAK1,HMGCL,PBX2,FBXO6,KDM2B,CTNNBIP1,RGP1,B3GALT6,NFX1,NHLRC3,RREB1,SDC1,NADK2,NCBP3,RNF34,VTI1A,PRC1,EAF1,FGFRL1,SPTLC3,DTNB,METTL15,MARK2,MYO7A,BCAP31,MAPKBP1,ZNF7,SATB2,EZH1,KAT8,KANSL2,REPIN1,UBN2,ANAPC13,SMUG1,MRPS30,COX18,WWC1,CCNC,ZNF706,CFL1,CDC27,RPS6KA1,TRERF1,GRAMD1A,PRR14,CITED2,PRPF40B,BRF1,MAPKAPK5,CNN2, WWOX,CENPX,COX6B1,UQCR11,SEC11C,TAF15,UQCC2,APBB1,SCOC,NSA2,MTRES1,BTBD8,CTCF,ATXN3,GET1,DYNC2H1,SLC25A52
	GOCC: 0043227	Membrane-bounded organelle	0.22	3.09E-18	COX15,OTUD5,RIN3,CDIPT,MGA,RNASEH2A,RAB3D,NPRL2,MED28,EAPP,KRT5, RARA,COQ3,BRD8,SNAP25,APC,AKIRIN2,USP37,HUS1,BRIP1,FAM8A1,BUD13,CLCN3,ARG2,SPG11,RAB11A,MTFR1,KMT2C,GATB,TUBB4A,PITX1,TARBP2,SDE2,LIMD1,NDUFS6,MARCHF6,NDUFB9,SCLT1,NIFK,FZD7,MTERF3,RNF126,MAP4K2, BBS5,MICALL2,MYORG,POLR2M,TERF2IP,CA4,ZCCHC4,RNFT1,GON7,SDC2,RAMAC,FEM1B,XXYLT1,SPNS1,ERCC4,ISCU,ZNF609,RMI1,PIBF1,GEMIN4, ZKSCAN1,MAP11,NR2F1,COX14,TMEM39A,SP1,CASP9,SPPL2C,SMAD3,TMEM179B,COQ6,PDE2A,EBAG9,TUBB4B,ENSA,LAMTOR4,KLHL7,FCF1,PTMA,NRDE2, FBXL3,RPS26,SLC25A29,PCBP2,MT-CO1,MT-ND4,DCLRE1A,COQ8A,FGFR1OP, UST,SUCO, SYT11,DPM3,PLEKHA1,MCL1,FMR1,MINPP1,NCOA3,SURF6,GPR107, DOLK,RLF,SLC25A25,BAK1,HMGCL,PBX2,FBXO6,KDM2B,CTNNBIP1,RGP1,B3GALT6,NFX1,NHLRC3,RREB1,SDC1,NADK2,NCBP3,RNF34,VTI1A,PRC1,EAF1,FGFRL

					1,SPTLC3,DTNB,METTL15,MARK2,MYO7A,BCAP31,MAPKBP1,ZNF7,SATB2,EZH1,KAT8,KANSL2,REPIN1,UBN2,ADAM9,ANAPC13,SMUG1,MRPS30,COX18,WWC1,CCNC,ZNF706,SPA17,CFL1,CDC27,RPS6KA1,TRERF1,GRAMD1A,PRR14,CITED2,PRPF40B,BRF1,MAPKAPK5,CNN2,WWOX,ACTG1,CENPX,COX6B1,UQCR11,SEC11C,TAF15,UQCC2,APBB1,SCOC,NSA2,MTRES1,BTBD8,RTTN,CTCF,ATXN3,GET1,DYNC2H1,SLC25A52
	GOCC: 0110165	Cellular anatomical entity	0.11	5.20E-12	COX15,OTUD5,RIN3,CDIPT,MGA,RNASEH2A,RAB3D,RPS12,NPRL2,MED28,CCDC92,KALRN,EAPP,KRT5,RARA,COQ3,BRD8,SNAP25,APC,AKIRIN2,USP37,HUS1,BRIP1,FAM8A1,BUD13,CLCN3,ARG2,SPG11,RAB11A,MTFR1,KMT2C,GATB,IFT46,TUBB4A,PITX1,HPS5,TARBP2,NEK8,SDE2,LIMD1,NDUFS6,MARCHF6,CDK16,NDUFB9,LDHAL6A,SCLT1,PLEKHH2,GRM1,NUBP1,NIFK,FZD7,MTERF3,RNF126,MAP4K2,BBS5,MICALL2,MYORG,POLR2M,TERF2IP,CA4,ZCCHC4,RNFT1,GON7,SDC2,RAMAC,FEM1B,TSPAN5,XXYLT1,SPNS1,ERCC4,ISCU,ARR3,ATAD5,ZNF609,RMI1,PIBF1,GEMIN4,ZKSCAN1,ENO3,MAP11,NR2F1,COX14,TMEM39A,SP1,CASP9,SPPL2C,OSBP2,SMAD3,TMEM179B,ABCC5,COQ6,PDE2A,EBAG9,TUBB4B,ENSA,LAMTOR4,KLHL7,FCF1,PTMA,RPL27A,NRDE2,FBXL3,RPS26,DYNLRB1,UBL5,SLC25A29,PCBP2,MT-CO1,PJA2,MT-ND4,DCLRE1A,COQ8A,FGFR1OP,UST,XPR1,SUCO,SYT11,DPM3,PLEKHA1, MCL1,FMR1,MINPP1,NCOA3,SURF6,GPR107,DOLK,RLF,SLC25A25,SLC31A1,BAK1,HMGCL,PBX2,FBXO6,KDM2B,CTNNBIP1,ATG2A,RGP1,B3GALT6,NFX1,NHLRC3,RREB1,SDC1,NADK2,NCBP3,RNF34,VTI1A,PRC1,RPL14,EAF1,FGFRL1,SPTLC3,DTNB,METTL15,PRKAR1B,MARK2,MYO7A,CPLANE1,BCAP31,MAPKBP1,ZNF7,CCN1,SATB2,EZH1,KAT8,KLC1,KANSL2,DUSP7,REPIN1,UBN2,ADAM9,ANAPC13,ZNF827,SORCS2,SMUG1,MRPS30,COX18,MIB2,WWC1,CCNC,ZNF706,SPA17,CFL1,MEX3A,CDC27,RPS6KA1,TRERF1,GRAMD1A,PRR14,CITED2,MYL6,PRPF40B,BRF1,MAPKAPK5,

					CNN2,WWOX,ACTG1,CENPX,COX6B1,UQCR11,SEC11C,TAF15,UQCC2,APBB1,SCOC,NSA2,MTRES1,BTBD8,RTTN,CTCF,MYO15A,ATXN3,GET1, DYNC2H1,SLC25A52
	GOCC: 0005737	Cytoplasm	0.19	2.34E-09	COX15,OTUD5,RIN3,CDIPT,RNASEH2A,RAB3D,RPS12,NPRL2,KALRN,EAPP, KRT5,RARA,COQ3,SNAP25,APC,BRIP1,FAM8A1,CLCN3,ARG2,SPG11,RAB11A,MTFR1,GATB,TUBB4A,HPS5,TARBP2,SDE2,LIMD1,NDUFS6,MARCHF6,CDK16, NDUFB9,LDHAL6A,SCLT1,PLEKHH2,NUBP1,NIFK,FZD7,MTERF3,RNF126,MAP4K2,MICALL2,TERF2IP,CA4,ZCCHC4,RNFT1,SDC2,FEM1B,XXYLT1,SPNS1, ISCU,ARR3,GEMIN4,ENO3,MAP11,COX14,TMEM39A,SP1,CASP9,SPPL2C,SMAD3, TMEM179B,COQ6,PDE2A,EBAG9,TUBB4B,LAMTOR4,KLHL7,PTMA,RPL27A, NRDE2,FBXL3,RPS26,DYNLRB1,UBL5,SLC25A29,PCBP2,MT-CO1,PJA2,MT-ND4, COQ8A,FGFR1OP,UST,SUCO,SYT11,DPM3,PLEKHA1,MCL1,FMR1,MINPP1,NCOA3,GPR107,DOLK,SLC25A25,BAK1,HMGCL,FBXO6,CTNNBIP1,ATG2A,RGP1,B3GALT6,NFX1,NHLRC3,RREB1,SDC1,NADK2,NCBP3,RNF34,VTI1A,RPL14,FGFRL1,SPTLC3,DTNB,METTL15,PRKAR1B,MARK2,MYO7A,BCAP31,MAPKBP1,CCN1,KANSL2,DUSP7,MRPS30,COX18,MIB2,WWC1,SPA17,CFL1,MEX3A,RPS6KA1,GRAMD1A, MYL6,MAPKAPK5,CNN2,WWOX,ACTG1,COX6B1,UQCR11,SEC11C,TAF15,UQCC2,APBB1,SCOC,MTRES1,BTBD8,ATXN3,GET1,DYNC2H1,SLC25A52
	GOCC: 0005634	Nucleus	0.23	1.63E-06	OTUD5,MGA,RNASEH2A,MED28,EAPP,KRT5,RARA,BRD8,APC,AKIRIN2,USP37, HUS1,BRIP1,BUD13,SPG11,KMT2C,PITX1,TARBP2,SDE2,LIMD1,MARCHF6,NIFK, RNF126,MYORG,POLR2M,TERF2IP,ZCCHC4,GON7,RAMAC,FEM1B,ERCC4, ZNF609,RMI1,PIBF1,GEMIN4,ZKSCAN1,NR2F1,SP1,SMAD3,PDE2A,EBAG9, ENSA,KLHL7,FCF1,PTMA,NRDE2,FBXL3,RPS26,PCBP2,DCLRE1A,FGFR1OP,PLEKHA1,MCL1,FMR1,NCOA3,SURF6,RLF,PBX2,KDM2B,NFX1,RREB1,NCBP3,RNF34,PRC1,EAF1,DTNB,MARK2,MAPKBP1,SATB2,EZH1,KAT8,KANSL2,REPIN1,UBN2,

					ANAPC13,SMUG1,WWC1,CCNC,ZNF706,CFL1,CDC27,RPS6KA1,TRERF1,PRR14,CITED2,PRPF40B,BRF1,MAPKAPK5,WWOX,CENPX,TAF15,APBB1,SCOC,NSA2,BTBD8,CTCF,ATXN3
	GOCC: 0032991	Protein-containing complex	0.2	8.06E-05	COX15,MGA,RNASEH2A,RPS12,NPRL2,MED28,RARA,COQ3,BRD8,SNAP25,APC,AKIRIN2,HUS1,BRIP1,FAM8A1,BUD13,RAB11A,KMT2C,GATB,IFT46,HPS5,TARBP2,LIMD1,NDUFS6,MARCHF6,NDUFB9,SCLT1,GRM1,NIFK,BBS5,POLR2M,TERF2IP,GON7,RAMAC,FEM1B,UTP23,ERCC4,ISCU,ATAD5,RMI1,GEMIN4,ENO3,MAP11,SP1,CASP9,SMAD3,COQ6,EBAG9,LAMTOR4,KLHL7,FCF1,FBXL3,RPS26,DYNLRB1,MT-CO1,MT-ND4,SUCO,DPM3,MCL1,FMR1,NCOA3,SLC31A1,BAK1,HMGCL,FBXO6,CTNNBIP1,RGP1,SDC1,NCBP3,VTI1A,EAF1,SPTLC3,DTNB,PRKAR1B,MYO7A,EZH1,KAT8,KLC1,KANSL2,REPIN1,ANAPC13,ZNF827,MRPS30,CCNC,CFL1,CDC27,MYL6,PRPF40B,BRF1,ACTG1,CENPX,COX6B1,UQCR11,SEC11C,NSA2,CTCF,MYO15A,GET1,DYNC2H1
	GOCC: 0043232	Intracellular non-membrane-bounded organelle	0.24	0.00051	RAB3D,RPS12,CCDC92,KALRN,KRT5,RARA,BRD8,SNAP25,APC,HUS1,RAB11A,TUBB4A,LIMD1,CDK16,SCLT1,PLEKHH2,NUBP1,NIFK,TERF2IP,ZCCHC4,ERCC4,ATAD5,RMI1,PIBF1,GEMIN4,MAP11,SP1,SMAD3,TUBB4B,RPL27A,NRDE2,RPS26,DYNLRB1,MT-CO1,PJA2,MT-ND4,DCLRE1A,FGFR1OP,FMR1,NCOA3,SURF6,RREB1,PRC1,RPL14,MARK2,MYO7A,BCAP31,MAPKBP1,EZH1,KLC1,KANSL2,REPIN1,ZNF827,SMUG1,MRPS30,CFL1,MEX3A,CDC27,TRERF1,MYL6,CNN2,ACTG1,CENPX,UQCC2,RTTN,CTCF,MYO15A,DYNC2H1
	GOCC: 0019866	Organelle inner membrane	0.55	0.0038	COX15,COQ3,NDUFS6,MARCHF6,NDUFB9,SMAD3,COQ6,PDE2A,EBAG9,SLC25A29,MT-CO1,MT-ND4,COX18,COX6B1,UQCR11,UQCC2

	GOCC: 0031090	Organelle membrane	0.26	0.0058	COX15,CDIPT,RAB3D,NPRL2,COQ3,SNAP25,BRIP1,FAM8A1,CLCN3,SPG11,RAB11A,NDUFS6,MARCHF6,NDUFB9,FZD7,MTERF3,BBS5,MYORG,CA4,XXYLT1,SPNS1,TMEM39A,SPPL2C,SMAD3,TMEM179B,COQ6,PDE2A,EBAG9,LAMTOR4,RPS26,SLC25A29,MT-CO1,MT-ND4,SUCO,DPM3,MCL1,DOLK,BAK1,RGP1,VTI1A,SPTLC3,MYO7A,BCAP31,COX18,GRAMD1A,COX6B1,UQCR11,SEC11C,UQCC2
	GOCC: 0005746	Mitochondrial respirasome	0.88	0.0102	COX15,NDUFS6,NDUFB9,MT-CO1,MT-ND4,COX6B1,UQCR11
	GOCC: 0005739	Mitochondrion	0.33	0.0128	COX15,COQ3,ARG2,MTFR1,GATB,NDUFS6,NDUFB9,MTERF3,ISCU,COX14,CASP9,COQ6,PDE2A,SLC25A29,MT-CO1,MT-ND4,COQ8A,MCL1,SLC25A25,BAK1,HMGCL,NADK2,METTL15,MRPS30,COX18,COX6B1,UQCR11,UQCC2,MTRES1,SLC25A52
	GOCC: 1902494	Catalytic complex	0.27	0.0202	MGA,BRD8,APC,FAM8A1,KMT2C,TARBP2,NDUFS6,MARCHF6,NDUFB9,NIFK,POLR2M,RAMAC,FEM1B,ISCU,ATAD5,ENO3,CASP9,EBAG9,KLHL7,FBXL3,DYNLRB1,MT-CO1,MT-ND4,DPM3,FBXO6,SDC1,SPTLC3,EZH1,KAT8,KANSL2,ANAPC13,CCNC,CDC27,PRPF40B,UQCR11,SEC11C,CTCF,DYNC2H1
	GOCC: 0005743	Mitochondrial inner membrane	0.51	0.0329	COX15,COQ3,NDUFS6,NDUFB9,COQ6,PDE2A,SLC25A29,MT-CO1,MT-ND4,COX18,COX6B1,UQCR11,UQCC2
	GOCC: 0031966	Mitochondrial membrane	0.44	0.0355	COX15,COQ3,NDUFS6,NDUFB9,MTERF3,COQ6,PDE2A,SLC25A29,MT-CO1,MT-ND4,MCL1,BAK1,COX18,COX6B1,UQCR11,UQCC2
	GOCC: 0031981	Nuclear lumen	0.25	0.0376	MGA,MED28,RARA,BRD8,USP37,HUS1,KMT2C,SDE2,NIFK,POLR2M,TERF2IP,ZCCHC4,RMI1,GEMIN4,SP1,SMAD3,NRDE2,RPS26,DCLRE1A,MCL1,FMR1,

					NCOA3,SURF6,KDM2B,RREB1,NCBP3,EAF1,MAPKBP1,SATB2,EZH1,KAT8,KANSL2,REPIN1,SMUG1,CCNC,TRERF1,PRR14,CENPX,CTCF
	GOCC: 0045202	Synapse	0.44	0.0376	KALRN,SNAP25,CLCN3,SPG11,RAB11A,GRM1,ARR3,PDE2A,SYT11,FMR1,RPL14,DTNB,MYO7A,ACTG1,APBB1,BTBD8
	GOCC: 0070013	Intracellular organelle lumen	0.2	0.0471	MGA,MED28,RARA,COQ3,BRD8,USP37,HUS1,ARG2,KMT2C,SDE2,NIFK,POLR2M,TERF2IP,ZCCHC4,SDC2,RMI1,GEMIN4,SP1,SMAD3,TUBB4B,NRDE2,RPS26,MT-CO1,MT-ND4,DCLRE1A,MCL1,FMR1,NCOA3,SURF6,HMGCL,KDM2B,NHLRC3,RREB1,SDC1,NADK2,NCBP3,EAF1,METTL15,MAPKBP1,SATB2,EZH1,KAT8,KANSL2,REPIN1,SMUG1,MRPS30,CCNC,TRERF1,PRR14,CNN2,CENPX,UQCC2,MTRES1,CTCF
	KW-0597	Phosphoprotein	0.15	4.01E-05	OTUD5,MGA,RNASEH2A,RAB3D,KALRN,SGPP1,EAPP,KRT5,RARA,BRD8,SNAP25,APC,AKIRIN2,USP37,BRIP1,FAM8A1,BUD13,LPIN2,SPG11,MTFR1,KMT2C,IFT46,TUBB4A,PITX1,HPS5,TARBP2,ZNF280D,NEK8,SDE2,LIMD1,CDK16,NDUFB9,ZC3H12C,LDHAL6A,SCLT1,GRM1,NUBP1,NIFK,RNF126,MAP4K2,MICALL2,TERF2IP,SDC2,RAMAC,UTP23,SPNS1,ERCC4,ATAD5,ZNF609,RMI1,GEMIN4,ZKSCAN1,ENO3,MAP11,SP1,CASP9,OSBP2,SMAD3,TMEM179B,ABCC5,EBAG9,TUBB4B,ENSA,PTMA,RPL27A,RPS26,PCBP2,PJA2,DCLRE1A,FGFR1OP,XPR1,SUCO,SYT11,PLEKHA1,MCL1,FMR1,NCOA3,SURF6,RLF,SLC31A1,PBX2,FBXO6,KDM2B,CTNNBIP1,ATG2A,NFX1,RREB1,SDC1,NADK2,NCBP3,RNF34,PRC1,RPL14,EAF1,DTNB,METTL15,PRKAR1B,MARK2,MYO7A,CPLANE1,MAPKBP1,ZNF7,WDR20,CCN1,SATB2,KAT8,CNBP,KLC1,KANSL2,REPIN1,UBN2,ADAM9,MIB2,WWC1,CCNC,TXNRD3,CFL1,MEX3A,CDC27,RPS6KA1,TRERF1,GRAMD1A,PRR14,MYL6,PRPF40B,BRF1,MAPKAPK5,ZNF592,CNN2,WWOX,TAF15,APBB1,NSA2,MTRES1,RTTN,CTCF,ATXN3

	KW-0539	Nucleus	0.2	0.00014	MGA,RNASEH2A,MED28,EAPP,RARA,BRD8,AKIRIN2,HUS1,BRIP1,BUD13,LPIN2,SPG11,KMT2C,PITX1,TARBP2,ZNF280D,SDE2,LIMD1,NUBP1,NIFK,RNF126,MYORG,POLR2M,TERF2IP,ZCCHC4,GON7,RAMAC,FEM1B,UTP23,ERCC4,ISCU,ATAD5,ZNF609,RMI1,PIBF1,MRFAP1,GEMIN4,ZKSCAN1,NR2F1,SP1,SMAD3,KLHL7,FCF1,PTMA,NRDE2,FBXL3,PCBP2,DCLRE1A,PLEKHA1,MCL1,SH3BGRL2,FMR1,NCOA3,SURF6,RLF,PBX2,STK19,KDM2B,CTNNBIP1,NFX1,RREB1,NCBP3,RNF34,PRC1,EAF1,MAPKBP1,ZNF7,SATB2,NR2C2AP,EZH1,KAT8,KANSL2,REPIN1,ANAPC13,ZNF827,SMUG1,WWC1,CCNC,TXNRD3,ZNF706,CFL1,MEX3A,CDC27,RPS6KA1,TRERF1,PRR14,CITED2,PRPF40B,BRF1,MAPKAPK5,ZNF592,WWOX,CENPX,TAF15,APBB1,NSA2,CTCF,ATXN3
	KW-0999	Mitochondrion inner membrane	0.63	0.00097	COQ3,NDUFS6,NDUFB9,SPNS1,COQ6,PDE2A,SLC25A29,MT-CO1,SLC25A25,COX18,SLC25A30,COX6B1,UQCR11,UQCC2,SLC25A52
	KW-0832	Ubl conjugation	0.26	0.0021	MGA,RARA,BRD8,APC,USP37,BUD13,ZNF280D,SDE2,MARCHF6,LDHAL6A,NIFK,FZD7,RNF126,MAP4K2,TERF2IP,UTP23,ERCC4,ATAD5,ZNF609,RMI1,ZKSCAN1,SP1,SMAD3,PTMA,PCBP2,DCLRE1A,SYT11,MCL1,FMR1,RLF,KDM2B,NFX1,RREB1,NCBP3,RNF34,RPL14,ZNF7,SATB2,EZH1,KANSL2,UBN2,ZNF827,MIB2,CFL1,PRPF40B,ZNF592,WWOX,TAF15,APBB1,NSA2,CTCF,ATXN3
	KW-1017	Isopeptide bond	0.31	0.0021	MGA,RARA,BRD8,BUD13,TUBB4A,ZNF280D,LDHAL6A,NIFK,TERF2IP,UTP23,ERCC4,ATAD5,ZNF609,RMI1,ZKSCAN1,SP1,SMAD3,TUBB4B,PTMA,PCBP2,DCLRE1A,MCL1,RLF,KDM2B,RREB1,NCBP3,RPL14,ZNF7,SATB2,EZH1,KANSL2,UBN2,ZNF827,CFL1,PRPF40B,ZNF592,ACTG1,TAF15,NSA2,CTCF,ATXN3

	KW-1274	Primary mitochondrial disease	0.73	0.0031	COX15,NDUFS6,NDUFB9,COX14,COQ6,MT-CO1,MT-ND4,<br>COQ8A,COX6B1,UQCC2
	KW-0496	Mitochondrion	0.35	0.0033	COX15,COQ3,ARG2,MTFR1,GATB,NDUFS6,NDUFB9,MTERF3,SPNS1,ISCU,COX14,<br>COQ6,PDE2A,SLC25A29,MT-CO1,MT-ND4,COQ8A,MCL1,SLC25A25,BAK1,<br>HMGCL,NADK2,MRPS30,COX18,SLC25A30,WWOX,COX6B1,UQCR11,UQCC2,MT<br>RES1,SLC25A52
	KW-0007	Acetylation	0.21	0.0047	RNASEH2A,RAB3D,RPS12,EAPP,COQ3,BRD8,APC,BRIP1,RAB11A,KMT2C,<br>PITX1,NDUFS6,MARCHF6,NDUFB9,LDHAL6A,SCLT1,NUBP1,NIFK,<br>RNF126,TERF2IP,GON7,RAMAC,SPNS1,ERCC4,RMI1,GEMIN4,ENO3,SP1,SMAD3,<br>TUBB4B,ENSA,LAMTOR4,PTMA,RPL27A,NRDE2,DYNLRB1,PJA2,FMR1,NCOA3,<br>BAK1,HMGCL,NADK2,RPL14,DTNB,MAPKBP1,WDR20,KAT8,CNBP,REPIN1,<br>UBN2,MIB2,SLC25A30,CFL1,TRERF1,PRR14,MYL6,PRPF40B,CNN2,ACTG1,CENPX,<br>COX6B1,TAF15,RTTN,CTCF
	KW-0010	Activator	0.41	0.0065	MED28,KMT2C,PITX1,TERF2IP,ZNF609,NR2F1,SP1,FMR1,NCOA3,PBX2,NFX1,RRE<br>B1,EAF1,KAT8,WWC1,CCNC,TRERF1,CITED2,BRF1,APBB1,CTCF
	KW-0963	Cytoplasm	0.15	0.0166	RIN3,RPS12,MED28,CCDC92,KALRN,EAPP,RARA,SNAP25,APC,HUS1,BRIP1,LPIN<br>2,SPG11,IFT46,TUBB4A,HPS5,TARBP2,NEK8,LIMD1,CDK16,LDHAL6A,SCLT1,PLE<br>KHH2,NUBP1,RNF126,MAP4K2,BBS5,MICALL2,TERF2IP,PPIC,ZCCHC4,FEM1B,<br>ISCU,PIBF1,MRFAP1,GEMIN4,ENO3,MAP11,SP1,SMAD3,PDE2A,TUBB4B,ENSA,<br>KLHL7,FBXL3,RPS26,DYNLRB1,UBL5,PCBP2,PJA2,FGFR1OP,PLEKHA1,MCL1,FM<br>R1,NCOA3,FBXO6,CTNNBIP1,RGP1,NCBP3,RNF34,PRC1,DTNB,MARK2,MYO7A,<br>MAPKBP1,CNBP,KLC1,DUSP7,MIB2,WWC1,TXNRD3,ZNF706,CFL1,MEX3A,RPS6K

					A1,MAPKAPK5,EML5,WWOX,ACTG1,TAF15,APBB1,SCOC,RTTN,MYO15A,DYNC2H1
	KW-0831	Ubiquinone biosynthesis	1.36	0.0319	COQ3,COQ6,COQ8A
SW1353 0.5 Gy	CL:34253	Pachyonychia congenita, and Epidermolysis bullosa simplex Dowling–Meara type	1.93	0.0039	KRT5,KRT6B,KRT16,KRT6A
	BTO: 0000000	Tissues, cell types and enzyme sources	0.08	6.70E-06	FAM76A,ERCC1,COX15,OTUD5,DHPS,DHRS7,ABCD1,C16orf70,SETD6,GSR,RAB3D,ARHGEF6,ABHD17A,KRT5,KRT6B,SESN2,GSE1,RARA,OC90,BRD8,TERF2,MPHOSPH6,UNC13C,PPP2R3C,CLCN3,KMT2C,RHBDF1,FARP2,DGKD,DNAH5,HPS5,CHST7,DOCK11,HAUS1,ATP6V0D2,MRAS,ACSM3,TEX55,LMBRD2,FABP5,IKBIP,KRT16,SDHAF2,PANK1,LRP8,UBE2E1,RNFT1,RFXANK,GON7,KRT74,TSPAN5,ERCC4,KRT2,TNS3,ATAD5,SLC36A4,MPI,IGDCC4,ZMYM3,E2F7,NR2F1,LNX2,SAMD9L,NADSYN1,RSBN1L,STARD10,TNRC6C,EBAG9,KPTN,APOL4,TMEM248,ENSA,KLHL7,CORO2A,DALRD3,FYN,POLR3H,UBE2C,SGO2,HSD17B11,WDR55,PBLD,H3C12,AKR1B10,COL4A2,PCMTD1,SCAF1,SHISA4,IBA57,FBXO28,XPR1,CEP350,SYT11,S100A9,LHPP,SH3BGRL2,GIPC2,VAV2,IL13RA2,RLF,SLC29A3,NDUFA8,SNX30,LPAR1,PBX2,CARNMT1,CLSTN1,RGP1,PLCB4,COX7A2L,AGRN,RREB1,KRT6A,CNTLN,SPAG1,PTOV1,ORMDL3,ELOVL6,ZNF143,DYNC1I2,HOOK2,CABIN1,CST3,FGFRL1,FKBP1A,METTL15,IFRD1,RASSF8,ZBTB40,MEX3C,PRKAR1B,MAP3K2,RDH13,FAM133B,SATB2,NDRG1,SYBU,DUSP7,RPL7L1,RWDD2B,PDK2,MACROH2

					A1,MRPS30,WWC1,RAB15,HSPA8,TNKS1BP1,TDRD3,ATF7IP,GRAMD1A,PRR14,MFSD5,TMEM258,EVI5L,ERH,XRCC3,LDLR,RAD51D,JOSD2,MAGEB5,APBB1,SSBP2,NEB,PIGN,GET1,DYNC2H1,ENOSF1,APLP2,UBE2W,NOTCH1,CARD8,H3-2
	BTO: 0000042	Animal	0.08	8.69E-06	FAM76A,ERCC1,COX15,OTUD5,DHPS,DHRS7,ABCD1,C16orf70,SETD6,GSR,RAB3D,ARHGEF6,ABHD17A,KRT5,KRT6B,SESN2,GSE1,RARA,OC90,BRD8,TERF2,MPHOSPH6,UNC13C,PPP2R3C,CLCN3,KMT2C,RHBDF1,FARP2,DGKD,DNAH5,HPS5,CHST7,DOCK11,HAUS1,ATP6V0D2,MRAS,ACSM3,TEX55,LMBRD2,FABP5,IKBIP,KRT16,SDHAF2,PANK1,LRP8,UBE2E1,RNFT1,RFXANK,GON7,KRT74,TSPAN5,ERCC4,KRT2,TNS3,ATAD5,SLC36A4,MPI,IGDCC4,ZMYM3,E2F7,NR2F1,LNX2,SAMD9L,NADSYN1,RSBN1L,STARD10,TNRC6C,EBAG9,KPTN,APOL4,TMEM248,ENSA,KLHL7,CORO2A,DALRD3,FYN,POLR3H,UBE2C,SGO2,HSD17B11,WDR55,PBLD,H3C12,AKR1B10,COL4A2,PCMTD1,SCAF1,SHISA4,IBA57,FBXO28,XPR1,CEP350,SYT11,S100A9,LHPP,SH3BGRL2,GIPC2,VAV2,IL13RA2,RLF,SLC29A3,NDUFA8,SNX30,LPAR1,PBX2,CARNMT1,CLSTN1,RGP1,PLCB4,COX7A2L,AGRN,RREB1,KRT6A,CNTLN,SPAG1,PTOV1,ORMDL3,ELOVL6,ZNF143,DYNC1I2,HOOK2,CABIN1,CST3,FGFRL1,FKBP1A,METTL15,IFRD1,RASSF8,ZBTB40,MEX3C,PRKAR1B,MAP3K2,RDH13,FAM133B,SATB2,NDRG1,SYBU,DUSP7,RPL7L1,RWDD2B,PDK2,MACROH2A1,MRPS30,WWC1,RAB15,HSPA8,TNKS1BP1,TDRD3,ATF7IP,GRAMD1A,PRR14,MFSD5,TMEM258,EVI5L,ERH,XRCC3,LDLR,RAD51D,JOSD2,MAGEB5,APBB1,SSBP2,NEB,PIGN,GET1,DYNC2H1,ENOSF1,APLP2,UBE2W,NOTCH1,CARD8
	GOCC: 0005622	Intracellular	0.12	7.57E-05	FAM76A,ERCC1,COX15,OTUD5,DHPS,DHRS7,ABCD1,C16orf70,SETD6,GSR,RAB3D,ARHGEF6,ABHD17A,KRT5,KRT6B,SESN2,RARA,BRD8,TERF2,MPHOSPH6,UNC13C,PPP2R3C,CLCN3,KMT2C,RHBDF1,FARP2,DGKD,DNAH5,HPS5,CHST7,DOCK11,LDHAL6A,HAUS1,ATP6V0D2,ACSM3,FABP5,IKBIP,KRT16,SDHAF2,PANK1,H2

					AC8,UBE2E1,RNFT1,RFXANK,GON7,KRT74,ERCC4,KRT2,TNS3,ATAD5,MPI,ZMYM3,E2F7,NR2F1,NADSYN1,STARD10,TNRC6C,EBAG9,KPTN,ENSA,KLHL7,FYN,POLR3H,UBE2C,SGO2,HSD17B11,WDR55,PBLD,H3C12,AKR1B10,COL4A2,IBA57,FBXO28,CEP350,SYT11,S100A9,LHPP,SH3BGRL2,VAV2,RLF,SLC29A3,NDUFA8,SNX30,LPAR1,PBX2,CARNMT1,RGP1,PLCB4,COX7A2L,AGRN,RREB1,KRT6A,CNTLN,SPAG1,PTOV1,FDX2,ORMDL3,ELOVL6,ZNF143,DYNC1I2,HOOK2,CABIN1,CST3,FGFRL1,FKBP1A,METTL15,ZBTB40,PRKAR1B,MAP3K2,RDH13,SATB2,NDRG1,SYBU,DUSP7,PDK2,MACROH2A1,MRPS30,WWC1,RAB15,HSPA8,TNKS1BP1,TDRD3,ATF7IP,GRAMD1A,PRR14,TMEM258,LYRM4,ERH,XRCC3,LDLR,RAD51D,JOSD2,FBLL1,APBB1,SSBP2,NEB,GET1,DYNC2H1,APLP2,UBE2W,NOTCH1,CARD8,H3-2
	GOCC: 0043226	Organelle	0.11	0.0127	FAM76A,ERCC1,COX15,OTUD5,DHRS7,ABCD1,SETD6,GSR,RAB3D,ABHD17A,KRT5,KRT6B,RARA,BRD8,TERF2,MPHOSPH6,PPP2R3C,CLCN3,KMT2C,RHBDF1,DGKD,DNAH5,CHST7,HAUS1,ATP6V0D2,ACSM3,FABP5,IKBIP,KRT16,SDHAF2,PANK1,H2AC8,UBE2E1,RNFT1,RFXANK,GON7,KRT74,ERCC4,KRT2,ATAD5,MPI,ZMYM3,E2F7,NR2F1,TNRC6C,EBAG9,KPTN,ENSA,KLHL7,FYN,POLR3H,UBE2C,SGO2,HSD17B11,WDR55,H3C12,AKR1B10,COL4A2,SCAF1,IBA57,FBXO28,CEP350,SYT11,S100A9,LHPP,RLF,SLC29A3,NDUFA8,SNX30,LPAR1,PBX2,CARNMT1,RGP1,COX7A2L,AGRN,RREB1,KRT6A,CNTLN,PTOV1,FDX2,ORMDL3,ELOVL6,ZNF143,DYNC1I2,HOOK2,CABIN1,CST3,FGFRL1,FKBP1A,METTL15,ZBTB40,PRKAR1B,MAP3K2,RDH13,SATB2,NDRG1,SYBU,PDK2,MACROH2A1,MRPS30,WWC1,RAB15,HSPA8,TNKS1BP1,TDRD3,ATF7IP,GRAMD1A,PRR14,TMEM258,LYRM4,ERH,XRCC3,LDLR,RAD51D,FBLL1,APBB1,NEB,GET1,DYNC2H1,APLP2,NOTCH1,CARD8,H3-2

	GOCC: 0043229	Intracellular organelle	0.12	0.0127	FAM76A,ERCC1,COX15,OTUD5,DHRS7,ABCD1,SETD6,GSR,RAB3D,ABHD17A,KRT5,KRT6B,RARA,BRD8,TERF2,MPHOSPH6,PPP2R3C,CLCN3,KMT2C,RHBDF1,DGKD,DNAH5,CHST7,HAUS1,ATP6V0D2,ACSM3,FABP5,IKBIP,KRT16,SDHAF2,PANK1,H2AC8,UBE2E1,RNFT1,RFXANK,GON7,KRT74,ERCC4,KRT2,ATAD5,ZMYM3,E2F7,NR2F1,TNRC6C,EBAG9,KPTN,ENSA,KLHL7,FYN,POLR3H,UBE2C,SGO2,HSD17B11,WDR55,H3C12,AKR1B10,COL4A2,IBA57,FBXO28,CEP350,SYT11,S100A9,LHPP,RLF,SLC29A3,NDUFA8,SNX30,LPAR1,PBX2,CARNMT1,RGP1,COX7A2L,AGRN,RREB1,KRT6A,CNTLN,FDX2,ORMDL3,ELOVL6,ZNF143,DYNC1I2,HOOK2,CABIN1,CST3,FGFRL1,FKBP1A,METTL15,ZBTB40,MAP3K2,RDH13,SATB2,NDRG1,SYBU,PDK2,MACROH2A1,MRPS30,WWC1,RAB15,HSPA8,TNKS1BP1,TDRD3,ATF7IP,GRAMD1A,PRR14,TMEM258,LYRM4,ERH,XRCC3,LDLR,RAD51D,FBLL1,APBB1,NEB,GET1,DYNC2H1,APLP2,NOTCH1,CARD8,H3-2
	GOCC: 0045095	Keratin filament	1.27	0.0127	KRT5,KRT6B,KRT74,KRT2,KRT6A
	GOCC: 0043231	Intracellular membrane-bounded organelle	0.13	0.0132	FAM76A,ERCC1,COX15,OTUD5,DHRS7,ABCD1,SETD6,GSR,RAB3D,ABHD17A,KRT5,RARA,BRD8,TERF2,MPHOSPH6,CLCN3,KMT2C,RHBDF1,DGKD,CHST7,ATP6V0D2,ACSM3,FABP5,IKBIP,SDHAF2,PANK1,H2AC8,UBE2E1,RNFT1,RFXANK,GON7,ERCC4,ZMYM3,E2F7,NR2F1,EBAG9,KPTN,ENSA,KLHL7,FYN,POLR3H,UBE2C,SGO2,WDR55,H3C12,AKR1B10,COL4A2,IBA57,SYT11,S100A9,LHPP,RLF,SLC29A3,NDUFA8,SNX30,LPAR1,PBX2,CARNMT1,RGP1,COX7A2L,AGRN,RREB1,CNTLN,FDX2,ORMDL3,ELOVL6,ZNF143,HOOK2,CABIN1,CST3,FGFRL1,FKBP1A,METTL15,ZBTB40,MAP3K2,RDH13,SATB2,NDRG1,SYBU,PDK2,MACROH2A1,MRPS30,WWC1,RAB15,HSPA8,TNKS1BP1,TDRD3,ATF7IP,GRAMD1A,PRR14,TME

					M258,LYRM4,ERH,XRCC3,LDLR,RAD51D,FBLL1,APBB1,GET1,DYNC2H1,APLP2,NOTCH1,CARD8,H3-2
	GOCC: 0005737	Cytoplasm	0.12	0.0443	COX15,OTUD5,DHPS,DHRS7,ABCD1,C16orf70,GSR,RAB3D,ARHGEF6,ABHD17A, KRT5,KRT6B,SESN2,RARA,MPHOSPH6,UNC13C,CLCN3,RHBDF1,FARP2,DGKD, DNAH5,HPS5,CHST7,DOCK11,LDHAL6A,ATP6V0D2,ACSM3,FABP5,IKBIP,KRT16, SDHAF2,PANK1,UBE2E1,RNFT1,RFXANK,KRT74,KRT2,TNS3,MPI,NADSYN1,TNRC6C,EBAG9,KPTN,KLHL7,FYN,UBE2C,PBLD,AKR1B10,COL4A2,IBA57,SYT11,S100A9,LHPP,VAV2,SLC29A3,NDUFA8,SNX30,LPAR1,CARNMT1,RGP1,PLCB4, COX7A2L,AGRN,RREB1,KRT6A,CNTLN,SPAG1,FDX2,ORMDL3,ELOVL6, DYNC1I2,HOOK2,CABIN1,CST3,FGFRL1,FKBP1A,METTL15, PRKAR1B,MAP3K2, RDH13,NDRG1,SYBU,DUSP7,PDK2,MRPS30,WWC1,RAB15,HSPA8,TNKS1BP1,GRAMD1A,TMEM258,LYRM4,ERH,XRCC3,LDLR,APBB1,NEB,GET1,DYNC2H1,APLP2,NOTCH1,CARD8
	PF16208	Keratin type II head	1.33	0.0176	KRT5,KRT6B,KRT74,KRT2,KRT6A
SW1353 2 Gy	GO:0031090	Organelle membrane	0.31	0.0078	COX15,AP4S1,C16orf70,RAB3D,TRAF2,TIMM10,FAM8A1,CLCN3,OSBPL5,SNX19, RP9,FABP5,UBXN6,RNFT1,MRPS24,SLC25A36,FZD2,EBAG9,LAMTOR4,P2RX4, SGMS1,SYT11,DPM3,AGPAT2,RGP1,HACD2,PLD3,RDH13,WDR83,SLC25A44,SSR3, GALNT4,SZT2,PLSCR3,SLC44A2,SCOC,ATXN3,APLP2

Table S10. MC615 STRING Network enrichment and associated proteins

	Term ID	Term description	Strength	False discovery rate	Matching proteins in your network (labels)
MC615 0.1 Gy	GO:0005737	Cytoplasm	0.12	0.0014	Sdhd,Grasp,Spa17,Kdelr1,Lipe,Eno2,Traf6,Tmem147,Wdr77,Mmp13,Timp3,Dynll2,Laptm4a,Sdc1,Recql5,Tubal3,Prelid1,Ptdss1,Sdc2,Samm50,Ncbp2,Hbb-bs, Nudt3,C330018D20Rik,Anapc11,Dcxr,Syngr2,Abcb6,Dusp19,Gzf1,Rab5if,Naa15,Lef1,Lamtor2,Gpr89,Ppcs,Calu,Dmpk,Bbs2,Hif1an,Arid4a,Apob,Mboat7,Ggact, Zfand1,Mrps6,Myo1d,Rasa4,Tmem109,Hgsnat,Pdk2,Chst7,Ap1g2,Snx10,Slc9a8, Dtx3,Thbs1,Anln,Myh14,Ostc,Mical2,Btbd10,Sirt6,Ndufv3,Timm8a1,Tk2,Pfn3, Snx21,Map7d1,Uqcr10,Myo3b,Lamtor4,Kif1b,Ppip5k1,Dgkq,Lyrm2,Abi3,Scg2, Slc39a6,Slc30a7,Rab7b,Immt,Opa3,Pstk,Cop1,Zdhhc8,Krt80,Itga1,Mettl6,Pcbp2, Lrp2,Impdh2,mt-Cytb,Tmem87a,March6, Rusc1,Ccnd1,Sec31a,Dhtkd1,Mtch1, Il1rl1,Alg8,Vamp5,Chac2,Tmem97,Mmadhc,Ptges,Jun,Anxa9,Cyth2,Rbis,Myo1c, Rgs19,Zmynd8,Zmiz2,Akt3,Pdcl,Ndufb11,Ube4a,Dsp,Cnih4,Myo5a,Abhd13,Wdr4,Ccnd3,Lrrcc1,Tecpr2,Snrpe,Ghitm,Gnb1,Ppt2,Sumo3,Spcs3,Prrc2c,Map4k4,Fgf2, Myadm,Ppp4c,Nap1l4,Parp16,Atp5md,Magt1
	GO:0005622	Intracellular anatomical structure	0.08	0.0039	Sdhd,Grasp,Spa17,Kdm7a,Kdelr1,Lipe,Eno2,Traf6,Tmem147,Wdr77,Mmp13,Timp3,Dynll2,Twistnb,Laptm4a,Sdc1,Recql5,Tubal3,Prelid1,Ptdss1,Sdc2,Samm50, Rrn3,Ncbp2,Hbb-bs,Nudt3,C330018D20Rik,Anapc11,Dcxr,Syngr2,Abcb6, Dusp19,Gzf1,Rab5if,Naa15, Lef1,Lamtor2,Gpr89,Ppcs,Calu,Dmpk,Brf2, Bbs2,Hif1an,Arid4a,Apob,Mboat7,Grm1,Ggact,Zfand1,Mrps6,Myo1d,Rasa4,Tmem109,Hgsnat,Pdk2,Med30,Chst7,Ap1g2,Snx10,Slc9a8,Dtx3,Thbs1,2410004B18Rik,Anln,Myh14,Ostc,Mical2,Btbd10,Klf16,Sirt6,Ndufv3,Timm8a1,Meaf6,Tk2,Pfn3,

					Snx21,Map7d1,Uqcr10,Myo3b,Lamtor4,Kif1b,Zbtb12,Ppip5k1,Dgkq,Lyrm2,Abi3,H1f1,Scg2,Slc39a6,Slc30a7,Rab7b,Immt,Klf13,Zfp618,Opa3,Pstk,Cop1,Zdhhc8,Krt80,Itga1,Mettl6,Pcbp2,Lrp2,Impdh2,Pop5,mt-Cytb,Radil,Tmem87a,March6,Rusc1,Ccnd1,Sec31a,Dhtkd1,Nr2c2ap,Mtch1,Il1rl1,Alg8,Vamp5,Chac2,Tmem97,Mmadhc,Ptges,Jun,Anxa9,Cyth2,Rbis,Myo1c,Rgs19,Zmynd8,Zmiz2,Asxl1,Zmynd11,Akt3,Zranb3,Pdcl,Ndufb11,Ube4a,Nfyc,Dsp,Cnih4,Prpf40b,Myo5a,Abhd13,Wdr4,Ccnd3,Lrrcc1,Tecpr2,Snrpe,Ghitm,Gnb1,Ppt2,Sumo3,Spcs3,Prrc2c,Map4k4,Fgf2,Myadm,Ppp4c,Nap1l4,Parp16,Polr2f,Atp5md,Magt1
	GO:0043226	Organelle	0.09	0.0039	Sdhd,Grasp,Spa17,Kdm7a,Grin2d,Kdelr1,Lipe,Traf6,Tmem147,Wdr77,Mmp13,Dynll2,Twistnb,Laptm4a,Sdc1,Recql5,Tubal3,Prelid1,Ptdss1,Sdc2,Samm50,Rrn3,Ncbp2,Nudt3,C330018D20Rik,Anapc11,Dcxr,Syngr2,Abcb6,Gzf1,Rab5if,Naa15,Lef1,Lamtor2,Gpr89,Ppcs,Calu,Dmpk,Brf2,Bbs2,Hif1an,Arid4a,Apob,Mboat7,Grm1,Zfand1,Mrps6,Myo1d,Tmem109,Hgsnat,Pdk2,Med30,Chst7,Ap1g2,Snx10,Slc9a8,Dtx3,Thbs1,2410004B18Rik,Anln,Myh14,Ostc,Mical2,Btbd10,Klf16,Sirt6,Ndufv3,Timm8a1,Meaf6,Tk2,Pfn3,Snx21,Map7d1,Uqcr10,Myo3b,Lamtor4,Kif1b,Zbtb12,Dgkq,Lyrm2,Abi3,H1f1,Scg2,Slc39a6,Slc30a7,Rab7b,Immt,Klf13,Zfp618,Opa3,Pstk,Cop1,Zdhhc8,Krt80,Itga1,Mettl6,Pcbp2,Lrp2,Impdh2,Pop5,mt-Cytb,Radil,Tmem87a,March6,Rusc1,Ccnd1,Sec31a,Dhtkd1,Nr2c2ap,Mtch1,Alg8,Vamp5,Tmem97,Mmadhc,Ptges,Jun,Anxa9,Cyth2,Rbis,Myo1c,Rgs19,Zmynd8,Zmiz2,Asxl1,Zmynd11,Akt3,Zranb3,Pdcl,Ndufb11,Ube4a,Nfyc,Dsp,Cnih4,Prpf40b,Myo5a,Wdr4,Ccnd3,Lrrcc1,Snrpe,Ghitm,Gnb1,Ppt2,Sumo3,Spcs3,Prrc2c,Fgf2,Myadm,Ppp4c,Nap1l4,Parp16,Polr2f,Atp5md,Magt1
	GO:0031090	Organelle membrane	0.25	0.0054	Sdhd,Kdelr1,Tmem147,Laptm4a,Ptdss1,Samm50,Syngr2,Abcb6,Rab5if,Lamtor2,Gpr89,Calu,Dmpk,Bbs2,Apob,Mboat7,Mrps6,Tmem109,Hgsnat,Chst7,Ap1g2,Snx10,Slc9a8,Ostc,Ndufv3,Timm8a1,Tk2,Snx21,Uqcr10,Lamtor4,Kif1b,Dgkq,Slc30a7,

					Rab7b,Immt,Cop1,Zdhhc8,mt-Cytb,Tmem87a,March6,Ccnd1,Sec31a,Mtch1,Alg8,Vamp5,Tmem97,Myo1c,Ndufb11,Cnih4,Ghitm,Gnb1,Spcs3,Parp16,Atp5md,Magt1
	GO:0016459	Myosin complex	1.05	0.0112	Dynll2,Myo1d,Myh14,Myo3b,Myo1c,Myo5a
	GO:0043229	Intracellular organelle	0.09	0.0177	Sdhd,Grasp,Kdm7a,Kdelr1,Lipe,Traf6,Tmem147,Wdr77,Mmp13,Dynll2,Twistnb,Laptm4a,Sdc1,Recql5,Tubal3,Prelid1,Ptdss1,Sdc2,Samm50,Rrn3,Ncbp2,Nudt3,C330018D20Rik,Anapc11,Dcxr,Syngr2,Abcb6,Gzf1,Rab5if,Naa15,Lef1,Lamtor2,Gpr89,Ppcs,Calu,Dmpk,Brf2,Bbs2,Hif1an,Arid4a,Apob,Mboat7,Grm1,Zfand1,Mrps6,Myo1d,Tmem109,Hgsnat,Pdk2,Med30,Chst7,Ap1g2,Snx10,Slc9a8,Dtx3,Thbs,2410004B18Rik,Anln,Myh14,Ostc,Mical2,Btbd10,Klf16,Sirt6,Ndufv3, Timm8a1,Meaf6,Tk2,Pfn3,Snx21,Map7d1,Uqcr10,Myo3b,Lamtor4,Kif1b,Zbtb12, Dgkq,Lyrm2,H1f1,Scg2,Slc39a6,Slc30a7,Rab7b,Immt,Klf13,Zfp618,Opa3,Pstk,Cop1,Zdhhc8,Krt80,Itga1,Mettl6,Pcbp2,Lrp2,Impdh2,Pop5,mt-Cytb, Radil,Tmem87a,March6,Rusc1,Ccnd1,Sec31a,Dhtkd1,Nr2c2ap,Mtch1,Alg8,Vamp5,Tmem97,Mmadhc,Ptges,Jun,Anxa9,Rbis,Myo1c,Rgs19,Zmynd8,Zmiz2,Asxl1,Zmynd11,Akt3,Zranb3,Ndufb11,Ube4a,Nfyc,Dsp,Cnih4,Prpf40b,Myo5a,Wdr4,Ccnd3,Lrrcc1,Snrpe,Ghitm,Ppt2,Sumo3,Spcs3,Prrc2c,Fgf2,Myadm,Ppp4c,Nap1l4,Parp16,Polr2f,Atp5md,Magt1
	GO:1902494	Catalytic complex	0.33	0.0177	Sdhd,Eno2,Wdr77,Dynll2,Twistnb,Recql5,Anapc11,Naa15,Arid4a,Pdk2,Med30,Ostc,Ndufv3,Meaf6,Uqcr10,Cop1,Pop5,mt-Cytb,Ccnd1,Myo1c,Ndufb11,Ube4a,Wdr4,Ccnd3, Snrpe,Gnb1,Sumo3,Spcs3,Ppp4c,Polr2f,Magt1
	GO:0098798	Mitochondrial protein-	0.61	0.0319	Sdhd,Samm50,Mrps6,Pdk2,Ndufv3,Timm8a1,Uqcr10,Immt,mt-Cytb,Ndufb11,Atp5md

		containing complex			
	GO:0043227	Membrane-bounded organelle	0.09	0.0426	Sdhd,Grasp,Spa17,Kdm7a,Kdelr1,Lipe,Traf6,Tmem147,Wdr77,Mmp13,Dynll2,Twistnb,Laptm4a,Sdc1,Recql5,Prelid1,Ptdss1,Sdc2,Samm50,Rrn3,Ncbp2,Nudt3, C330018D20Rik,Anapc11,Syngr2,Abcb6,Gzf1,Rab5if,Naa15,Lef1,Lamtor2, Gpr89,Ppcs,Calu,Dmpk,Brf2,Bbs2,Hif1an,Arid4a,Apob,Mboat7,Grm1,Mrps6,Myo1d,Tmem109,Hgsnat,Pdk2,Med30,Chst7,Ap1g2,Snx10,Slc9a8,Dtx3,Thbs1,2410004B18Rik,Anln,Ostc,Mical2,Btbd10,Klf16,Sirt6,Ndufv3,Timm8a1,Meaf6, Tk2,Pfn3,Snx21,Uqcr10,Myo3b,Lamtor4,Kif1b,Zbtb12,Dgkq,Lyrm2,H1f1, Scg2,Slc39a6,Slc30a7,Rab7b,Immt,Klf13,Zfp618,Opa3,Pstk,Cop1,Zdhhc8,Itga1,Mettl6,Pcbp2,Lrp2,Impdh2,Pop5,mt-Cytb,Tmem87a,March6,Rusc1,Ccnd1, Sec31a,Dhtkd1,Nr2c2ap,Mtch1,Alg8,Vamp5,Tmem97,Mmadhc,Ptges,Jun,Anxa9, Rbis,Myo1c,Rgs19,Zmynd8,Zmiz2,Asxl1,Zmynd11,Akt3,Zranb3,Pdcl,Ndufb11,Ube4a,Nfyc,Cnih4,Prpf40b,Myo5a,Wdr4,Ccnd3,Snrpe,Ghitm,Gnb1,Ppt2,Sumo3,Spcs3,Fgf2,Ppp4c,Nap1l4,Parp16,Polr2f,Atp5md,Magt1
	GO:0005746	Mitochondrial respirasome	0.86	0.05	Sdhd,Rab5if,Ndufv3,Uqcr10,mt-Cytb,Ndufb11
	BTO: 0000000	Tissues, cell types and enzyme sources	0.14	0.007	Sdhd,Grasp,Spa17,Kdm7a,Grin2d,Kdelr1,Lipe,Eno2,Tmem147,Mmp13,Timp3,Dynll2,Laptm4a,Sdc1,Recql5,Prelid1,Ptdss1,Sdc2,Samm50,Ncbp2,Hbb-bs,Nudt3, Gzf1,Naa15, Sema3a,Calu,Dmpk,Sema4b,Bbs2,Hif1an,Apob,Mboat7,Grm1, Zfand1,Myo1d,Rasa4,Hgsnat,Pdk2,Med30,Ap1g2,Snx10,Dtx3,Thbs1,Anln,Myh14,Ostc,Dap,Mical2,Gipc3,Timm8a1,Meaf6,Pfn3,Map7d1,Lamtor4,Kif1b,Ppip5k1, Abi3,H1f1,Scg2,Slc39a6,Slc30a7,Rab7b,Immt,Klf13,Opa3,Zdhhc8,Mettl6,Pcbp2,Ubl3,4921509C19Rik,Lrp2,Impdh2,Pop5,Mug2,mt-Cytb,Radil,March6,Ccnd1,

					Sec31a,Plekhg2,Dhtkd1,Mtch1,Alg8,Vamp5,Tmem97, Ptges,Jun,Cyth2,Myo1c, Rgs19,Asxl1,Akt3,Pdcl,Timp1,Nfyc,Dsp,Myo5a,Ccnd3,Lrrcc1,Snrpe,Ghitm,Gnb1, Ppt2,Slc29a1,Sumo3,Spcs3,Prrc2c,Map4k4,Fgf2,Myadm,Ppp4c,Nap1l4,Parp16, Atp5md,Magt1
	BTO: 0001489	Whole body	0.14	0.007	Sdhd,Grasp,Spa17,Kdm7a,Grin2d,Kdelr1,Lipe,Eno2,Tmem147,Mmp13,Timp3,Dynll2,Laptm4a,Sdc1,Recql5,Prelid1,Ptdss1,Sdc2,Samm50,Ncbp2,Hbb-bs,Nudt3, Gzf1,Naa15, Sema3a,Calu,Dmpk,Sema4b,Bbs2,Hif1an,Apob,Mboat7,Grm1, Zfand1,Myo1d,Rasa4,Hgsnat,Pdk2,Med30,Ap1g2,Snx10,Dtx3,Thbs1,Anln,Myh14,Ostc,Dap,Mical2,Gipc3,Timm8a1,Meaf6,Pfn3,Map7d1,Lamtor4,Kif1b,Ppip5k1, Abi3,H1f1,Scg2,Slc39a6,Slc30a7,Rab7b,Immt,Klf13,Opa3,Zdhhc8,Mettl6,Pcbp2,Ubl3,4921509C19Rik,Lrp2,Impdh2,Pop5,Mug2,mt-Cytb,Radil,March6,Ccnd1, Sec31a,Dhtkd1,Mtch1,Alg8,Vamp5,Tmem97,Ptges, Jun,Cyth2,Myo1c,Rgs19, Asxl1,Akt3,Pdcl,Timp1,Nfyc,Dsp,Myo5a,Ccnd3,Lrrcc1,Snrpe,Ghitm,Gnb1,Ppt2,Slc29a1,Sumo3,Spcs3,Prrc2c,Map4k4,Fgf2,Myadm,Ppp4c,Nap1l4,Parp16, Atp5md,Magt1
	BTO: 0001491	Viscus	0.25	0.007	Sdhd,Lipe,Eno2,Tmem147,Dynll2,Laptm4a,Sdc1,Prelid1,Ptdss1,Samm50,Ncbp2, Hbb-bs,Nudt3,Sema4b,Mboat7,Myo1d,Rasa4,Hgsnat,Pdk2,Med30,Thbs1, Myh14,Ostc,Dap,Lamtor4,Kif1b,Abi3,Scg2,Slc39a6,Slc30a7,Klf13,Ubl3,Lrp2, Mug2,mt-Cytb,Radil,Sec31a, Dhtkd1,Mtch1,Tmem97,Ptges,Jun,Rgs19,Asxl1, Pdcl,Timp1,Nfyc,Dsp,Myo5a,Ccnd3,Snrpe,Ghitm,Gnb1,Ppt2,Slc29a1,Sumo3,Spcs3,Myadm,Ppp4c,Parp16,Atp5md,Magt1
	BTO: 0000522	Gland	0.18	0.0413	Sdhd,Spa17,Lipe,Eno2,Tmem147,Dynll2,Laptm4a,Sdc1,Prelid1,Ptdss1,Samm50, Ncbp2,Hbb-bs,Nudt3,Naa15,Sema4b,Bbs2,Mboat7,Zfand1,Myo1d,Hgsnat, Pdk2,Med30, Dtx3,Thbs1,Anln,Ostc,Dap,Timm8a1,Meaf6,Pfn3,Lamtor4,Kif1b,

					Scg2,Slc39a6,Slc30a7,Klf13,Ubl3,4921509C19Rik,Lrp2,Mug2,mt-Cytb,Radil, Ccnd1,Sec31a,Dhtkd1,Mtch1, Alg8,Tmem97,Jun,Asxl1,Pdcl,Timp1,Dsp,Myo5a,Ccnd3,Lrrcc1,Snrpe,Ghitm,Gnb, Ppt2,Slc29a1,Sumo3,Spcs3,Prrc2c,Myadm,Ppp4c,Parp16,Atp5md,Magt1
	BTO: 0000988	Pancreas	0.47	0.0413	Tmem147,Sdc1,Ptdss1,Nudt3,Sema4b,Pdk2,Ostc,Scg2,Slc30a7,Radil,Tmem97, Myo5a,Ccnd3,Spcs3,Ppp4c,Parp16,Magt1
	BTO: 0000345	Digestive gland	0.28	0.0436	Lipe,Tmem147,Dynll2,Sdc1,Ptdss1,Hbb-bs,Nudt3,Sema4b,Mboat7,Pdk2,Thbs1, Ostc,Dap,Lamtor4,Kif1b,Scg2,Slc30a7,Mug2,mt-Cytb,Radil,Sec31a,Dhtkd1, Tmem97, Timp1,Dsp,Myo5a,Ccnd3,Ghitm,Slc29a1,Sumo3,Spcs3,Ppp4c,Parp16, Atp5md,Magt1
	GOCC: 0005622	Intracellular	0.15	1.68E-06	Sdhd,Grasp,Spa17,Kdm7a,Kdelr1,Lipe,Eno2,Traf6,Wdr77,Mmp13,Dynll2,Twistnb ,Laptm4a,Sdc1,Recql5,Tubal3,Prelid1,Ptdss1,Sdc2,Samm50,Rrn3,Ncbp2,Hbb-bs,Nudt3, C330018D20Rik,Anapc11,Dcxr,Syngr2,Abcb6,Dusp19,Gzf1,Rab5if, Naa15,Lef1,Lamtor2,Gpr89,Ppcs,Calu,Dmpk,Bbs2,Hif1an,Arid4a,Apob,Grm1, Ggact,Zfand1,Mrps6,Myo1d,Rasa4,Tmem109,Hgsnat,Pdk2,Med30,Ap1g2,Snx10, Thbs1,Anln,Myh14,Ostc,Mical2,Btbd10,Klf16,Sirt6,Ndufv3,Timm8a1,Meaf6,Tk2, Snx21,Uqcr10,Myo3b,Lamtor4,Kif1b,Zbtb12,Ppip5k1,Dgkq,Lyrm2,Abi3,H1f1, Scg2,Slc39a6,Slc30a7,Rab7b,Immt,Klf13,Zfp618,Opa3,Cop1,Zdhhc8,Krt80,Mettl6 ,Pcbp2,Lrp2,Pop5,mt-Cytb,Tmem87a,March6,Rusc1, Ccnd1,Sec31a,Dhtkd1, Vamp5,Tmem97,Mmadhc,Ptges,Jun,Anxa9,Cyth2,Myo1c,Rgs19,Zmynd8,Zmiz2,Asxl1,Zmynd11,Akt3,Zranb3,Pdcl,Ndufb11,Ube4a,Nfyc,Cnih4,Prpf40b,Myo5a,Abhd13,Zbtb8os,Wdr4,Ccnd3,Lrrcc1,Snrpe,Ghitm,Gnb1,Ppt2,Sumo3,Spcs3,Prrc2c,Map4k4,Fgf2,Myadm,Ppp4c,Nap1l4,Parp16,Polr2f,Atp5md,Magt1

	GOCC: 0043231	Intracellular membrane-bounded organelle	0.19	5.90E-05	Sdhd,Grasp,Kdm7a,Kdelr1,Lipe,Traf6,Mmp13,Twistnb,Laptm4a,Sdc1,Recql5,Prelid1,Ptdss1,Sdc2,Samm50,Rrn3,C330018D20Rik,Anapc11,Syngr2,Abcb6,Gzf1,Rab5if,Lef1,Lamtor2,Gpr89,Calu,Dmpk,Arid4a,Apob,Grm1,Mrps6,Myo1d,Tmem109,Hgsnat,Pdk2,Med30,Ap1g2,Snx10,Thbs1,Anln,Ostc,Mical2,Btbd10,Klf16,Sirt6,Ndufv3,Timm8a1,Meaf6,Tk2,Snx21,Uqcr10,Lamtor4,Kif1b,Zbtb12,Dgkq,Lyrm2,H1f1,Scg2,Slc39a6,Slc30a7,Rab7b,Immt,Klf13,Opa3,Cop1,Zdhhc8,Pcbp2,Lrp2,Pop5,mt-Cytb,Tmem87a,March6,Rusc1, Ccnd1,Sec31a,Dhtkd1,Vamp5,Tmem97,Mmadhc,Ptges,Jun,Myo1c,Zmynd8,Zmiz2,Asxl1,Zmynd11,Zranb3,Ndufb11,Nfyc,Cnih4,Prpf40b,Myo5a,Ccnd3,Snrpe,Ghitm,Ppt2,Sumo3,Spcs3,Fgf2,Ppp4c,Nap1l4,Parp16,Polr2f,Atp5md,Magt1
	GOCC: 0043226	Organelle	0.16	6.94E-05	Sdhd,Grasp,Spa17,Kdm7a,Grin2d,Kdelr1,Lipe,Traf6,Mmp13,Dynll2,Twistnb,Laptm4a,Sdc1,Recql5,Tubal3,Prelid1,Ptdss1,Sdc2,Samm50,Rrn3,C330018D20Rik,Anapc11,Dcxr,Syngr2,Abcb6,Gzf1,Rab5if,Lef1,Lamtor2,Gpr89,Calu,Dmpk,Bbs2,Arid4a,Apob,Mboat7,Grm1,Ggact,Zfand1,Mrps6,Myo1d,Tmem109,Hgsnat,Pdk2,Med30,Ap1g2,Snx10,Thbs1,Anln,Myh14,Ostc,Mical2,Btbd10,Klf16,Sirt6,Ndufv3,Timm8a1,Meaf6,Tk2,Snx21,Uqcr10,Myo3b,Lamtor4,Kif1b,Zbtb12,Dgkq,Lyrm2,H1f1,Scg2,Slc39a6,Slc30a7,Rab7b,Immt,Klf13,Zfp618,Opa3,Cop1,Zdhhc8,Krt80,Pcbp2,Lrp2,Pop5,mt-Cytb,Tmem87a,March6, Rusc1,Ccnd1,Sec31a,Dhtkd1,Vamp5,Tmem97,Mmadhc,Ptges,Jun,Cyth2,Myo1c,Rgs19,Zmynd8,Zmiz2,Asxl1,Zmynd11,Zranb3,Ndufb11,Nfyc,Cnih4,Prpf40b,Myo5a,Ccnd3,Lrrcc1,Snrpe,Ghitm,Gnb1,Ppt2,Sumo3,Spcs3,Fgf2,Myadm,Ppp4c,Nap1l4,Parp16,Polr2f,Atp5md,Magt1

	GOCC: 0043227	Membrane-bounded organelle	0.17	0.00011	Sdhd,Grasp,Spa17,Kdm7a,Kdelr1,Lipe,Traf6,Mmp13,Twistnb,Laptm4a,Sdc1,Recql5,Prelid1,Ptdss1,Sdc2,Samm50,Rrn3,C330018D20Rik,Anapc11,Syngr2,Abcb6,Gzf1,Rab5if,Lef1,Lamtor2,Gpr89,Calu,Dmpk,Bbs2,Arid4a,Apob,Grm1,Mrps6,Myo1d,Tmem109,Hgsnat,Pdk2,Med30,Ap1g2,Snx10,Thbs1,Anln,Ostc,Mical2,Btbd10,Klf16,Sirt6,Ndufv3,Timm8a1,Meaf6,Tk2,Snx21,Uqcr10,Myo3b,Lamtor4,Kif1b,Zbtb12,Dgkq,Lyrm2,H1f1,Scg2,Slc39a6,Slc30a7,Rab7b,Immt,Klf13,Opa3,Cop1,Zdhhc8,Pcbp2,Lrp2,Pop5,mt-Cytb,Tmem87a, March6,Rusc1,Ccnd1,Sec31a,Dhtkd1,Vamp5,Tmem97,Mmadhc,Ptges,Jun,Myo1c,Rgs19,Zmynd8,Zmiz2,Asxl1,Zmynd11,Zranb3,Ndufb11,Nfyc,Cnih4,Prpf40b,Myo5a,Ccnd3,Snrpe,Ghitm,Gnb1,Ppt2,Sumo3,Spcs3,Fgf2,Ppp4c,Nap1l4,Parp16,Polr2f,Atp5md,Magt1
	GOCC: 0043229	Intracellular organelle	0.16	0.00011	Sdhd,Grasp,Kdm7a,Kdelr1,Lipe,Traf6,Mmp13,Dynll2,Twistnb,Laptm4a,Sdc1,Recql5,Tubal3,Prelid1,Ptdss1,Sdc2,Samm50,Rrn3,C330018D20Rik,Anapc11,Dcxr,Syngr2,Abcb6,Gzf1,Rab5if,Lef1,Lamtor2,Gpr89,Calu,Dmpk,Bbs2,Arid4a,Apob,Grm1,Ggact,Zfand1,Mrps6,Myo1d,Tmem109,Hgsnat,Pdk2,Med30,Ap1g2,Snx10,Thbs1,Anln,Myh14,Ostc,Mical2,Btbd10,Klf16,Sirt6,Ndufv3,Timm8a1,Meaf6,Tk2,Snx21,Uqcr10,Myo3b,Lamtor4,Kif1b,Zbtb12,Dgkq,Lyrm2,H1f1,Scg2,Slc39a6,Slc30a7,Rab7b,Immt,Klf13,Zfp618,Opa3,Cop1,Zdhhc8,Krt80,Pcbp2,Lrp2,Pop5,mt-Cytb,Tmem87a,March6,Rusc1,Ccnd1,Sec31a,Dhtkd1, Vamp5,Tmem97,Mmadhc,Ptges,Jun,Myo1c,Rgs19,Zmynd8,Zmiz2,Asxl1,Zmynd11,Zranb3,Ndufb11,Nfyc,Cnih4,Prpf40b,Myo5a,Ccnd3,Lrrcc1,Snrpe,Ghitm,Ppt2,Sumo3,Spcs3,Fgf2,Myadm,Ppp4c,Nap1l4,Parp16,Polr2f,Atp5md,Magt1
	GOCC: 0110165	Cellular anatomical entity	0.1	0.00035	Sdhd,Grasp,Spa17,Kdm7a,Grin2d,Kdelr1,Lipe,Eno2,Traf6,Tmem147,Wdr77,Mmp13,Timp3,Dynll2,Twistnb,Laptm4a,Sdc1,Recql5,Tubal3,Prelid1,Ptdss1,Sdc2,Samm50,Rrn3,Hbb-bs,Celsr3,C330018D20Rik,Anapc11,Dcxr,Syngr2,Abcb6,Lamc1,Dusp19,Gzf1,Rab5if, Naa15,Lef1,Lamtor2,Gpr89,Ppcs,Sema3a,Calu,

					Dmpk,Sema4b,Bbs2,Arid4a,Apob,Mboat7,Grm1,Ggact,Zfand1,Mrps6,Myo1d,Rasa4,Tmem109,Hgsnat,Pdk2,Med30,Ap1g2,Snx10,Dtx3,Thbs1,Anln,Myh14,Ostc,Mical2,Btbd10,Klf16,Sirt6,Ndufv3,Timm8a1,Meaf6,Tk2,Snx21,Uqcr10,Myo3b,Lamtor4,Kif1b,Zbtb12,Dgkq,Lyrm2,Abi3,H1f1,Scg2,Slc39a6,Slc30a7,Rab7b,Immt,Klf13,Zfp618,C4b,Opa3,Cop1,Zdhhc8,Krt80,Itga1,Mettl6,Pcbp2,Lrp2,Pop5,Mug2,mt-Cytb,Tmem87a,March6,Rusc1,Ccnd1,Sec31a,Plekhg2,Dhtkd1,Il1rl1, Vamp5, Tmem97,Rtn4rl1,Mmadhc,Ptges,Jun,Anxa9,Cyth2,Myo1c,Rgs19,Zmynd8,Zmiz2, Asxl1,Zmynd11,Akt3,Zranb3,Pdcl,Timp1,Ndufb11,Ube4a,Nfyc,Dsp,Cnih4,Prpf40b,Myo5a,Abhd13,Ccnd3,Lrrcc1,Snrpe,Ghitm,Gnb1,Ppt2,Slc29a1,Sumo3,Spcs3,Map4k4,Fgf2,Myadm,Ppp4c,Nap1l4,Parp16,Polr2f,Atp5md,Magt1
	GOCC: 0005737	Cytoplasm	0.17	0.00061	Sdhd,Spa17,Kdelr1,Lipe,Eno2,Traf6,Wdr77,Mmp13,Laptm4a,Sdc1,Prelid1, Ptdss1,Sdc2,Samm50,Hbb-bs,C330018D20Rik,Dcxr,Syngr2,Abcb6,Dusp19, Rab5if,Naa15,Lamtor2, Gpr89,Ppcs,Calu,Dmpk,Apob,Zfand1,Mrps6,Myo1d, Rasa4,Hgsnat,Pdk2,Ap1g2,Snx10,Thbs1,Anln,Myh14,Ostc,Mical2,Btbd10, Sirt6,Ndufv3,Timm8a1,Tk2,Snx21,Uqcr10,Myo3b,Lamtor4,Kif1b,Dgkq,Lyrm2, Abi3,Scg2,Slc39a6,Slc30a7,Rab7b,Immt,Opa3,Cop1,Zdhhc8,Mettl6,Pcbp2,Lrp2, mt-Cytb,Tmem87a,March6,Rusc1,Ccnd1,Sec31a,Dhtkd1,Vamp5, Tmem97, Mmadhc,Ptges,Jun,Anxa9,Cyth2,Myo1c,Rgs19,Akt3,Pdcl,Ndufb11,Ube4a,Cnih4, Myo5a,Abhd13,Ccnd3,Snrpe,Ghitm,Ppt2,Spcs3,Fgf2,Myadm,Ppp4c,Parp16, Atp5md,Magt1
	GOCC: 0031090	Organelle membrane	0.33	0.0018	Sdhd,Kdelr1,Laptm4a,Ptdss1,Samm50,Syngr2,Abcb6,Rab5if,Lamtor2,Gpr89, Calu,Dmpk,Bbs2,Apob,Tmem109,Hgsnat,Snx10,Ostc,Ndufv3,Snx21,Uqcr10,Lamtor4,Kif1b,Slc30a7,Immt,Cop1,mt-Cytb,Tmem87a,March6,Sec31a,Tmem97, Myo1c,Ndufb11,Ghitm,Gnb1,Spcs3,Atp5md,Magt1

	GOCC: 1902494	Catalytic complex	0.34	0.0041	Sdhd,Eno2,Wdr77,Dynll2,Twistnb,Sdc1,Recql5,Anapc11,Naa15,Ppcs,Arid4a,Pdk2,Anln,Ostc,Ndufv3,Meaf6,Uqcr10,Pstk,Cop1,Pop5,mt-Cytb,Ccnd1,Myo1c,Asxl1, Ndufb11, Wdr4,Ccnd3,Snrpe,Gnb1,Sumo3,Spcs3,Ppp4c,Polr2f,Magt1
	GOCC: 0016459	Myosin complex	1.05	0.0055	Dynll2,Myo1d,Myh14,Myo3b,Myo1c,Myo5a
	GOCC: 0005739	Mitochondrion	0.35	0.024	Sdhd,Lipe,Prelid1,Samm50,C330018D20Rik,Abcb6,Rab5if,Dmpk,Mrps6,Pdk2, Anln,Ndufv3,Timm8a1,Tk2,Uqcr10,Kif1b,Lyrm2,Immt,Opa3,Zdhhc8,mt-Cytb, Dhtkd1,Mmadhc, Ndufb11,Ghitm,Atp5md
	GOCC: 0005746	Mitochondrial respirasome	0.91	0.0245	Sdhd,Rab5if,Ndufv3,Uqcr10,mt-Cytb,Ndufb11
	GOCC: 0098798	Mitochondrial protein complex	0.6	0.0257	Sdhd,Samm50,Mrps6,Pdk2,Ndufv3,Timm8a1,Uqcr10,Immt,mt-Cytb, Ndufb11,Atp5md
	GOCC: 0016461	Unconventional myosin complex	1.18	0.0345	Dynll2,Myo3b,Myo1c,Myo5a
	KW-0813	Transport	0.31	0.0161	Sdhd,Grin2d,Kdelr1,Dynll2,Laptm4a,Prelid1,Ncbp2,Hbb-bs,C330018D20Rik, Abcb6, Gpr89,Bbs2,Apob,Myo1d,Tmem109,Ap1g2,Snx10,Slc9a8,Ndufv3, Timm8a1,Snx21,Uqcr10,Slc36a4,Slc39a6,Slc30a7,Rab7b,Lrp2,mt-Cytb,Sec31a, Mtch1,Ndufb11,Cnih4,Myo5a,Mfsd14b,Slc29a1,Magt1
	KW-0007	Acetylation	0.24	0.0191	Eno2,Laptm4a,Ptdss1,Ncbp2,Hbb-bs,Nudt3,Dcxr,Syngr2,Dusp19,Naa15,Ppcs, Calu, Hif1an,Apob,Myo1d,Med30,Anln,Myh14,Dap,Sirt6,Meaf6,

					Lamtor4,Kif1b,Slc36a4,H1f1,Immt,Zfp618,Impdh2,March6,Mmadhc,Jun,Rbis,Myo1c,Akt3,Pdcl,Ube4a,Prpf40b,Myo5a,Zbtb8os,Wdr4,Gnb1,Fam177a,Prrc2c,Map4k4,Ppp4c,Nap1l4,Wdcp,Polr2f,Atp5md
	KW-0518	Myosin	1.05	0.0233	Myo1d,Myh14,Myo3b,Myo1c,Myo5a
	KW-0597	Phosphoprotein	0.14	0.0233	Grasp,Kdm7a,Grin2d,Kdelr1,Lipe,Eno2,Wdr77,Mmp13,2310033P09Rik,Twistnb,Sdc1,Recql5,Ptdss1,Sdc2,Rrn3,Ncbp2,Hbb-bs,Celsr3,Syngr2,Lamc1,Gzf1,Naa15,Lef1,Calu, Dmpk,Sema4b,Brf2,Arid4a,Apob,Grm1,Myo1d,Hgsnat,Pdk2,Chst7,Slc9a8,2410004B18Rik,Anln,Myh14,Dap,Mical2,9530068E07Rik,Klf16,Sirt6,Ndufv3,Timm8a1,Meaf6,Map7d1,Kif1b,Ppip5k1,Dgkq,Abi3,H1f1,Scg2,Slc39a6,Rab7b,Immt,Klf13,Zdhhc8,Krt80,Pcbp2,Lrp2,Impdh2,Pop5,Radil,Tmem87a,Rusc1,Ccnd1,Sec31a,Plekhg2,Vamp5,Jun,Rbis,Myo1c,Rgs19,Asxl1,Zmynd11,Akt3,Mpzl1,Pdcl,Timp1,Dsp,Prpf40b,Myo5a,Wdr4,Ccnd3,Gnb1,Slc29a1,Fam177a,Fam193a,Prrc2c,Map4k4,Fgf2,Nap1l4,Wdcp,Polr2f
	KW-0505	Motor protein	0.78	0.0257	Dynll2,Myo1d,Myh14,Myo3b,Kif1b,Myo1c,Myo5a
	KW-0999	Mitochondrion inner membrane	0.58	0.0363	Sdhd,Rab5if,Ndufv3,Timm8a1,Uqcr10,Immt,mt-Cytb,Mtch1,Ndufb11,Ghitm
	KW-0249	Electron transport	0.79	0.0431	Sdhd,C330018D20Rik,Ndufv3,Uqcr10,mt-Cytb,Ndufb11
	SM00242	Myosin. Large ATPases.	1.15	0.0386	Myo1d,Myh14,Myo3b,Myo1c,Myo5a
	GO:0043231	Intracellular membrane-	0.14	0.0131	Grasp,Serpinf1,Ifi211,Abca8b,Itm2b,Sdc2,Tmbim6,Nme4,Dcp2,Banf1,Zfhx4,Plk4,Rab5if,Lamtor2,Aff1,Ate1,Rp2,Fadd,Hif1an,Grm1,Myo1d,Ythdc1,Cdk19,

		bounded organelle			Spag9,Slc9a8,Vasn,2410004B18Rik,Ostc,Cpe,Sirt6,Utp14b,Rnf7,Meaf6,Uqcr10,Slc25a44,A4galt,Tmed3,Maml1,Scg2,Klf13,Klhdc10,Brsk2,Pstk,Fnip2,4930453N24Rik,Mettl6,Lrp2,Cenpm,Tmem87a,Rusc1,Kxd1,Chchd2,Entpd6,Sec31a,Dhtkd1,Jund,Alg8,Vamp5,Rhbdf2,Tmem97,Mmadhc,Rbis,Tmem87b,Zmynd11,Sh3bgrl2,Ei24,Cep164,Cnih4,Myo5a,Repin1,Pip4p1,Cpsf4,Ccnd3,Lsm5,Ms4a2,Hivep2,Parp16,Cd63,Gpr108
MC615 0.5 Gy	GO:0043227	Membrane-bounded organelle	0.13	0.0155	Grasp,Serpinf1,Ifi211,Abca8b,Itm2b,Sdc2,Tmbim6,Nme4,Dcp2,Banf1,Zfhx4,Plk4,Rab5if,Lamtor2,Aff1,Ate1,Rp2,Fadd,Hif1an,Grm1,Myo1d,Ythdc1,Cdk19,Spag9,Ttll7,Slc9a8,Vasn,2410004B18Rik,Ostc,Cpe,Sirt6,Utp14b,Rnf7,Meaf6,Uqcr10,Slc25a44,A4galt,Tmed3,Maml1,Scg2,Klf13,Klhdc10,Brsk2,Pstk,Fnip2,4930453N24Rik,Mettl6,Lrp2,Cenpm,Tmem87a,Rusc1,Kxd1,Chchd2,Entpd6,Sec31a,Dhtkd1,Jund,Alg8,Vamp5,Rhbdf2,Tmem97,Mmadhc,Rbis,Tmem87b,Zmynd11,Sh3bgrl2,Ei24,Cep164,Cnih4,Myo5a,Repin1,Pip4p1,Cpsf4,Ccnd3,Lsm5,Ms4a2,Hivep2,Parp16,Cd63,Gpr108
	GO:0043229	Intracellular organelle	0.12	0.0155	Grasp,Serpinf1,Ifi211,Abca8b,Itm2b,Sdc2,Tmbim6,Nme4,Dcp2,Banf1,Zfhx4,Plk4,Rab5if,Lamtor2,Aff1,Ate1,Rp2,Fadd,Hif1an,Grm1,Myo1d,Ythdc1,Cdk19,Spag9,Ttll7,Slc9a8,Vasn,2410004B18Rik,Ostc,Cpe,Sirt6,Utp14b,Rnf7,Meaf6,Map7d1,Uqcr10,Slc25a44,A4galt,Tmed3,Maml1,Scg2,Klf13,Klhdc10,Brsk2,Pstk,Fnip2,4930453N24Rik,Mettl6,Lrp2,Cenpm,Tmem87a,Rusc1,Kxd1,Chchd2,Entpd6,Sec31a,Dhtkd1,Jund,Alg8,Vamp5,Rhbdf2,Tmem97,Mmadhc,Rbis,Tmem87b,Ripor2,Zmynd11,Sh3bgrl2,Ei24,Cep164,Dsp,Cnih4,Myo5a,Repin1,Pip4p1,Cpsf4,Ccnd3,Lsm5,Ms4a2,Hivep2,Parp16,Cd63,Gpr108
	GOCC:	Intracellular membrane-	0.22	0.0088	Grasp,Ifi211,Sdc2,Tmbim6,Nme4,Dcp2,Banf1,Zfhx4,Plk4,Rab5if,Lamtor2,Aff1,Ate1,Rp2,Fadd,Grm1,Myo1d,Ythdc1,Cdk19,Spag9,Vasn,Ostc,Cpe,Sirt6,Utp14b,

	0043231	bounded organelle			Rnf7,Meaf6,Uqcr10,Slc25a44,Tmed3,Maml1,Scg2,Klf13,Lrp2,Tmem87a,Rusc1, Chchd2,Entpd6,Sec31a,Dhtkd1,Jund,Vamp5,Rhbdf2,Tmem97, Mmadhc, Zmynd11,Cep164,Cnih4,Myo5a,Repin1,Pip4p1,Cpsf4,Ccnd3,Lsm5,Hivep2, Parp16,Cd63,Gpr108
	GOCC: 0043227	Membrane-bounded organelle	0.19	0.0197	Grasp,Ifi211,Itm2b,Sdc2,Tmbim6,Nme4,Dcp2,Banf1,Zfhx4,Plk4,Rab5if,Lamtor2,Aff1,Ate1,Rp2,Fadd,Grm1,Myo1d,Ythdc1,Cdk19,Spag9,Ttll7,Vasn,Ostc,Cpe,Sirt6,Utp14b,Rnf7,Meaf6,Uqcr10,Slc25a44,Tmed3,Maml1,Scg2,Klf13,Lrp2,Tmem87a,Rusc1,Chchd2,Entpd6,Sec31a,Dhtkd1,Jund,Vamp5,Rhbdf2,Tmem97, Mmadhc,Zmynd11,Cep164,Cnih4,Myo5a,Repin1,Pip4p1,Cpsf4,Ccnd3,Lsm5, Hivep2,Parp16,Cd63,Gpr108
	GOCC: 0110165	Cellular anatomical entity	0.12	0.0209	Grasp,Serpinf1,Ifi211,Lims1,Abca8b,Itm2b,Sdc2,Tmbim6,Twsg1,Nme4,Dcp2, Banf1,Zfhx4,Plk4,Lamc1,Zmynd19,Rab5if,Lamtor2,Aff1,Ate1,Rp2,Fadd,Ninj1, Slc38a7,Grm1,Myo1d,Frem3,Ythdc1,Cdk19,Spag9,Ttll7,Slco4a1,Vasn,Ostc,Cpe,Sirt6,Utp14b,Rnf7,Meaf6,Uqcr10,Slc25a44,Tmed3,Maml1,Scg2,Klf13,C4b,Nectin2, Brsk2,Fnip2,Mettl6,Lrp2,Tmem87a,Rusc1,Kxd1,Chchd2,Entpd6,Sec31a, Dhtkd1,Jund,Vamp5,Rhbdf2,Tmem97,Rtn4rl1,Mmadhc,Ripor2,Zmynd11,Tspan9, Timp1,Cep164,Dsp,Cnih4,Myo5a,Repin1,Slc26a2,Pip4p1,Cpsf4,Ccnd3,Lsm5, Ms4a2,Hivep2,Parp16,Cd63,Gpr108
	PF06814	Lung seven transmembrane receptor	2.2	0.0081	Tmem87a,Tmem87b,Gpr108
	IPR009637	Transmembrane protein	2.2	0.0252	Tmem87a,Tmem87b,Gpr108

		GPR107/GPR108-like			
MC615 2 Gy	GO:0005622	Intracellular anatomical structure	14759	0.11	Sdhd,Grk6,Gstt3,Tmem39a,Eno2,Smpd4,Elk3,Ifi211,Wdr77,Tubb1,Ndufa1,Ccdc12,Fbxo5,Recql5,Pimreg,Actn1,Tubal3,Ptdss1,Anxa8,Sub1,Samm50,Zfp263,Ncbp2,Cfap298,Nudt3,Diaph1,Bad,Dcxr,Hras,Syngr2,Plk4,Coq10b,Nmi,Zmynd19,Rab5if,Naa15,Ssr3,Ints12,Lamtor2,Calu,Gabarapl1,Lhpp,Slc10a7,Rp9,Snx1,Nudt16,Hif1an,Mboat7,Fkbp1a,Ggact,Mrps6,Foxred1,Ythdc1,Tmem109,Hgsnat,Cdk19,Pdk2,Med30,Snx10,Dtx3,2410004B18Rik,Secisbp2,H1f2,Myh14,Ostc,Sirt6,Timm8a1,Cep112,D1Ertd622e,Utp14b,Nfib,Meaf6,Tk2,Dpy19l3,Uqcr10,Myo3b,Slc17a5,Dnajb8,Lamtor4,Kif1b,Slc25a44,Maml1,Pcmtd1,Rcan1,Lyrm2,Zfp24,Rab7b,D430042O09Rik,Immt,Ldhd,H2-Q1,Smtn,Hdhd5,Pstk,Fnip2,Cop1,Mettl6,Pcbp2,Impdh2,Dysf,mt-Cytb,Mob2,Slc31a1,Tmem87a,Tmem167b,Rusc1,Naa80,Chchd2,Entpd6,Sec31a,Mtch1,Alg8,Zfp280d,Sesn1,Lmo7,Rybp,Vamp5,Chac2,Mtmr4,Tmem97,Mmadhc,Usp20,Hdgfl3,Zfp62,Fam110a,Mkks,Zmynd11,Sh3bgrl2,Cntnap2,Tead3,Tcf4,Ei24,Hnrnpk,Ube4a,Arsk,Ngrn,Cnih4,Myo5a,Abhd13,Lsm11,Ppm1m,Rfx5,Dad1,Slc37a2,Pip4p1,Hps1,Tusc3,Ccnd3,Lrrcc1,Lsm5,Sgk3,Ghitm,Gnb1,Jakmip3,Ppt2,Llgl2,Usp27x,Map4k4,Nme7,Fgf2,Ppp4c,Parp16,Cdkn2d,ENSMUSP00000154304,Rab18,Atp5md,Magt1
	GO:0031090	Organelle membrane	3373	0.33	Sdhd,Tmem39a,Smpd4,Ndufa1,Actn1,Ptdss1,Anxa8,Samm50,Bad,Hras,Syngr2,Coq10b,Rab5if,Ssr3,Lamtor2,Calu,Gabarapl1,Slc10a7,Snx1,Mboat7,Fkbp1a,Mrps6,Foxred1,Tmem109,Hgsnat,Snx10,Ostc,Timm8a1,Tk2,Dpy19l3,Uqcr10,Slc17a5,Lamtor4,Kif1b,Slc25a44,Rab7b,Immt,Ldhd,H2-Q1,Fnip2,Cop1,Dysf,mt-Cytb,Tmem87a,Tmem167b,Entpd6,Sec31a,Mtch1,Alg8,Vamp5,Tmem97,

					Sh3bgrl2,Ei24,Ngrn,Cnih4,Dad1,Slc37a2,Pip4p1,Tusc3,Ghitm,Gnb1,Parp16,Atp5md,Magt1
	GO:0043226	Organelle	13445	0.12	Sdhd,Tmem39a,Smpd4,Elk3,Ifi211,Wdr77,Tubb1,Ndufa1,Ccdc12,Fbxo5,Recql5,Pimreg,Actn1,Tubal3,Ptdss1,Anxa8,Sub1,Samm50,Zfp263,Ncbp2,Cfap298,Nudt3,Diaph1,Bad,Dcxr,Hras,Syngr2,Plk4,Coq10b,Nmi,Rab5if,Naa15,Stoml3,Ssr3,Ints12,Lamtor2,Calu,Gabarapl1,Lhpp,Slc10a7,Rp9,Snx1,Nudt16,Hif1an,Mboat7,Fkbp1a,Mrps6,Foxred1,Ythdc1,Tmem109,Hgsnat,Cdk19,Pdk2,Med30,Snx10,Dtx3,2410004B18Rik,Secisbp2,H1f2,Myh14,Ostc,Sirt6,Timm8a1,Cep112,D1Ertd622e,Utp14b,Nfib,Meaf6,Tk2,Dpy19l3,Uqcr10,Myo3b,Slc17a5,Dnajb8,Lamtor4,Kif1b,Slc25a44,Maml1,Rcan1,Lyrm2,Zfp24,Rab7b,D430042O09Rik,Immt,Ldhd,H2-Q1,Smtn,Hdhd5,Pstk,Fnip2,Cop1,Mettl6,Pcbp2,Impdh2, Dysf,mt-Cytb,Mob2,Slc31a1,Tmem87a,Tmem167b,Rusc1,Chchd2,Entpd6,Sec31a,Mtch1,Alg8,Zfp280d,Sesn1,Lmo7,Rybp,Vamp5,Mtmr4,Tmem97,Mmadhc,Usp20,Hdgfl3,Zfp62,Fam110a,Mkks,Zmynd11,Sh3bgrl2,Cntnap2,Tead3,Tcf4,Ei24,Hnrnpk,Ube4a,Arsk,Ngrn,Cnih4,Myo5a,Lsm11,Ppm1m,Rfx5,Dad1,Slc37a2,Pip4p1,Hps1,Tusc3,Ccnd3,Lrrcc1,Lsm5,Sgk3,Ghitm,Gnb1,Jakmip3,Ppt2,Llgl2,Usp27x,Nme7,Fgf2,Ppp4c,Parp16,Cdkn2d,ENSMUSP00000154304,Rab18,Atp5md,Magt1
	GO:0043229	Intracellular organelle	13119	0.12	Sdhd,Tmem39a,Smpd4,Elk3,Ifi211,Wdr77,Tubb1,Ndufa1,Ccdc12,Fbxo5,Recql5,Pimreg,Actn1,Tubal3,Ptdss1,Anxa8,Sub1,Samm50,Zfp263,Ncbp2,Cfap298,Nudt3,Diaph1,Bad,Dcxr,Hras,Syngr2,Plk4,Coq10b,Nmi,Rab5if,Naa15,Ssr3,Ints12,Lamtor2,Calu,Gabarapl1,Lhpp,Slc10a7,Rp9,Snx1,Nudt16,Hif1an,Mboat7,Fkbp1a,Mrps6,Foxred1,Ythdc1,Tmem109,Hgsnat,Cdk19,Pdk2,Med30,Snx10,Dtx3,2410004B18Rik,Secisbp2,H1f2,Myh14,Ostc,Sirt6,Timm8a1,Cep112,Utp14b,Nfib,Meaf6,Tk2,Dpy19l3,Uqcr10,Myo3b,Slc17a5,Dnajb8,Lamtor4,Kif1b,Slc25a44,Maml1,Rcan1,Lyrm2,Zfp24,Rab7b,D430042O09Rik,Immt,Ldhd,H2-Q1,Smtn,

					Hdhd5,Pstk,Fnip2,Cop1,Mettl6,Pcbp2,Impdh2,Dysf,mt-Cytb,Mob2, Slc31a1, Tmem87a,Tmem167b,Rusc1,Chchd2,Entpd6,Sec31a,Mtch1,Alg8,Zfp280d, Sesn1,Lmo7,Rybp,Vamp5,Mtmr4,Tmem97,Mmadhc,Usp20,Hdgfl3,Zfp62,Fam110a,Mkks,Zmynd11,Sh3bgrl2,Cntnap2,Tead3,Tcf4,Ei24,Hnrnpk,Ube4a,Arsk,Ngrn, Cnih4,Myo5a,Lsm11,Ppm1m,Rfx5,Dad1,Slc37a2,Pip4p1,Hps1,Tusc3,Ccnd3,Lrrcc1,Lsm5,Sgk3,Ghitm,Jakmip3,Ppt2,Llgl2,Usp27x,Nme7,Fgf2,Ppp4c,Parp16,Cdkn2d,ENSMUSP00000154304,Rab18,Atp5md,Magt1
	GO:0005737	Cytoplasm	11788	0.14	Sdhd,Grk6,Gstt3,Tmem39a,Eno2,Smpd4,Elk3,Ifi211,Wdr77,Tubb1,Ndufa1,Fbxo5,Recql5,Actn1,Tubal3,Ptdss1,Anxa8,Samm50,Ncbp2,Cfap298,Nudt3,Diaph1,Bad, Dcxr,Hras,Syngr2,Plk4,Coq10b,Nmi,Zmynd19,Rab5if,Naa15,Ssr3,Lamtor2,Calu, Gabarapl1,Lhpp,Slc10a7,Snx1,Nudt16,Hif1an,Mboat7,Fkbp1a,Ggact,Mrps6, Foxred1,Tmem109,Hgsnat,Cdk19,Pdk2,Snx10,Dtx3,Secisbp2,Myh14,Ostc,Sirt6, Timm8a1,Cep112,D1Ertd622e,Utp14b,Tk2,Uqcr10,Myo3b,Slc17a5,Dnajb8,Lamtor4,Kif1b,Slc25a44,Pcmtd1,Rcan1,Lyrm2,Rab7b,D430042O09Rik,Immt,Ldhd,H2-Q1,Smtn,Hdhd5,Pstk,Fnip2,Cop1,Mettl6,Pcbp2, Impdh2,Dysf,mt-Cytb,Mob2, Slc31a1,Tmem87a,Tmem167b,Rusc1,Naa80,Chchd2, Entpd6,Sec31a,Mtch1, Alg8,Sesn1,Lmo7,Rybp,Vamp5,Chac2,Mtmr4,Tmem97,Mmadhc,Usp20,Hdgfl3,Fam110a,Mkks,Cntnap2,Ei24,Hnrnpk,Ube4a,Arsk,Ngrn,Cnih4,Myo5a,Abhd13,Dad1,Slc37a2,Pip4p1,Hps1,Tusc3,Ccnd3,Lrrcc1,Lsm5,Sgk3,Ghitm,Gnb1,Jakmip3,Ppt2, Llgl2,Usp27x,Map4k4,Nme7,Fgf2,Ppp4c,Parp16,Cdkn2d,ENSMUSP00000154304, Rab18,Atp5md,Magt1
	GO:0043227	Membrane-bounded organelle	12435	0.13	Sdhd,Tmem39a,Smpd4,Elk3,Ifi211,Wdr77,Ndufa1,Ccdc12,Fbxo5,Recql5,Pimreg, Actn1,Ptdss1,Anxa8,Sub1,Samm50,Zfp263,Ncbp2,Cfap298,Nudt3,Diaph1,Bad, Hras,Syngr2,Plk4,Coq10b,Nmi,Rab5if,Naa15,Stoml3,Ssr3,Ints12,Lamtor2,Calu, Gabarapl1,Lhpp,Slc10a7,Rp9,Snx1,Nudt16,Hif1an,Mboat7,Fkbp1a,Mrps6,

					Foxred1,Ythdc1,Tmem109,Hgsnat,Cdk19,Pdk2,Med30,Snx10,Dtx3,2410004B18Rik,Secisbp2,H1f2,Ostc,Sirt6,Timm8a1,D1Ertd622e,Utp14b,Nfib,Meaf6,Tk2,Dpy19l3,Uqcr10,Myo3b,Slc17a5,Dnajb8,Lamtor4,Kif1b,Slc25a44,Maml1,Rcan1,Lyrm2,Zfp24,Rab7b,Immt,Ldhd,H2-Q1,Hdhd5,Pstk,Fnip2,Cop1, Mettl6,Pcbp2,Impdh2,Dysf,mt-Cytb,Mob2,Slc31a1,Tmem87a,Tmem167b,Rusc1,Chchd2,Entpd6,Sec31a,Mtch1,Alg8,Zfp280d,Sesn1,Lmo7,Rybp,Vamp5,Mtmr4,Tmem97,Mmadhc,Hdgfl3,Zfp62,Mkks,Zmynd11,Sh3bgrl2,Cntnap2,Tead3,Tcf4,Ei24,Hnrnpk,Ube4a,Arsk,Ngrn,Cnih4,Myo5a,Lsm11,Ppm1m,Rfx5,Dad1,Slc37a2,Pip4p1,Hps1,Tusc3,Ccnd3,Lsm5,Sgk3,Ghitm,Gnb1,Jakmip3,Ppt2,Llgl2,Usp27x,Nme7,Fgf2,Ppp4c,Parp16,Cdkn2d,ENSMUSP00000154304,Rab18,Atp5md,Magt1
	GO:0043231	Intracellular membrane-bounded organelle	11954	0.13	Sdhd,Tmem39a,Smpd4,Elk3,Ifi211,Wdr77,Ndufa1,Ccdc12,Fbxo5,Recql5,Pimreg,Actn1,Ptdss1,Anxa8,Sub1,Samm50,Zfp263,Ncbp2,Nudt3,Diaph1,Bad,Hras,Syngr2,Plk4,Coq10b,Nmi,Rab5if,Naa15,Ssr3,Ints12,Lamtor2,Calu,Gabarapl1,Lhpp,Slc10a7,Rp9,Snx1,Nudt16,Hif1an,Mboat7,Fkbp1a,Mrps6,Foxred1,Ythdc1,Tmem109,Hgsnat,Cdk19,Pdk2,Med30,Snx10,Dtx3,2410004B18Rik,Secisbp2,H1f2,Ostc,Sirt6,Timm8a1,Utp14b,Nfib,Meaf6,Tk2,Dpy19l3,Uqcr10,Slc17a5,Dnajb8,Lamtor4,Kif1b,Slc25a44,Maml1,Rcan1,Lyrm2,Zfp24,Rab7b,Immt,Ldhd,H2-Q1,Hdhd5,Pstk,Fnip2,Cop1,Mettl6,Pcbp2,Impdh2,Dysf,mt-Cytb,Mob2,Slc31a1,Tmem87a,Tmem167b,Rusc1,Chchd2,Entpd6,Sec31a,Mtch1,Alg8,Zfp280d,Sesn1,Lmo7,Rybp,Vamp5,Mtmr4,Tmem97,Mmadhc,Hdgfl3,Zfp62,Mkks,Zmynd11,Sh3bgrl2,Cntnap2,Tead3,Tcf4,Ei24,Hnrnpk,Ube4a,Arsk,Ngrn,Cnih4,Myo5a,Lsm11,Ppm1m,Rfx5,Dad1,Slc37a2,Pip4p1,Hps1,Tusc3,Ccnd3,Lsm5,Sgk3,Ghitm,Jakmip3,Ppt2,Llgl2,Usp27x,Fgf2,Ppp4c,Parp16,Cdkn2d,ENSMUSP00000154304,Rab18,Atp5md,Magt1

	GO:0008250	Oligosaccharyltra nsferase complex	14	1.51	Ostc,Dad1,Tusc3,Magt1
	GO:0031967	Organelle envelope	1324	0.38	Sdhd,Smpd4,Ndufa1,Samm50,Bad,Coq10b,Rab5if,Mrps6,Foxred1,Tmem109,Timm8a1,Tk2,Dpy19l3,Uqcr10,Slc25a44,Immt,Ldhd,mt-Cytb,Chchd2,Mtch1,Lmo7,Tmem97,Sh3bgrl2,Ei24,Ngrn,Ghitm,Parp16,Atp5md
	GO:0098798	Mitochondrial protein-containing complex	297	0.66	Sdhd,Ndufa1,Samm50,Mrps6,Foxred1,Pdk2,Timm8a1,Uqcr10,Immt,mt-Cytb,ENSMUSP00000154304,Atp5md
	GO:0005743	Mitochondrial inner membrane	556	0.51	Sdhd,Ndufa1,Samm50,Coq10b,Rab5if,Mrps6,Foxred1,Timm8a1,Tk2,Uqcr10,Slc25a44,Immt,Ldhd,mt-Cytb,Ghitm,Atp5md
	GO:0019866	Organelle inner membrane	617	0.49	Sdhd,Ndufa1,Samm50,Coq10b,Rab5if,Mrps6,Foxred1,Timm8a1,Tk2,Dpy19l3,Uqcr10,Slc25a44,Immt,Ldhd,mt-Cytb,Ghitm,Atp5md
	GO:0005740	Mitochondrial envelope	854	0.42	Sdhd,Ndufa1,Samm50,Bad,Coq10b,Rab5if,Mrps6,Foxred1,Timm8a1,Tk2,Uqcr10,Slc25a44,Immt,Ldhd,mt-Cytb,Chchd2,Mtch1,Ngrn,Ghitm,Atp5md
	GO:0031966	Mitochondrial membrane	795	0.43	Sdhd,Ndufa1,Samm50,Bad,Coq10b,Rab5if,Mrps6,Foxred1,Timm8a1,Tk2,Uqcr10,Slc25a44,Immt,Ldhd,mt-Cytb,Mtch1,Ngrn,Ghitm,Atp5md
	GO:0005746	Mitochondrial respirasome	90	0.88	Sdhd,Ndufa1,Rab5if,Foxred1,Uqcr10,mt-Cytb

	GO:0098827	Endoplasmic reticulum subcompartment	1106	0.35	Tmem39a,Smpd4,Ptdss1,Ssr3,Calu,Slc10a7,Mboat7,Fkbp1a,Tmem109,Ostc,H2-Q1,Sec31a,Alg8,Tmem97,Ei24,Cnih4,Dad1,Slc37a2,Tusc3,Parp16,Rab18,Magt1
	CL:17259	Oligosaccharyltransferase complex, and Post-translational protein targeting to membrane, translocation	27	1.32	Ssr3,Ostc,Dad1,Tusc3,Magt1
	GOCC: 0005622	Intracellular	10948	0.16	Sdhd,Grk6,Gstt3,Eno2,Smpd4,Elk3,Ifi211,Wdr77,Tubb1,Ndufa1,Fbxo5,Recql5,Pimreg,Actn1,Tubal3,Ptdss1,Anxa8,Sub1,Samm50,Zfp263,Ncbp2,Nudt3,Diaph1,Bad,Dcxr,Hras,Syngr2,Plk4,Coq10b,Nmi,Zmynd19,Rab5if,Naa15,Ints12,Lamtor2,Calu,Gabarapl1,Lhpp,Slc10a7,Rp9,Snx1,Hif1an,Fkbp1a,Ggact,Mrps6,Foxred1,Ythdc1,Tmem109,Hgsnat,Cdk19,Pdk2,Med30,Snx10,Secisbp2,H1f2,Myh14,Ostc,Sirt6,Timm8a1,Cep112,Utp14b,Nfib,Meaf6,Tk2,Uqcr10,Myo3b,Slc17a5,Dnajb8,Lamtor4,Kif1b,Slc25a44,Maml1,Rcan1,Lyrm2,Zfp24,Rab7b,Immt,Ldhd,H2-Q1,Hdhd5,Fnip2,Cop1,Mettl6,Pcbp2,Dysf,mt-Cytb,Mob2, Slc31a1,Tmem87a,Rusc1,Naa80,Chchd2,Entpd6,Sec31a,Sesn1,Lmo7,Rybp,Vamp5,Mtmr4,Tmem97,Mmadhc,Hdgfl3,Fam110a,Mkks,Zmynd11,Tead3,Tcf4,Hnrnpk,Ube4a,Arsk,Ngrn,Cnih4,Myo5a,Abhd13,Lsm11,Ppm1m,Rfx5,Zbtb8os,Dad1,Pip4p1,Hps1,Tusc3,Ccnd3,Lrrcc1,Lsm5,Sgk3,Ghitm,Gnb1,Ppt2,Llgl2,Map4k4,Nme7,Fgf2,Ppp4c,Parp16,Cdkn2d,Rab18,Atp5md,Magt1

	GOCC: 0043226	Organelle	9352	0.16	Sdhd,Smpd4,Elk3,Ifi211,Tubb1,Ndufa1,Fbxo5,Recql5,Pimreg,Actn1,Tubal3,Ptdss1,Anxa8,Sub1,Samm50,Zfp263,Diaph1,Bad,Dcxr,Hras,Syngr2,Plk4,Coq10b,Rab5if,Stoml3,Ints12,Lamtor2,Calu,Gabarapl1,Slc10a7,Rp9,Snx1,Mboat7,Fkbp1a,Ggact,Mrps6,Foxred1,Ythdc1,Tmem109,Hgsnat,Cdk19,Pdk2,Med30,Snx10,Secisbp2,H1f2,Myh14,Ostc,Sirt6,Timm8a1,Cep112,D1Ertd622e,Utp14b,Nfib,Meaf6,Tk2,Uqcr10,Myo3b,Slc17a5,Lamtor4,Kif1b,Slc25a44,Maml1,Rcan1,Lyrm2,Zfp24,Rab7b,Immt,Ldhd,H2-Q1,Hdhd5,Fnip2,Cop1, Pcbp2,Dysf,mt-Cytb,Slc31a1,Tmem87a,Rusc1,Chchd2,Entpd6,Sec31a,Sesn1,Lmo7, Rybp,Vamp5,Tmem97,Mmadhc,Hdgfl3,Fam110a,Mkks,Zmynd11,Tead3,Tcf4,Hnrnpk,Arsk,Ngrn,Cnih4,Myo5a,Lsm11,Ppm1m,Rfx5,Dad1,Slc37a2,Pip4p1,Tusc3,Ccnd3,Lrrcc1,Lsm5,Sgk3,Ghitm,Gnb1,Ppt2,Nme7,Fgf2,Ppp4c,Parp16,Rab18,Atp5md,Magt1
	GOCC: 0043227	Membrane-bounded organelle	8117	0.18	Sdhd,Smpd4,Elk3,Ifi211,Ndufa1,Fbxo5,Recql5,Pimreg,Actn1,Ptdss1,Anxa8,Sub1,Samm50,Zfp263,Diaph1,Bad,Hras,Syngr2,Plk4,Coq10b,Rab5if,Stoml3,Ints12,Lamtor2,Calu,Gabarapl1,Slc10a7,Rp9,Snx1,Fkbp1a,Mrps6,Foxred1,Ythdc1,Tmem109,Hgsnat,Cdk19,Pdk2,Med30,Snx10,Secisbp2,H1f2,Ostc,Sirt6,Timm8a1,Utp14b,Nfib,Meaf6,Tk2,Uqcr10,Myo3b,Slc17a5,Lamtor4,Kif1b,Slc25a44,Maml1,Rcan1,Lyrm2,Zfp24,Rab7b,Immt,Ldhd,H2-Q1,Hdhd5,Cop1,Pcbp2,Dysf,mt-Cytb,Slc31a1,Tmem87a,Rusc1,Chchd2,Entpd6,Sec31a,Sesn1,Lmo7,Rybp,Vamp5,Tmem97,Mmadhc,Hdgfl3,Mkks,Zmynd11,Tead3,Tcf4,Hnrnpk,Arsk,Ngrn,Cnih4,Myo5a,Lsm11,Ppm1m,Rfx5,Dad1,Slc37a2,Pip4p1,Tusc3,Ccnd3,Lsm5,Sgk3,Ghitm,Gnb1,Ppt2,Nme7,Fgf2,Ppp4c,Parp16,Rab18,Atp5md,Magt1
	GOCC: 0043231	Intracellular membrane-	7393	0.19	Sdhd,Smpd4,Elk3,Ifi211,Ndufa1,Fbxo5,Recql5,Pimreg,Actn1,Ptdss1,Sub1,Samm50,Zfp263,Diaph1,Bad,Hras,Syngr2,Plk4,Coq10b,Rab5if,Ints12,Lamtor2,Calu,Gabarapl1,Slc10a7,Rp9,Snx1,Fkbp1a,Mrps6,Foxred1,Ythdc1,Tmem109,Hgsnat,Cdk19,Pdk2,Med30,Snx10,Secisbp2, H1f2,Ostc,Sirt6,Timm8a1,

		bounded organelle			Utp14b,Nfib,Meaf6,Tk2,Uqcr10,Slc17a5,Lamtor4,Kif1b,Slc25a44,Maml1,Rcan1, Lyrm2,Zfp24,Rab7b,Immt,Ldhd,H2-Q1,Hdhd5,Cop1, Pcbp2,Dysf,mt-Cytb, Slc31a1,Tmem87a,Rusc1,Chchd2,Entpd6,Sec31a,Sesn1,Lmo7, Rybp,Vamp5, Tmem97,Mmadhc,Hdgfl3,Zmynd11,Tead3,Tcf4,Hnrnpk,Arsk,Ngrn,Cnih4,Myo5a, Lsm11,Ppm1m,Rfx5,Dad1,Pip4p1,Tusc3,Ccnd3,Lsm5,Sgk3,Ghitm,Ppt2,Fgf2,Ppp4 c,Parp16,Rab18,Atp5md,Magt1
	GOCC: 0043229	Intracellular organelle	8917	0.16	Sdhd,Smpd4,Elk3,Ifi211,Tubb1,Ndufa1,Fbxo5,Recql5,Pimreg,Actn1,Tubal3, Ptdss1,Anxa8,Sub1,Samm50,Zfp263,Diaph1,Bad,Dcxr,Hras,Syngr2,Plk4,Coq10b, Rab5if,Ints12,Lamtor2,Calu,Gabarapl1,Slc10a7,Rp9,Snx1,Fkbp1a,Ggact,Mrps6, Foxred1,Ythdc1,Tmem109,Hgsnat,Cdk19,Pdk2,Med30,Snx10,Secisbp2,H1f2, Myh14,Ostc,Sirt6,Timm8a1,Cep112,Utp14b,Nfib,Meaf6,Tk2,Uqcr10,Myo3b,Slc1 7a5,Lamtor4,Kif1b,Slc25a44,Maml1,Rcan1,Lyrm2,Zfp24,Rab7b,Immt,Ldhd,H2-Q1,Hdhd5,Fnip2,Cop1,Pcbp2,Dysf,mt-Cytb,Slc31a1, Tmem87a,Rusc1,Chchd2,Entpd6,Sec31a,Sesn1,Lmo7,Rybp,Vamp5,Tmem97,Mm adhc,Hdgfl3,Fam110a,Mkks,Zmynd11,Tead3,Tcf4,Hnrnpk,Arsk,Ngrn,Cnih4,Myo5 a,Lsm11,Ppm1m,Rfx5,Dad1,Pip4p1,Tusc3,Ccnd3,Lrrcc1,Lsm5,Sgk3,Ghitm,Ppt2,N me7,Fgf2,Ppp4c,Parp16,Rab18,Atp5md,Magt1
	GOCC: 0005737	Cytoplasm	7282	0.18	Sdhd,Grk6,Gstt3,Eno2,Smpd4,Ifi211,Wdr77,Tubb1,Ndufa1,Fbxo5,Actn1,Ptdss1,A nxa8,Samm50,Diaph1,Bad,Dcxr,Hras,Syngr2,Plk4,Coq10b,Zmynd19,Rab5if, Naa15,Lamtor2,Calu,Gabarapl1,Slc10a7,Snx1,Fkbp1a,Mrps6,Foxred1,Hgsnat, Pdk2,Snx10,Myh14,Ostc,Sirt6,Timm8a1,Tk2,Uqcr10,Myo3b,Slc17a5,Dnajb8,Lam tor4,Kif1b,Slc25a44,Rcan1,Lyrm2,Rab7b,Immt,Ldhd,H2-Q1,Hdhd5,Fnip2,Cop1, Mettl6,Pcbp2,Dysf,mt-Cytb,Mob2, Slc31a1,Tmem87a,Rusc1,Naa80,Chchd2,Entpd6,Sec31a,Sesn1,Lmo7,Vamp5,Mt mr4,Tmem97,Mmadhc,Hdgfl3,Fam110a,Hnrnpk,Ube4a,Arsk,Ngrn,Cnih4,Myo5a,

					Abhd13,Dad1,Pip4p1,Hps1,Tusc3,Ccnd3,Sgk3,Ghitm,Ppt2,Llgl2,Nme7,Fgf2,Ppp4c,Parp16,Rab18,Atp5md,Magt1
	GOCC: 0031090	Organelle membrane	1929	0.37	Sdhd,Smpd4,Ndufa1,Actn1,Ptdss1,Anxa8,Samm50,Bad,Syngr2,Rab5if,Lamtor2,Calu,Gabarapl1,Snx1,Fkbp1a,Foxred1,Tmem109,Hgsnat,Snx10,Ostc,Uqcr10,Slc17a5,Lamtor4,Kif1b,Slc25a44,Immt,H2-Q1,Cop1,mt-Cytb,Tmem87a,Sec31a,Tmem97,Dad1,Slc37a2,Pip4p1,Tusc3,Ghitm,Gnb1,Atp5md,Magt1
	GOCC: 0110165	Cellular anatomical entity	13279	0.1	Sdhd,Grk6,Gstt3,Tmem39a,Eno2,Smpd4,Elk3,Ifi211,Wdr77,Tubb1,Ndufa1,Fbxo5,Recql5,Pimreg,Actn1,Tubal3,Ptdss1,Anxa8,Sub1,Samm50,Zfp263,Cldnd1,Diaph1,Bad,Dcxr,Inpp5a,Hras,Syngr2,Plk4,Coq10b,Lamc1,Nmi,Zmynd19,Rab5if,Naa15,Stoml3,Ints12,Lamtor2,Calu,Gabarapl1,Sema4b,Slc10a7,Rp9,Snx1,Mboat7,Fkbp1a,Ggact,Mrps6,Frem3,Foxred1,Ythdc1,Tmem109,Hgsnat,Cdk19,Pdk2,Med30,Snx10,Dtx3,Secisbp2,H1f2,Myh14,Ostc,Sirt6,Timm8a1,Cep112,D1Ertd622e,Utp14b,Nfib,Meaf6,Tk2,Uqcr10,Myo3b,Slc17a5,Dnajb8,Lamtor4,Kif1b,Slc25a44,Maml1,Rcan1,Lyrm2,Zfp24,Rab7b,Immt,Ldhd,H2-Q1,Unc5c,Hdhd5,Fnip2,Cop1,Mettl6,Pcbp2,Mug2,Dysf,mt-Cytb,Mob2,Slc31a1,Tmem87a,Rusc1,Naa80,Chchd2,Entpd6,Sec31a,Sesn1,Lmo7,Rybp,Vamp5,Mtmr4,Tmem97,Rtn4rl1,Mmadhc,Hdgfl3,Fam110a,Mkks,Zmynd11,Cntnap2,Tead3,Tcf4,Timp1,Hnrnpk,Ube4a,Arsk,Ngrn,Cnih4,Myo5a,Abhd13,Lsm11,Ppm1m,Rfx5,Dad1,Slc37a2,Pip4p1,Hps1,Tusc3,Ccnd3,Lrrcc1,Lsm5,Sgk3,Ghitm,Gnb1,Ppt2,Pear1,Llgl2,Map4k4,Nme7,Fgf2,Ppp4c,Parp16,Rab18,Atp5md,Magt1
	GOCC: 0008250	Oligosaccharyltransferase complex	14	1.51	Ostc,Dad1,Tusc3,Magt1

	GOCC: 0005746	Mitochondrial respirasome	80	0.93	Sdhd,Ndufa1,Rab5if,Foxred1,Uqcr10,mt-Cytb
	GOCC: 0098798	Mitochondrial protein complex	304	0.61	Sdhd,Ndufa1,Samm50,Mrps6,Foxred1,Pdk2,Timm8a1,Uqcr10,Immt,mt-Cytb,Atp5md
	GOCC: 0031967	Organelle envelope	851	0.4	Sdhd,Smpd4,Ndufa1,Samm50,Bad,Rab5if,Foxred1,Tmem109,Timm8a1,Uqcr10,Slc25a44,Immt,mt-Cytb,Chchd2,Lmo7,Tmem97,Ghitm,Parp16,Atp5md
	GOCC: 0005770	Late endosome	234	0.64	Anxa8,Lamtor2,Lamtor4,Rab7b,Dysf,Slc31a1,Vamp5,Myo5a,Pip4p1
	GOCC: 0005743	Mitochondrial inner membrane	348	0.55	Sdhd,Ndufa1,Samm50,Rab5if,Foxred1,Uqcr10,Slc25a44,Immt,mt-Cytb,Ghitm,Atp5md
	KW-0007	Acetylation	3080	0.24	Eno2,Ccdc12,Actn1,Ptdss1,Sub1,Ncbp2,Nudt3,Diaph1,Dcxr,Hras,Syngr2,Plk4,Naa15,Ssr3,Calu,Snx1,Hif1an,Fkbp1a,Cdk19,Med30,H1f2,Myh14,Sirt6,D1Ertd622e,Meaf6,Lamtor4,Kif1b,Maml1,Immt,Ldhd,Smtn,Impdh2,Mmadhc,Tead3,Ei24,Hnrnpk,Ube4a,Myo5a,Rfx5,Zbtb8os,Dad1,Lsm5,Gnb1,Map4k4,Ppp4c,Cdkn2d,Rab18,Atp5md
	KW-0999	Mitochondrion inner membrane	287	0.63	Sdhd,Ndufa1,Coq10b,Rab5if,Foxred1,Timm8a1,Uqcr10,Immt,mt-Cytb,Mtch1,Ghitm

	KW-0496	Mitochondrion	1131	0.36	Sdhd,Ndufa1,Samm50,Bad,Coq10b,Rab5if,Mrps6,Foxred1,Pdk2,Timm8a1,Tk2,Uqcr10,Kif1b,Slc25a44,Immt,Ldhd,mt-Cytb,Chchd2,Mtch1,Mmadhc,Ngrn,Ghitm,Atp5md
	KW-0597	Phosphoprotein	7592	0.13	Grk6,Eno2,Smpd4,Elk3,Wdr77,Tubb1,Ccdc12,Fbxo5,Recql5,Pimreg,Actn1,Ptdss1,Sub1,Ncbp2,Diaph1,Bad,Syngr2,Plk4,Lamc1,Nmi,Naa15,Stoml3,Ssr3,Ints12,Calu,Sema4b,Rp9,Snx1,Lrrfip2,Ythdc1,Hgsnat,Cdk19,Pdk2,2410004B18Rik,H1f2,Myh14,9530068E07Rik,Sirt6,Timm8a1,D1Ertd622e,Utp14b,Nfib,Meaf6,Kif1b,Maml1,Rcan1,Zfp24,Rab7b,D430042O09Rik,Immt,Smtn,Unc5c,Fnip2,Pcbp2,Impdh2,Dysf,Mob2,Slc31a1,Tmem87a,Rusc1,Sec31a,Zfp280d,Sesn1,Rybp,Vamp5,Mtmr4,Usp20,Hdgfl3,Zfp62,Zmynd11,Cntnap2,Tead3,Tcf4,Ei24,Timp1,Hnrnpk,Ngrn,Fsd1l,Myo5a,Lsm11,Rfx5,Pip4p1,Ccnd3,Sgk3,Gnb1,Jakmip3,Pear1,Llgl2,Map4k4,Fgf2,Rab18

A

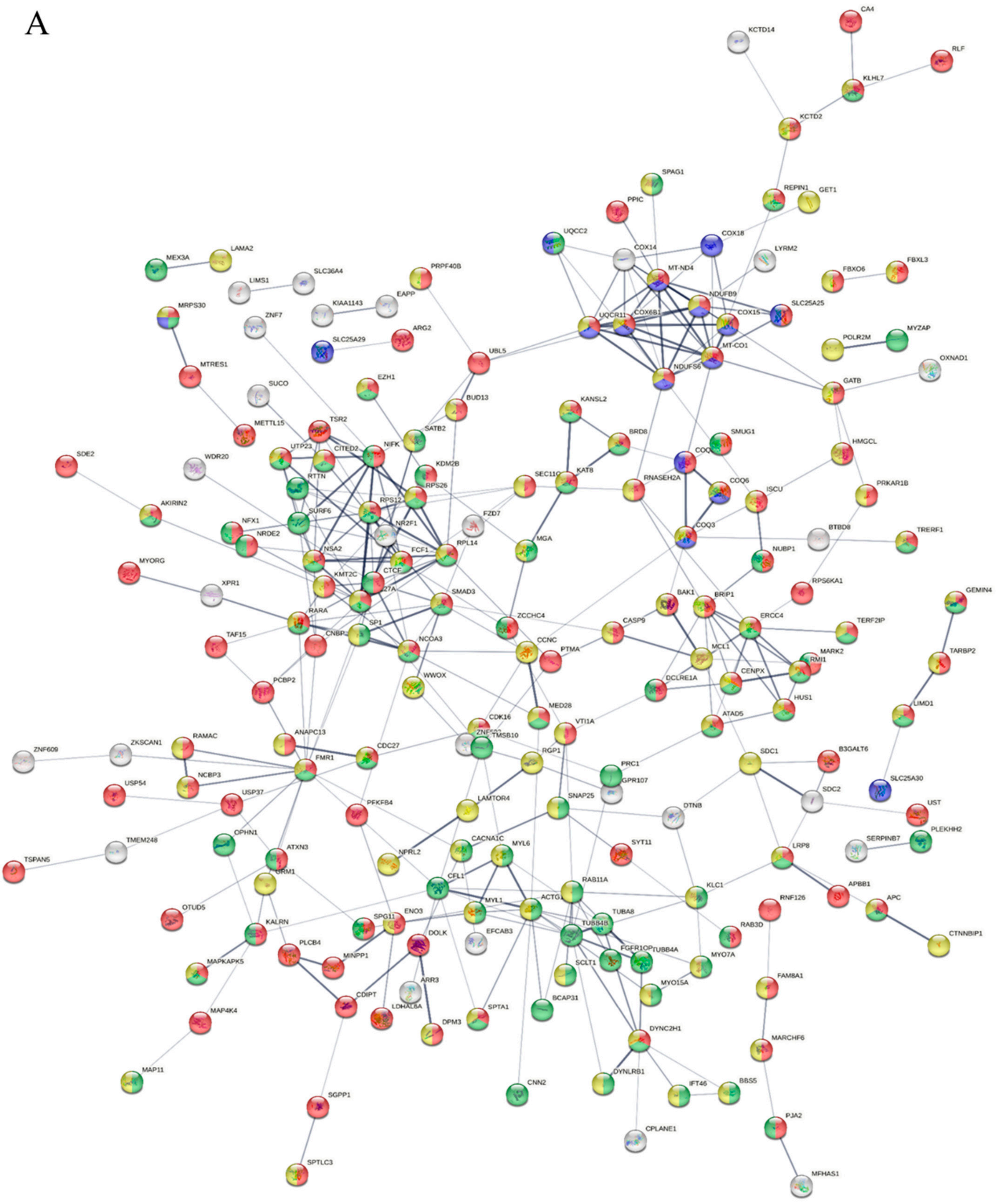
CA4
KCTD14
RLF
KLHL7
KCTD2
SPAG1
REPIN1
GET1
PPIC
UQCC2
COX18
COX14
LYRM2
MT-ND4
FBXO6
FBXL3
NDUFB9
SLC25A25
UQCR11
COX6B1
COX15
POLR2M
MYZAP
MT-CO1
OXNAD1
NDUFS6
GATB
MEX3A
LAMA2
LIMS1
SLC36A4
PRPF40B
MRPS30
KIAA1143
EAPP
ZNF7
SLC25A29
ARG2
UBL5
MTRES1
EZH1
SUCO
BUD13
KANSL2
METTL15
TSR2
SATB2
BRD8
SMUG1
HMGCL
UTP23
CITED2
NIFK
SDE2
WDR20
KDM2B
KAT8
RNASEH2A
COQ6
ISCU
PRKAR1B
RTTN
RPS26
AKIRIN2
SURF6
RPS12
FZD7
NFX1
NRDE2
NR2F1
MGA
COQ3
BTBD8
TRERF1
NSA2
FCF1
RPL14
NUBP1
MYORG
KMT2C
CTCF
XPR1
RPS6KA1
SMAD3
BAK1
BRIP1
GEMIN4
RARA
ZCCHC4
CASP9
ERCC4
TERF2IP
TAF15
CNBP
SP1
NCOA3
CCNC
PTMA
MCL1
MARK2
RMI1
TARBP2
DCLRE1A
CENPX
PCBP2
WWOX
HUS1
LIMD1
MED28
ATAD5
CDK16
VTI1A
ANAPC13
TMSB10
ZNF609
ZKSCAN1
RAMAC
CDC27
RGP1
SDC1
B3GALT6
FMR1
PRC1
GPR107
NCBP3
USP54
USP37
SLC25A30
SDC2
SNAP25
PFKFB4
LAMTOR4
DTNB
UST
PLEKHH2
SERPINB7
TMEM248
OPHN1
SYT11
CACNA1C
MYL6
ATXN3
LRP8
TSPAN5
NPRL2
GRM1
CFL1
RAB11A
APBB1
KLC1
APC
MYL1
ACTG1
OTUD5
ENO3
TUBA8
RNF126
KALRN
SPG11
TUBB4B
EFCAB3
DOLK
RAB3D
CTNNBIP1
PLCB4
MINPP1
FGFR1OP
TUBB4A
MYO7A
MAPKAPK5
SCLT1
ARR3
CDIPT
MYO15A
FAM8A1
LDHAL6A
MAP4K4
BCAP31
SPTA1
DPM3
DYNC2H1
MARCHF6
MAP11
CNN2
DYNLRB1
IFT46
BBS5
SGPP1
CPLANE1
PJA2
SPTLC3
MFHAS1

B

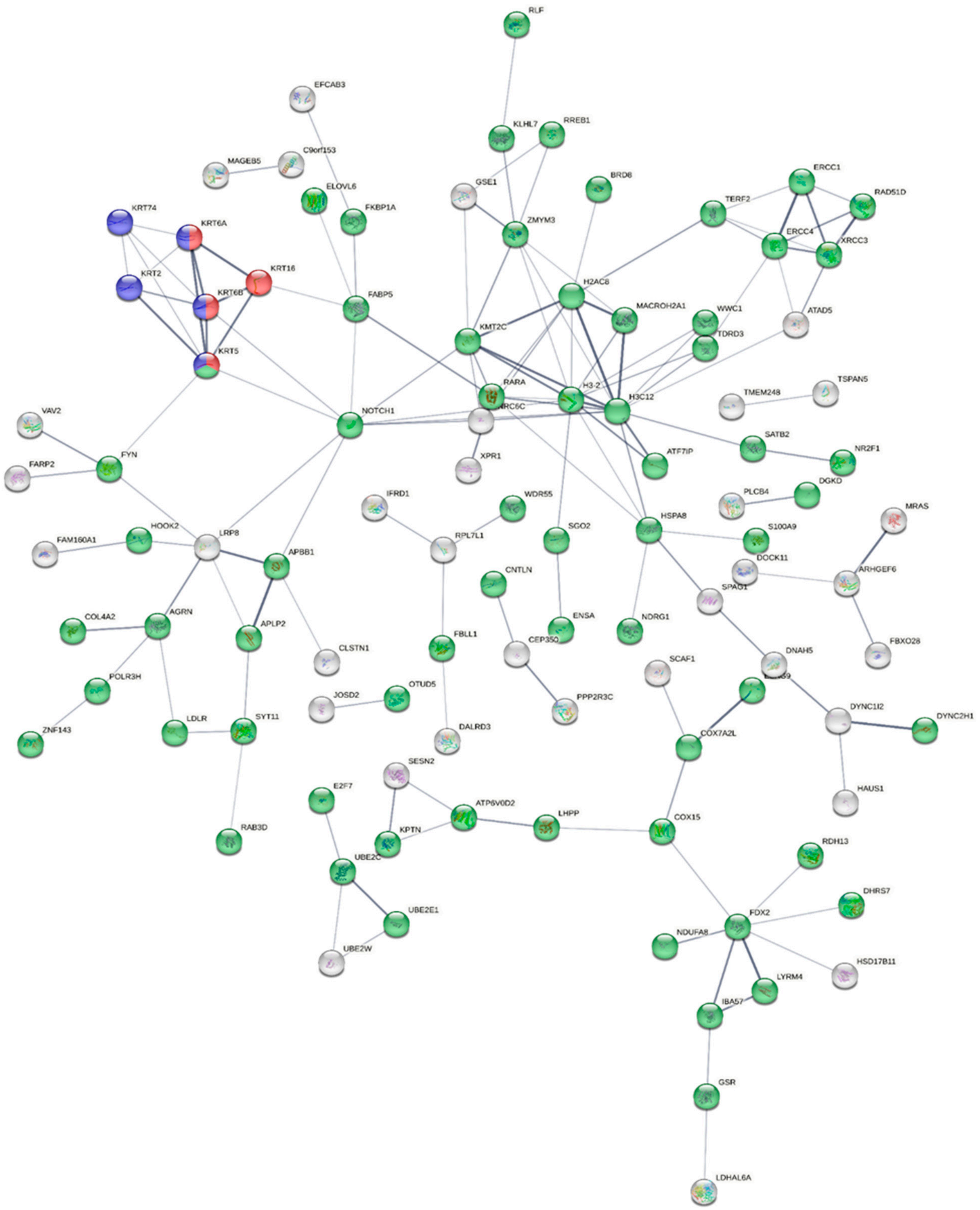
RLF
EFCAB3
KLHL7
RREB1
C9orf153
MAGEB5
ELOVL6
GSE1
BRD8
ERCC1
RAD51D
TERF2
KRT74
FKBP1A
ZMYM3
ERCC4
XRCC3
KRT6A
KRT16
KRT2
H2AC8
KRT6B
FABP5
MACROH2A1
WWC1
ATAD5
KMT2C
TDRD3
KRT5
RARA
H3-2
H3C12
TSPAN5
TMEM248
VAV2
NOTCH1
SATB2
XPR1
ATF7IP
NR2F1
FYN
FARP2
IFRD1
WDR55
PLCB4
DGKD
MRAS
HOOK2
SGO2
HSPA8
S100A9
FAM160A1
LRP8
RPL7L1
APBB1
DOCK11
ARHGEF6
CNTLN
COL4A2
AGRN
ENSA
NDRG1
APLP2
FBLL1
CEP350
FBXO28
CLSTN1
DNAH5
SCAF1
POLR3H
OTUD5
JOSD2
PPP2R3C
DYNC1I2
DYNC2H1
ZNF143
LDLR
SYT11
DALRD3
COX7A2L
SESN2
HAUS1
E2F7
ATP6V0D2
LHPP
RAB3D
KPTN
COX15
UBE2C
RDH13
DHRS7
UBE2E1
FDX2
NDUFA8
UBE2W
HSD17B11
LYRM4
IBA57
GSR
LDHAL6A

C

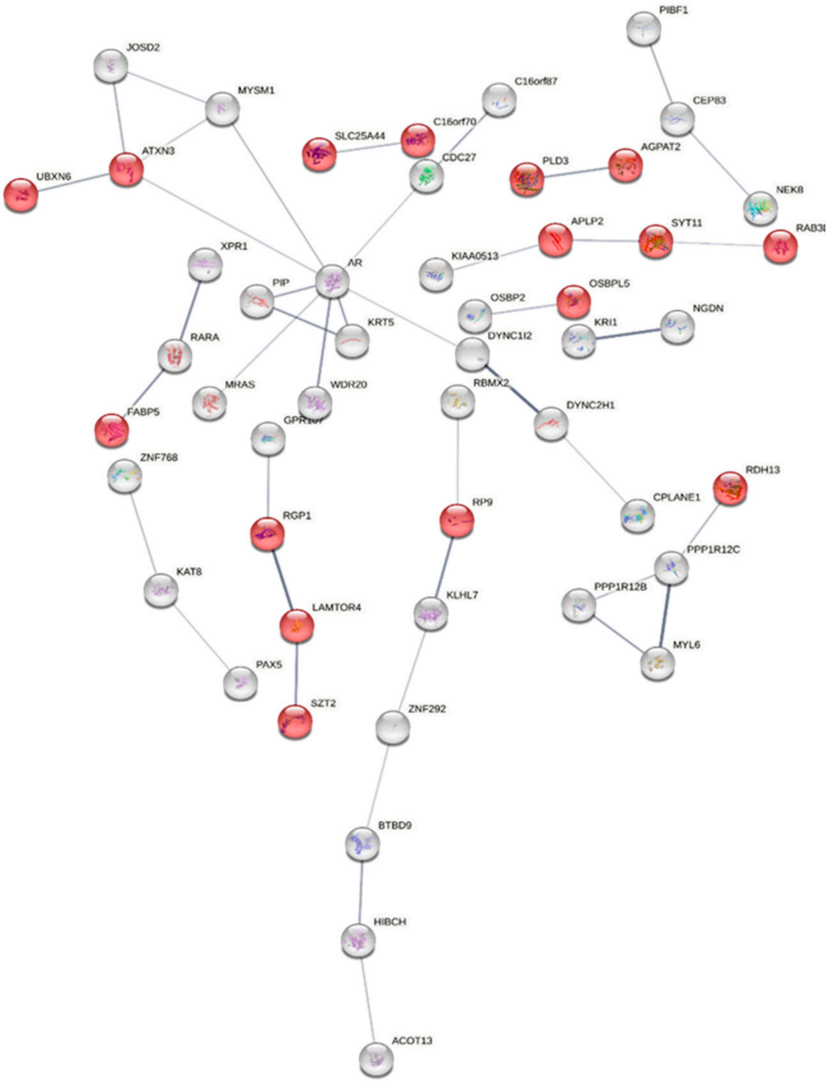
PIBF1
JOSD2
MYSM1
C16orf87
C16orf70
SLC25A44
CEP83
ATXN3
CDC27
AGPAT2
UBXN6
PLD3
NEK8
APLP2
SYT11
RAB3I
XPR1
AR
KIAA0513
PIP
OSBPL5
OSBP2
KRT5
NGDN
KRI1
DYNC1I2
RARA
MRAS
WDR20
RBMX2
DYNC2H1
FABP5
ZNF768
RDH13
RP9
CPLANE1
RGP1
PPP1R12C
KAT8
PPP1R12B
KLHL7
LAMTOR4
MYL6
PAX5
SZT2
ZNF292
BTBD9
HIBCH
ACOT13

D

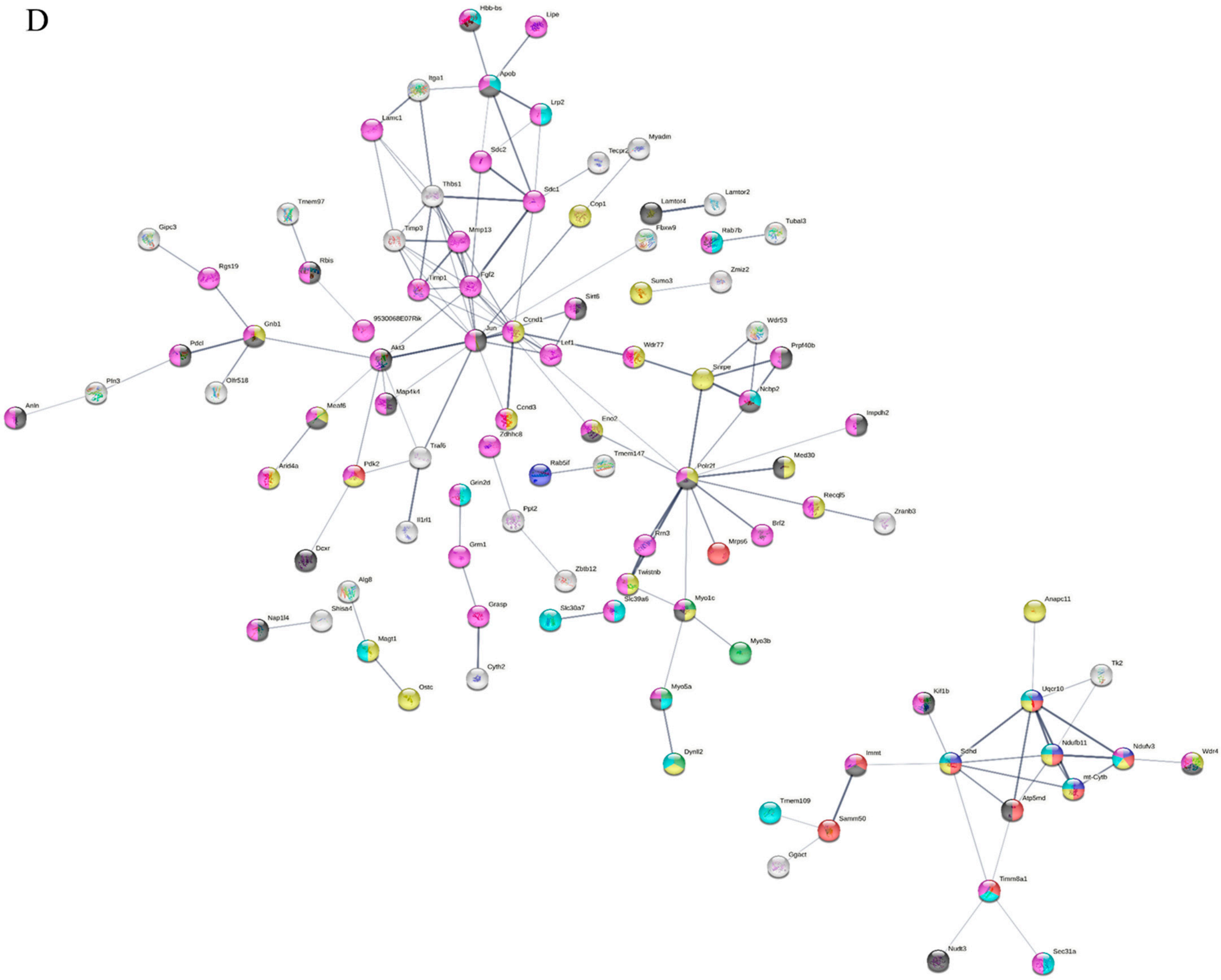
Hbb-bs
Lipe
Itga1
Apob
Lrp2
Lamc1
Sdc2
Myadm
Tecpr2
Thbs1
Sdc1
Tmem97
Cop1
Lamtor4
Lamtor2
Gipc3
Timp3
Mmp13
Fbxw9
Rab7b
Tubal3
Rbis
Rgs19
Timp1
Fgf2
Sumo3
Zmiz2
Sirt6
9530068E07Rik
Gnb1
Jun
Ccnd1
Wdr53
Pdcl
Akt3
Lef1
Wdr77
Prpf40b
Snrpe
Olfr518
Pfn3
Map4k4
Nchp2
Anln
Meaf6
Ccnd3
Impdh2
Zdhhc8
Eno2
Traf6
Rab5if
Tmem147
Med30
Arid4a
Pdk2
Polr2f
Grin2d
Recql5
Ppl2
Zranb3
Il1rl1
Brf2
Rrn3
Mrps6
Dcxr
Grm1
Zbtb12
Alg8
Twistnb
Slc39a6
Myo1c
Shisa4
Grasp
Slc30a7
Anapc11
Nap1l4
Magt1
Myo3b
Tk2
Cyth2
Ostc
Kif1b
Uqcr10
Myo5a
Ndufb11
Ndufv3
Wdr4
Sdhd
Immt
Dynll2
mt-Cytb
Atp5md
Tmem109
Samm50
Ggact
Timm8a1
Nudt3
Sec31a

E

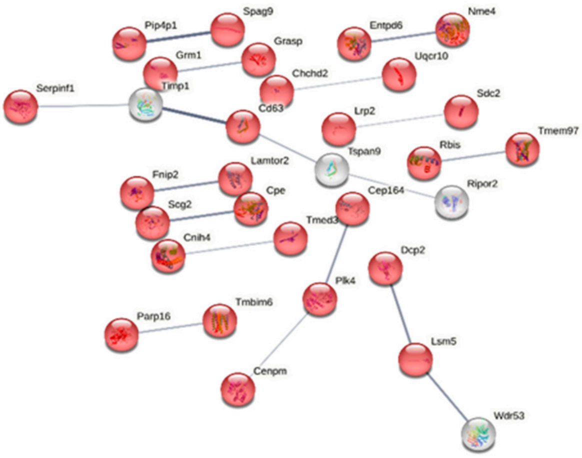
Spag9
Pip4p1
Grasp
Grm1
Entpd6
Nme4
Uqcr10
Chchd2
Serpinf1
Timp1
Cd63
Sdc2
Lrp2
Tmem97
Rbis
Tspan9
Lamtor2
Fnip2
Cpe
Scg2
Cep164
Ripor2
Tmed3
Cnih4
Dcp2
Plk4
Tmbim6
Parp16
Lsm5
Cenpm
Wdr53

F

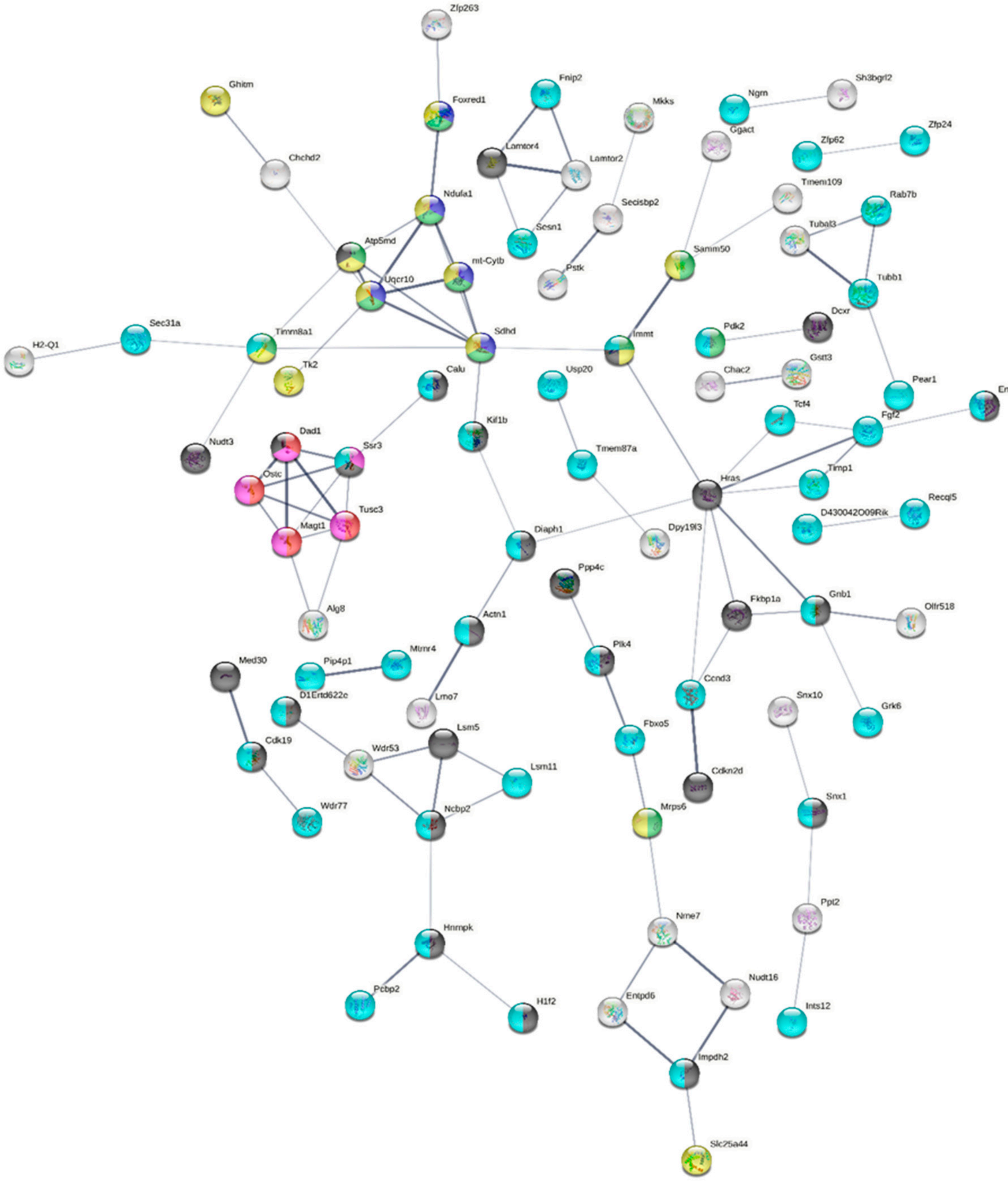
Zfp263
Ghitm
Foxred1
Fnip2
Mkks
Ngrn
Sh3bgrl2
Ggact
Zfp62
Zfp24
Chchd2
Lamtor4
Lamtor2
Tmem109
Rab7b
Ndufa1
Secisbp2
Atp5md
Sesn1
Tubal3
Samm50
mt-Cytb
Uqcr10
Pstk
Tubb1
Sec31a
H2-Q1
Timm8a1
Sdhd
Immt
Pdk2
Dcxr
Tk2
Calu
Usp20
Chac2
Gstt3
Pear1
Eno2
Nudt3
Dad1
Kif1b
Tcf4
Fgf2
Ssr3
Ostc
Tmem87a
Timp1
Hras
Tusc3
Recql5
D430042O09Rik
Magt1
Diaph1
Dpy19l3
Ppp4c
Alg8
Actn1
Fkbp1a
Gnb1
Olfr518
Med30
Pip4p1
Mtmr4
Plk4
D1Ertd622e
Lmo7
Ccnd3
Snx10
Grk6
Lsm5
Wdr53
Fbxo5
Cdk19
Lsm11
Cdkn2d
Snx1
Wdr77
Ncbp2
Mrps6
Ppt2
Hnrnpk
Nme7
Nudt16
Pcbp2
H1f2
Entpd6
Ints12
Impdh2
Slc25a44

Figure S1. Identity and interaction of proteins within the functional network that are altered 24h after proton exposure. STRING database was used to illustrate the interactions of the proteins that were altered by exposure to low (0.1 Gy), medium (0.5 Gy) and high (2Gy) doses in SW1353 and MC615. Edges represent both functional and physical protein associations, inferred based on confidence scores. The thickness of the connecting lines reflects the strength of the interactions, with a medium confidence score set above a 0.4 confidence threshold. (A) Proteins altered in SW1353 at 0.1 Gy are associated to metabolic process (red), mitochondrial inner membrane (blue), intracellular non-membrane-bound organelle (green) and protein-containing complex (yellow). Hub proteins in the network: COX6B1, MT-CO1, NDUFS6, COX15, COX14, UQCR11, MT-ND4, NDUFB9, RPL14 and NSA2.(B) Proteins altered inSW1353 at 0.5 Gy are associated with intracellular membrane-bounded organelle (green), Pachyonychia congenita and Epidermolysis bullosa simplex Dowling–Meara type (red) and Keratin type II head (blue). Hub proteins in the network: H3-2, H3C12, KMT2C, H2AC8, KRT5, KRT6B, RARA, KRT6A, KRT2, KRT74. (C) Proteins altered in SW1353 at 2 Gy are associated with organelle membrane (red). Hub proteins in the network: AR, ATXN3, MYSM11, PPP1R12C, DYNC1I2, PIP, RP9, HIBCH, KAT8, RARA. (D) Proteins altered in MC615 at 0.1 Gy are associated with myosin complex (green), catalytic complex (yellow), mitochondrial protein-containing complex (red), mitochondrial respirasome (blue), transport (cyan), acetylation (black), phosphoprotein (magenta). Hub proteins in the network: Jun, CCnd1, Fgf2, Thbs1, Timp3, MMp13, Timp1, Uqcr10, Ndufb11 and Sdhd. (E) Proteins altered in MC615 at 0.5 Gy are associated with intracellular membrane-bounded organelle (red) Hub proteins in the network: Timp1, Cd63, Tspan 9, Lsm5, Plk4, Parp16, Tmbim6, Spag9, Grm1, Tmem97. (F) Proteins altered in MC615 at 2 Gy are associated with oligosaccharyltransferase complex (red), mitochondrial protein-containing complex (lime green), mitochondrial inner membrane (yellow), mitochondrial respirasome (blue), acetylation (black), phosphoprotein (cyan), post-translational protein targeting the membrane and translocation (magenta). Hub proteins in the network: Tusc3, Magt1, Ssr3, Ostc, Dad1, Sdhd, Uqcr10, Ndufa1, Hras and Atp5md.
